# Delivering low-intensity psychological interventions in low- and middle-income countries: A systematic review and meta-ethnographic synthesis of lay health workers’ experiences

**DOI:** 10.1017/gmh.2026.10271

**Published:** 2026-07-07

**Authors:** Aditya Putra Kurniawan, Prima Agustia Nova, Gading Ekapuja Aurizki, Herni Susanti, Rebecca Pedley, Penny Bee, Helen Brooks

**Affiliations:** 1 Division of Nursing, Midwifery, and Social Work, The University of Manchester Faculty of Biology Medicine and Health, UK; 2 Psychology, Universitas Mercu Buana Yogyakarta, Indonesia; 3 Faculty of Nursing, Universitas Indonesia, Indonesia; 4 Division of Nursing, Midwifery and Social Work, The University of Manchester, UK; 5 Faculty of Nursing, Universitas Airlangga, Indonesia

**Keywords:** anxiety, depression, mental health, health policy, healthcare workers

## Abstract

Task-sharing psychological treatment to non-specialist providers, including lay health workers (LHWs), is central to WHO guidance in low- and middle-income countries (LMICs). LHWs are community members without formal mental health qualifications who are trained to deliver low-intensity psychological interventions (LIPIs) for depression and anxiety. Although trials show LHW-delivered LIPIs can reduce symptoms, less is known about LHWs’ experiences or the relational and emotional demands of delivery. This review synthesised qualitative evidence on LHWs’ experiences of delivering LIPIs in LMICs. We searched CINAHL, Embase, PsycINFO, MEDLINE, Cochrane Central, ASSIA and ProQuest Dissertations & Theses Global to June 2025, supplemented by grey-literature searches. We included qualitative, mixed-methods studies reporting interview or focus group data from LHWs delivering LIPIs for adults with depression and/or anxiety. Data were synthesised using meta-ethnography; quality and confidence were assessed using CASP and GRADE-CERQual. Thirty-three publications representing 28 studies from Africa, Asia and Latin America were included. Four themes were generated: doing more than psychological therapy; embedded in the community yet precariously held by the system; growing in the role; and the hidden cost of caring. Task-shifting redistributes not only technical tasks but also relational and emotional labour to LHWs, requiring support through training, supervision, safety and remuneration.

## Graphical abstract

**Figure:**
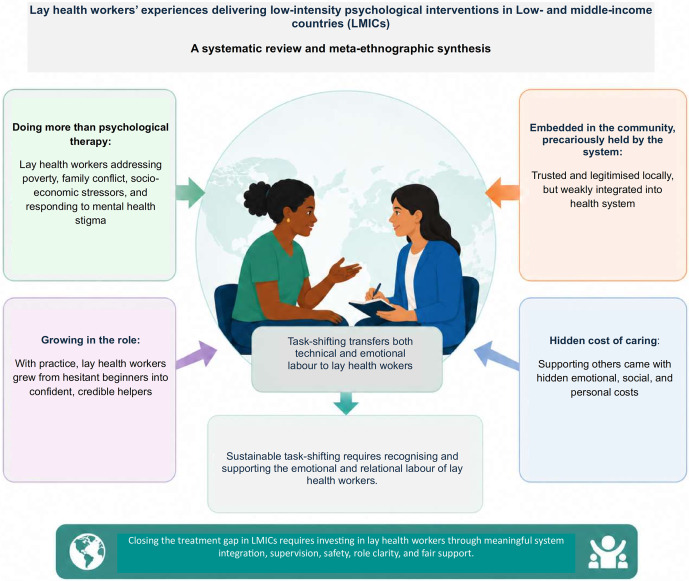

## Impact statement

In response to the severe shortage of mental health specialists in low- and middle-income countries (LMICs), the WHO and national governments have promoted task-shifting, delegating psychological care to trained non-specialist providers, including lay health workers (LHWs). These workers are often community members without formal specialist mental health qualifications who deliver brief psychological interventions for depression and anxiety with focused training and supervision. Most research on task-shifting in LMICs has focused on intervention effectiveness and implementation, with far less attention to how LHWs themselves experience delivering care. Our systematic review and meta-ethnography synthesise qualitative evidence from multiple LMICs and show that this work depends not only on technical competence, but also on substantial relational and emotional labour. LHWs often move beyond manuals to respond to poverty, family conflict, stigma and risk, while managing their own distress, boundaries and safety. These hidden costs of care are not only emotional but also structural, shaped by working conditions, weak recognition, limited resources, supervision and organisational support. Efforts to scale up lay-delivered mental healthcare should therefore go beyond strengthening training and supervision alone. They should also recognise relational and emotional labour as core components of service delivery and embed this recognition in fairer remuneration, role clarity, protected time, safeguarding, supportive supervision and stronger organisational support.

## Introduction

Depression and anxiety are major global health concerns and disproportionately affect low- and middle-income countries (LMICs), as classified by the World Bank based on gross national income per capita (World Bank, 2024). An estimated 5.53% of adults in LMICs live with depression and 4.52% with anxiety, conditions that frequently co-occur (Saha et al., 2021; WHO, 2022). Despite the burden, many people in LMICs face substantial barriers to accessing care, including limited mental health literacy, stigma, poor availability of services and lack of perceived need for therapy (Andrade et al., 2014; Saha et al., 2021; Roberts et al., 2022).

These challenges are exacerbated by a severe shortage of mental health professionals (Eaton et al., 2011; Patel et al., 2011; WHO, 2021). LMICs have only 3.8 mental health workers per 100,000 population compared with 72.7 in high-income countries (WHO, 2021), and just 13.7% of people with depression and anxiety receive condition-appropriate care (Evans-Lacko et al., 2018). In response, WHO has promoted task-shifting, which involves transferring routine tasks from specialists to trained non-specialists, including lay health workers (LHWs) (Liu et al., 2011; WHO, 2012). When supported by structured training and supervision, task-shifting can improve health outcomes, reduce specialist workload and expand access to early psychological care, which can prevent more severe illness and costly hospital care (Kakuma et al., 2011; Seidman and Atun, 2017; Heller et al., 2019). The WHO’s Mental Health Gap Action Programme (mhGAP) provides guidance and training materials to support these roles (Kohrt et al., 2018; WHO, 2019).

A key application of task-shifting is training LHWs to deliver low-intensity psychological interventions (LIPIs): brief, structured psychological interventions delivered by trained non-specialists with focused training and supervision. Many LIPIs draw on CBT principles, including thought challenging, behavioural activation, problem-solving and psychoeducation, and are designed to reduce distress and improve daily functioning (Bennett-Levy et al., 2010; Shafran et al., 2021). Many also adopt a transdiagnostic framework, targeting shared psychological processes across depression, anxiety and related forms of distress rather than a single diagnosis alone (McManus et al., 2010; Dalgleish et al., 2020). This review focused on depression and anxiety because they are major common mental health problems and central targets of task-shared psychological care in LMICs, and because many included interventions addressed shared processes across depression, anxiety and related distress (Karyotaki et al., 2022; Kim et al., 2023). LIPIs also represent scalable strategies for addressing the treatment gap in resource-constrained settings (Patel et al., 2011; WHO, 2019).

In this review, LHWs are defined as non-specialist providers without formal specialist mental health qualifications who are trained to deliver LIPIs (Lewin et al., 2005; WHO, 2013). Depending on context, they include community volunteers, peers, lay counsellors and other community-based workers, often drawn from and embedded within the communities they serve, and, in some cases, linked to existing health or NGO services and may have had broader health-related roles. Examples include grandmothers delivering problem-solving therapy in Zimbabwe and local mothers (“ *Sakhis* ”) supporting perinatal mental health in India (Chibanda et al., 2017; Singla et al., 2020). Their roles often extend beyond formal counselling to include psychosocial support, health promotion and community mobilisation (Hartzler et al., 2018; Glenton et al., 2021; Liana and Windarwati, 2021).

Systematic reviews have demonstrated that LHW-delivered LIPIs can reduce symptoms of depression and anxiety in LMICs (Singla et al., 2017; Connolly et al., 2021; van Ginneken et al., 2021), yet evidence on how these interventions are experienced by LHWs remains limited. Existing reviews have explored feasibility, acceptability and training needs (Shahmalak et al., 2019; Verhey et al., 2020), or programme reach (Ahmed et al., 2022), and the perceived costs of task-shifting within health systems (Coales et al., 2023), highlighting that success depends not only on technical competence or adherence to manuals but also on training, supervision, cultural sensitivity and organisational support. However, these reviews remain largely programme-centred, and have not foregrounded LHWs’ lived experiences as they navigate cultural expectations, organisational constraints and social relationships in under-resourced settings. LHWs often rely on local understandings of mental health, community norms and personal relationships rather than formal diagnostic categories (Chase et al., 2024). Their roles are deeply rooted in social and cultural contexts (Chase et al., 2024). In many such settings, mental health initiatives rely more on community engagement, social trust and local networks than on formal systems (Chutiyami et al., 2025). In this context, the human dimensions of LHW work, their experiences, including emotional demands, relationships with patients, work–life balance and personal growth shape both their well-being and their capacity to deliver high-quality care (Padmanabhanunni 2020; Draper et al., 2024; Sangraula et al., 2024), yet these aspects have not been widely explored in LMICs.

As task-shifting to LHWs expands globally and they become a critical workforce in LMIC health systems, understanding their lived experiences is increasingly urgent. Their challenges directly affect intervention quality, burnout, retention and the sustainability of community-based mental health programmes. Attending to these experiences is essential for supporting current LHWs and developing future generations of community mental health workers. This review synthesises qualitative evidence on how LHWs experience delivering LIPIs for depression and anxiety in LMICs. It was guided by the main review question, what are the experiences of LHWs delivering these interventions in LMICs?, and by a more focused sub-question examining what psychosocial, emotional, cultural and organisational factors shape LHWs’ delivery of LIPIs.

## Methods

We followed Preferred Reporting Items for Systematic Reviews and Meta-Analyses (PRISMA) guidelines (Moher et al., 2009). The protocol is registered in PROSPERO (CRD42023459609).

### Eligibility criteria

The SPIDER tool (Sample, Phenomenon of Interest, Design, Evaluation, Research Type) guided eligibility criteria (Cooke et al., 2012). We included qualitative studies and qualitative components of mixed-methods studies reporting interview-based findings from process or trial-embedded evaluations of LHWs’ experiences delivering LIPIs for depression and/or anxiety in World Bank–classified LMICs (2022–2023) (World Bank, 2024). For the purposes of this review, LHWs were defined as non-specialist providers delivering the primary intervention without formal mental health qualifications. This included community volunteers, peers, lay counsellors and other community-based workers, some of whom were linked to existing health or NGO services and may have had broader health-related roles. LIPIs were defined as brief, structured psychological interventions delivered by trained non-specialists with focused training and supervision, typically involving less therapeutic input than conventional therapy and often drawing on CBT-informed, behavioural, cognitive, problem-solving or psychoeducational approaches. Studies were eligible if they involved adults (≥18 years). Mixed-age studies were eligible when adult-focused data predominated; mixed-provider studies when LHW-specific data were identifiable; and mixed-diagnosis studies when the intervention and qualitative findings were primarily relevant to depression, anxiety or closely related common mental distress targeted through the included LIPI. Mixed physical-mental health studies were eligible when the intervention delivery and qualitative findings focused primarily on depression and/or anxiety outcomes. Studies were excluded where this distinction could not be made with sufficient clarity. No language and date restrictions were applied. We excluded studies conducted outside LMICs, studies focused solely on severe mental disorders, interventions not described as a defined psychological intervention, papers without qualitative data and studies without accessible full texts (see Supplementary material 1 for full eligibility criteria).

### Search strategy

We searched electronic bibliographic databases, CINAHL, Embase, PsycINFO, Medline, Cochrane Central, ASSIA and ProQuest Dissertation & Theses Global, from inception to January 22, 2024, with an update on June 17, 2025. Search strings combined terms for the sample (e.g., “lay health worker,” “community health worker”), phenomenon of interest (e.g., “depression,” “anxiety,” “CBT”), design (e.g., “interview,” “focus group”), evaluation (e.g., “experiences,” “perceptions,” “views”) and research type (e.g., “qualitative,” “mixed-methods”). Terms were informed by prior systematic reviews (van Ginneken et al., 2013; Shahmalak et al., 2019; Coales et al., 2023) and developed in consultation with an academic librarian, then adapted to database-specific syntax and controlled vocabularies (e.g., MeSH in MEDLINE). Supplementary searches included WHO and IHME websites, Google Scholar and backward and forward citation tracking (see Supplementary material 2 for full strategies).

### Selection process

The screening process was conducted using Covidence (Veritas Health Innovation, Melbourne, Australia), enabling independent duplicate screening of titles, abstracts and full texts. Each record was screened by two of three reviewers (APK, PAN and GEA). Inter-rater reliability was assessed using Cohen’s Kappa. For title and abstract screening, Kappa values were 0.50 (APK–GEA) and 0.30 (APK–PAN); for full-text screening, agreement improved to 0.60 (APK–GEA) and 0.40 (APK–PAN). Lower agreement at the title and abstract stage likely reflected the breadth and inconsistent reporting of LHW roles, intervention terminology and study focus in the literature. To minimise the risk of prematurely excluding potentially relevant studies, uncertain records were retained for full-text screening. Discrepancies were resolved through discussion, with a third reviewer adjudicating where needed. Any remaining unresolved cases were reviewed with the senior reviewer (HB) for a final decision.

Following initial screening, the operational application of the eligibility criteria was refined in relation to mixed-condition studies to ensure that included interventions and qualitative findings were primarily relevant to depression, anxiety or closely related common mental distress targeted through the included LIPI. This led to four included studies being returned to full-text screening for reappraisal. Following eligibility reassessment and author contact, two studies were excluded from the final synthesis because the populations were not sufficiently relevant to the review focus. The impact of these exclusions on theme development and synthesis interpretation is detailed in Supplementary material 7.

### Data extraction

Two standardised extraction forms were developed following meta-ethnography guidelines (Noblit and Hare, 1988). APK extracted descriptive study data (aims, participants, setting, methods, key findings), while qualitative data (first- and second-order constructs) were double-extracted by APK and PAN, reconciled and organised into preliminary conceptual categories in Excel. For studies involving mixed diagnostic groups or broader common mental disorder populations, extraction focused on LHW delivery of interventions targeting depression, anxiety or related common mental distress. Broader mental health terminology, screening categories and local idioms of distress were accepted without requiring explicit diagnostic labels and any uncertainties were noted and interpreted cautiously during synthesis.

### Quality appraisal

We appraised study quality using a modified Critical Appraisal Skills Programme (CASP) tool that included assessment of authors’ theoretical foundations (Long et al., 2020). For each study, we recorded domain-level CASP judgements and wrote a brief narrative summary of key methodological strengths and limitations (Long et al., 2020). Two reviewers (APK and PAN) conducted the appraisal; 30% of studies were double-appraised, and the remainder reviewed by one and verified by the other. No studies were excluded, in line with guidance that limited reporting does not necessarily indicate poor quality (Dixon-Woods et al., 2004; Dixon-Woods et al., 2007; Atkins et al., 2008). All studies were retained to preserve contextual diversity, and methodological strengths and limitations were incorporated into the synthesis (Carroll et al., 2012). Decisions were documented in Excel. We also applied Grading of Recommendations Assessment, Development and Evaluation–Confidence in the Evidence from Reviews of Qualitative research (GRADE-CERQual) to assess confidence in each key finding.

### Synthesis

We conducted a meta-ethnographic synthesis following Noblit and Hare (1988). First-order constructs (participants’ accounts) and second-order constructs (study authors’ interpretations) were identified and compared across studies. Reciprocal translation involved comparing conceptually similar findings and translating them into shared analytic concepts. For example, accounts of using personal hardship to empathise with clients, together with authors’ interpretations of self-disclosure, empathy and emotional triggering, were translated into caring through shared pain. Related accounts of emotional suppression during sessions, sacrifice of personal time and neglect of one’s own well-being were translated into when caring consumes the self. These translated concepts contributed to the third-order construct. The hidden cost of caring, and, across themes, informed an overarching line-of-argument synthesis about LHWs’ roles in community-based psychological care. (see Table 1; Supplementary Material 5).

**Table 1.** Example of synthesis process Table 1. long description.

First-order construct	Second-order construct	Translation	Third-order construct
*When they were telling their problems, I used to tell them that I had similar circumstances, but God has helped me to overcome it. Time never stays the same, I was stressed when my children were young, now my tension is gone. Mothers used to benefit from my experiences. IDI–33PV (Atif, 2015)*	**Experience of being a mother** (Atif, 2015) All PVs, except one, were mothers themselves and about a quarter revealed that they had experienced perinatal depression. These experiences provided the PVs with good understanding of the mothers’ issues and the ability to empathise with them.	**Caring through shared pain** The study by Atif (2015) demonstrated how LHWs used self-disclosure based on their own experiences to empathise with patients and build strong relationships. By sharing personal experiences, LHWs created a foundation of trust and relatability. Similarly, Chibanda et al. (2017) showed that LHWs used self-disclosure by sharing experiences, such as living with HIV, to show empathy and further encourage patients to open up emotionally. Likewise, Pachichana-Quinayáz et al. (2016) highlighted the role of empathy in building patient trust, showing shared traumatic experiences with patients. This approach, using self-disclosure as an “empathy hook,” was seen as a valuable way to engage patients in therapy. Ahmed et al. (2024) further explored how LHWs’ shared experiences with clients created both emotional closeness and internal strain where clients’ problems mirrored their own struggles. One LHW acknowledged that certain client narratives “hit close to home,” making it difficult to remain unaffected. Despite this, LHWs want to stay objective and continue providing support, even when it was personally triggering.	**The hidden cost of caring** Caring through shared pain: Shared pain and social proximity helped LHWs build trust and therapeutic connection, yet this same closeness left them emotionally vulnerable, as others’ suffering often resonated with their own lives. When caring consumes the self: To remain available and therapeutically present, LHWs often suppressed their own emotions, sacrificed personal time and repeatedly prioritised clients’ needs over their own well-being, creating a gradual pattern of self-neglect. When the work never ends: Counselling was layered onto existing community, domestic and livelihood responsibilities, making care feel continuous and difficult to contain within formal work boundaries. Care in risky and stigmatised contexts Gendered expectations, harassment, stigma and cultural or religious resistance intensified the emotional and relational burden of care.
*Most of us are also displacement victims, then it is also very easy to understand, I think I know how to create empathy with people and that gives them confidence (Pacichana-Quinayáz et al., 2016)*	**Characteristics and LPCWs performance (**Pacichana-Quinayáz et al., 2016) Respondents mentioned an important point within the CETA intervention: In a first contact, LPCW must make an effective “hook” which allows one to determine if participants are interested in attending therapy. Many agreed that LPCW empathy was one of the main strengths, developed because of their traumatic experiences.		
*There are scenarios where my client will be talking about [a] problem that I am basically going through right now. But when I try to refer him to another counsellor, he does not want that other counsellor. He wants specifically to see me. (Ahmed et al., 2024)*	**Counsellor Psychological Well-being** (Ahmed et al., 2024) Counsellors commonly reported challenges with experiencing the same hardships as their clients which can compromise their ability to provide problem-solving therapy.		
*“I can make an excuse to go drink water” (Oratile) and “I had to pretend like I wasn’t going through what [the client] is going through” (Ahmed et al., 2024)*	**Counsellor Psychological Well-being** (Ahmed et al., 2024) Counsellors mentioned ways in which they tried to conceal their emotions during the counselling sessions. These emotionally triggering experiences can make it difficult for counsellors to continue with the therapy sessions and may therefore prompt an unwanted referral to another counsellor	**When caring consumes the self** LHWs’ accounts suggest that caring often became self-consuming, requiring them to suppress their own emotions during sessions, sacrifice personal time and repeatedly prioritise clients’ needs over their own well-being. Ahmed et al. (2024) show how some LHWs had to conceal their own emotional distress in order to remain composed during counselling. One worker described making an excuse “to go drink water” to manage overwhelming feelings, while also “pretend[ing] like I wasn’t going through what [the client] is going through,” highlighting the strain of maintaining therapeutic presence while holding back personal emotion. This emotional containment was part of a wider pattern in which care extended beyond the session itself. In Wood et al. (2021), a female LHW described the cumulative demands of travel, counselling and administrative work as overwhelming: “You are thinking as they are talking… it is too much, very horrible,” and characterised the role as “emotionally taxing,” suggesting that emotional and cognitive strain were embedded in the work itself. **When the work never ends** LHWs experienced significant workload strain as a result of integrating psychological interventions into their already full schedules. In the study by Jacobs (2020), designated counsellors described how limited staffing meant the absence of one team member led to additional burdens on others: “we are three and the one is being booked for Project MIND, the pressure is on the other two.” Similarly, these LHWs juggled dual responsibilities delivering Project MIND while continuing to assist in psychiatric clinics highlighting blurred boundaries between intervention roles and routine duties. **Caring under gendered risk** Several studies illustrate how entrenched gender norms and social stigma surrounding mental health present significant challenges for lay health workers (LHWs) in delivering psychological interventions. In the study by Wood et al. (2021), LHWs reported facing resistance from their own families due to prevailing gender expectations, such as the notion that daughters-in-law should not work outside the home. Additionally, LHWs, particularly women, faced gender-based harassment from male patients, which created a hostile and unsafe environment for intervention delivery.	
*Because [during counselling] you are thinking as they are talking. And travelling is so much. After doing all this you come to [the facility], you sit and some other work of typing…So it is too much, very horrible. This I did not like…Yes, it is too much, yes. Work is often emotionally taxing. (LHW, Female, 37 years) (Wood et al., 2021)*	**Workload** (Wood et al., 2021) Many LHWs spoke of feeling burdened by their workload, while a few said that they felt motivated despite having lots of work.		
*The challenge is always like coming here and also rescheduling issues… because maybe I will come here knowing my client will be here at two o’clock and then at half past two they will be telling me ‘no, I have family issues’… and then we reschedule to a different date [to] accommodate my client with me now, and I have to make sacrifices for my client because I understand it’s one of the things that we were told about. That sometimes you are going to have difficulties that you are going to invade some of the personal, private time”. (Ahmed et al., 2024)*	**Scheduling Procedures** (Ahmed et al., 2024) Several counsellors implied that the initial procedures used to schedule clients for counselling sessions were barriers to Safe Haven implementation. At the beginning of the pilot, when counsellors were scheduling their clients by appointment only, clients were missing their scheduled appointments or attending late		
*We forgot to take care of ourselves as counsellors. We put a lot of focus on the clients. We have our own problems and need to heal first before we help other people… (Counsellor 4) (Munodawafa et al., 2017)*	**Implementation** (Munodawafa et al., 2017) Three counsellors mentioned how providing emotional support to mothers reminded them of their own problems and the other three mentioned how counselling participants was emotionally taxing for them.		
*“It is very important, because before I used to take on the whole world. Now, I know that as I care for my client, I must care for myself, because if I do not care for myself, I am going to burn out.” (ID18, designated counsellor). (Jacobs et al., 2021)*	**Counsellors’ Perceptions of Clinical Supervision and Debriefing** (Jacobs et al., 2021) FBCs also valued the regular debriefing they received as part of their weekly supervision session, particularly in relation to prioritising self-care and preventing burnout. Regular opportunities for debriefing created a “safe space” to discuss difficult patient cases, personal issues that may be impacting on their counselling, issues around self-care and strategies for coping with stress:		
*“At the beginning it was fine but as time went by it was such a lot of people we had to see and we had to see our work as well. So, it was tiring, because an hour to sit with a person is a lot when you know my colleagues need to see now maybe 20 or 30 more people because I am busy in this project.” (ID11, designated counsellor). (Jacobs et al., 2021)*	**Impact of the intervention in their work routine at the UBS (Institutional impact)** (Henrique et al., 2021) The main complaint was related to the difficulty of managing the time to perform the routine tasks at the UBS with psychosocial sessions at home, all within working hours. The home sessions were added to their other tasks at the UBS.		
*“When I discussed about working for this [program], at that time, my mother-in-law was alive. She stopped me. [She said,] “In our family, our daughters-in-law do not work.” (LHW, Female, 45 years) (Wood et al., 2021)*	**Social context (Gender)** (Wood et al., 2021) Social and cultural norms negatively impacted LHWs’ work in a variety of domains, most prominently gender, social class, religion and stigma about mental illness.		
*”For me, the person I’m seeing is unwell, I don’t [care] whether it is men or ladies. But, things like that, he [the patient] pinched my lip and kissed. Those kinds of problems are there. (LHW, Female, 27 years) (Wood et al., 2021)"*	**Social context (Gender)** (Wood et al., 2021) Female LHWs spoke about gender-based harassment and feeling unsafe or uncomfortable when performing their work, especially when travelling to remote areas or conducting home visits with male patients.		

## Results

**Figure 1.**
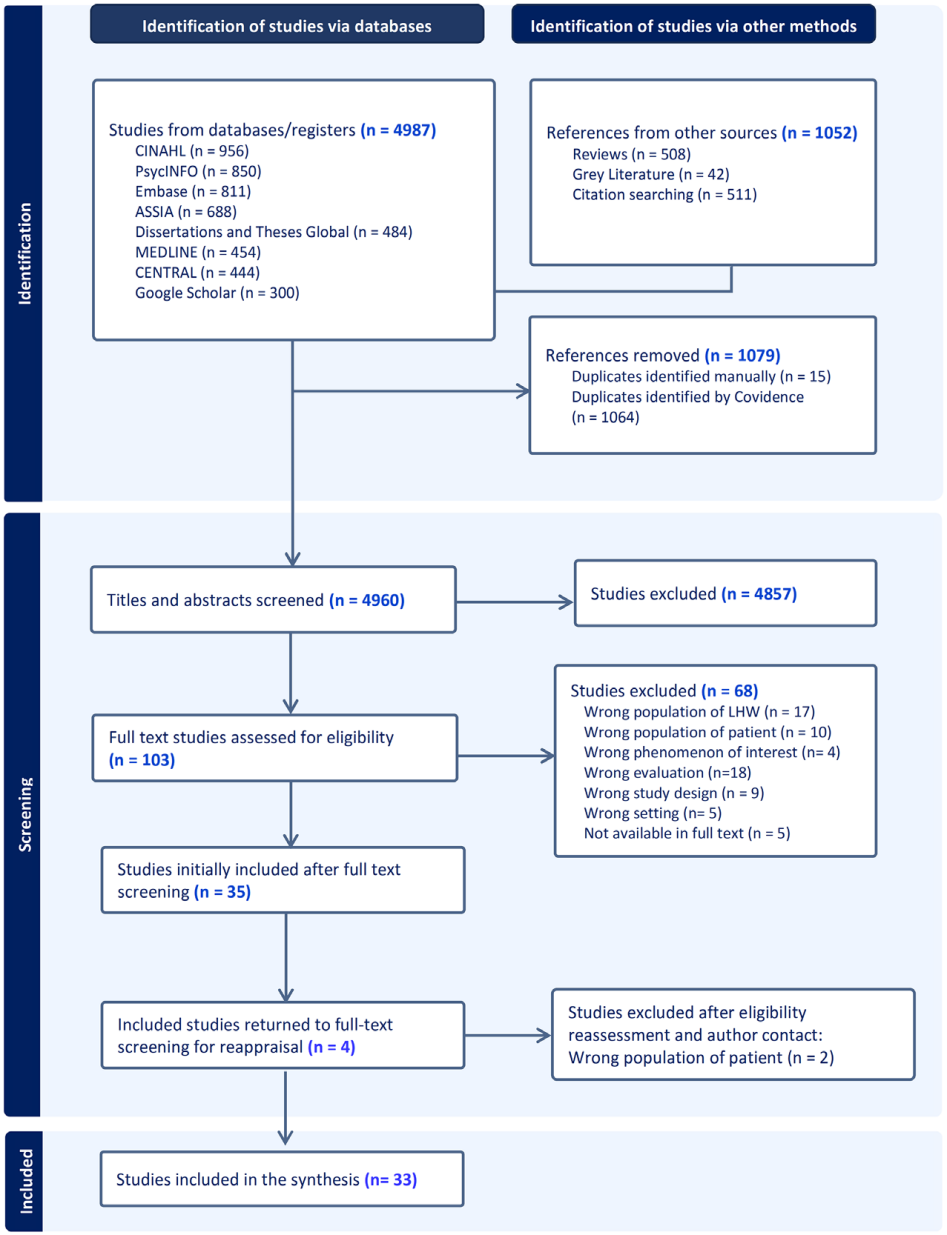
PRISMA flow diagram of study selection. Figure 1. long description.

A total of 4,987 records were identified through eight database searches and 1,052 through other sources. Following screening and eligibility reappraisal, 33 studies were included in the final synthesis (Figure 1). Twelve of these appeared in multiple formats (e.g., theses and journal articles) (Atif, 2015; Atif et al., 2016; Chibanda, 2016; Nyatsanza et al., 2016; Atif et al., 2017; Chibanda et al., 2017; Munodawafa et al., 2017; Munodawafa, 2018; Jacobs, 2020; Chau et al., 2021; Jacobs et al., 2021; Chau et al., 2024). To avoid duplication, each set was treated as a single study, prioritising theses for methodological detail and using journal articles to supplement information, resulting in five unique studies. In total, 28 unique studies were included in the final synthesis.

### Characteristics of studies

Studies spanned multiple LMICs, grouped by World Bank region. Sub-Saharan Africa contributed 53.6% of studies, primarily from South Africa, Zimbabwe and Kenya, followed by South Asia with 32.1% from India and Pakistan. Latin America accounted for 10.7% (Brazil, Colombia, Mexico), and Southeast Asia was represented by a single study from Vietnam (3.3%) (Table 2). Most studies were conducted in lower-middle-income countries (53.16%; Zimbabwe, Kenya, India, Pakistan, Vietnam), with 35.7% from upper-middle-income countries (Botswana, Brazil, Colombia, Mexico, South Africa) and 10.7% from low-income countries (Ethiopia, Malawi, Sierra Leone) (Table 3; Supplementary Material 3).

**Table 2.** Distribution of studies by country, region and income classification Table 2. long description.

Region	Countries	Income classification	Total of studies	% of total studies
Sub-Saharan Africa (53.6%)	South Africa	Upper-middle income	6	21.4%
	Zimbabwe	Lower-middle income	3	10.7%
	Kenya	Lower-middle income	2	7.1%
	Ethiopia	Low income	1	3.6%
	Botswana	Upper-middle income	1	3.6%
	Malawi	Low income	1	3.6%
	Sierra Leone	Low income	1	3.6%
South Asia (32.1%)	India	Lower-middle income	7	25%
	Pakistan	Lower-middle income	2	7.1%
Latin America (10.7%)	Brazil	Upper-middle income	1	3.6%
	Colombia	Upper-middle income	1	3.6%
	Mexico	Upper-middle income	1	3.6%
Southeast Asia (3.6%)	Vietnam	Lower-middle income	1	3.6%

**Table 3.** Characteristics of studies Table 3. long description.

Author (s)	Countries	Design, data collection and analysis	Number of participants (LHWs)	Gender and age range of LHWs	Intervention target	Interventions	Background of LHW and organisational linkage	Key findings
Abas et al. (2016)	Zimbabwe	Mixed-methods study (qualitative component extracted), Routine data on patients’ attendance, FGDs Quantitative analysis and thematic analysis	40–60	Female, (Mean age: 62.	Depression	Problem-solving therapy (PST) with components of behaviour activation	Community health worker, Community-based Primary care-linked (government-employed)	Positive reception: The collaborative care intervention for CMDs was well-received by patients. Rewarding for LHWs: LHWs found the intervention to be personally rewarding. Cost-effectiveness: The intervention proved to be cost-effective over time.
Ahmed et al. (2024)	Botswana	Qualitative design Interviews Thematic analysis	8	Mixed gender (range of age 21–28, Mean = 24)	Depression and anxiety	PST, behavioural activation, psychoeducation	Peer provider Project-employed / trial-employed Facility-based delivery	Barriers to implementation of Friendship Bench Client reticence and confidentiality concerns Parental disapproval Limited accessibility Counsellor psychological burden Scheduling conflicts Inadequate financial resources Facilitators of Implementation Peer delivery increased acceptability Strong counsellor commitment and perceived value
Atif (2015)* Atif et al. (2016)* Atif et al. (2017)*	India, Pakistan	Qualitative design, interviews, framework analysis	8	Female, (range: 26–40 years, Mean: 33)	Perinatal depression	Core principles and techniques of CBT, such focusing on the here and now, behaviour activation and problem-solving	Peer volunteer Community-based Primary care-linked	Factors enhancing LHWs’ acceptability: Local presence and community reputation Empathy and trustworthiness Connection to primary healthcare Acceptability of the intervention: Use of vignettes, pictures and simple language made the intervention easy to understand. Peers effectively used behavioural activation techniques. Shared life experiences facilitated the intervention’s delivery.
Bah et al. (2025)	Sierra Leone	Mixed-methods study (qualitative component extracted)PHQ–9, Sierra Leone Perinatal Distress Scale, WHO–5 well-being index Descriptive statistic, t-Test, thematic analysis	10	Female, age was not clearly described	Perinatal depression and postpartum depression	Problem-solving therapy (PST)	Community-based volunteer Community-based Community health system-linked	The feasibility, acceptability and preliminary effectiveness of problem-solving therapy (PST) by trained mothers Feasibility High recruitment and retention Lay counsellor delivery was practical: Strong implementation in home and group settings Acceptability Positive reception by participants and counsellors: Cultural relevance of adapted content: Strengthened therapeutic alliance: Preliminary effectiveness Reduction in psychological distress Improved problem-solving skills and social support
Burgess et al. (2025)	Ethiopia	Qualitative design Interviews Thematic analysis	4	Female (range of age 25–50)	Postpartum depression and anxiety	Simplified CBT-based programme combined with family planning counselling	Community health worker Health-post-based Community-linked	Intervention was acceptable to postpartum women; content was engaging and relatable. Group sessions reduced isolation and encouraged peer support. Health extension workers feasibly delivered simplified CBT after short training, though additional coaching was needed. Participants reported better emotional coping, increased motivation to seek family planning (FP) and improved spousal communication. Time constraints and limited training were key feasibility challenges. Some religious barriers to FP remained unaddressed.
Chau et al. (2021) Chau et al. (2024) *	Vietnam	Qualitative design Interviews Thematic analysis	47	Mixed gender (78.7% were women), Mean of age 55 (48.97% were over 55)	Depression	Behavioural activation, problem-solving, psychoeducation, linkage to psychosocial services	Lay social worker Community-based, Social-service-linked (health–social sector hybrid)	A strong relationship between patients and LHWs fosters openness and responsiveness. LHWs manage programme mobilisation, conduct home visits and provide patient support. Involvement of the community enhances the effectiveness of the intervention. Older LHWs face difficulties in learning new knowledge. Low educational levels among LHWs reduce patient trust. LHWs balance their work duties with personal responsibilities, such as harvesting.
								Social embeddedness enhanced acceptability of the intervention delivered by social collaborators. Social collaborators as bridges, acting as connectors between clients and formal health services, especially for people unfamiliar with or hesitant to seek professional care.
Chibanda (2016) * Chibanda et al. (2017) *	Zimbabwe	Qualitative design Interviews Thematic analysis	7	Female (Mean of age: 58)	Depression and other common mental disorders, particularly for those living with HIV/AIDS	PST with components behavioural activation	Community health worker Facility-based primary care (government-employed) Community-linked	The study identified the following benefits of working with social collaborators: Increased awareness of mental health in the family and community. Reduced stigma; A better understanding that depression is treatable. Increased help-seeking; and Improved access to care.
								Locally adapted problem-solving therapy (PST): The PST was adapted based on local concepts from LHWs and clients. It remains user-friendly while aligning with traditional PST objectives, like the Friendship Bench model. The first session is crucial due to the high mobility of clients. It is important for those delivering the intervention to reside locally for better accessibility and continuity.
Davies et al. (2022)	South Africa	Qualitative design Interviews, counselling transcripts Grounded theory	6	Female, (range of age: 28–46, Mean = 37.2)	Perinatal depression	Psychoeducation, PST, behavioural activation, cognitive reframing and relaxation training	Community health worker NGO-based Delivering a facility-linked trial intervention	Challenges to intervention effectiveness: Socio-economic difficulties faced by participants limited the effectiveness of the intervention. Discrepancies between the counselling strategies used and the intervention manual were also a factor. Reported benefits: Participants reported improvements in mood and cognition. These gains were attributed to therapeutic elements that aligned with the previously identified “common elements” of therapy.
Henrique et al. (2021)	Brazil	Qualitative design Interviews Thematic analysis	11	Female, (range of age: 29–60 years, Mean: 38)	Depression	Behavioural activation, psychoeducation, psychosocial intervention	Community health worker (nurse assistant) Primary care-based (government-employed) Delivering a home-based psychosocial intervention	The PROACTIVE intervention was feasible and well-received by both health workers and older adults. Qualitative assessments indicated a need for improved training and supervision to ensure better adherence to the intervention protocol.
Jacobs (2020) * Jacobs et al. (2021) *	South Africa	Qualitative design Interviews Framework analysis	18	Female = 13 (72.2%) Male = 5 (27.8), (age range 28–55, Mean: 40)	Common mental disorders, specifically depression or problem alcohol use	PST, motivational interviewing, psychoeducation on depression and alcohol, how to cope with negative thought	Facility-based counsellor Primary healthcare facility-based Designated or dedicated within chronic disease services	Barriers to intervention delivery: Competing tasks, low pay, lack of space and privacy concerns were significant barriers to implementation. Facilitators: Seeing patient improvements and using the intervention to address personal issues facilitated implementation. Regular supervision and debriefing sessions were helpful. Role-playing enhanced skills, while audio-recorded feedback was feasible but faced logistical challenges.
Joag et al. (2020)	India	Mixed-methods study (qualitative component extracted) Pre–post questionnaires, interviews Logistic regression, thematic analysis	16	37 males and 28 females, (range of age: 28–45 years)	Common mental disorders, including depression and anxiety	Problem-solving, behavioural activation, psychoeducation on healthy lifestyle behaviours, as well as facilitating access to social benefits	Community volunteer Community-based Community-led intervention with referral linkage	Effectiveness of community-led interventions: Community-led interventions with local volunteers can successfully facilitate access to social welfare benefits. The intervention taps into community social capital to engage and empower members in addressing mental health issues.
Kidia et al. (2024)	Zimbabwe	Qualitative design Interviews Thematic analysis	12	Male = 1, Female = 11, age was not specified	Common mental disorders, including depression and anxiety	PST and income generating components	Community health worker Community-based, NGO-implemented with government support Rural health clinic-linked	Positive feedback on the Friendship Bench intervention. Issues of accessibility were highlighted. Feasibility: While acceptable, the intervention was not deemed feasible or sustainable in its current form. Considering the sociocultural context is crucial when developing mental health interventions in rural areas.
Munodawafa et al. (2017) * Nyatsanza et al. (2016) * Munodawafa (2018) *	South Africa	Qualitative design Interviews Thematic analysis	6	Female, (range of age 28–46 years, Mean: 37.2)	Perinatal depression	Psychoeducation, PST, behavioural activation, cognitive reframing (healthy-thinking) and relaxation training	Lay community counsellor NGO-based Delivering a facility-linked and home-based trial intervention	Facilitating factors of treatment delivery: The intervention content was effective in delivery. Continuous training and supervision supported successful implementation. Adherence to a structured counselling manual was key. Conducting sessions in the local language, isiXhosa, was beneficial. Confident and motivated counsellors contributed to success. Older age, commitment and desire for help among participants facilitated intervention delivery. Barriers to Delivery: Poverty, crime and lack of space for sessions hindered the intervention. Participants’ young age, the nature of their problems and avoidance of counsellors posed challenges. There was significant variation in fidelity ratings and dropout rates among different counsellors.
			4					Acceptability and feasibility of task-sharing counselling The intervention was acceptable and feasible for depressed women. Female patients preferred female counsellors for the sessions. Monthly sessions in clinics with experienced Xhosa-speaking counsellors from the community were preferred. The intervention needed to include psychoeducation on: cognitive and behavioural effects of depression, coping with interpersonal issues, managing financial stress.
			6					Task-shared individual counselling for perinatal depression, delivered by trained and supervised lay community health workers using a structured manual, is a feasible and acceptable strategy to reduce the treatment gap for depressed pregnant women in low-resource settings like Khayelitsha. The thesis argues that applying the MRC process-evaluation framework (attention to context, implementation and mechanisms) is crucial for developing culturally relevant, scalable interventions; future policy and programmes should support scaling up such task-shared models, with investment in training, supervision, fidelity assessment and adaptation of session dose and content to women’s needs.
Musyimi et al. (2019)	Kenya	Qualitative design Interviews, FGDs Thematic analysis	88	Female, (range of age: 36–65 years)	Maternal depression	Psychological counselling, emotional support, psychosocial intervention	Informal health provider (traditional birth attendant) Community-based Potential primary care linkage through training and collaboration	Key themes from the qualitative data: Involving traditional birth attendants (TBAs) in perinatal mental healthcare. Leveraging rapport and counselling experience: Utilising TBAs’ existing rapport with the community and their counselling skills. Recognition and valuation of TBAs: Recognising and valuing the role of TBAs within the formal healthcare system. Training and collaboration: Providing training for TBAs and fostering collaboration between them and healthcare workers.
Myers et al. (2019)	South Africa	Mixed-methods study (qualitative component extracted) Depression scale, interviews Framework analysis	7	Female, age is not provided	Depression among patients with HIV/AIDS, diabetes, alcohol use	PST and motivational interviewing	Community health worker NGO-based Primary care-linked Delivering counselling within chronic disease services	Willingness to receive counselling: About 67% of eligible patients were willing to receive mental health counselling. Patients with depression were more interested in counselling compared to those with hazardous alcohol use. CHWs’ acceptance and requests: Both dedicated and designated community health workers (CHWs) found the counselling package highly acceptable. CHWs requested additional training and support to improve the effectiveness of the intervention’s implementation.
Ng’oma et al. (2024)	Malawi	Mixed-methods study (qualitative component extracted) Depression scales, interviews t-Test, thematic analysis	7	Female, (range of age 32–51 years, Mean of age: 36)	Perinatal depression	Psychoeducation, facilitating family support, behavioural activation and problem-solving	Peer volunteer Community-based Primary care-linked	Acceptability and feasibility of THPP (traditional home-based postnatal programme): Mothers and their families found the modified THPP to be both acceptable and practicable. Implementation challenges: Peer volunteers faced challenges due to inadequate incentives.
Pacichana-Quinayáz et al. (2016)	Colombia	Qualitative case study Interviews Narrative content analysis	5	Not provided	Depression and anxiety	Common Elements Treatment Approach (CETA), based on CBT, and the other was the Narrative Community-based Group Therapy based on narrative therapies	Lay psychosocial community worker Community-based Programme-based psychosocial intervention with specialist supervision	Challenges to implementation psychological intervention: The continuing conflict in the region hampers the implementation of the Common Elements Treatment Approach. Economic instability in the region further complicates the intervention’s delivery. The scarcity of mental health resources affects both the implementation and effectiveness of the intervention.
Pereira et al. (2011)	India	Qualitative design, Interviews, thematic analysis	17	Female, Mean: 27	Depression and anxiety	psychoeducation, antidepressants and interpersonal therapy	Lay health counsellor Primary care-based Programme-based collaborative care intervention with specialist support	Key factors for enhancing LHWs’ acceptability and integration: Adequate training for LHWs is essential. Building trust with patients and primary care teams is crucial. Allowing sufficient time for LHWs to establish relationships and deliver care. Demonstrating positive patient outcomes helps with acceptance. Regular supervision and support from psychiatrists is vital.
Rodríguez-Cuevas et al. (2021)	Mexico	Qualitative design A field report Not provided	4	Female, (range of age: 18–50)	Depression, anxiety and psychosomatic complaints	Coping with depression course, CBT techniques including psychoeducation on mental health, problem-solving, relaxation techniques, behavioural activation and family support mobilisation	Community health worker Community-based Primary care-linked NGO-supported home-based psychosocial intervention with clinic and specialist collaboration	The PM+ intervention positively impacted patients’ symptoms and improved LHW attitudes towards mental health and themselves. Challenges such as intimate partner violence, structural stress and COVID–19-related grief need to be addressed to better serve underserved areas, despite previous adaptations to the intervention.
Saleem et al. (2021)	Pakistan	Mixed-methods study (qualitative component extracted) Psychometric scales, interviews t-Test, thematic analysis	3	Female, age was not provided	Depression and anxiety	Psychoeducation and various methods of family therapy	Lay counsellor Community-based Primary care-linked Facilitating community multi-family groups through a primary care centre catchment area	Community treatment of common mental health disorders using multi-family groups led by non-specialists is feasible. The approach is well-received by participants. Improved quality of life. Reduction in depression and anxiety. Enhanced social outcomes.
Selohilwe et al. (2023)	South Africa	Qualitative design Interviews Framework analysis	19	Not provided	Depression among patients with coexisting hypertension. Patients with mild to moderate symptoms	Brief counselling intervention informed by adapted cognitive behavioural interventions, including problem-solving, cognitive restructuring and behavioural activation	Lay counsellor Facility-based Project-employed depression counselling service	Facilitators of the intervention: Supervision and support for counsellors. Person-focused counselling approach. Integration of counsellors within healthcare facilities. Barriers: Insufficient organisational support Lack of dedicated space for counselling High counsellor turnover Absence of a designated role for the intervention within the system Exclusion of mental health treatment, including counselling, from mental health indicators
Singla et al. (2020)	India	Mixed-methods study (qualitative component extracted) Psychological scales, interviews Paired t-Test, framework analysis	26	Female, (range of age 27–50 years, Mean: 37.85)	Perinatal depression	Behavioural activation, modifying unhealthy behaviours with healthy ones, active listening, collaboration with the family, guided discovery using pictures and stories, homework	Peer mother Community-based Home-based THPP delivery, trial-supported	Non-specialist providers can be trained to conduct peer supervision and reliably assess therapy quality using valid measures. Peer supervision involving experts was preferred for providing structured and reliable feedback.
Szajna et al. (2024)	India	Mixed-methods study (qualitative component extracted) EPDS scale and Interviews The pre-test/post-test analysis and thematic analysis	17	Female, age was not provided	Postpartum depression	Brief behavioural activation	Community health worker Community-based Primary healthcare-linked Home-based behavioural activation delivery	Feasibility and acceptability of behavioural activation (BA) High intervention uptake and retention Positive reception from participants Community health worker (CHW) delivery was well-received Improvements in depressive symptoms Enhanced daily activity and engagement Improvements in depressive symptoms Challenges and considerations Effective delivery depended on adequate CHW training and supervision. Balancing the intervention with other CHW responsibilities posed potential strain. Adapting content to the local context and aligning with postpartum norms helped improve relevance and uptake.
Wood et al. (2021)	India	Qualitative design Interviews Thematic analysis	32	Men (*n* = 8) and women (*n* = 24), Mean: 37-year-old	A range of mental illnesses including depression, anxiety, alcohol use disorders	Psychological counselling, behavioural activation	Lay mental health worker NGO-based, mainly community-based Home visits plus some clinic-based counselling	Barriers and facilitators of intervention delivery by LHWs Factors include personal traits, family support, daily work relationships and supervision. Compensation issues were highlighted as a key factor. Gender discrimination and stigma were identified as barriers.
Yator et al. (2022)	Kenya	Qualitative design Interviews Thematic analysis	8	Female, Mean of age: 37	Postpartum depression	Group interpersonal therapy (IPT-G)	Community health worker Primary healthcare-linked, community-linked facility-based IPT-G delivery	LHWs valued the intervention for improving their knowledge and skills. Patients reported better interpersonal relationships, reduced distress and less HIV-related stigma. Primary healthcare workers successfully integrated the intervention sessions into their routine clinic activities.

* Included as a single study due to complementary data across multiple sources.

Although the included LIPIs varied in terminology and format, most drew on a shared set of brief psychological strategies, especially problem-solving, behavioural activation and psychoeducation, often alongside CBT-informed techniques and, in some studies, motivational, interpersonal, family or psychosocial support elements (Table 3). LHWs were heterogeneous rather than a homogenous group (Table 3). Their organisational linkage ranged from government primary care and primary care-linked roles to NGO-, programme and project-based positions, often in hybrid arrangements combining community embeddedness with facility, NGO or project structures. Where reporting was limited, classification was based on authors’ descriptions of role, employment, setting and supervision. Although most studies focused on depression and/or anxiety, a small subset (8 of 28 studies) included broader common mental disorder or mixed-condition populations, these were retained only where findings relevant to depression and/or anxiety were extractable.

### Quality appraisal

Overall, the contributing studies demonstrated generally acceptable methodological quality, most clearly stated their aims, used appropriate qualitative methods and addressed ethical issues. However, theoretical underpinnings and researchers’ ontological or epistemological positions were rarely articulated. Reflexivity was limited, with little acknowledgement of researchers’ influence on data collection and interpretation. Although thematic or framework analyses were common, analytic procedures were often underdescribed, offering scant detail on theme development or divergent data. These limitations may reduce the depth and credibility of findings and underrepresent nuanced perspectives (see Supplementary material 4).

### Findings

Through the meta-ethnographic synthesis, this review translated and interpreted findings across studies to develop four overarching constructs that explain LHWs’ lived experiences delivering LIPIs for depression and anxiety in LMICs. Together, these constructs offer a conceptual understanding of the complexity of their work, how they negotiate recognition within the health system, their trajectories of growth in practice and the emotional demands embedded in community-based care.

#### Doing more than psychological therapy

Across studies, LHWs described work that extended far beyond structured low-intensity interventions. Symptom alleviation and core techniques such as psychoeducation, problem-solving, relaxation and healthy-thinking remained central (Atif, 2015; Abas et al., 2016; Pacichana-Quinayáz et al., 2016; Chibanda et al., 2017; Munodawafa et al., 2017), but LHWs also addressed the social and family circumstances shaping distress. They helped patients navigate poverty, secure food or finances, link to social services, resolve family conflict and respond to domestic violence (Pereira et al., 2011; Abas et al., 2016; Joag et al., 2020; Chau et al., 2021). In doing so, they routinely moved beyond formal intervention parameters into wider family and community issues to support safety and stability. For example, LHWs supporting women with depression also engaged husbands whose abusive behaviour contributed to their distress, not as part of the therapeutic protocol, but as a dynamic response to the surrounding context (Joag et al., 2020).

In one family, the husband used to beat his wife, when I came to know about them, I visited them. I counselled them. Showed them the films on quarrels, addiction. Now fights between them have reduced to a large extent. (Joag et al., 2020)

In areas with high mental health stigma, LHWs had to negotiate cultural and religious norms that discouraged engagement with psychological treatments such as counselling and behavioural activation. To overcome these barriers, they extended their work beyond manualised techniques, adapting therapeutic conversations, reframing symptoms and using culturally resonant practices to build trust and reduce resistance (Atif, 2015; Wood et al., 2021). Such adaptations illustrated both relational labour (managing relationships and cultural expectations) and emotional labour (modifying emotions to remain respectful, patient and reassuring when facing rejection).

When we visit [religious group] houses, they have temples and all and the patient and others are not ready to accept us and they said we don’t want your treatment and avoided us and they said our gods don’t like you coming here. But we convinced them and made a good relation with them and I even prayed at their home. (LHW, Female, 30 years). (Wood et al., 2021)

#### Embedded in the community, precariously held by the system

Many LHWs had lived in the same communities for years, sharing everyday spaces and life events with those they supported. This deep social embeddedness fostered trust and familiarity, so they were recognised not only in their formal roles but as longstanding community members (Atif, 2015; Chibanda et al., 2017; Chau et al., 2021; Wood et al., 2021; Chau et al., 2024; Bah et al., 2025). Their relationships extended beyond clinical contexts into markets, homes and religious gatherings, where shared histories and common experiences strengthened rapport (Atif, 2015; Chibanda et al., 2017; Saleem et al., 2021). This shared lived experience became a therapeutic resource, enabling patients to feel understood and safe. As one LHW noted, longstanding relationships often encouraged people to open up in ways they would not with outsiders.

So because we are known as Ambuya Utano (grandmother health worker) there is a certain level of trust we get from PLWH. We have lived here for more than 20 years so we are known because with most of them we see each other at the market, we have helped them in the past maybe during the cholera outbreak, or when we carried out a home visit to see if they had medication for the HIV illness. (Chibanda et al., 2017)

However, belonging did not always guarantee recognition and trust. For some LHWs, being deeply embedded in the community blurred the boundary between neighbour and professional. Familiarity could also generate tension, old conflicts resurfaced and fears of gossip or breaches of confidentiality made some patients reluctant to open up (Atif, 2015; Jacobs, 2020; Ahmed et al., 2024). LHWs noted that mothers in their village often dismissed their guidance, whereas women in neighbouring villages valued their advice more precisely because they were seen as outsiders (Atif, 2015).

A mother living in the same village as her PV (Peer Volunteer) might not pay much attention to what she is being told. Whereas if a PV is from a different village, she will think that she has come from far and acknowledge her better. Moreover if she is from same village, mother might or might not have good relationship with her. If the families have interpersonal conflicts, the PV might feel uncomfortable visiting them. (Atif, 2015)

While community recognition affirmed LHWs’ roles, support from formal health systems, such as training, psychoeducation materials, financial support and supervision, was equally vital in reinforcing their sense of legitimacy and supporting their development in the role (Atif, 2015; Pacichana-Quinayáz et al., 2016; Joag et al., 2020; Henrique et al., 2021; Kidia et al., 2024; Ng’oma et al., 2024; Szajna et al., 2024; Bah et al., 2025; Burgess et al., 2025). Supervision, in particular, created a space where LHWs could reflect on their practice, receive feedback and feel acknowledged in their efforts (Jacobs, 2020).

The supervisor will listen to the recording and then he comes every week for supervision. So at least he will tell me this, this and this we must improve there and there. We did well here, very good, this and this. So, the supervision helps me…. (Jacobs, 2020)

Despite their critical community role, many LHWs felt marginalised within formal health systems, treated as outsiders and excluded from decision-making (Musyimi et al., 2019; Myers et al., 2019; Jacobs et al., 2021; Wood et al., 2021; Selohilwe et al., 2023). This lack of recognition created practical barriers, including limited workspace, unclear role boundaries (Nyatsanza et al., 2016; Munodawafa et al., 2017; Jacobs, 2020; Henrique et al., 2021; Yator et al., 2022; Selohilwe et al., 2023; Kidia et al., 2024) and inadequate financial support (Abas et al., 2016; Atif et al., 2017; Jacobs et al., 2021; Wood et al., 2021; Ng’oma et al., 2024). LHWs also described the emotional toll of feeling undervalued, especially when their limited formal education led clinic staff to dismiss their contributions (Wood et al., 2021). These frustrations were compounded by unsafe work environments (conflict-affected areas with high criminality), long travel distances (Munodawafa et al., 2017; Wood et al., 2021) and minimal transport allowances, reinforcing a sense of being unsupported by the systems they served (Munodawafa et al., 2017; Wood et al., 2021; Kidia et al., 2024).

There are [staff] in the clinics who are more educated than us. It’s difficult for them to accept us and what we do. It’s difficult for them to accept the change that we bring and it’s hard for them to blend in with us. They will not coordinate with us and they speak offensively about us. It’s also hard for other people to accept us since we are not highly educated and non-professionals. And they prefer doctors and professionals. (Wood et al., 2021)

Those who trained us had said when we’ll get … uniforms, bicycles, $10 transport. And now we are in the field and transport allowance is $2 and I use $2.50 a day. (Kidia et al., 2024)

#### Growing in the role

While community recognition and embeddedness gave LHWs trust and legitimacy, it was not enough to sustain them through challenges. They had to translate recognition into effective therapeutic work, a process requiring confidence, adaptation and skill-building over time. Many struggled to balance protocol fidelity with patient needs, especially when manuals felt too rigid or when counselling demanded personalised responses (Munodawafa et al., 2017; Ng’oma et al., 2024). This learning process was rarely straightforward and often emotionally demanding. LHWs described frustration and a sense that their therapeutic skills were limited, particularly when patients did not complete CBT homework (Davies et al., 2022). For some, pressure to deliver the intervention correctly prompted more directive, even scolding, responses when homework was left undone (Davies et al., 2022). These reactions reflected a strong sense of responsibility for patients’ progress, broader healthcare norms that equate firmness with care and the strain of adhering to structured guidelines when patients could not engage as manuals assumed.

I was worried if I would be able to follow the manual and not add my own stuff. I was worried if I would be able to help the client with the problem they had, but at the end I was able to do so much so that others would tell me which sessions they enjoyed. (Munodawafa et al., 2017)

The last session you spoke about your likes and dislikes right? Where is your book? You do not write [your exercises]. I gave an example for you and you did not write. You see? I even said choose here all the way to this side. You see?. I gave an example and even made brackets for you… You did not write. Write! Write! At all. Are you getting old? … Do not think you have escaped because you still have the book [at home]. I want that book and this one full for Session 5. Do you understand? (Davies et al., 2022)

Across the studies, LHWs ranged from their early 20s to late 60s. Some, particularly older providers, described feeling less confident in adapting to new therapeutic methods, especially when comparing themselves to younger colleagues whom they perceived as more technologically skilled or quicker to learn (Chau et al., 2021). Similar anxieties arose during supervisory observations, where being watched made some LHWs feel scrutinised and unsure of their abilities (Munodawafa et al., 2017). Interactions with more educated patients could also undermine confidence, as some LHWs felt that differences in education or status weakened their authority and legitimacy as helpers (Atif, 2015). Their development into the role involved not only skill acquisition, but also negotiating insecurity and legitimacy.

I think young people like you [referring to the interviewer] are very talented. Old person like me cannot be talented like you. In fact, people said that they would like to see the old doctor, because they are full of experience, but I think there is no experience because the things they have learnt in the past is different from now. Now when I went to big hospital like (….), it is full of talented young doctors, I found that the youth is talented. (Chau et al., 2021)

Despite these early struggles, LHWs often described becoming more confident and capable through practice, supervision and repeated engagement with patients. Over time, some developed greater autonomy in delivering intervention and a stronger sense of themselves as credible helpers. (Atif, 2015; Atif et al., 2016; Pacichana-Quinayáz et al., 2016; Henrique et al., 2021; Jacobs et al., 2021; Rodríguez-Cuevas et al., 2021). Yet this growth was not only technical. For some, taking up the role reshaped how they understood and managed their emotions and worries, fostering greater confidence, agency and purpose, and inspiring aspirations to further education (Myers et al., 2019; Joag et al., 2020; Rodríguez-Cuevas et al., 2021; Wood et al., 2021). These accounts show that the LHW role was not fixed by treatment manuals but evolved as a dynamic process in which skills, confidence and legitimacy were gradually negotiated and strengthened through experience.

I live with my mom, and I used to fear making my own decisions before (becoming a CMHW). I would expect others to decide on my behalf, but not anymore. Now I make my own decisions and my mother and family respect them…I only studied until middle school, but now I am determined to major in psychology. I know this (job) made me fall for psychology, and I would not like to stop doing my work. You can help people not only by being a doctor or a nurse, but by being like me, some sort of a psychologist. (Rodríguez-Cuevas et al., 2021)

I used to worry a lot. When I was doing the intervention then I realised that there are things we worry about that are not important…so it seems like I am helping someone else, and I am also helping myself. (Myers et al., 2019)

#### The hidden cost of caring

LHWs’ accounts suggest that the effectiveness of care often depended on emotional closeness, but that this closeness came at a cost. Many used self-disclosure and shared experience to build trust and relatability with patients (Atif, 2015; Pacichana-Quinayáz et al., 2016; Chibanda et al., 2017; Ahmed et al., 2024). While this strengthened rapport, it also made the work emotionally demanding, especially when patients’ stories echoed their own. In these moments, LHWs were not only supporting others, but also managing their own emotional responses in order to remain calm, trustworthy and therapeutically present (Wood et al., 2021; Ahmed et al., 2024).

When they were telling their problems, I used to tell them that I had similar circumstances, but God has helped me to overcome it. Time never stays the same, I was stressed when my children were young, now my tension is gone. Mothers used to benefit from my experiences. (Atif, 2015)

“I can make an excuse to go drink water” (Oratile) and “I had to pretend like I wasn’t going through what [the client] is going through.” (Ahmed et al., 2024)

This emotional demand extended beyond the session itself. LHWs described care as consuming personal time, energy and attention, as intervention was layered onto existing family, community and clinical responsibilities (Henrique et al., 2021; Jacobs et al., 2021; Burgess et al., 2025). Care was often difficult to contain within formal work boundaries: patients rescheduled, arrived late or sought help outside agreed times, leaving LHWs waiting, readjusting plans and feeling obliged to remain available (Chibanda et al., 2017; Wood et al., 2021; Ahmed et al., 2024). Over time, this repeated extension of care could displace attention from LHWs’ own needs.

You know, sometimes a client will come and knock on my door (at home) hours after our session and say ‘Ambuya’ (grandma) I know which problem we have to focus on. (Chibanda et al., 2017)

if we decided that we should meet with the client around one, then the client decides to come around two… it’s a problem because I do have other commitments outside there. So, waiting an hour for a client is a problem. (Ahmed et al., 2024)

This emotional burden was further intensified by the contexts in which LHWs worked. They described stigma, suspicion and rejection during home visits as an additional emotional burden, while some women also faced direct safety risks, including sexual harassment from male patients (Wood et al., 2021). These accounts show that LHWs were often expected to keep caring despite hurt, fear, frustration and exposure to risk. In doing so, they absorbed not only emotional strain, but also the social and gendered costs of making psychological care possible in everyday community settings.

For me, the person I’m seeing is unwell, I don’t [care] whether it is men or ladies. But, things like that, he [the patient] pinched my lip and kissed. Those kinds of problems are there. (Wood et al., 2021)

Overall, we had moderate confidence in each of the four review findings, as assessed using GRADE-CERQual (Table 4).

**Table 4.** Summary of qualitative findings and GRADE-CERQual assessments Table 4. long description.

Summary of review findings	Contributing studies (n)	Methodological limitations*	Coherence*	Adequacy*	Relevance*	GRADE-CERQual assessment of confidence in the evidence*
**Doing more than psychological therapy** LHWs’ delivery of LIPIs routinely extended beyond symptom-focused techniques to include social, practical and family support.	10	Moderate	Minor	Minor	Minor	**Moderate confidence** Several contributing studies exhibited methodological limitations, including limited reflexivity and theoretical transparency, and some also introduced minor indirectness because they involved broader CMD or mixed-condition populations rather than depression/anxiety alone.
**Embedded in the community, precariously held by the system** LHWs were trusted and embedded in communities but remained marginal within formal systems, with fragile support and low recognition.	25	Moderate	Minor	Minor	Minor	**Moderate confidence** Methodological limitations across the 25 contributing studies, including limited reflexive reporting, underspecified theoretical orientation and incomplete description of sampling and credibility strategies.
**Growing in the role** LHWs described a trajectory from initial concern and role strain towards greater confidence, autonomy and a developing role identity.	10	Moderate	Minor	Minor	Minor	**Moderate confidence** Moderate methodological concerns, but rich, consistent accounts of role development across diverse programmes.
**The hidden cost of caring** LHWs’ work involved substantial emotional labour, with empathy, self-disclosure, ongoing care demands, stigma and safety risks contributing to cumulative emotional burden.	10	Moderate	Minor	Minor	Minor	**Moderate confidence** Moderate concerns about methodological limitations; minor concerns about relevance due to some contextual and population variation; good coherence and adequate data support the finding.

* Full justifications are provided in Supplementary Material 6.

## Discussion

Our synthesis revealed how LHWs experience and sustain the delivery of LIPIs in LMICs. Unlike existing reviews that frame task-shifting primarily as a pragmatic response to workforce shortages (Kakuma et al., 2011; Patel et al., 2011; van Ginneken et al., 2021), we found that LHWs’ delivery of psychological interventions is profoundly relational, adaptive and emotionally laborious. Most included studies were conducted in sub-Saharan Africa and South Asia, leaving relational and emotional dimensions of task-shifting underexamined in regions such as Latin America and Southeast Asia. Given that task-shifting is now central to the development and maintenance of mental health systems in Southeast Asia (Sharan et al., 2017), this represents a critical blind spot. This review contributes early cross-programme insights into LHWs’ relational and emotional labour in these underrepresented regions.

Our synthesis shows that LHWs in LMICs occupied dual identities as community members and mental health providers. They often struggled to reconcile being relatable neighbours and kin with the need to appear professional to gain legitimacy within health systems. Many shared the same poverty, domestic violence, stigma and social conflicts as those they supported. Many worked in settings where interdependence, mutual support and obligations to family and community were prominent, fostering belonging and social responsibility and shaping flexible, informal ways of delivering care. In this context, task-shifting emerged not simply as a technical transfer of tasks (Patel et al., 2011; WHO, 2019) but as a redistribution of emotional and relational labour, sustained through empathy, social connection and shared vulnerability. LHWs moved beyond symptom alleviation to address socio-economic drivers of distress by linking patients to social services, navigating bureaucracy, involving families and engaging with abusive partners. Yet they faced stigma, rejection and risks, including sexual harassment and emotionally demanding practices such as joining prayers or adapting patients’ worldviews (Atif et al., 2016; Nyatsanza et al., 2016; Wood et al., 2021). In this context, community embeddedness was therefore double-edged: the same shared social worlds that fostered trust and engagement could also expose LHWs to moral scrutiny and overlapping obligations shaped by local norms of kinship, deservingness and social responsibility (Chase, 2023). Their work depended not only on building relationships, but also on navigating local values about what counts as the right way to respond to distress.

Our synthesis suggests that organisational support arrangements shaped LHWs’ ability to sustain intervention delivery. This interpretation is also informed by the heterogeneity of the included studies: LHWs ranged from community volunteers and peers to lay counsellors and primary care- or NGO-linked workers, while the LIPIs varied in terminology and format but generally shared a core of brief structured strategies, particularly problem-solving, behavioural activation and psychoeducation, with some also including cognitive restructuring or other cognitive techniques. Overall, the findings suggest that variation in LHW role, organisational linkage and delivery setting shaped access to supervision, recognition, role clarity and support. However, these distinctions were not always easy to disentangle, because LHWs represented a heterogeneous workforce and primary studies did not consistently describe their training, role boundaries or relationship to the wider health system. This means that differences between LHW roles should be interpreted cautiously, as they may reflect broader features of community-based intervention delivery as well as specific worker characteristics. Support often appeared more structured where LHWs were embedded within formal or service-linked arrangements offering training, adapted resources, collaborative processes and supervision (Myers et al., 2019; Selohilwe et al., 2023). Yet these benefits depended not only on formal embedding, but also on genuine integration into service settings; recognition as part of the care team; protected time within the workflow to deliver sessions, attend supervision and manage associated tasks alongside other responsibilities; and organisational backing. LHWs linked to facilities, NGOs, government services or funded projects often received regular supervision, debriefing, fidelity monitoring, peer learning and specialist support, although some community-based and volunteer models also demonstrated robust supervisory structures (Joag et al., 2020; Singla et al., 2020; Ng’oma et al., 2024; Bah et al., 2025). This pattern was therefore not uniform. Integration alone did not shield LHWs from low pay, weak recognition, role overload or funding insecurity (Myers et al., 2019; Selohilwe et al., 2023). Rather, organisational embeddedness mattered insofar as it provided role clarity, sustained supervision, recognition, protected time and emotional support. Although critical ethnographic scholarship has cautioned that the formalisation of community mental health roles may create tensions between community-rooted care and system demands (Kottai and Ranganathan, 2020), the studies synthesised here more often showed LHWs maintaining strong community embeddedness and drawing on relational, adaptive and culturally resonant practices, even as they remained marginalised within formal systems.

However, structured supervision and organisational support did not, on their own, guarantee effective delivery. Even in settings where supervision and organisational supports were more structured, intervention delivery depended on more than technical skills or treatment fidelity alone. In many LMICs, where services are scarce and formal support structures remain weak, intervention delivery is often sustained through personal relationships, which become a key route through which care is enacted and support accessed (Chutiyami et al., 2025). People facing socio-economic vulnerability frequently rely on community cooperation to navigate external constraints and scarce resources (Wagner, 1995; Iacoviello and Lorenzi-Cioldi, 2019), and social expectations such as mutual support, attentiveness to others and solidarity shape how care is enacted, emphasising social obligation and moral connection over hierarchy (Mthembu et al., 2023). Within this context, LHWs operate simultaneously as community members and as extensions of the health system (Liu et al., 2011; Laurenzi et al., 2021). The effectiveness of psychological interventions therefore hinges on trust, relational resilience and LHWs’ ability to manage emotional demands, as they navigate blurred boundaries and balance friendship with professionalism to sustain interventions and maintain community acceptance (Atif, 2015; Pacichana-Quinayáz et al., 2016).

This relational engagement, however, often required LHWs to absorb patients’ distress and internalise emotional tension. This amplified the emotional labour needed to sustain therapeutic relationships. Emotional labour, managing one’s feelings to meet care expectations, often maintaining calm or empathy despite internal strain (Hochschild, 1983), emerged as the hidden mechanism enabling relational task-shifting. Unlike formal clinical settings where emotional display rules are explicit, LHWs’ emotion work was intuitive and embedded in shared community life, cultural norms of compassion and moral obligation. Unlike professionals with clinic boundaries that prevent overwork and allow recovery (Eriksson et al., 2018; Rapp et al., 2021; Clarke et al., 2024), they remained continuously accessible across community settings, leading patients to view them as always available sources of support. Similar concerns have been described in ethnographic work showing that the emotional demands of delivering psychological interventions can become morally and emotionally consequential for peer volunteers, particularly when care extends beyond formal sessions into memory, identity and continuing obligation, even after programme or trial structures bring support to an end (Leocata et al., 2021).

Because prevailing models of task-shifting emphasise technical skills, the relational and emotional dimensions of LHWs’ work often remain unrecognised. When overlooked, empathy, trust-building and community connectedness can be marginalised in training and supervision, which reduces care to procedural fidelity and symptom management, and leaving emotional labour invisible. This leaves LHWs underprepared for the emotional demands of delivering LIPIs and raises ethical concerns about unpaid or weakly recognised care work. Emotional and relational labour should therefore be recognised as integral to the LHW role and treated as part of formal service delivery rather than invisible goodwill. This recognition should be reflected in policy, training, supervision, workload planning, remuneration and referral systems. These capacities should not be treated as personal virtues alone, but as essential components of care that must be resourced, supervised, protected and fairly valued within health systems. This aligns with wider community health worker literature emphasising fair working conditions, opportunities for advocacy and stronger partnerships between community health worker, communities and policymakers to address structural inequities (Ahmed et al., 2022). It is also important from a gendered perspective, given that many LHWs in this review were women. Delegating clinical and emotional responsibilities to women without adequate pay risks reinforcing norms that position them as moral caregivers rather than legitimate wage earners, while undermining the sustainability of their work (Chase et al., 2021; Chase et al., 2022). At the same time, our findings showed that task-shifting could foster confidence, autonomy and aspiration.

Although mhGAP guidance emphasises observable skills such as detection, referral and adherence (Kohrt et al., 2018; WHO, 2019), our synthesis suggests that competence also develops through emotional labour, relational engagement and reflective practice. Recent competency-based initiatives such as WHO/UNICEF’s EQUIP represent an important step towards more structured training and supervision for non-specialists, including the assessment of foundational helping skills across intervention types (Kohrt et al., 2025). While competency frameworks can assess important helping skills, our findings suggest that effective LHW practice also depends on emotional, relational and organisational dimensions that are less easily captured in standardised measures. Supervision should therefore extend beyond treatment fidelity to support emotional insight and personal growth.

## Future research directions

As emotional labour is strongly associated with burnout, emotional exhaustion and withdrawal from caring roles (Clarke et al., 2024), failing to address it among LHWs in community-based care risks undermining workforce well-being, retention and the sustainability of task-sharing models in already fragile LMIC health systems. Future studies should examine how LHWs perform and regulate emotional labour across cultural contexts, and how these processes affect well-being and intervention quality by shaping resilience or vulnerability to burnout. Future intervention and implementation studies should also assess provider well-being alongside patient and service outcomes, using contextually appropriate measures of burnout, emotional burden, role strain and organisational support, and should consider longitudinal and qualitative approaches to better understand how these experiences develop over time. In addition, because emotional labour is demanding and embedded within the same communities where LHWs live, understanding the support networks they rely on is crucial. Social networks, connections among individuals or groups through interaction and exchange (Newman et al., 2002; Bae et al., 2015), provide emotional, informational and appraisal support that promote self-evaluation and health awareness (Ashida and Heaney, 2008; Crotty et al., 2015). Evidence shows that diverse networks enhance mental health by expanding access to emotional and practical resources (Fiori et al., 2007; Sultan et al., 2014). In this review, these networks include both formal and informal relationships such as supervisors, family and peers that help sustain resilience and well-being in community-based psychological care. Moreover, future research should also examine how different organisational arrangements shape LHWs’ supervision, emotional support and working conditions, and how these in turn affect well-being, retention and intervention sustainability (Myers et al., 2019; Selohilwe et al., 2023). This should include attention to how structural conditions such as remuneration, workload, role clarity and service integration contribute to unpaid or weakly recognised emotional labour, and which workforce development strategies are most effective in supporting and sustaining community providers.

## Strengths and limitations

Meta-ethnography enabled interpretation of LHWs’ lived experiences across studies and highlighted latent mechanisms, including emotional labour, relational trust and personal transformation, that more aggregative approaches may overlook. Using the GRADE-CERQual approach, we judged confidence in all four review findings to be moderate, due to moderate concerns about methodological limitations and only minor concerns about coherence, adequacy and relevance. Overall, the findings are likely to reasonably represent the phenomena, though future high-quality studies may refine them. However, this interpretive synthesis across diverse health systems reduces local specificity and limits reproducibility, as different reviewers may construct different conceptual models. Limited theoretical articulation and reflexivity in several included studies further constrained interpretive depth. In addition, although many LIPIs are transdiagnostic and may address broader distress rather than depression or anxiety alone, we retained a depression/anxiety focus to provide a clearer and more coherent analytical scope for the review. However, a small number of included studies involved broader common mental disorder or mixed-condition populations, and although extraction focused on depression/anxiety-relevant findings where possible, the influence of other conditions on some reported experiences cannot be fully excluded. A further limitation concerns the heterogeneity of LHW roles and the variable reporting of their training, responsibilities and organisational linkage across studies. This made neat classification difficult and limited the specificity with which some differences could be attributed to particular types of LHWs rather than to broader features of community-based intervention delivery. Most primary studies sampled LHWs who remained in post, with little insight into those who left, so the synthesis may overrepresent workers who felt able to continue and underrepresent those who disengaged or burned out. Finally, although broader literature exists on community-based mental health work in other LMICs, including countries such as Nepal, Uganda, Nigeria and Bangladesh, many such studies fell outside this review’s scope because they examined neither defined LIPIs targeting depression and/or anxiety nor LHWs’ qualitative delivery experiences. The concentration of studies in India, Zimbabwe and South Africa therefore allowed identification of cross-contextual patterns, but the underrepresentation of regions such as Latin America and Southeast Asia restricts transferability, and findings should be applied cautiously in these settings.

## Conclusion

Across the included programmes, largely from Africa and Asia, with more limited representation from Latin America, this review shows that LHW-delivered LIPIs depend on intensive relational and emotional labour, not only the transfer of technical tasks. Yet this work remains insufficiently recognised in policy and programme design, leaving LHWs vulnerable to burnout, role strain and attrition, and ultimately undermining workforce retention and programme sustainability. If ministries of health and global agencies want task-sharing models to endure, emotional and relational labour must be recognised as core components of care and reflected in training, supervision, remuneration, role clarity, safeguarding, organisational support and opportunities for development and recognition, rather than being left to individual goodwill.

## Supporting information

10.1017/gmh.2026.10271.sm001 Kurniawan et al. supplementary material Kurniawan et al. supplementary material

## Data Availability

All data supporting the findings of this review are contained within the article, its supplementary materials and the published studies it cites.

## Supplementary material

The supplementary material for this article can be found at http://doi.org/10.1017/gmh.2026.10271.

## Long descriptions

### Table 1. Long description

The table is structured into four columns representing different levels of qualitative synthesis.

* First-order construct: Contains direct quotes from participants in various studies (Atif 2015, Pacichana-Quinayáz et al. 2016, Ahmed et al. 2024, Wood et al. 2021, Munodawafa et al. 2017, and Jacobs et al. 2021). These quotes detail personal experiences of motherhood, shared trauma, emotional suppression during counseling, and the burden of heavy workloads.

* Second-order construct: Provides thematic interpretations of the quotes. Key themes include Experience of being a mother, Characteristics and L P C W performance, Counselor Psychological Well-being, Workload, Scheduling Procedures, Implementation, Perceptions of Clinical Supervision, and Social context regarding gender.

* Translation: Groups the second-order constructs into four broader narrative categories:

1. Caring through shared pain: Focuses on how shared experiences build trust but increase vulnerability.

2. When caring consumes the self: Describes the suppression of personal emotions and self-neglect to maintain professional presence.

3. When the work never ends: Details the strain of integrating psychological interventions into existing community and domestic duties.

4. Caring under gendered risk: Highlights challenges from gender norms, family resistance, and harassment.

* Third-order construct: The final synthesis titled The hidden cost of caring. It summarizes the four translation categories into a cohesive finding that while social proximity builds therapeutic connection, it leaves L H W s emotionally vulnerable and prone to self-neglect due to continuous, risky, and gendered work demands.

Navigate back to Table 1..

### Figure 1. Long description

The flowchart is divided into three vertical sections: Identification, Screening, and Included.

1. Identification Phase:

- Top Left: Identification of studies via databases. Studies from databases/registers n = 4987. Sources include C I N A H L n = 956, Psyc I N F O n = 850, Embase n = 811, A S S I A n = 688, Dissertations and Theses Global n = 484, M E D L I N E n = 454, C E N T R A L n = 444, and Google Scholar n = 300.

- Top Right: Identification of studies via other methods. References from other sources n = 1052. Sources include Reviews n = 508, Grey Literature n = 42, and Citation searching n = 511.

- An arrow from both boxes leads to a central point where 1079 references are removed: Duplicates identified manually n = 15 and Duplicates identified by Covidence n = 1064.

2. Screening Phase:

- Titles and abstracts screened n = 4960. An arrow points right to Studies excluded n = 4857.

- Full text studies assessed for eligibility n = 103. An arrow points right to Studies excluded n = 68. Reasons include: Wrong population of L H W n = 17, Wrong population of patient n = 10, Wrong phenomenon of interest n = 4, Wrong evaluation n = 18, Wrong study design n = 9, Wrong setting n = 5, and Not available in full text n = 5.

- Studies initially included after full text screening n = 35.

- Included studies returned to full-text screening for reappraisal n = 4. An arrow points right to Studies excluded after eligibility reassessment and author contact: Wrong population of patient n = 2.

3. Included Phase:

- At the bottom, the final box indicates Studies included in the synthesis n = 33.

Navigate back to Figure 1..

### Table 2. Long description

The table is organized into five columns: Region, Countries, Income classification, Total of studies, and percentage of total studies.

* Sub-Saharan Africa (53.6 percent of total):

- South Africa: Upper-middle income, 6 studies, 21.4 percent.

- Zimbabwe: Lower-middle income, 3 studies, 10.7 percent.

- Kenya: Lower-middle income, 2 studies, 7.1 percent.

- Ethiopia: Low income, 1 study, 3.6 percent.

- Botswana: Upper-middle income, 1 study, 3.6 percent.

- Malawi: Low income, 1 study, 3.6 percent.

- Sierra Leone: Low income, 1 study, 3.6 percent.

* South Asia (32.1 percent of total):

- India: Lower-middle income, 7 studies, 25 percent.

- Pakistan: Lower-middle income, 2 studies, 7.1 percent.

* Latin America (10.7 percent of total):

- Brazil: Upper-middle income, 1 study, 3.6 percent.

- Colombia: Upper-middle income, 1 study, 3.6 percent.

- Mexico: Upper-middle income, 1 study, 3.6 percent.

* Southeast Asia (3.6 percent of total):

- Vietnam: Lower-middle income, 1 study, 3.6 percent.

Navigate back to Table 2..

### Table 3. Long description

A detailed table with nine columns: Author s, Countries, Design, data collection and analysis, Number of participants L H Ws, Gender and age range of L H Ws, Intervention target, Interventions, Background of L H W and organisational linkage, and Key findings.

Key entries include:

* Abas et al. 2016: Zimbabwe, mixed-methods, 40 to 60 female participants, targeting depression with problem-solving therapy P S T. Findings show positive reception and cost-effectiveness.

* Ahmed et al. 2024: Botswana, qualitative design, 8 mixed-gender participants, targeting depression and anxiety with P S T and behavioural activation. Barriers included client reticence and financial resources.

* Atif et al. 2015 to 2017: India and Pakistan, qualitative design, 8 female participants, targeting perinatal depression using C B T principles. Acceptability was enhanced by local presence and shared life experiences.

* Bah et al. 2025: Sierra Leone, mixed-methods, 10 female participants, targeting perinatal and postpartum depression with P S T. Reported high recruitment and reduction in psychological distress.

* Burgess et al. 2025: Ethiopia, qualitative, 4 female participants, targeting postpartum depression and anxiety with simplified C B T and family planning. Group sessions reduced isolation.

* Chau et al. 2021 and 2024: Vietnam, qualitative, 47 mixed-gender participants, targeting depression. Findings highlight that strong relationships foster openness but low education levels can reduce trust.

* Chibanda et al. 2016 and 2017: Zimbabwe, qualitative, 7 female participants, targeting depression and common mental disorders in people living with H I V or A I D S using P S T. Benefits included reduced stigma and improved access to care.

* Davis et al. 2022: South Africa, qualitative, 6 female participants, targeting perinatal depression. Socio-economic difficulties were a challenge, but participants reported improved mood.

* Henrique et al. 2021: Brazil, qualitative, 11 female participants, targeting depression. The P R O A C T I V E intervention was well-received but required better training.

* Jacobs et al. 2020 and 2021: South Africa, qualitative, 18 mixed-gender participants, targeting depression or alcohol use. Barriers included low pay and lack of space.

* Joag et al. 2020: India, mixed-methods, 16 participants, targeting common mental disorders. Community-led interventions successfully facilitated access to social benefits.

* Kidia et al. 2024: Zimbabwe, qualitative, 12 participants, targeting common mental disorders with P S T and income generation. Sustainability was a concern.

* Munodawafa et al. 2017 and 2018: South Africa, qualitative, multiple cohorts of 4 to 6 female participants, targeting perinatal depression. Success was linked to structured manuals and local language use.

* Musyimi et al. 2019: Kenya, qualitative, 88 female participants, targeting maternal depression by involving traditional birth attendants.

* Myers et al. 2019: South Africa, mixed-methods, 7 female participants, targeting depression in patients with H I V or A I D S and diabetes.

* Ng’oma et al. 2024: Malawi, mixed-methods, 7 female participants, targeting perinatal depression. Peer volunteers faced challenges with inadequate incentives.

* Pacichana-Quinayáz et al. 2016: Colombia, qualitative case study, 5 participants, targeting depression and anxiety. Conflict and economic instability were major barriers.

* Pereira et al. 2011: India, qualitative, 17 female participants, targeting depression and anxiety. Integration required building trust with primary care teams.

* Rodríguez-Cuevas et al. 2021: Mexico, qualitative, 4 female participants, targeting depression and anxiety. P M plus intervention improved L H W attitudes.

* Saleem et al. 2021: Pakistan, mixed-methods, 3 female participants, targeting depression and anxiety using family therapy.

* Selohilwe et al. 2023: South Africa, qualitative, 19 participants, targeting depression with hypertension. Barriers included high counsellor turnover.

* Singla et al. 2020: India, mixed-methods, 26 female participants, targeting perinatal depression. Peer supervision was preferred for quality assessment.

* Szajna et al. 2024: India, mixed-methods, 17 female participants, targeting postpartum depression with brief behavioural activation.

* Wood et al. 2021: India, qualitative, 32 mixed-gender participants, targeting a range of mental illnesses. Compensation and stigma were key barriers.

* Yator et al. 2022: Kenya, qualitative, 8 female participants, targeting postpartum depression with group interpersonal therapy I P T G.

Navigate back to Table 3..

### Table 4. Long description

A table with seven columns: Summary of review findings, Contributing studies (n), Methodological limitations, Coherence, Adequacy, Relevance, and G R A D E dash C E R Q u a l assessment of confidence in the evidence.

Row 1: Doing more than psychological therapy. L H W s’ delivery of L I P I s extended to social, practical, and family support. 10 studies. Limitations: Moderate. Coherence, Adequacy, and Relevance: Minor. Assessment: Moderate confidence due to limited reflexivity and theoretical transparency.

Row 2: Embedded in the community, precariously held by the system. L H W s were trusted in communities but marginal in formal systems. 25 studies. Limitations: Moderate. Coherence, Adequacy, and Relevance: Minor. Assessment: Moderate confidence due to underspecified theoretical orientation and incomplete sampling descriptions.

Row 3: Growing in the role. L H W s moved from role strain to confidence and autonomy. 10 studies. Limitations: Moderate. Coherence, Adequacy, and Relevance: Minor. Assessment: Moderate confidence based on rich, consistent accounts of role development.

Row 4: The hidden cost of caring. L H W s’ work involved emotional labor, stigma, and safety risks. 10 studies. Limitations: Moderate. Coherence, Adequacy, and Relevance: Minor. Assessment: Moderate confidence despite minor concerns about relevance due to population variation.

Navigate back to Table 4..
