# Supplementary material for: Delivering low-intensity psychological interventions in low- and middle-income countries: A systematic review and meta-ethnographic synthesis of lay health workers’ experiences
Source: Glob Ment Health (Camb). 2026 Jul 7;13:e157. doi: 10.1017/gmh.2026.10271 (PMC13433339; doi:10.1017/gmh.2026.10271)
Supplement: Supplementary file 1 [file S2054425126102714sup001.zip › Supplementary material 1 - Eligibility criteria.docx]

**Supplementary material 1**

**Eligibility Criteria**

| SPIDER Components | Inclusion criteria | Exclusion criteria |
| --- | --- | --- |
| Sample of population | - LHWs operating in LMICs as per World Bank classification for 2022 and 2023 - Studies where LHWs are the primary interventionists - Mixed populations (LHWs with professional health workers) included if LHW delivery is clearly distinguishable and extractable | - Studies conducted in High Income Countries - Studies where LHWs’ roles are not separable from those of other health providers, or where LHWs are not the primary interventionists |
|  | - Adult patients aged 18 and older with depression and anxiety - Adolescent patients (ages 13-17) will be included only if the primary focus is on adult populations or if adolescents are included alongside adults - Patients with comorbid physical illnesses (e.g., diabetes, cardiovascular diseases, HIV/AIDS) alongside depression and anxiety. - Mixed patient samples (severe mental illness plus depression/anxiety) included only when depression and/or anxiety data were clearly identifiable and extractable. | - Studies including patients diagnosed with severe mental illnesses (e.g., schizophrenia, bipolar disorder, psychoses) without comorbid depression or anxiety - Studies focusing solely on adolescent patients (ages 13-17) without the inclusion of adult patients |
| Phenomenon of Interest | - the delivery process of LIPIs by LHWs for patients with depression and anxiety - LIPIs were defined as brief, structured psychological interventions delivered by trained non-specialists with supervision, requiring less therapist input than conventional therapy and often drawing on CBT principles, such as behavioural, cognitive, problem-solving, or psychoeducational approaches. Interventions could be delivered through guided self-help, digital formats, group sessions, or brief face-to-face contact. | - Studies that do not explore the delivery process of LIPIs by LHWs for patients with depression and anxiety - Interventions that are not described as a defined psychological intervention |
| Design | - Empirical studies with primary qualitative data (e.g. individual interviews, focus group discussions) specifically related to LHWs, with data disaggregated from other health professionals where relevant. - Mixed-methods studies that include a qualitative component (e.g. nested process evaluations, interview or focus-group sub-studies) on LHWs’ experiences. | - Studies where qualitative data cannot be clearly attributed to LHWs (e.g. only combined with data from other cadres or professionals and not separable). - Articles without primary qualitative interview or focus-group data on LHWs’ experiences (e.g. quantitative-only studies, reviews, commentaries, editorials, opinion pieces). |
| Evaluation | - LHWs’ experiences in delivering LIPIs for patients with depression and anxiety | - Studies that do not explore LHWs’ experiences in delivering LIPIs |
| Research types | - Qualitative studies - Mixed-methods studies with qualitative components - No restrictions on publication dates or languages. | - Quantitative studies without involving qualitative component on LHWs’ experiences - Reviews, editorials, commentaries or opinion pieces (although reference lists of reviews were screened for eligible primary studies). - Studies for which the full text could not be obtained, even after attempts via institutional access and by contacting authors. |
