# Supplementary material for: Delivering low-intensity psychological interventions in low- and middle-income countries: A systematic review and meta-ethnographic synthesis of lay health workers’ experiences
Source: Glob Ment Health (Camb). 2026 Jul 7;13:e157. doi: 10.1017/gmh.2026.10271 (PMC13433339; doi:10.1017/gmh.2026.10271)
Supplement: Supplementary file 1 [file S2054425126102714sup001.zip › Supplementary material 2 - Search strategy.docx]

## **Supplementary material 2**

## **Search strategy**

## June 17, 2025

**CINAHL**

| **Sample of Population** | **Result** |
| --- | --- |
| barefoot doctor* | 26 |
| "Consejero" | 21 |
| (MH "Caregivers") | 44,086 |
| (Community N3 (worker* or advisor* or advocate* or educator* or visitor* or attendant* or aide or aides or support* or person* or helper* or carer* or caregiver* or consultant* or advisor* or counselor* or counsellor* or assistant* or staff or facilitator* or nurse* or practitioner* or personnel* or volunteer*)) | 40,270 |
| (MH "community health workers") | 4,567 |
| "health auxiliary" | 4 |
| "health cadres" or "cadres" or "Kader" | 1,469 |
| (MH "health educators") | 1,972 |
| (MH "home health aides") | 1,541 |
| (MH "Health Personnel, Unlicensed") or mental health personnel | 9,351 |
| indigenous* N2 volunteer* | 8 |
| (informal N3 (caregiver* or "care giver" or "care givers" or carer*)) | 4,891 |
| lady* N3 worker* | 74 |
| (lay* N3 (worker* or advisor* or advocate* or educator* or visitor* or attendant* or aide or aides or support* or person* or facilitator* or helper* or carer* or caregiver* or "care giver" or "care givers" or consultant* or advisor* or counselor* or counsellor* or assistant* or staff or facilitator* or practitioner* or personnel* or volunteer*)) | 2,399 |
| (non-medical N3 (worker* or visitor* or attendant* or aide or aides or support* or person* or helper* or carer* or caregiver* or "care giver" or "care givers" or consultant* or advisor* or counselor* or counsellor* or assistant* or personnel* or staff)) | 372 |
| (nonprofessional* N3 (worker* or visitor* or attendant* or aide or aides or support* or person* or helper* or carer* or caregiver* or "care giver" or "care givers" or consultant* or advisor* or counselor* or counsellor* or assistant* or staff)) | 129 |
| (Non-specialist* N3 (worker* or advisor* or advocate* or educator* or visitor* or attendant* or aide or aides or support* or person* or helper* or carer* or caregiver* or "care giver" or "care givers" or consultant* or advisor* or counselor* or counsellor* or assistant* or staff or facilitator* or practitioner* or personnel* or volunteer*)) | 161 |
| (Paraprofessional* N3 (worker* or advisor* or advocate* or educator* or visitor* or attendant* or aide or aides or support* or person* or helper* or carer* or caregiver* or "care giver" or "care givers" or consultant* or counselor* or counsellor* or assistant* or staff or facilitator* or practitioner* or personnel* or volunteer*)) | 265 |
| (Peer N3 (worker* or advisor* or advocate* or educator* or visitor* or attendant* or aide or aides or support* or person* or helper* or carer* or caregiver* or "care giver" or "care givers" or consultant* or advisor* or counselor* or counsellor* or assistant* or staff or facilitator* or practitioner* or personnel* or volunteer*)) | 11,117 |
| (Rural N3 (worker* or advisor* or advocate* or educator* or visitor* or attendant* or aide or aides or support* or person* or helper* or carer* or caregiver* or "care giver" or "care givers" or consultant* or advisor* or counselor* or counsellor* or assistant* or staff or facilitator* or practitioner* or personnel* or volunteer*)) | 4,848 |
| (MH "rural health personnel") | 782 |
| (untrained N3 (worker* or visitor* or attendant* or aide or aides or support* or person* or helper* or carer* or caregiver* or care giver* or "care givers" or consultant* or advisor* or counselor* or counsellor* or assistant* or staff or nurse* or doctor* or personnel* or physician* or therapist*)) | 358 |
| (unlicensed N3 (worker* or visitor* or attendant* or aide or aides or support* or person* or helper* or carer* or caregiver* or "care giver" or "care givers" or consultant* or advisor* or counselor* or counsellor* or assistant* or staff or nurse* or doctor* or personnel* or physician* or therapist*)) | 4,154 |
| Village N3 (worker* or advisor* or advocate* or educator* or visitor* or attendant* or aide or aides or support* or person* or helper* or carer* or caregiver* or "care giver" or "care givers" or consultant* or advisor* or counselor* or counsellor* or assistant* or staff or facilitator* or practitioner* or personnel* or volunteer*)) | 535 |
| (voluntary N3 (worker* or visitor* or attendant* or aide or aides or support* or person* or helper* or carer* or caregiver* or "care giver" or "care givers" or consultant* or advisor* or counselor* or counsellor* or assistant* or staff)) | 659 |
| (MH "Volunteer Workers") | 15,147 |
| Total | 149,206 |
| **Phenomenon of Interest (LIPIs)** | **Result** |
| (MH "health care delivery") | 66,428 |
| intervention N3 delivery | 3,955 |
| treatment N3 offer | 3,757 |
| Treatment N3 delivery | 3,834 |
| "low-intensity interventio*" | 158 |
| (MH "Behavior Therapy") | 13,776 |
| "behavior activation" | 18 |
| brief* N3 therap* | 1,656 |
| (MH "Psychotherapy, Brief") | 1,442 |
| cognitive N3 therap* | 29,556 |
| (MH "Cognitive Restructuring") | 104 |
| (MH "cognitive therapy") | 21,669 |
| (MH "Emotional Regulation") or emotion regulation | 6,718 |
| (MH "internet-based intervention") or web-based interventions' or 'e-health' or 'internet-based intervention or digital intervention | 27,263 |
| (MH "Mindfulness") OR (MH "Mind Body Techniques") OR (MH "Mental Healing") | 12,759 |
| (MH "Stress Management") or stress reduction or relaxation training | 14,532 |
| (MH "Meditation") | 5,943 |
| Relaxation therapy or relaxation theraph* | 2,647 |
| solution N2 therap* | 3,646 |
| mind body therapy or mind body exercis* or mind-body exercis* or Mind-Body Therapie* | 2,675 |
| peer N2 counsel* | 1,654 |
| Peer counselling or "support group*" or "group counselling" | 16,246 |
| problem N3 therap* | 2,759 |
| (MH "psychoeducation") | 4,170 |
| psychosocial* N3 intervention* | 6,748 |
| psychological* N3 intervention* | 6,841 |
| psychosocial* N3 counsel* | 1,464 |
| psychosocial N2 care* | 33,209 |
| (self help or self-help devices or self-help group* or support group*) | 38,661 |
| (MH "Support Groups") | 11,422 |
| "task-shifting" | 689 |
| ("thinking healthy" or "thinking healthy program*") | 22 |
| Total | 346,421 |
| **Phenomenon of Interest (Mental health issues)** | **Result** |
| (mental N3 (problem* or issue* or disorder*)) | 103,217 |
| (MH "Psychological Distress") OR (MH "Symptom Distress") | 8,524 |
| (MH "depression") or "mixed mania and depression" or minor depression or antenatal depression or major depression or long-term depression or agitated depression or late life depression or postoperative depression or recurrent brief depression or depression or "mixed depression and dementia" or bipolar depression or "mixed anxiety and depression" or chronic depression or reactive depression | 218,854 |
| (MH "Emotional Lability") or emotional problems or emotional disturbances or affective disorders or affective symptoms | 24,049 |
| (MH "Affective Disorders") or mood disorder or emotional disorder or emotional distress | 26,041 |
| perinatal depression | 1,349 |
| (MH "Depression, Postpartum") | 7,025 |
| (MH "Anxiety") OR (MH "Social Anxiety Disorders") OR (MH "Anxiety Disorders") OR (MH "Separation Anxiety") OR (MH "Generalized Anxiety Disorder") OR (MH "Anticipatory Anxiety") OR (MH "Dental Anxiety") or castration anxiety or anxiety neurosis or anticipatory anxiety or travel anxiety or death anxiety or illness anxiety disorder or speech anxiety or "mixed anxiety and depression" or performance anxiety | 78,240 |
| (MH "Stress Disorders, Post-Traumatic") OR (MH "Stress, Occupational") OR (MH "Stress, Psychological") OR acute stress or chronic stress or mental stress or parental stress or psychological stress or psychological distress | 128,718 |
| (MH "Affective Symptoms") OR "psychological symptoms" OR "psychological problems" | 11,123 |
| "psychiatric symptoms" or "psychiatric disorders" or "distress syndrome" | 78,506 |
| "common mental disorders" | 1,432 |
| (MH "Cognition Disorders") OR "mild cognitive impairment" or cognitive impairment or cognitive problems | 60,447 |
| "negative emotions" or "negative feelings" or "rumination" | 7,583 |
| problem behavior or behavior disorder | 28,462 |
| (MH "Panic Disorder") or "panic attack" | 2,594 |
| Total | 786,164 |
| **Design, Research type, Evaluation** | **Result** |
| (MH "Qualitative Studies") OR "qualitative research" OR "qualitative analysis" | 149,913 |
| (mixed N2 (method or study or design)) | 33,247 |
| (MH "interviews") | 170,000 |
| (MH "Focus Groups") or "focus group discussion or "FGD" | 51,626 |
| (MH "Phenomenological Research") or "phenomenological studies" | 19,847 |
| (MH "Case Studies") or "case reports" | 42,679 |
| "experienc*" | 599,261 |
| "perspectiv*" | 176,478 |
| "perceptio*" | 195,493 |
| "opinio*" | 52,075 |
| "vie*" | 184,565 |
| Total | 1,675,184 |
| **LMICs** | **Result** |
| (MH “low and middle income countries”) OR (MH "Developing Countries") | 23,545 |
| (MH "Africa") OR (MH "Africa South of the Sahara") OR (MH "Africa, Western") OR (MH "Africa, Central") OR (MH "Africa, Southern") OR (MH "Africa, Eastern") OR (MH "Africa, Northern") OR (MH "South Africa") | 38,163 |
| (MH "Asia") OR (MH "Asia, Southern") OR (MH "Asia, Western") OR (MH "Asia, Southeastern") OR (MH "Asia, Central") | 8,954 |
| (MH "Latin America") OR (MH "Central America") | 4,250 |
| South* Asia or Africa* or "South America*" or "Latin America*" or "Central America*" or Carribean or "Sub-Saharan Africa*" or (Afghanistan or Albania or Algeria or "American Samoa" or Angola or Argentina or "Argentine Republic” or Algeria or Armenia" or Azerbaijan or Africa or “South America” of Bangladesh or Belarus or Byelarus or Belorussia or Belize or Benin or Bhutan or Bolivia or Bosnia or Herzegovina or Hercegovina or "Bosnia and Herzegovina" or Botswana or Brazil or Bulgaria or "Burkina Faso" or Burundi or "Cabo Verde" or "Cape Verde" or Cambodia or Cameroon or "Central African Republic" or Chad or China or Colombia or Comoros or Congo or "Costa Rica" or "Cote d'Ivoire" or "Ivory Coast" or Cuba or Djibouti or Dominica or "Dominican Republic" or Ecuador or Egypt or "El Salvador" or "Equatorial Guinea" or Eritrea or Eswatini or Ethiopia or Fiji or Gabon or Gambia or Georgia or Ghana or Grenada or Grenadines or Guatemala or Guinea or Guinea-Bissau or Guyana or Haiti or Honduras or India or Indonesia or Iran or Iraq or Jamaica or Jordan or Kazakhstan or Kenya or Kiribati or "North Korea" or "DPR Korea" or Kosovo or Kyrgyz or Kyrgyzstan or "Kyrgyz Republic" or "Lao PDR" or Laos or Lebanon or Lesotho or Liberia or Libya or Madagascar or Malawi or Malaysia or Malaya or Maldives or Mali or "Marshall Islands" or Mauritania or Mauritius or Mexico or Micronesia or Moldova or Mongolia or Montenegro or Morocco or Mozambique or Myanmar or Burma or Namibia or Nepal or Niue or Nicaragua or Niger or Nigeria or Macedonia or "North Macedonia" or Pakistan or Palau or Panama or "Papua New Guinea" or Paraguay or Peru or Philippines or "Russian Federation" or Russia or Rwanda or Ruanda or Samoa or Saint Helena or Saint Lucia or "Sao Tome and Principe" or Senegal or Serbia or "Sierra Leone" or "Solomon Islands" or Somalia or "South Africa" or "South Sudan" or Suriname or "Sri Lanka" or "St. Lucia" or "St. Vincent and Grenadines" or Sudan or Suriname or Syria or Tajikistan or Tanzania or Thailand or Timor-Leste or Togo or Tokelau or Tonga or Tunisia or Turkiye or Turkey or Turkmenistan or Tuvalu or Uganda or Ukraine or Uzbekistan or Vanuatu or Vietnam or Venezuela or "Wallis and Futuna" or "West Bank and Gaza" or Palestine or Gaza or "West Bank" or Yemen or Zambia or Zimbabwe | 667240 |
| **Total** | 957 |

**EMBASE**

| **Sample of Population** | **Result** |
| --- | --- |
| barefoot doctor*.mp | 155 |
| consejero.mp | 7 |
| caregiver/ | 117,839 |
| (Community adj3 (worker* or advisor* or advocate* or educator* or visitor* or attendant* or aide or aides or support* or person* or helper* or carer* or caregiver* or consultant* or advisor* or counselor* or counsellor* or assistant* or staff or facilitator* or nurse* or practitioner* or personnel* or volunteer*)) | 62,459 |
| health auxiliary/ | 9,906 |
| (health cadre* or mental health cadre* or cadre* or Kader).mp | 6,317 |
| health educator/ | 2403 |
| "home health aides".mp | 291 |
| ("mental health personnel" or "unlicensed health personnel").mp | 119 |
| indigenous* adj2 volunteer* | 15 |
| (informal adj3 (caregiver* or "care giver" or "care givers" or carer*)) | 8,008 |
| lady* adj3 worker* | 205 |
| (lay adj3 (worker* or advisor* or advocate* or educator* or visitor* or attendant* or aide or aides or support* or person* or facilitator* or helper* or carer* or caregiver* or "care giver" or "care givers" or consultant* or advisor* or counselor* or counsellor* or assistant* or staff or facilitator* or practitioner* or personnel* or volunteer*)) | 4,369 |
| lay health worker/ | 199 |
| (non medical adj3 (worker* or visitor* or attendant* or aide or aides or support* or person* or helper* or carer* or caregiver* or "care giver" or "care givers" or consultant* or advisor* or counselor* or counsellor* or assistant* or personnel* or staff)) | 1,355 |
| (nonprofessional* adj3 (worker* or visitor* or attendant* or aide or aides or support* or person* or helper* or carer* or caregiver* or "care giver" or "care givers" or consultant* or advisor* or counselor* or counsellor* or assistant* or staff)) | 249 |
| (Non-specialist* adj3 (worker* or advisor* or advocate* or educator* or visitor* or attendant* or aide or aides or support* or person* or helper* or carer* or caregiver* or "care giver" or "care givers" or consultant* or advisor* or counselor* or counsellor* or assistant* or staff or facilitator* or practitioner* or personnel* or volunteer*)) | 399 |
| (Paraprofessional* adj3 (worker* or advisor* or advocate* or educator* or visitor* or attendant* or aide or aides or support* or person* or helper* or carer* or caregiver* or "care giver" or "care givers" or consultant* or counselor* or counsellor* or assistant* or staff or facilitator* or practitioner* or personnel* or volunteer*)) | 384 |
| (Peer adj3 (worker* or advisor* or advocate* or educator* or visitor* or attendant* or aide or aides or support* or person* or helper* or carer* or caregiver* or "care giver" or "care givers" or consultant* or advisor* or counselor* or counsellor* or assistant* or staff or facilitator* or practitioner* or personnel* or volunteer*)) | 16,749 |
| (Rural adj3 (worker* or advisor* or advocate* or educator* or visitor* or attendant* or aide or aides or support* or person* or helper* or carer* or caregiver* or "care giver" or "care givers" or consultant* or advisor* or counselor* or counsellor* or assistant* or staff or facilitator* or practitioner* or personnel* or volunteer*)) | 6,556 |
| (untrained adj3 (worker* or visitor* or attendant* or aide or aides or support* or person* or helper* or carer* or caregiver* or care giver* or "care givers" or consultant* or advisor* or counselor* or counsellor* or assistant* or staff or nurse* or doctor* or personnel* or physician* or therapist*)) | 1,049 |
| (unlicensed adj3 (worker* or visitor* or attendant* or aide or aides or support* or person* or helper* or carer* or caregiver* or "care giver" or "care givers" or consultant* or advisor* or counselor* or counsellor* or assistant* or staff or nurse* or doctor* or personnel* or physician* or therapist*)) | 494 |
| (Village adj3 (worker* or advisor* or advocate* or educator* or visitor* or attendant* or aide or aides or support* or person* or helper* or carer* or caregiver* or "care giver" or "care givers" or consultant* or advisor* or counselor* or counsellor* or assistant* or staff or facilitator* or practitioner* or personnel* or volunteer*)) | 1,367 |
| (voluntary adj3 (worker* or visitor* or attendant* or aide or aides or support* or person* or helper* or carer* or caregiver* or "care giver" or "care givers" or consultant* or advisor* or counselor* or counsellor* or assistant* or staff)) | 6361 |
| Voluntary Worker/ | 5,139 |
| Total | 252,394 |
| **Phenomenon of Interest (LIPIs)** | **Result** |
| health care delivery/ | 211,762 |
| intervention adj3 delivery.mp | 4,720 |
| treatment adj3 offer.mp | 7,782 |
| treatment delivery.mp | 7,063 |
| low-intensity adj3 intervention.mp | 527 |
| behavior therapy/ | 46,508 |
| behavio?r* activation.mp | 3,251 |
| brief adj3 therapy.mp | 2,269 |
| Psychotherapy, Brief/ | 442 |
| cognitive behavioral therapy/ | 26,083 |
| cognitive restructuring/ | 364 |
| cognitive therapy/ | 44,669 |
| (cognitive adj3 therapy*).mp | 80,800 |
| emotion regulation/ | 6,688 |
| web-based intervention/ or internet based intervention* or digital intervention*.mp | 5,622 |
| mindfulness-based stress reduction/ or mindfulness/ or mindfulness-based cognitive therapy/ or mindfulness meditation/ | 16,976 |
| stress reduction.mp or stress management/ or relaxation training/ | 24,769 |
| meditation.mp | 13,783 |
| Relaxation therapy.mp | 1021 |
| solution-focused therapy/ or solution-focused brief therapy/ | 129 |
| (mind body therapy or mind body exercise* or mind-body exercise*).mp. or Mind-Body Therapies/ | 50,166 |
| (peer adj3 counselling).mp | 285 |
| (Peer counselling or "support group*" or "group counselling").mp | 23,387 |
| problem solving therapy/ | 179 |
| psychoeducation/ | 12,280 |
| psychosocial intervention/ | 2,312 |
| (psychological adj3 intervention).mp | 7,569 |
| (psychosocial adj3 counselling.mp) | 423 |
| psychosocial care/ | 23,764 |
| self help/ or self help grou* or self help*.mp | 22,714 |
| "task-shifting" or (task* adj3 (shar* or shift*)).mp | 6,161 |
| ("thinking healthy" or "thinking healthy program*").mp | 49 |
| Total | 654,517 |
| **Phenomenon of Interest (Mental health issues)** | **Result** |
| (mental adj2 (proble* or issu* or disorde*)).mp. or mental disease/ | 367,176 |
| "mixed mania and depression"/ or minor depression/ or antenatal depression/ or major depression/ or long-term depression/ or depression.mp or agitated depression/ or late life depression/ or postoperative depression/ or recurrent brief depression/ or depression/ or "mixed depression and dementia"/ or bipolar depression/ or "mixed anxiety and depression"/ or chronic depression/ or reactive depression/ | 874,303 |
| emotional proble*.mp or emotional disturbance or affective disorder or affective symptoms.mp | 25,269 |
| mood disorder/ or emotional disorder/ or emotional distress.mp | 85,221 |
| perinatal depression/ | 1,423 |
| postnatal depression/ | 7,682 |
| social anxiety/ or dental anxiety/ or separation anxiety/ or anxiety disorder/ or castration anxiety/ or anxiety/ or anxiety neurosis/ or generalized anxiety disorder/ or anticipatory anxiety/ or anxiety.mp or travel anxiety or death anxiety or illness anxiety disorder or speech anxiety or "mixed anxiety and depression"/ or performance anxiety/ or social anxiety.mp | 512,886 |
| acute stress/ or chronic stress/ or mental stress/ or parental stress/ or psychological stress or psychological distress or occupational stress or stress disorder.mp | 237,502 |
| psychological proble* or psychological symptoms.mp | 23,595 |
| distress syndrome/ or psychiatric symptoms or psychiatric disorder.mp | 103,714 |
| "common mental disorders".mp | 3,739 |
| exp Cognitive Impairment/ or exp Mild Cognitive Impairment/ | 626,714 |
| Negative Emotions/ or negative feelin* or rumination.mp | 11,453 |
| problem behavior/ or behavior disorder/ | 70916 |
| Panic disorders or panic attack.mp | 2,524 |
| Total | 2,954,117 |
| **Design, Research type, Evaluation** | **Result** |
| qualitative analysis/ or qualitative research/ or qualitative studi*.mp | 209,585 |
| (mixed adj2 (metho* or desig* or studi*)).mp | 61,417 |
| interview/ or intervie*.mp | 653,580 |
| focus group discussio* or focus groups or FGD.mp | 66,658 |
| phenomenology/ or phenomenological studi*.mp | 13,833 |
| case study/ or case report/ | 3,034,574 |
| experience/ or experienc*.mp or personal experience/ | 2,078,422 |
| perspectiv*.mp | 559,499 |
| perception/ | 165,199 |
| opinio*.mp | 190,275 |
| vie*.mp | 778,910 |
| Total | 7,811,952 |
| **LMICs** | **Result** |
| low-middle income country.mp. or developing country/ | 102,499 |
| africa.mp. or "Africa south of the Sahara"/ or South Africa/ or Africa/ or North Africa/ or Central Africa/ | 226,225 |
| asia.mp. or central Asia/ or western Asia/ or Asia/ or Southeast Asia/ or South Asia/ or northern Asia/ | 170,747 |
| latin america.mp. or "South and Central America"/ | 35,420 |
| (South* Asia or Africa* or "South America*" or "Latin America*" or "Central America*" or Carribean or "Sub-Saharan Africa*" or (Afghanistan or Albania or Algeria or "American Samoa" or Angola or Argentina or "Argentine Republic” or Algeria or Armenia" or Azerbaijan or Africa or “South America” of Bangladesh or Belarus or Byelarus or Belorussia or Belize or Benin or Bhutan or Bolivia or Bosnia or Herzegovina or Hercegovina or "Bosnia and Herzegovina" or Botswana or Brazil or Bulgaria or "Burkina Faso" or Burundi or "Cabo Verde" or "Cape Verde" or Cambodia or Cameroon or "Central African Republic" or Chad or China or Colombia or Comoros or Congo or "Costa Rica" or "Cote d'Ivoire" or "Ivory Coast" or Cuba or Djibouti or Dominica or "Dominican Republic" or Ecuador or Egypt or "El Salvador" or "Equatorial Guinea" or Eritrea or Eswatini or Ethiopia or Fiji or Gabon or Gambia or Georgia or Ghana or Grenada or Grenadines or Guatemala or Guinea or Guinea-Bissau or Guyana or Haiti or Honduras or India or Indonesia or Iran or Iraq or Jamaica or Jordan or Kazakhstan or Kenya or Kiribati or "North Korea" or "DPR Korea" or Kosovo or Kyrgyz or Kyrgyzstan or "Kyrgyz Republic" or "Lao PDR" or Laos or Lebanon or Lesotho or Liberia or Libya or Madagascar or Malawi or Malaysia or Malaya or Maldives or Mali or "Marshall Islands" or Mauritania or Mauritius or Mexico or Micronesia or Moldova or Mongolia or Montenegro or Morocco or Mozambique or Myanmar or Burma or Namibia or Nepal or Niue or Nicaragua or Niger or Nigeria or Macedonia or "North Macedonia" or Pakistan or Palau or Panama or "Papua New Guinea" or Paraguay or Peru or Philippines or "Russian Federation" or Russia or Rwanda or Ruanda or Samoa or Saint Helena or Saint Lucia or "Sao Tome and Principe" or Senegal or Serbia or "Sierra Leone" or "Solomon Islands" or Somalia or "South Africa" or "South Sudan" or Suriname or "Sri Lanka" or "St. Lucia" or "St. Vincent and Grenadines" or Sudan or Suriname or Syria or Tajikistan or Tanzania or Thailand or Timor-Leste or Togo or Tokelau or Tonga or Tunisia or Turkiye or Turkey or Turkmenistan or Tuvalu or Uganda or Ukraine or Uzbekistan or Vanuatu or Vietnam or Venezuela or "Wallis and Futuna" or "West Bank and Gaza" or Palestine or Gaza or "West Bank" or Yemen or Zambia or Zimbabwe)).mp. | 2,914,661 |
| Total LMICs | 3,449,552 |
| **Total:** | **938** |

**PsyINFO**

| **Sample of Population** | **Result** |
| --- | --- |
| barefoot doctor*.mp | 8 |
| consejero.mp | 16 |
| caregivers/ | 38,098 |
| (Community adj3 (worker* or advisor* or advocate* or educator* or visitor* or attendant* or aide or aides or support* or person* or helper* or carer* or caregiver* or consultant* or advisor* or counselor* or counsellor* or assistant* or staff or facilitator* or nurse* or practitioner* or personnel* or volunteer*)) | 26,130 |
| community health workers.mp | 1,704 |
| health auxiliary.mp | 1 |
| (health cadre* or mental health cadre* or cadre* or Kader).mp | 3,385 |
| exp Intervention/ or exp Community Health/ or exp Public Health/ or exp Health Promotion/ or exp Community Services/ | 245,875 |
| home care personnel/ | 569 |
| ("mental health personnel" or "unlicensed health personnel").mp | 13424 |
| (indigenous* adj2 volunteer*).mp | 10 |
| (informal adj3 (caregiver* or "care giver" or "care givers" or carer*)) | 3705 |
| lady* adj3 worker* | 20 |
| (lay adj3 (worker* or advisor* or advocate* or educator* or visitor* or attendant* or aide or aides or support* or person* or facilitator* or helper* or carer* or caregiver* or "care giver" or "care givers" or consultant* or advisor* or counselor* or counsellor* or assistant* or staff or facilitator* or practitioner* or personnel* or volunteer*)) | 2485 |
| (non medical adj3 (worker* or visitor* or attendant* or aide or aides or support* or person* or helper* or carer* or caregiver* or "care giver" or "care givers" or consultant* or advisor* or counselor* or counsellor* or assistant* or personnel* or staff)) | 192 |
| (nonprofessional* adj3 (worker* or visitor* or attendant* or aide or aides or support* or person* or helper* or carer* or caregiver* or "care giver" or "care givers" or consultant* or advisor* or counselor* or counsellor* or assistant* or staff)) | 574 |
| (Non-specialist* adj3 (worker* or advisor* or advocate* or educator* or visitor* or attendant* or aide or aides or support* or person* or helper* or carer* or caregiver* or "care giver" or "care givers" or consultant* or advisor* or counselor* or counsellor* or assistant* or staff or facilitator* or practitioner* or personnel* or volunteer*)) | 138 |
| (Paraprofessional* adj3 (worker* or advisor* or advocate* or educator* or visitor* or attendant* or aide or aides or support* or person* or helper* or carer* or caregiver* or "care giver" or "care givers" or consultant* or counselor* or counsellor* or assistant* or staff or facilitator* or practitioner* or personnel* or volunteer*)) | 2,163 |
| exp Paraprofessional Personnel/ | 2,598 |
| (Peer adj3 (worker* or advisor* or advocate* or educator* or visitor* or attendant* or aide or aides or support* or person* or helper* or carer* or caregiver* or "care giver" or "care givers" or consultant* or advisor* or counselor* or counsellor* or assistant* or staff or facilitator* or practitioner* or personnel* or volunteer*)) | 11,922 |
| (Rural adj3 (worker* or advisor* or advocate* or educator* or visitor* or attendant* or aide or aides or support* or person* or helper* or carer* or caregiver* or "care giver" or "care givers" or consultant* or advisor* or counselor* or counsellor* or assistant* or staff or facilitator* or practitioner* or personnel* or volunteer*)) | 2,504 |
| (untrained adj3 (worker* or visitor* or attendant* or aide or aides or support* or person* or helper* or carer* or caregiver* or care giver* or "care givers" or consultant* or advisor* or counselor* or counsellor* or assistant* or staff or nurse* or doctor* or personnel* or physician* or therapist*)) | 313 |
| (unlicensed adj3 (worker* or visitor* or attendant* or aide or aides or support* or person* or helper* or carer* or caregiver* or "care giver" or "care givers" or consultant* or advisor* or counselor* or counsellor* or assistant* or staff or nurse* or doctor* or personnel* or physician* or therapist*)) | 115 |
| (Village adj3 (worker* or advisor* or advocate* or educator* or visitor* or attendant* or aide or aides or support* or person* or helper* or carer* or caregiver* or "care giver" or "care givers" or consultant* or advisor* or counselor* or counsellor* or assistant* or staff or facilitator* or practitioner* or personnel* or volunteer*)) | 209 |
| (voluntary adj3 (worker* or visitor* or attendant* or aide or aides or support* or person* or helper* or carer* or caregiver* or "care giver" or "care givers" or consultant* or advisor* or counselor* or counsellor* or assistant* or staff)) | 659 |
| volunteers/ or "assistance (social behavior)"/ or community involvement/ | 17,616 |
| Total | 17,616 |
| **Phenomenon of Interest (LIPIs)** | **Result** |
| health care delivery/ | 22,863 |
| intervention adj3 delivery | 1,628 |
| treatment adj3 offer | 1,218 |
| treatment delivery.mp | 960 |
| (low-intensity adj3 intervention).mp | 247 |
| exp Behavior Therapy/ | 112,235 |
| behavioral activation system/ or behavio?r* activation.mp | 3,441 |
| brief adj3 therapy.mp | 3,953 |
| exp Brief Psychotherapy/ | 8,820 |
| cognitive behavior therapy/ | 25,020 |
| exp Cognitive Restructuring/ | 1,239 |
| exp Cognitive Therapy/ | 14,122 |
| (cognitive adj3 therapy*).mp | 55,728 |
| exp Emotional Control/ or exp Emotional Regulation/ | 20,922 |
| digital intervention/ or web-based intervention* or internet based intervention*.mp | 3,225 |
| exp Mindfulness-Based Interventions/ or exp Mindfulness/ | 15,607 |
| exp Stress Management/ or relaxation training or stress reductions.mp | 14,382 |
| meditation.mp | 10,731 |
| Relaxation therapy.mp | 5,433 |
| solution focused therapy/ or solution focused brief therapy.mp | 1231 |
| (mind-body therapy or mind body exercise* or mind-body exercise*).mp. or exp Mind Body Therapy/ | 9987 |
| peer counseling/ | 1,256 |
| (Peer counselling or "support group*" or "group counselling").mp | 11,176 |
| solution focused therapy/ or problem solving/ | 31,751 |
| psychoeducation/ | 5,643 |
| (psychosocial adj3 intervention).mp | 3,529 |
| (psychological adj3 intervention).mp | 5,035 |
| (psychosocial adj3 counselling).mp | 104 |
| psychosocial care.mp | 1,407 |
| psychotherapeutic processes/ | 38,753 |
| self help groups.mp | 4,365 |
| self help*.mp | 14,411 |
| task-shifting.mp | 477 |
| (task* adj3 (shar* or shift*)).mp | 4,068 |
| ("thinking healthy" or "thinking healthy program*").mp | 34 |
| Total | 455,001 |
| **Phenomenon of Interest (Mental health issues)** | **Result** |
| mental disorders/ or (mental adj3 (problem* or issue* or disorder*)) | 228,407 |
| exp Late Life Depression/ or exp Major Depression/ or exp Recurrent Depression/ or exp "Depression (Emotion)"/ or exp "Long-term Depression (Neuronal)"/ or depression.mp. or exp Reactive Depression or depressive disorder.mp | 406,625 |
| exp Emotional Disturbances/ or exp Affective Disorders/ | 189,538 |
| mood disorder.mp. or exp Affective Disorders/ or exp Emotional Disturbances/ or emotional distress.mp | 199,590 |
| perinatal depression.mp | 1,124 |
| exp Postpartum Depression/ | 6,245 |
| exp Illness Anxiety Disorder/ or exp Computer Anxiety/ or exp Generalized Anxiety Disorder/ or exp Speech Anxiety/ or exp Separation Anxiety/ or exp Separation Anxiety Disorder/ or exp Travel Anxiety/ or exp Death Anxiety/ or exp Anxiety Sensitivity/ or exp Health Anxiety/ or exp Performance Anxiety/ or exp Social Anxiety/ or exp Castration Anxiety/ or anxiety/ or anxiety disorders/ or anxiety problems.mp | 112,873 |
| exp Stress/ or distress/ or acute stress.mp. or chronic stress.mp. or parental stress.mp. or stress disorders.mp or psychological stress.mp. or psychological distress.mp. or parental stress.mp. or occupational stress.mp | 278,928 |
| psychological problem* or psychological symptom*.mp | 17,944 |
| exp Psychiatric Symptoms/ or psychiatric disorders.mp | 56,775 |
| common mental disorder*.mp | 2,723 |
| exp Cognitive Impairment/ or cognitive disorders.mp | 47,333 |
| negative emotions/ or negative feelings or exp "Rumination (Eating)"/ or exp "Rumination (Cognitive Process)"/ or rumination.mp | 20,841 |
| exp Behavior Problems/ or problem behavior.mp | 36,696 |
| exp Panic Attack/ or exp Panic Disorder/ | 8,563 |
| Total | 1,614,205 |
|  |  |
| **Design, Research type, Evaluation** | **Result** |
| qualitative methods/ | 11,406 |
| exp qualitative measures/ | 116 |
| (mixed adj2 (methods or designs or studies)).mp | 30,333 |
| exp Interviews/ or intervie*.mp | 498,719 |
| (focus group discussion* or FGD).mp. or Focus Groups/ | 6,494 |
| Case Reports/ or case studies.mp | 34,158 |
| exp Phenomenology/ or phenomenological studies.mp | 18,810 |
| exp "Experiences (Events)"/ or experience*.mp | 904,027 |
| perspective*.mp | 346,740 |
| perceptio*.mp | 611,237 |
| opinio*.mp | 65,846 |
| vie*.mp | 367,892 |
| Total | 2,895,778 |
|  |  |
| **LMICs** | **Result** |
| exp Developing Countries/ or "low and middle income countries".mp | 10,652 |
| Africa.mp | 30,315 |
| Asia.mp | 11,907 |
| exp "Latinos/Latinas"/ or latin america.mp | 39,283 |
| (South* Asia or Africa* or "South America*" or "Latin America*" or "Central America*" or Carribean or "Sub-Saharan Africa*" or (Afghanistan or Albania or Algeria or "American Samoa" or Angola or Argentina or "Argentine Republic” or Algeria or Armenia" or Azerbaijan or Africa or “South America” of Bangladesh or Belarus or Byelarus or Belorussia or Belize or Benin or Bhutan or Bolivia or Bosnia or Herzegovina or Hercegovina or "Bosnia and Herzegovina" or Botswana or Brazil or Bulgaria or "Burkina Faso" or Burundi or "Cabo Verde" or "Cape Verde" or Cambodia or Cameroon or "Central African Republic" or Chad or China or Colombia or Comoros or Congo or "Costa Rica" or "Cote d'Ivoire" or "Ivory Coast" or Cuba or Djibouti or Dominica or "Dominican Republic" or Ecuador or Egypt or "El Salvador" or "Equatorial Guinea" or Eritrea or Eswatini or Ethiopia or Fiji or Gabon or Gambia or Georgia or Ghana or Grenada or Grenadines or Guatemala or Guinea or Guinea-Bissau or Guyana or Haiti or Honduras or India or Indonesia or Iran or Iraq or Jamaica or Jordan or Kazakhstan or Kenya or Kiribati or "North Korea" or "DPR Korea" or Kosovo or Kyrgyz or Kyrgyzstan or "Kyrgyz Republic" or "Lao PDR" or Laos or Lebanon or Lesotho or Liberia or Libya or Madagascar or Malawi or Malaysia or Malaya or Maldives or Mali or "Marshall Islands" or Mauritania or Mauritius or Mexico or Micronesia or Moldova or Mongolia or Montenegro or Morocco or Mozambique or Myanmar or Burma or Namibia or Nepal or Niue or Nicaragua or Niger or Nigeria or Macedonia or "North Macedonia" or Pakistan or Palau or Panama or "Papua New Guinea" or Paraguay or Peru or Philippines or "Russian Federation" or Russia or Rwanda or Ruanda or Samoa or Saint Helena or Saint Lucia or "Sao Tome and Principe" or Senegal or Serbia or "Sierra Leone" or "Solomon Islands" or Somalia or "South Africa" or "South Sudan" or Suriname or "Sri Lanka" or "St. Lucia" or "St. Vincent and Grenadines" or Sudan or Suriname or Syria or Tajikistan or Tanzania or Thailand or Timor-Leste or Togo or Tokelau or Tonga or Tunisia or Turkiye or Turkey or Turkmenistan or Tuvalu or Uganda or Ukraine or Uzbekistan or Vanuatu or Vietnam or Venezuela or "Wallis and Futuna" or "West Bank and Gaza" or Palestine or Gaza or "West Bank" or Yemen or Zambia or Zimbabwe)).mp. |  |
| Total LMICs | 421,690 |
| **Total:** | 849 |

**MEDLINE**

| **Sample of Population** | **Result** |
| --- | --- |
| barefoot doctor*.mp | 148 |
| consejero.mp | 7 |
| caregivers/ | 51,697 |
| (Community adj3 (worker* or advisor* or advocate* or educator* or visitor* or attendant* or aide or aides or support* or person* or helper* or carer* or caregiver* or consultant* or advisor* or counselor* or counsellor* or assistant* or staff or facilitator* or nurse* or practitioner* or personnel* or volunteer*)) | 44,458 |
| Community Health Workers/ | 6,718 |
| "health auxiliary".mp | 14 |
| (health cadre* or mental health cadre* or cadre* or Kader).mp" | 6927 |
| Health Educators/ | 497 |
| "home health aides".mp | 948 |
| ("mental health personnel" or "unlicensed health personnel").mp | 116 |
| (indigenous* adj2 volunteer*).mp | 19 |
| (informal adj3 (caregiver* or "care giver" or "care givers" or carer*)) | 6,404 |
| lady* adj3 worker* | 165 |
| (lay adj3 (worker* or advisor* or advocate* or educator* or visitor* or attendant* or aide or aides or support* or person* or facilitator* or helper* or carer* or caregiver* or "care giver" or "care givers" or consultant* or advisor* or counselor* or counsellor* or assistant* or staff or facilitator* or practitioner* or personnel* or volunteer*)) | 3,469 |
| lay health workers.mp | 412 |
| (non medical adj3 (worker* or visitor* or attendant* or aide or aides or support* or person* or helper* or carer* or caregiver* or "care giver" or "care givers" or consultant* or advisor* or counselor* or counsellor* or assistant* or personnel* or staff)) | 853 |
| (nonprofessional* adj3 (worker* or visitor* or attendant* or aide or aides or support* or person* or helper* or carer* or caregiver* or "care giver" or "care givers" or consultant* or advisor* or counselor* or counsellor* or assistant* or staff)) | 241 |
| (Non-specialist* adj3 (worker* or advisor* or advocate* or educator* or visitor* or attendant* or aide or aides or support* or person* or helper* or carer* or caregiver* or "care giver" or "care givers" or consultant* or advisor* or counselor* or counsellor* or assistant* or staff or facilitator* or practitioner* or personnel* or volunteer*)) | 313 |
| (Paraprofessional* adj3 (worker* or advisor* or advocate* or educator* or visitor* or attendant* or aide or aides or support* or person* or helper* or carer* or caregiver* or "care giver" or "care givers" or consultant* or counselor* or counsellor* or assistant* or staff or facilitator* or practitioner* or personnel* or volunteer*)) | 328 |
| (Peer adj3 (worker* or advisor* or advocate* or educator* or visitor* or attendant* or aide or aides or support* or person* or helper* or carer* or caregiver* or "care giver" or "care givers" or consultant* or advisor* or counselor* or counsellor* or assistant* or staff or facilitator* or practitioner* or personnel* or volunteer*)) | 12,659 |
| (Rural adj3 (worker* or advisor* or advocate* or educator* or visitor* or attendant* or aide or aides or support* or person* or helper* or carer* or caregiver* or "care giver" or "care givers" or consultant* or advisor* or counselor* or counsellor* or assistant* or staff or facilitator* or practitioner* or personnel* or volunteer*)) | 5,726 |
| (untrained adj3 (worker* or visitor* or attendant* or aide or aides or support* or person* or helper* or carer* or caregiver* or care giver* or "care givers" or consultant* or advisor* or counselor* or counsellor* or assistant* or staff or nurse* or doctor* or personnel* or physician* or therapist*)) | 870 |
| (unlicensed adj3 (worker* or visitor* or attendant* or aide or aides or support* or person* or helper* or carer* or caregiver* or "care giver" or "care givers" or consultant* or advisor* or counselor* or counsellor* or assistant* or staff or nurse* or doctor* or personnel* or physician* or therapist*)) | 450 |
| (Village adj3 (worker* or advisor* or advocate* or educator* or visitor* or attendant* or aide or aides or support* or person* or helper* or carer* or caregiver* or "care giver" or "care givers" or consultant* or advisor* or counselor* or counsellor* or assistant* or staff or facilitator* or practitioner* or personnel* or volunteer*)) | 1,213 |
| (voluntary adj3 (worker* or visitor* or attendant* or aide or aides or support* or person* or helper* or carer* or caregiver* or "care giver" or "care givers" or consultant* or advisor* or counselor* or counsellor* or assistant* or staff)) | 1,156 |
| volunteers/ or "health volunteers".mp | 11,794 |
| (untrained adj3 (worker* or visitor* or attendant* or aide or aides or support* or person* or helper* or carer* or caregiver* or care giver* or "care givers" or consultant* or advisor* or counselor* or counsellor* or assistant* or staff or nurse* or doctor* or personnel* or physician* or therapist*)) | 870 |
| Total | 157,602 |
| **Phenomenon of Interest (LIPIs)** | **Result** |
| Delivery of Health Care/ | 120,525 |
| intervention adj3 delivery | 3,718 |
| treatment adj3 offer.mp | 5,249 |
| treatment delivery.mp | 3,492 |
| (low-intensity adj3 intervention).mp | 439 |
| Behavior Therapy/ | 30,650 |
| behavio?r* activation.mp | 2573 |
| brief adj3 therapy.mp | 1,599 |
| brief psychotherapy/ | 3,692 |
| cognitive behavioral therapy/ | 30,818 |
| Cognitive Restructuring/ | 29 |
| cognitive therapy.mp | 3,873 |
| (cognitive adj3 therapy*).mp | 44,208 |
| emotion regulation.mp | 12,646 |
| internet-based intervention/ or web-based intervention*.mp | 2,668 |
| Mindfulness/ | 6,592 |
| stress reduction or stress management.mp | 11,206 |
| Meditation/ | 3,883 |
| Relaxation Therapy/ | 6,580 |
| (solution adj3 therapy).mp | 732 |
| (mind body therapy or mind body exercise* or mind-body exercise*).mp. or Mind-Body Therapies/ | 1,678 |
| (peer adj2 counseling).mp | 404 |
| (Peer counselling or "support group*" or "group counselling").mp | 9,688 |
| (problem solving adj3 therapy).mp | 623 |
| psychoeducation.mp | 4,740 |
| psychosocial intervention/ | 1054 |
| (psychological adj3 intervention).mp | 5,233 |
| (psychosocial adj3 counselling).mp | 238 |
| psychosocial care.mp | 2,462 |
| psychotherapeutic processes/ | 1,321 |
| Self-Help Groups/ | 9,646 |
| self help*.mp | 21,867 |
| task-shifting.mp | 1,571 |
| (task* adj3 (shar* or shift*)).mp | 5,262 |
| ("thinking healthy" or "thinking healthy program*").mp | 44 |
| Total | 361,003 |
| **Phenomenon of Interest (Mental health issues)** | **Result** |
| Mental Disorders/ or (mental adj3 (problem* or issue* or disorder*)).mp | 257,663 |
| Depressive Disorder/ or Depression/ or mixed mania depression or minor depression or antenatal depression or major depression or long-term depression or depression.mp. or agitated depression or late life depression or postoperative depression or recurrent brief depression or brief depression or mixed depression-dementia or bipolar depression or chronic depression or reactive depression.mp | 530,104 |
| emotional disturbances.mp. or Affective Symptoms/ | 14,934 |
| mood disorders/ or emotional disorders or affective disorders or negative emotions or emotional distress.mp | 48,485 |
| Depression, Postpartum/ | 7,688 |
| perinatal depression.mp | 1,688 |
| anxiety/ or anxiety disorders/ or Performance Anxiety/ or Anxiety/ or Anxiety, Castration/ or Anxiety Disorders/ or Anxiety, Separation/ or generalized anxiety disorder or social anxiety or mixed anxiety depression or separation anxiety or death anxiety or health anxiety or performance anxiety or travel anxiety or speech anxiety.mp | 154,280 |
| Stress Disorders, Post-Traumatic/ or Stress, Psychological/ or Psychological Distress/ or stress or chronic stress or acute stress or psychological stress psychological distress or occupational stress or parental stress.mp | 1,198,161 |
| psychological problems or psychological symptoms.mp | 17,123 |
| psychiatric symptoms or psychiatirc disorders.mp | 13,598 |
| common mental disorder*.mp | 3,921 |
| Cognitive Dysfunction/ or cognitive disorders or cognitive impairment or mild cognitive impairment.mp | 103,939 |
| negative feelings or rumination.mp | 8359 |
| Problem Behavior/ | 4,095 |
| Panic attack or Panic disorder*.mp | 12,664 |
| Total | 2,376,702 |
|  |  |
| **Design, Research type, Evaluation** | **Result** |
| qualitative research/ | 84,961 |
| (mixed adj2 (design or method or research or study)).mp | 34,091 |
| Interview/ or interview*.mp | 505,305 |
| (focus group discussion* or FGD).mp. or Focus Groups/ | 46,691 |
| phenomenological studies or phenomenology.mp | 12,187 |
| Case Reports/ or case studies.mp | 2,419,516 |
| experience*.mp | 1,404,873 |
| perspective*.mp | 473,868 |
| perception*.mp | 540,785 |
| opinion*.mp. or Attitude/ | 202,514 |
| view*.mp | 569,435 |
| Total | 6,294,226 |
|  |  |
| **LMICs** | **Result** |
| low and middle income country/ or Developing Countries/ | 81,666 |
| Africa, Western/ or Africa/ or Africa, Central/ or South Africa/ or Africa, Northern/ or "Africa South of the Sahara"/ or Africa, Eastern/ or Africa, Southern/ | 105,960 |
| Asia, Northern/ or Asia, Eastern/ or Asia, Western/ or Asia/ or Asia, Southern/ or Asia, Southeastern/ or Asia, Central/ | 42,334 |
| latin america/ | 13,429 |
| (South* Asia or Africa* or "South America*" or "Latin America*" or "Central America*" or Carribean or "Sub-Saharan Africa*" or (Afghanistan or Albania or Algeria or "American Samoa" or Angola or Argentina or "Argentine Republic” or Algeria or Armenia" or Azerbaijan or Africa or “South America” of Bangladesh or Belarus or Byelarus or Belorussia or Belize or Benin or Bhutan or Bolivia or Bosnia or Herzegovina or Hercegovina or "Bosnia and Herzegovina" or Botswana or Brazil or Bulgaria or "Burkina Faso" or Burundi or "Cabo Verde" or "Cape Verde" or Cambodia or Cameroon or "Central African Republic" or Chad or China or Colombia or Comoros or Congo or "Costa Rica" or "Cote d'Ivoire" or "Ivory Coast" or Cuba or Djibouti or Dominica or "Dominican Republic" or Ecuador or Egypt or "El Salvador" or "Equatorial Guinea" or Eritrea or Eswatini or Ethiopia or Fiji or Gabon or Gambia or Georgia or Ghana or Grenada or Grenadines or Guatemala or Guinea or Guinea-Bissau or Guyana or Haiti or Honduras or India or Indonesia or Iran or Iraq or Jamaica or Jordan or Kazakhstan or Kenya or Kiribati or "North Korea" or "DPR Korea" or Kosovo or Kyrgyz or Kyrgyzstan or "Kyrgyz Republic" or "Lao PDR" or Laos or Lebanon or Lesotho or Liberia or Libya or Madagascar or Malawi or Malaysia or Malaya or Maldives or Mali or "Marshall Islands" or Mauritania or Mauritius or Mexico or Micronesia or Moldova or Mongolia or Montenegro or Morocco or Mozambique or Myanmar or Burma or Namibia or Nepal or Niue or Nicaragua or Niger or Nigeria or Macedonia or "North Macedonia" or Pakistan or Palau or Panama or "Papua New Guinea" or Paraguay or Peru or Philippines or "Russian Federation" or Russia or Rwanda or Ruanda or Samoa or Saint Helena or Saint Lucia or "Sao Tome and Principe" or Senegal or Serbia or "Sierra Leone" or "Solomon Islands" or Somalia or "South Africa" or "South Sudan" or Suriname or "Sri Lanka" or "St. Lucia" or "St. Vincent and Grenadines" or Sudan or Suriname or Syria or Tajikistan or Tanzania or Thailand or Timor-Leste or Togo or Tokelau or Tonga or Tunisia or Turkiye or Turkey or Turkmenistan or Tuvalu or Uganda or Ukraine or Uzbekistan or Vanuatu or Vietnam or Venezuela or "Wallis and Futuna" or "West Bank and Gaza" or Palestine or Gaza or "West Bank" or Yemen or Zambia or Zimbabwe)).mp. |  |
| Total LMICs | 2,428,386 |
| **Total:** | 454 |

**Cochrane (Central Register for Clinical Trials)**

| **Sample of Population** | **Result** |
| --- | --- |
| barefoot doctor*.mp | 0 |
| consejero.mp | 0 |
| Caregivers/ or caregiver*.mp | 19,054 |
| Community adj3 (worker* or advisor* or advocate* or educator* or visitor* or attendant* or aide or aides or support* or person* or helper* or carer* or caregiver* or consultant* or advisor* or counselor* or counsellor* or assistant* or staff or facilitator* or nurse* or practitioner* or personnel* or volunteer*) | 7,580 |
| community health workers/ | 708 |
| Health auxiliary.mp | 827 |
| (health cadres or cadres or Kader).mp. | 84 |
| health educators.mp. or Health Educators/ | 296 |
| Home Health Aides/ or health aides.mp | 56 |
| (mental health personnel or unlicensed health personnel).mp | 12 |
| (indigenous* adj2 volunteer*).mp | 2 |
| (informal adj3 (caregiver* or "care giver" or "care givers" or carer*)) | 1,003 |
| ((lady health workers or lady*) adj3 worker*).mp | 65 |
| (lay adj3 (worker* or advisor* or advocate* or educator* or visitor* or attendant* or aide or aides or support* or person* or facilitator* or helper* or carer* or caregiver* or "care giver" or "care givers" or consultant* or advisor* or counselor* or counsellor* or assistant* or staff or facilitator* or practitioner* or personnel* or volunteer*)) | 894 |
| lay health worker*.mp | 202 |
| (non medical adj3 (worker* or visitor* or attendant* or aide or aides or support* or person* or helper* or carer* or caregiver* or "care giver" or "care givers" or consultant* or advisor* or counselor* or counsellor* or assistant* or personnel* or staff)) | 77 |
| (nonprofessional* adj3 (worker* or visitor* or attendant* or aide or aides or support* or person* or helper* or carer* or caregiver* or "care giver" or "care givers" or consultant* or advisor* or counselor* or counsellor* or assistant* or staff)) | 23 |
| (Non-specialist* adj3 (worker* or advisor* or advocate* or educator* or visitor* or attendant* or aide or aides or support* or person* or helper* or carer* or caregiver* or "care giver" or "care givers" or consultant* or advisor* or counselor* or counsellor* or assistant* or staff or facilitator* or practitioner* or personnel* or volunteer*)) | 80 |
| (Paraprofessional* adj3 (worker* or advisor* or advocate* or educator* or visitor* or attendant* or aide or aides or support* or person* or helper* or carer* or caregiver* or "care giver" or "care givers" or consultant* or counselor* or counsellor* or assistant* or staff or facilitator* or practitioner* or personnel* or volunteer*)) | 74 |
| (Peer adj3 (worker* or advisor* or advocate* or educator* or visitor* or attendant* or aide or aides or support* or person* or helper* or carer* or caregiver* or "care giver" or "care givers" or consultant* or advisor* or counselor* or counsellor* or assistant* or staff or facilitator* or practitioner* or personnel* or volunteer*)) | 3,007 |
| (untrained adj3 (worker* or visitor* or attendant* or aide or aides or support* or person* or helper* or carer* or caregiver* or care giver* or "care givers" or consultant* or advisor* or counselor* or counsellor* or assistant* or staff or nurse* or doctor* or personnel* or physician* or therapist*)) | 93 |
| (unlicensed adj3 (worker* or visitor* or attendant* or aide or aides or support* or person* or helper* or carer* or caregiver* or "care giver" or "care givers" or consultant* or advisor* or counselor* or counsellor* or assistant* or staff or nurse* or doctor* or personnel* or physician* or therapist*)) | 15 |
| (Village adj3 (worker* or advisor* or advocate* or educator* or visitor* or attendant* or aide or aides or support* or person* or helper* or carer* or caregiver* or "care giver" or "care givers" or consultant* or advisor* or counselor* or counsellor* or assistant* or staff or facilitator* or practitioner* or personnel* or volunteer*)) | 237 |
| (voluntary adj3 (worker* or visitor* or attendant* or aide or aides or support* or person* or helper* or carer* or caregiver* or "care giver" or "care givers" or consultant* or advisor* or counselor* or counsellor* or assistant* or staff)) | 112 |
| volunteers/ or "health volunteers".mp | 700 |
| Total | 35,626 |
|  |  |
| **Phenomenon of Interest (LIPIs)** | **Result** |
| "Delivery of Health Care"/ | 1,368 |
| intervention adj3 delivery | 2,863 |
| treatment adj3 offer.mp | 677 |
| treatment delivery.mp | 578 |
| (low-intensity adj3 intervention).mp | 530 |
| Behavior Therapy/ | 5,732 |
| behavio?r* activation.mp | 1,306 |
| brief adj3 therapy.mp | 1,046 |
| Psychotherapy, Brief/ | 1,150 |
| Cognitive Behavioral Therapy/ | 10,821 |
| Cognitive Restructuring/ | 19 |
| cognitive therapy.mp | 6,992 |
| (cognitive adj3 therapy*).mp | 26,597 |
| emotion regulation.mp. or Emotional Regulation/ | 2,988 |
| web-based intervention.mp. or Internet-Based Intervention/ | 2,019 |
| Mindfulness/ | 2,148 |
| (Stress reduction or stress management).mp | 5,746 |
| Meditation/ | 1,083 |
| Relaxation/ or Muscle Relaxation/ or Relaxation Therapy/ | 2,412 |
| (solution adj3 therapy).mp | 415 |
| (mind body therapy or mind body exercise* or mind-body exercise*).mp. or Mind-Body Therapies/ | 417 |
| (Peer counselling or "support group*" or "group counselling").mp | 2,519 |
| (problem solving adj3 therapy).mp | 643 |
| psychoeducation.mp | 4,043 |
| Psychosocial Intervention/ or (psychosocial adj3 intervention).mp | 2,323 |
| (Psychological intervention or (psychological adj3 intervention)).mp | 3,460 |
| (psychosocial counselling or (psychosocial adj3 counselling)).mp. | 49 |
| psychosocial care.mp | 1,119 |
| Psychotherapeutic Processes/ | 88 |
| support groups.mp. or Self-Help Groups/ | 1,582 |
| Counseling/ or task shifting.mp | 6,036 |
| thinking healthy.mp | 40 |
| Total | 98,809 |
|  |  |
| **Phenomenon of Interest (Mental health issues)** | **Result** |
| mental disorders/ or (mental adj3 (problem* or issue* or disorder*)).mp. | 19,392 |
| depression/ or Depressive Disorder/ or Depressive Disorder, Major/ or mixed mania depression or minor depression or antenatal depression or major depression or long-term depression or depression.mp. or agitated depression or late life depression or postoperative depression or recurrent brief depression or brief depression or mixed depression-dementia or bipolar depression or chronic depression or reactive depression.mp | 101,052 |
| emotional disturbance* or emotional problem* or affective disorder or affective symptom*.mp | 2,864 |
| Mood Disorders/ or emotional problems or emotional distress or emotional disorder.mp | 3,804 |
| affective disorders.mp. or Mood Disorders/ or emotional distress.mp. or Psychological Distress/ | 3,849 |
| Depression, Postpartum/ or perinatal depression.mp. or antenatal depression.mp | 1,258 |
| Anxiety/ or Anxiety, Separation/ or Anxiety Disorders/ or Performance Anxiety/ or Dental Anxiety/ or social anxiety.mp. or separation anxiety.mp. or generalized anxiety disorder.mp. or performance anxiety.mp. or mixed anxiety depression.mp. | 21,716 |
| Occupational Stress/ or Stress Disorders, Post-Traumatic/ or stress.mp. or Stress Disorders, Traumatic, Acute/ or Stress, Psychological/ or Stress Disorders, Traumatic/ or Heat Stress Disorders/ or psychological stress or psychological distress or parental stress.mp | 80,885 |
| psychological problem* or psychological symptoms.mp | 2,939 |
| psychiatric symptoms or psychiatirc disorders.mp | 1,668 |
| common mental disorders.mp | 443 |
| Cognition Disorders/ or mild cognitive disorders.mp. | 3,964 |
| negative emotions or negative feelings or ruminations.mp | 1,650 |
| Problem Behavior/ | 669 |
| panic attack.mp. or Panic Disorder/ | 1,155 |
| Total | 247,8308 |
|  |  |
| **Design, Research type, Evaluation** | **Result** |
| Qualitative Research/ or qualitative studies.mp | 2,808 |
| (mixed adj2 (design or method or research or study)).mp | 4,433 |
| Interview/ or interview*.mp | 48,353 |
| focus group discussions.mp. or Focus Groups/ | 1,628 |
| phenomenology or phenomenological studie*.mp | 209 |
| case studies.mp. or Case Reports/ | 996 |
| experience*.mp | 131,562 |
| perspectives.mp | 4,494 |
| Perception/ or perception*.mp | 38,225 |
| opinion*.mp. or Attitude/ | 10,194 |
| view*.mp | 26,034 |
| grounded theory.mp. or Grounded Theory/ | 452 |
| Total | 269,388 |
|  |  |
| **LMICs** | **Result** |
| Developing Countries/ or (low and middle income countries).mp. | 2,977 |
| Africa, Western/ or Africa, Northern/ or South Africa/ or Africa, Eastern/ or Africa, Central/ or "Africa South of the Sahara"/ or Africa/ or Africa, Southern/ | 2,442 |
| Asia, Central/ or Asia/ or Asia, Western/ or Asia, Southeastern/ | 1,068 |
| Latin America/ or south and central america.mp | 432 |
| (South* Asia or Africa* or "South America*" or "Latin America*" or "Central America*" or Carribean or "Sub-Saharan Africa*" or (Afghanistan or Albania or Algeria or "American Samoa" or Angola or Argentina or "Argentine Republic” or Algeria or Armenia" or Azerbaijan or Africa or “South America” of Bangladesh or Belarus or Byelarus or Belorussia or Belize or Benin or Bhutan or Bolivia or Bosnia or Herzegovina or Hercegovina or "Bosnia and Herzegovina" or Botswana or Brazil or Bulgaria or "Burkina Faso" or Burundi or "Cabo Verde" or "Cape Verde" or Cambodia or Cameroon or "Central African Republic" or Chad or China or Colombia or Comoros or Congo or "Costa Rica" or "Cote d'Ivoire" or "Ivory Coast" or Cuba or Djibouti or Dominica or "Dominican Republic" or Ecuador or Egypt or "El Salvador" or "Equatorial Guinea" or Eritrea or Eswatini or Ethiopia or Fiji or Gabon or Gambia or Georgia or Ghana or Grenada or Grenadines or Guatemala or Guinea or Guinea-Bissau or Guyana or Haiti or Honduras or India or Indonesia or Iran or Iraq or Jamaica or Jordan or Kazakhstan or Kenya or Kiribati or "North Korea" or "DPR Korea" or Kosovo or Kyrgyz or Kyrgyzstan or "Kyrgyz Republic" or "Lao PDR" or Laos or Lebanon or Lesotho or Liberia or Libya or Madagascar or Malawi or Malaysia or Malaya or Maldives or Mali or "Marshall Islands" or Mauritania or Mauritius or Mexico or Micronesia or Moldova or Mongolia or Montenegro or Morocco or Mozambique or Myanmar or Burma or Namibia or Nepal or Niue or Nicaragua or Niger or Nigeria or Macedonia or "North Macedonia" or Pakistan or Palau or Panama or "Papua New Guinea" or Paraguay or Peru or Philippines or "Russian Federation" or Russia or Rwanda or Ruanda or Samoa or Saint Helena or Saint Lucia or "Sao Tome and Principe" or Senegal or Serbia or "Sierra Leone" or "Solomon Islands" or Somalia or "South Africa" or "South Sudan" or Suriname or "Sri Lanka" or "St. Lucia" or "St. Vincent and Grenadines" or Sudan or Suriname or Syria or Tajikistan or Tanzania or Thailand or Timor-Leste or Togo or Tokelau or Tonga or Tunisia or Turkiye or Turkey or Turkmenistan or Tuvalu or Uganda or Ukraine or Uzbekistan or Vanuatu or Vietnam or Venezuela or "Wallis and Futuna" or "West Bank and Gaza" or Palestine or Gaza or "West Bank" or Yemen or Zambia or Zimbabwe)).mp. |  |
| Total LMICs | 125,720 |
| **Total:** | 444 |

**ASSIA**

| **Sample of Population** | **Result** |
| --- | --- |
| noft(barefoot doctors) | 13 |
| noft(consejero) | 3 |
| MAINSUBJECT.EXACT("Caregivers") | 17,612 |
| noft(Community NEAR/3 (workers or advisors or advocates or educators or visitors)) | 3,287 |
| noft(Community NEAR/3 (attendants or aides or supports or persons or helpers or carers or caregivers or consultants)) | 10,167 |
| noft(Community NEAR/3 (advisors or counselors or counsellors or assistants or staff or facilitators or nurses or practitioners or personnels or volunteers)) | 16,353 |
| MAINSUBJECT.EXACT("Community health care") | 3,384 |
| noft(health auxiliary) | 214 |
| noft(Health cadres or cadres or kader) | 729 |
| noft(health educators) | 5,916 |
| MAINSUBJECT.EXACT("Home health aides") | 36 |
| noft(unlicensed health care personnel) | 202 |
| noft(indigenous volunteer) | 21 |
| noft(informal NEAR/3 (caregiver* or "care giver" or "care givers" or carer*)) | 1,960 |
| MAINSUBJECT.EXACT("Informal helping networks") | 57 |
| noft(unlicensed health care personnel) | 202 |
| noft(indigenous volunteer) | 21 |
| noft(informal NEAR/3 (caregiver* or "care giver" or "care givers" or carer*)) | 1,960 |
| MAINSUBJECT.EXACT("Informal helping networks") | 57 |
| noft(lady* NEAR/3 worker*) | 14 |
| noft(lay NEAR/3 (workers or advisors or advocates or educators or visitors or attendants)) | 341 |
| noft(lay NEAR/3 (carers or caregivers or "care giver" or "care givers" or consultants or advisors)) | 210 |
| noft(lay NEAR/3 (counselors or counsellors or assistants or staff or facilitators or practitioners or personnels)) | 144 |
| noft(lay NEAR/3 (volunteers or aide or aides or support or persons or facilitators or helpers)) | 1,031 |
| noft(lay health workers) | 335 |
| noft(non medical NEAR/3 (workers or visitors or attendants or aide or aides)) | 57 |
| noft(non medical NEAR/3 ("care givers" or consultants or advisors or counselors)) | 32 |
| noft(non medical NEAR/3 (supports or persons or helpers or carers or caregivers or "care giver")) | 308 |
| noft(non medical NEAR/3 (counsellors or assistants or personnels or staff)) | 2,406 |
| noft(nonprofessional NEAR/3 (workers or visitors or attendants or aides)) | 12 |
| noft(nonprofessional NEAR/3 (caregivers or consultants)) | 19 |
| noft(nonprofessional NEAR/3 (aides or supports or persons or helpers or carers or caregivers)) | 40 |
| noft(nonprofessional NEAR/3 (advisors or counselors or counsellors or assistants or staff)) | 8 |
| noft(Non-specialist NEAR/3 (workers or advisors or advocates or educators or visitors)) | 23 |
| noft(Non-specialist NEAR/3 (persons or helpers or carers or caregivers or "caregiver")) | 3 |
| noft(Non-specialist NEAR/3 (assistants or staff or facilitators or practitioners or personnels or volunteers)) | 32 |
| noft(Non-specialist NEAR/3 ("caregivers" or consultants or advisors or counselors or counsellors)) | 4 |
| noft(Non-specialist NEAR/3 (attendants or aide or aides or supports)) | 6 |
| noft(Paraprofessional NEAR/3 (workers or advisors or advocates or educators or visitors)) | 43 |
| noft(Paraprofessional NEAR/3 (supports or persons or helpers or carers or caregivers)) | 41 |
|  |  |
| Total | 65,061 |
|  |  |
| **Phenomenon of Interest (LIPIs)** | **Result** |
| MAINSUBJECT.EXACT("Health care delivery") | 4,746 |
| MAINSUBJECT.EXACT("Intervention") | 37,645 |
| noft(intervention NEAR/3 delivery or treatment offer) | 7,417 |
| noft(treatment delivery) | 7,115 |
| noft(Low-intensity interventions or low-intensity NEAR/3 interventions) | 377 |
| MAINSUBJECT.EXACT("Behavioural psychotherapy") OR MAINSUBJECT.EXACT("Behaviour therapy") | 741 |
| noft(Behavior activations) | 2,028 |
| MAINSUBJECT.EXACT("Brief therapy") or noft(brief NEAR/3 therapy) | 1,092 |
| noft(brief psychotherapy) | 1,624 |
| MAINSUBJECT.EXACT("Cognitive therapy") | 4,466 |
| MAINSUBJECT.EXACT("Cognitive restructuring") | 132 |
| MAINSUBJECT.EXACT("Cognitive behavioral therapy") OR MAINSUBJECT.EXACT("Cognitive psychotherapy") or noft(cognitive therapy) | 19,677 |
| noft(emotion regulation or emotion regulation therapy or emotion regulation techniques) | 6,873 |
| noft(Internet based interventions or web-based interventions or digital interventions) | 5,659 |
| noft(mindfulness) | 4,047 |
| MAINSUBJECT.EXACT("Relaxation") | 954 |
| noft(stress reduction or stress management) or MAINSUBJECT.EXACT("Stress management training") | 9,608 |
| MAINSUBJECT.EXACT("Meditation") or noft(relaxation therapy or muscle relaxation therapy) | 2,030 |
| noft(solution NEAR/3 therapy) | 366 |
| noft(mind body therapy or mind body exercise or mind-body exercise or Mind-Body Therapies) | 544 |
| MAINSUBJECT.EXACT("Solutions based brief therapy") | 98 |
| noft(peer NEAR/2 counseling or "support group" or "group counselling") or MAINSUBJECT.EXACT("Group counseling") | 2,511 |
| MAINSUBJECT.EXACT("Counseling") or noft(community counselling) | 14,395 |
| noft(problem solving therapy or problem solving NEAR/2 therapy) | 1,292 |
| MAINSUBJECT.EXACT("Psychoeducational treatment") | 956 |
| noft(psychoeducation) | 1,776 |
| MAINSUBJECT.EXACT("Community health care") OR MAINSUBJECT.EXACT("Mental health care") or noft("community mental health care") | 21,374 |
| MAINSUBJECT.EXACT("Psychosocial intervention") or noft(psychosocial NEAR/3 intervention) | 3,038 |
| MAINSUBJECT.EXACT("Psychosocial therapy") | 478 |
| MAINSUBJECT.EXACT("Psychological intervention") or noft(psychological NEAR/3 interventions) | 3,541 |
| noft(psychosocial NEAR/3 counselling) | 167 |
|  |  |
| Total | 177,723 |
|  |  |
| **Phenomenon of Interest (Mental health issues)** | **Result** |
| MAINSUBJECT.EXACT("Mental disorders") or noft(mental NEAR/2 (issues or disorders or problems or illness)) | 60,347 |
| MAINSUBJECT.EXACT("Mental depression") or MAINSUBJECT.EXACT("Mental depression") or MAINSUBJECT.EXACT("Parental depression") OR MAINSUBJECT.EXACT("Paternal depression") OR MAINSUBJECT.EXACT("Mental disorders") OR MAINSUBJECT.EXACT("Mental depression") OR MAINSUBJECT.EXACT("Maternal depression") or noft(depression or "mixed mania and depression" or minor depression or antenatal depression or major depression or long-term depression or agitated depression or late life depression or recurrent brief depression or "mixed depression and dementia" or bipolar depression or "mixed anxiety and depression" or chronic depression or reactive depression) | 106,249 |
| MAINSUBJECT.EXACT("Emotional distress") OR MAINSUBJECT.EXACT("Emotional sensitivity") OR MAINSUBJECT.EXACT("Emotional disturbance") OR MAINSUBJECT.EXACT("Emotional instability") OR MAINSUBJECT.EXACT("Emotional-Behavioural problems") OR MAINSUBJECT.EXACT("Emotional disorders") or noft(emotional problems or affective disorders or affective symptoms or mood disorders or emotional distress or emotional distress or psychological distress) | 51,218 |
|  |  |
|  |  |
| MAINSUBJECT.EXACT("Postpartum women") OR MAINSUBJECT.EXACT("Postpartum depression") or noft(perinatal depressions) | 3,275 |
| MAINSUBJECT.EXACT("Psychological problems") or noft(psychological symptoms) | 26,169 |
| noft(psychiatric disorders or psychiatric symptoms) or MAINSUBJECT.EXACT("Neuropsychiatric symptoms") OR MAINSUBJECT.EXACT("Psychiatric symptoms") | 35,046 |
| noft(common mental disorders) | 7,277 |
| MAINSUBJECT.EXACT("Mild cognitive disorders") OR MAINSUBJECT.EXACT("Cognitive disorders") or noft(cognitive impairment) | 11,011 |
| MAINSUBJECT.EXACT("Negative emotions") OR MAINSUBJECT.EXACT("Negative affect") OR (MAINSUBJECT.EXACT("Rumination") OR MAINSUBJECT.EXACT("Obsessional ruminations")) | 5,766 |
| noft(problem behaviors or behavioural problems) | 50,320 |
| MAINSUBJECT.EXACT("Panic disorders") or noft(panic attack) | 1,994 |
| Total | 251,534 |
|  |  |
| **Design, Research type, Evaluation** | **Result** |
| MAINSUBJECT.EXACT("Qualitative research") or noft(qualitative studies or qualitative analysis) | 72,311 |
| noft(mixed research study or mixed method or mixed design or mixed study design) | 20,907 |
| MAINSUBJECT.EXACT("Interviews") | 12,516 |
| MAINSUBJECT.EXACT("Phenomenology") or noft(phenomenological studies or phenomenological approach) | 7,959 |
| MAINSUBJECT.EXACT("Case studies") or noft(case report) | 23,002 |
| MAINSUBJECT.EXACT("Focus groups") or noft(focus group discussion or FGD) | 10,716 |
| MAINSUBJECT.EXACT("Experiences") | 1,865 |
| MAINSUBJECT.EXACT("Perspectives") | 1,149 |
| MAINSUBJECT.EXACT("Perceptions") | 30,623 |
| MAINSUBJECT.EXACT("Opinions") | 453 |
| noft(views) | 54,648 |
| Total | 193,191 |
|  |  |
| **LMICs** | **Result** |
| noft(low and middle income countries) or MAINSUBJECT.EXACT("Developing countries--LDCs") | 8,216 |
| africa | 36,911 |
| asia | 25,265 |
| Asia, Southeastern | 347 |
| latin america | 9,793 |
| (South* Asia or Africa* or "South America*" or "Latin America*" or "Central America*" or Carribean or "Sub-Saharan Africa*" or (Afghanistan or Albania or Algeria or "American Samoa" or Angola or Argentina or "Argentine Republic” or Algeria or Armenia" or Azerbaijan or Africa or “South America” of Bangladesh or Belarus or Byelarus or Belorussia or Belize or Benin or Bhutan or Bolivia or Bosnia or Herzegovina or Hercegovina or "Bosnia and Herzegovina" or Botswana or Brazil or Bulgaria or "Burkina Faso" or Burundi or "Cabo Verde" or "Cape Verde" or Cambodia or Cameroon or "Central African Republic" or Chad or China or Colombia or Comoros or Congo or "Costa Rica" or "Cote d'Ivoire" or "Ivory Coast" or Cuba or Djibouti or Dominica or "Dominican Republic" or Ecuador or Egypt or "El Salvador" or "Equatorial Guinea" or Eritrea or Eswatini or Ethiopia or Fiji or Gabon or Gambia or Georgia or Ghana or Grenada or Grenadines or Guatemala or Guinea or Guinea-Bissau or Guyana or Haiti or Honduras or India or Indonesia or Iran or Iraq or Jamaica or Jordan or Kazakhstan or Kenya or Kiribati or "North Korea" or "DPR Korea" or Kosovo or Kyrgyz or Kyrgyzstan or "Kyrgyz Republic" or "Lao PDR" or Laos or Lebanon or Lesotho or Liberia or Libya or Madagascar or Malawi or Malaysia or Malaya or Maldives or Mali or "Marshall Islands" or Mauritania or Mauritius or Mexico or Micronesia or Moldova or Mongolia or Montenegro or Morocco or Mozambique or Myanmar or Burma or Namibia or Nepal or Niue or Nicaragua or Niger or Nigeria or Macedonia or "North Macedonia" or Pakistan or Palau or Panama or "Papua New Guinea" or Paraguay or Peru or Philippines or "Russian Federation" or Russia or Rwanda or Ruanda or Samoa or Saint Helena or Saint Lucia or "Sao Tome and Principe" or Senegal or Serbia or "Sierra Leone" or "Solomon Islands" or Somalia or "South Africa" or "South Sudan" or Suriname or "Sri Lanka" or "St. Lucia" or "St. Vincent and Grenadines" or Sudan or Suriname or Syria or Tajikistan or Tanzania or Thailand or Timor-Leste or Togo or Tokelau or Tonga or Tunisia or Turkiye or Turkey or Turkmenistan or Tuvalu or Uganda or Ukraine or Uzbekistan or Vanuatu or Vietnam or Venezuela or "Wallis and Futuna" or "West Bank and Gaza" or Palestine or Gaza or "West Bank" or Yemen or Zambia or Zimbabwe)).mp. |  |
| Total LMICs | 249,145 |
| **Total:** | 793 |

**Proquest Theses and Disertations**

| **Sample of Population** | **Result** |
| --- | --- |
| noft(barefoot doctors) | 13 |
| noft(consejero) | 44 |
| MAINSUBJECT.EXACT("Caregivers") | 2,981 |
| noft(Community NEAR/3 (workers or advisors or advocates or educators or visitors)) | 4,427 |
| noft(Community NEAR/3 (attendants or aides or supports or persons or helpers or carers or caregivers or consultants)) | 15,485 |
| noft(Community NEAR/3 (advisors or counselors or counsellors or assistants or staff or facilitators or nurses or practitioners or personnels or volunteers)) | 6,892 |
| MAINSUBJECT.EXACT("Community health care") | 168 |
| noft(health auxiliary) | 13 |
| noft(Health cadres or cadres or kader) | 999 |
| noft(health educators) | 11,604 |
| MAINSUBJECT.EXACT("Home health aides") | 9,764 |
| noft(unlicensed health care personnel) | 0 |
| noft(indigenous volunteer) | 39 |
| noft(informal NEAR/3 (caregiver* or "care giver" or "care givers" or carer*)) | 123 |
| MAINSUBJECT.EXACT("Informal helping networks") | 1,154 |
| noft(unlicensed health care personnel) | 0 |
| noft(indigenous volunteer) | 12 |
| noft(informal NEAR/3 (caregiver* or "care giver" or "care givers" or carer*)) | 305 |
| MAINSUBJECT.EXACT("Informal helping networks") | 94 |
| noft(lady* NEAR/3 worker*) | 336 |
| noft(lay NEAR/3 (workers or advisors or advocates or educators or visitors or attendants)) | 1,885 |
| noft(lay NEAR/3 (carers or caregivers or "care giver" or "care givers" or consultants or advisors)) | 256 |
| noft(lay NEAR/3 (counselors or counsellors or assistants or staff or facilitators or practitioners or personnels)) | 96 |
| noft(lay NEAR/3 (volunteers or aide or aides or support or persons or facilitators or helpers)) | 31 |
| noft(lay health workers) | 567 |
| noft(non medical NEAR/3 (workers or visitors or attendants or aide or aides)) | 1,762 |
| noft(non medical NEAR/3 ("care givers" or consultants or advisors or counselors)) | 35 |
| noft(non medical NEAR/3 (supports or persons or helpers or carers or caregivers or "care giver")) | 9 |
| noft(non medical NEAR/3 (counsellors or assistants or personnels or staff)) | 34 |
| noft(nonprofessional NEAR/3 (workers or visitors or attendants or aides)) | 30 |
| noft(nonprofessional NEAR/3 (caregivers or consultants)) | 7 |
| noft(nonprofessional NEAR/3 (aides or supports or persons or helpers or carers or caregivers)) | 3 |
| noft(nonprofessional NEAR/3 (advisors or counselors or counsellors or assistants or staff)) | 21 |
| noft(Non-specialist NEAR/3 (workers or advisors or advocates or educators or visitors)) | 3 |
| noft(Non-specialist NEAR/3 (persons or helpers or carers or caregivers or "caregiver")) | 6 |
| noft(Non-specialist NEAR/3 (assistants or staff or facilitators or practitioners or personnels or volunteers)) | 110 |
| noft(Non-specialist NEAR/3 ("caregivers" or consultants or advisors or counselors or counsellors)) | 141 |
| noft(Non-specialist NEAR/3 (attendants or aide or aides or supports)) | 194 |
| noft(Paraprofessional NEAR/3 (workers or advisors or advocates or educators or visitors)) | 83 |
| noft(Paraprofessional NEAR/3 (supports or persons or helpers or carers or caregivers)) | 64 |
|  |  |
| Total | 67,192 |
|  |  |
| **Phenomenon of Interest (LIPIs)** | **Result** |
| MAINSUBJECT.EXACT("Health care delivery") | 426 |
| MAINSUBJECT.EXACT("Intervention") | 4835 |
| noft(intervention NEAR/3 delivery or treatment offer) | 16,917 |
| noft(treatment delivery) | 14,755 |
| noft(Low-intensity interventions or low-intensity NEAR/3 interventions) | 297 |
| MAINSUBJECT.EXACT("Behavioural psychotherapy") OR MAINSUBJECT.EXACT("Behaviour therapy") | 0 |
| noft(Behavior activations) | 12,678 |
| MAINSUBJECT.EXACT("Brief therapy") or noft(brief NEAR/3 therapy) | 607 |
| noft(brief psychotherapy) | 2,987 |
| MAINSUBJECT.EXACT("Cognitive therapy") | 12,769 |
| MAINSUBJECT.EXACT("Cognitive restructuring") | 4 |
| MAINSUBJECT.EXACT("Cognitive behavioral therapy") OR MAINSUBJECT.EXACT("Cognitive psychotherapy") or noft(cognitive therapy) | 24,753 |
| noft(emotion regulation or emotion regulation therapy or emotion regulation techniques) | 5,851 |
| noft(Internet based interventions or web-based interventions or digital interventions) | 4,822 |
| noft(mindfulness) | 11,704 |
| MAINSUBJECT.EXACT("Relaxation") | 1,715 |
| noft(stress reduction or stress management) or MAINSUBJECT.EXACT("Stress management training") | 0 |
| MAINSUBJECT.EXACT("Meditation") or noft(relaxation therapy or muscle relaxation therapy) | 1,255 |
| noft(solution NEAR/3 therapy) | 1,202 |
| noft(mind body therapy or mind body exercise or mind-body exercise or Mind-Body Therapies) | 1,145 |
| MAINSUBJECT.EXACT("Solutions based brief therapy") | 0 |
| noft(peer NEAR/2 counseling or "support group" or "group counselling") or MAINSUBJECT.EXACT("Group counseling") | 2,563 |
| MAINSUBJECT.EXACT("Counseling") or noft(community counselling) | 64 |
| noft(problem solving therapy or problem solving NEAR/2 therapy) | 4,746 |
| MAINSUBJECT.EXACT("Psychoeducational treatment") | 11,704 |
| noft(psychoeducation) | 1,715 |
| MAINSUBJECT.EXACT("Community health care") OR MAINSUBJECT.EXACT("Mental health care") or noft("community mental health care") | 0 |
| MAINSUBJECT.EXACT("Psychosocial intervention") or noft(psychosocial NEAR/3 intervention) | 1,255 |
| MAINSUBJECT.EXACT("Psychosocial therapy") | 1,202 |
| MAINSUBJECT.EXACT("Psychological intervention") or noft(psychological NEAR/3 interventions) | 1,145 |
| noft(psychosocial NEAR/3 counselling) | 0 |
|  |  |
| Total | 263,382 |
|  |  |
| **Phenomenon of Interest (Mental health issues)** | **Result** |
| MAINSUBJECT.EXACT("Mental disorders") or noft(mental NEAR/2 (issues or disorders or problems or illness)) | 24,353 |
| MAINSUBJECT.EXACT("Mental depression") or MAINSUBJECT.EXACT("Mental depression") or MAINSUBJECT.EXACT("Parental depression") OR MAINSUBJECT.EXACT("Paternal depression") OR MAINSUBJECT.EXACT("Mental disorders") OR MAINSUBJECT.EXACT("Mental depression") OR MAINSUBJECT.EXACT("Maternal depression") or noft(depression or "mixed mania and depression" or minor depression or antenatal depression or major depression or long-term depression or agitated depression or late life depression or recurrent brief depression or "mixed depression and dementia" or bipolar depression or "mixed anxiety and depression" or chronic depression or reactive depression) | 67,245 |
| MAINSUBJECT.EXACT("Emotional distress") OR MAINSUBJECT.EXACT("Emotional sensitivity") OR MAINSUBJECT.EXACT("Emotional disturbance") OR MAINSUBJECT.EXACT("Emotional instability") OR MAINSUBJECT.EXACT("Emotional-Behavioural problems") OR MAINSUBJECT.EXACT("Emotional disorders") or noft(emotional problems or affective disorders or affective symptoms or mood disorders or emotional distress or emotional distress or psychological distress) | 38,161 |
| MAINSUBJECT.EXACT("Postpartum women") OR MAINSUBJECT.EXACT("Postpartum depression") or noft(perinatal depressions) | 681 |
| MAINSUBJECT.EXACT("Psychological problems") or noft(psychological symptoms) | 44,925 |
| noft(psychiatric disorders or psychiatric symptoms) or MAINSUBJECT.EXACT("Neuropsychiatric symptoms") OR MAINSUBJECT.EXACT("Psychiatric symptoms") | 56,155 |
| noft(common mental disorders) | 14,590 |
| MAINSUBJECT.EXACT("Mild cognitive disorders") OR MAINSUBJECT.EXACT("Cognitive disorders") or noft(cognitive impairment) | 11,593 |
| MAINSUBJECT.EXACT("Negative emotions") OR MAINSUBJECT.EXACT("Negative affect") OR (MAINSUBJECT.EXACT("Rumination") OR MAINSUBJECT.EXACT("Obsessional ruminations")) | 3,698 |
| noft(problem behaviors or behavioural problems) | 11,902 |
| MAINSUBJECT.EXACT("Panic disorders") or noft(panic attack) | 0 |
| Total | 284,421 |
|  |  |
| **Design, Research type, Evaluation** | **Result** |
| MAINSUBJECT.EXACT("Qualitative research") or noft(qualitative studies or qualitative analysis) | 259,597 |
| noft(mixed research study or mixed method or mixed design or mixed study design) | 104,573 |
| MAINSUBJECT.EXACT("Interviews") | 2,929 |
| MAINSUBJECT.EXACT("Phenomenology") or noft(phenomenological studies or phenomenological approach) | 45,251 |
| MAINSUBJECT.EXACT("Case studies") or noft(case report) | 44,323 |
| MAINSUBJECT.EXACT("Focus groups") or noft(focus group discussion or FGD) | 16,659 |
| MAINSUBJECT.EXACT("Experiences") | 515729 |
| MAINSUBJECT.EXACT("Perspectives") | 277,287 |
| MAINSUBJECT.EXACT("Perceptions") | 15,500 |
| MAINSUBJECT.EXACT("Opinions") | 116 |
| noft(views) | 268,109 |
| Total | 1,132,411 |
|  |  |
| **LMICs** | **Result** |
| noft(low and middle income countries) or MAINSUBJECT.EXACT("Developing countries--LDCs") | 11,136 |
| africa | 83,020 |
| asia | 19,367 |
| Asia, Southeastern | 41,372 |
| latin america | 11,555 |
| (South* Asia or Africa* or "South America*" or "Latin America*" or "Central America*" or Carribean or "Sub-Saharan Africa*" or (Afghanistan or Albania or Algeria or "American Samoa" or Angola or Argentina or "Argentine Republic” or Algeria or Armenia" or Azerbaijan or Africa or “South America” of Bangladesh or Belarus or Byelarus or Belorussia or Belize or Benin or Bhutan or Bolivia or Bosnia or Herzegovina or Hercegovina or "Bosnia and Herzegovina" or Botswana or Brazil or Bulgaria or "Burkina Faso" or Burundi or "Cabo Verde" or "Cape Verde" or Cambodia or Cameroon or "Central African Republic" or Chad or China or Colombia or Comoros or Congo or "Costa Rica" or "Cote d'Ivoire" or "Ivory Coast" or Cuba or Djibouti or Dominica or "Dominican Republic" or Ecuador or Egypt or "El Salvador" or "Equatorial Guinea" or Eritrea or Eswatini or Ethiopia or Fiji or Gabon or Gambia or Georgia or Ghana or Grenada or Grenadines or Guatemala or Guinea or Guinea-Bissau or Guyana or Haiti or Honduras or India or Indonesia or Iran or Iraq or Jamaica or Jordan or Kazakhstan or Kenya or Kiribati or "North Korea" or "DPR Korea" or Kosovo or Kyrgyz or Kyrgyzstan or "Kyrgyz Republic" or "Lao PDR" or Laos or Lebanon or Lesotho or Liberia or Libya or Madagascar or Malawi or Malaysia or Malaya or Maldives or Mali or "Marshall Islands" or Mauritania or Mauritius or Mexico or Micronesia or Moldova or Mongolia or Montenegro or Morocco or Mozambique or Myanmar or Burma or Namibia or Nepal or Niue or Nicaragua or Niger or Nigeria or Macedonia or "North Macedonia" or Pakistan or Palau or Panama or "Papua New Guinea" or Paraguay or Peru or Philippines or "Russian Federation" or Russia or Rwanda or Ruanda or Samoa or Saint Helena or Saint Lucia or "Sao Tome and Principe" or Senegal or Serbia or "Sierra Leone" or "Solomon Islands" or Somalia or "South Africa" or "South Sudan" or Suriname or "Sri Lanka" or "St. Lucia" or "St. Vincent and Grenadines" or Sudan or Suriname or Syria or Tajikistan or Tanzania or Thailand or Timor-Leste or Togo or Tokelau or Tonga or Tunisia or Turkiye or Turkey or Turkmenistan or Tuvalu or Uganda or Ukraine or Uzbekistan or Vanuatu or Vietnam or Venezuela or "Wallis and Futuna" or "West Bank and Gaza" or Palestine or Gaza or "West Bank" or Yemen or Zambia or Zimbabwe)).mp. |  |
| Total LMICs | 1,234,355 |
| **Total:** | 490 |
