# Supplementary material for: Delivering low-intensity psychological interventions in low- and middle-income countries: A systematic review and meta-ethnographic synthesis of lay health workers’ experiences
Source: Glob Ment Health (Camb). 2026 Jul 7;13:e157. doi: 10.1017/gmh.2026.10271 (PMC13433339; doi:10.1017/gmh.2026.10271)
Supplement: Supplementary file 1 [file S2054425126102714sup001.zip › Supplementary material 3 - Data extraction form.docx]

**Supplementary Table 1. Characteristics of studies**

| **Author (s)** | **Countries** | **Title** | **Research aim** | **Design, data collection, and analysis** | **Number of Participants (LHWs)** | **Gender and age range of LHWs** | **Key findings** |
| --- | --- | --- | --- | --- | --- | --- | --- |
| Abas et.al. (2016) | Zimbabwe | ‘Opening up the mind’: problem-solving therapy delivered by female lay health workers to improve access to evidence-based care for depression and other common mental disorders through the Friendship Bench Project in Zimbabwe | to learn lessons about the acceptability and implementation of a collaborative care intervention driven by trained and supervised existing community health lay workers in a low-income setting in Sub-Saharan Africa. The study also aims to inform potential scale-up through existing platforms in Zimbabwe and other low-income settings. | - Mixed-methods study (qualitative component extracted), - routine data on patients’ attendance, FGDs - Quantitative analysis and thematic analysis | 40 to 60 | Female, (Mean of age: 62. | - Positive Reception: The collaborative care intervention for CMDs was well-received by patients. - Rewarding for LHWs: LHWs found the intervention to be personally rewarding. - Cost-Effectiveness: The intervention proved to be cost-effective over time. |
| Ahmed et al. (2024) | Botswana | Implementation Determinants of Problem‑Solving Therapy Delivered by Near‑Peer Lay Counselors for Youth Living with HIV in Botswana: Lay Counsellor Perspectives | To adapt and pilot-test the Friendship Bench model (a lay counselor-delivered mental health intervention) for adolescents living with HIV (ALHIV) in Botswana.  To evaluate feasibility, acceptability, and preliminary effectiveness of the adapted intervention, called Safe Haven, in reducing symptoms of depression and anxiety among ALHIV. | - Qualitative design - Interviews - Thematic analysis | 8 | Mixed gender (range of age 21–28, Mean= 24) | Barriers to Implementation of Friendship Bench   - Client reticence and confidentiality concerns - Parental disapproval - Limited accessibility - Counsellor psychological burden - Scheduling conflicts - Inadequate financial resources   Facilitators of Implementation   - Peer delivery increased acceptability - Strong counsellor commitment and perceived value |
| Atif (2015) | India, Pakistan | The acceptability of peer volunteers as delivery agents of a psychosocial intervention for perinatal depression in rural Pakistan: a qualitative study (Doctoral thesis) | to gain an in-depth understanding of the issues pertaining to the acceptability of the PVs, delivering a psychological intervention for perinatal depression in a rural Pakistan. | - Qualitative design, - interviews, - framework analysis | 8 | Female,  (range: 26–40 years, Mean: 33) | - Peer volunteers are a feasible, acceptable workforce for perinatal depression care in rural Pakistan. - Can help reduce the treatment gap in low-resource settings. - Requires careful selection (e.g., CHAMPS checklist). - Needs strong training and supervision. - Should be integrated with PHC/LHW systems. - Must address stigma and family resistance. - Requires support for volunteer motivation and wellbeing. - Supports broader task-shifting and women’s empowerment. |
| Atif et al. (2016) | India, Pakistan | Barefoot therapists: barriers and facilitators to delivering maternal mental health care through peer volunteers in Pakistan: a qualitative study | to explore the facilitators and barriers to the acceptability of peer volunteers (PVs)—volunteer lay women from the community with shared socio-demographic and life experiences with the target population—as delivery agents of a psychosocial intervention for perinatal depression in a rural area of Pakistan. |  |  |  | Factors Enhancing LHWs' Acceptability:   - Local Presence and Community Reputation - Empathy and Trustworthiness - Connection to Primary Health Care   Acceptability of the Intervention:   - Use of vignettes, pictures, and simple language made the intervention easy to understand. - Peers effectively used behavioural activation techniques. - Shared Life Experiences facilitated the intervention’s delivery. |
| Atif et al. (2017) | India, Pakistan | Mother-to-mother therapy in India and Pakistan: adaptation and feasibility evaluation of the peer-delivered Thinking Healthy Programme | to adapt the THP so that it could be delivered by peers to depressed mothers in the community in two different settings in India and Pakistan, and to test the adapted intervention for feasibility in a sample of peers and mothers in both settings |  |  |  | - Trained community peers can feasibly and acceptably deliver adapted Thinking Healthy Programme interventions. - Applicable across diverse South Asian settings. - Peers are a valuable resource where CHWs are overburdened. - Requires appropriate selection, training, and supervision. - Must address confidentiality and stigma. - Needs context-specific adaptations (e.g., simplified manuals, sustainable supervision). - Has potential for scale-up in low-resource settings. - Can help reduce the perinatal depression treatment gap. |
| Bah et al. (2025) | Sierra Leone | Feasibility, acceptability and preliminary effectiveness of a culturally adapted nonspecialist delivery Problem-Solving Therapy: Friendship Bench Intervention for perinatal psychological distress in Sierra Leone | Determine whether a culturally adapted Problem-Solving Therapy – Friendship Bench Intervention (PST-FBI) can be successfully delivered by nonspecialists (Mother-to-Mother Support Groups, MMSGs) to perinatal women in Sierra Leone.  Explore how acceptable the intervention is to both perinatal women and the lay health workers delivering it, including perceptions of its relevance, complexity, and cultural fit.  Investigate whether the adapted PST-FBI reduces perinatal psychological distress, measured using the Sierra Leone Perinatal Psychological Distress Scale (SLPPDS), compared to usual care. | - Mixed-methods study (qualitative component extracted)PHQ-9, Sierra Leone Perinatal Distress Scale, WHO-5 well-being index - Descriptive statistic, t-Test, thematic analysis | 10 | Female, age was not clearly described | the feasibility, acceptability, and preliminary effectiveness of Problem-Solving Therapy (PST) by trained mothers  Feasibility   - High recruitment and retention - Lay counsellor delivery was practical: - Strong implementation in home and group settings   Acceptability   - Positive reception by participants and counsellors: - Cultural relevance of adapted content: - Strengthened therapeutic alliance:   Preliminary Effectiveness   - Reduction in psychological distress - Improved problem-solving skills and social support |
| Burgess et al. (2025) | Ethiopia | The feasibility and acceptability of implementing simplified cognitive behavioral therapy approaches to support postpartum mental health and address associated social and behavioral barriers to postpartum family planning in Amhara, Ethiopia: a qualitative study | To assess the feasibility and acceptability of implementing a simplified Cognitive Behavioral Therapy (CBT)-based intervention, called Mothers Time, delivered by community health workers to postpartum women in rural Amhara, Ethiopia, who experience mild to moderate symptoms of depression or anxiety and have an unmet need for family planning. | - Qualitative design - Interviews - Thematic analysis | 4 | Female (range of age 25-50) | - Intervention was acceptable to postpartum women; content was engaging and relatable. - Group sessions reduced isolation and encouraged peer support. - Health Extension Workers feasibly delivered simplified CBT after short training, though additional coaching was needed. - Participants reported better emotional coping, increased motivation to seek Family Planning (FP), and improved spousal communication. - Time constraints and limited training were key feasibility challenges. - Some religious barriers to FP remained unaddressed. |
| Chau et al. (2021) | Vietnam | Lay social workers implementing a task-sharing approach to managing depression in Vietnam | The purpose of the study was to identify factors influencing the effectiveness of delivery of Supported Self Management by social collaborators and delineate areas for further attention that are relevant for scale-up | - Qualitative design - Interviews - Thematic analysis | 47 | Mixed gender (78.7% were women), Mean of age 55 (48.97% were over 55) | - A strong relationship between patients and LHWs fosters openness and responsiveness. - LHWs manage program mobilisation, conduct home visits, and provide patient support. - Involvement of the community enhances the effectiveness of the intervention. - Older LHWs face difficulties in learning new knowledge. - Low educational levels among LHWs reduce patient trust. - LHWs balance their work duties with personal responsibilities, such as harvesting |
| Chau et al. (2024) | Vietnam | Client experiences of a task-shifting supported self-management intervention for depression in Vietnam | to explore the contextual facilitators and barriers that impact the client experience of a task-shifting supported self-management (SSM) intervention for depression in Vietnam. |  |  |  | - Social embeddedness enhanced acceptability of the intervention delivered by social collaborators - Social collaborators as bridges, acting as connectors between clients and formal health services, especially for people unfamiliar with or hesitant to seek professional care |
| Chibanda (2016) | Zimbabwe | Development and scaling up of a psychological intervention for common mental disorders among people living with HIV in Zimbabwe: Thesis | To review the current evidence for the effectiveness of psychological interventions for common mental disorders (CMD) among people living with HIV (PLWH) in low- and middle-income countries (LMIC).  To identify the common features of these psychological interventions (e.g. content, theoretical basis, delivery, duration).  To examine, within this literature, the role and effectiveness of task-shifting (use of non-specialist/lay health workers) as a strategy for addressing the treatment gap for CMD in LMIC. | - Qualitative design - Interviews - Thematic analysis | 7 | Female (Mean of age: 58) | the study identified the following benefits of working with social collaborators:   - increased awareness of mental health in the family and community. - reduced stigma; - a better understanding that depression is treatable. - increased help-seeking; and - improved access to care. |
| Chibanda et al. (2017) | Zimbabwe | Lay Health Workers’ Experience of Delivering a Problem Solving Therapy Intervention for Common Mental Disorders Among People Living with HIV: A Qualitative Study from Zimbabwe | The study sought to describe the LHWs’ experience of delivering the Friendship Bench intervention with the aim of using the information to inform the finalization of an intervention package for a planned cluster RCT. The authors also sought to establish the local indigenous terms that were being used by LHWs and by their clients to describe the different components of the Friendship Bench sessions and the number of sessions they were having with clients. |  |  |  | Locally Adapted Problem-Solving Therapy (PST):   - The PST was adapted based on local concepts from LHWs and clients. - It remains user-friendly while aligning with traditional PST objectives, like the Friendship Bench model. - The first session is crucial due to the high mobility of clients.   It's important for those delivering the intervention to reside locally for better accessibility and continuity |
| Davis et al. (2022) | South Africa | The study sought to describe the LHWs’ experience of delivering the Friendship Bench intervention with the aim of using the information to inform the finalization of an intervention package for a planned cluster RCT. The authors also sought to establish the local indigenous terms that were being used by LHWs and by their clients to describe the different components of the Friendship Bench sessions and the number of sessions they were having with clients. | This study aimed to conduct a process evaluation of a task-shared psychosocial intervention for perinatal depression in South Africa through a grounded theory analysis. | - Qualitative design - Interviews, counselling transcripts - Grounded theory | 6 | Female, (range of age: 28 – 46, Mean= 37.2) | Challenges to Intervention Effectiveness:   - Socio-economic difficulties faced by participants limited the effectiveness of the intervention. - Discrepancies between the counselling strategies used and the intervention manual were also a factor.   Reported Benefits:   - Participants reported improvements in mood and cognition. - These gains were attributed to therapeutic elements that aligned with the previously identified ‘common elements’ of therapy |
| Henrique et al.(2021) | Brazil | Acceptability and fidelity of a psychosocial intervention (PROACTIVE) for older adults with depression in a basic health unit in São Paulo Brazil - a qualitative study | to investigate: 1. Health workers’ fidelity to the intervention protocol; 2. Acceptability of the psychosocial intervention from the viewpoint of older adult participants; and 3. Perceptions of the psychosocial intervention by the health workers. | - Qualitative design - Interviews - Thematic analysis | 11 | Female, (range of age: 29 to 60 years, Mean: 38) | - The PROACTIVE intervention was feasible and well-received by both health workers and older adults. - Qualitative assessments indicated a need for improved training and supervision to ensure better adherence to the intervention protocol |
| Jacobs (2020)***** | South Africa | Master Thesis: The experiences of facility-based counsellors who provide a psychosocial intervention for mental illness in primary healthcare facilities in the Western Cape | the study aims to explore FBCs’ perceptions of (1) the training they received to deliver the MIND intervention, (2) difficulties in implementing the intervention within PHC services and (3) their perceptions of the supervision they received to support intervention delivery. | - Qualitative design - Interviews - Framework analysis | 18 | Female= 13 (72.2%)  Male= 5 (27.8),  (age range 28 – 55, Mean: 40) | Barriers to intervention delivery:   - Competing tasks, low pay, lack of space, and privacy concerns were significant barriers to implementation.   Facilitators:   - Seeing patient improvements and using the intervention to address personal issues facilitated implementation. - Regular supervision and debriefing sessions were helpful. - Role-playing enhanced skills, while audio-recorded feedback was feasible but faced logistical challenges. |
| Jacobs et al. (2021)***** | South Africa | Task Sharing or Task Dumping: Counsellors Experiences of Delivering a Psychosocial Intervention for Mental Health Problems in South Africa |  |  |  |  |  |
| Joag et al (2020) | India | Feasibility and acceptability of a novel community-based mental health intervention delivered by community volunteers in Maharashtra, India: the *Atmiyata programme* | To identify and understand the challenges of delivering a community-led mental health intervention and evaluate its feasibility and acceptability for larger-scale implementation in rural India. | - Mixed-methods study (qualitative component extracted) - Pre-post questionnaires, interviews - Logistic regression, thematic analysis | 16 | 37 males and 28 females, (range of age: 28 to 45 years) | Effectiveness of Community-Led Interventions:   - Community-led interventions with local volunteers can successfully facilitate access to social welfare benefits. - The intervention taps into community social capital to engage and empower members in addressing mental health issues |
| Kidia et al. (2024) | Zimbabwe | Rural Friendship Bench: A qualitative study in Zaka district, Zimbabwe | This study sought to understand the intervention's implementation in terms of acceptability, feasibility, and sustainability as well as local attitudes towards mental wellbeing in rural Zimbabwe. to understand attitudes about mental wellbeing in the community that may be relevant both to implementation and adaptation and for future work on mental health in rural parts of Zimbabwe. | - Qualitative design - Interviews - Thematic analysis | 12 | Male=1, Female=11, age was not specified | - Positive Feedback on the Friendship Bench intervention - Issues of accessibility were highlighted. - Feasibility: While acceptable, the intervention was not deemed feasible or sustainable in its current form. - Considering the sociocultural context is crucial when developing mental health interventions in rural areas |
| Munodawafa et al. (2017) ***** | South Africa | A process evaluation exploring the lay counsellor experience of delivering a task shared psycho-social intervention for perinatal depression in Khayelitsha, South Africa | This study conducted a process evaluation exploring the perceptions of counsellors who delivered a task shared psycho-social counselling intervention for perinatal depression in Khayelitsha, Cape Town together with independent fidelity ratings | - Qualitative design - Interviews - Thematic analysis | 6 | Female, (range of age 28 to 46 years, Mean: 37.2) | Facilitating Factors of treatment delivery:   - The intervention content was effective in delivery. - Continuous training and supervision supported successful implementation - Adherence to a structured counselling manual was key. - Conducting sessions in the local language, isiXhosa, was beneficial - Confident and motivated counsellors contributed to success. - Older age, commitment, and desire for help among participants facilitated intervention delivery.   Barriers to Delivery:   - poverty, crime, and lack of space for sessions hindered the intervention - Participants’ young age, the nature of their problems, and avoidance of counsellors posed challenges - There was significant variation in fidelity ratings and dropout rates among different counsellors |
| Nyatsanza et al. (2016)***** | South Africa | Filling the treatment gap - developing a task sharing counselling intervention for perinatal depression in Khayelitsha South Africa | to describe the development of a task sharing counselling intervention for perinatal depression |  | 4 |  | Acceptability and Feasibility of Task-Sharing Counselling   - The intervention was acceptable and feasible for depressed women - Female patients preferred female counsellors for the sessions. - Monthly sessions in clinics with experienced Xhosa-speaking counsellors from the community were preferred - The intervention needed to include psychoeducation on: cognitive and behavioural effects of depression, coping with interpersonal issues, managing financial stress |
| Munodawafa, (2018)***** | South Africa | Filling the Gap: Development and qualitative process evaluation of a task sharing psycho-social counselling intervention for perinatal depression in Khayelitsha, South Africa. Doctoral thesis | This study conducted a process evaluation exploring the perceptions of counsellors who delivered a task shared psycho-social counselling intervention for perinatal depression in Khayelitsha, Cape Town together with independent fidelity ratings |  | 6 |  | - Task‑shared individual counselling for perinatal depression, delivered by trained and supervised lay community health workers using a structured manual, is a feasible and acceptable strategy to reduce the treatment gap for depressed pregnant women in low‑resource settings like Khayelitsha. - The thesis argues that applying the MRC process‑evaluation framework (attention to context, implementation and mechanisms) is crucial for developing culturally relevant, scalable interventions; future policy and programmes should support scaling up such task‑shared models, with investment in training, supervision, fidelity assessment and adaptation of session dose and content to women’s needs |
| Musyimi et al. (2019) | Kenya | Integration of Traditional Birth Attendants into Mental Healthcare: A Multistakeholder Qualitative Study Exploration | to gain insight into the feasibility of integrating TBAs into maternal mental healthcare using multiple stakeholder views. | - Qualitative design - Interviews, FGDs - Thematic analysis | 88 | Female, (range of age: 36 to 65 years) | Key Themes from the Qualitative Data:   - Involving Traditional Birth Attendants (TBAs) in perinatal mental healthcare. - Leveraging Rapport and Counselling Experience: Utilising TBAs' existing rapport with the community and their counselling skills. - Recognition and Valuation of TBAs: Recognising and valuing the role of TBAs within the formal healthcare system. - Training and Collaboration: Providing training for TBAs and fostering collaboration between them and healthcare workers |
| Myers et al. (2019) | South Africa | Community health worker-delivered counselling for common mental disorders among chronic disease patients in South Africa: a feasibility study | This study examined the feasibility of integrating a dedicated and a designated approach to CHW-delivered counselling for CMDs into chronic disease care in PHC facilities in the Western Cape province of South Africa. Specific aims were to explore (1) the feasibility of recruiting and retaining patients with chronic disease for CHW-delivered mental health counselling and (2) dedicated and designated CHWs’ perceptions of the feasibility and acceptability of delivering mental health counselling to patients with chronic disease. Findings will inform patient recruitment and retention protocols and CHW training and intervention protocols that will be used in a future trial examining the relative effectiveness and cost-effectiveness of these two approaches to CHW-delivered counselling | - Mixed-methods study (qualitative component extracted) - Depression scale, interviews - Framework analysis | 7 | Female, age is not provided | Willingness to Receive Counselling:   - About 67% of eligible patients were willing to receive mental health counselling. - Patients with depression were more interested in counselling compared to those with hazardous alcohol use.   CHWs' Acceptance and Requests:   - Both dedicated and designated Community Health Workers (CHWs) found the counselling package highly acceptable. - CHWs requested additional training and support to improve the effectiveness of the intervention's implementation. |
| Ng'oma et al. (2024) | Malawi | Piloting a psychosocial intervention for perinatal depression, the Thinking Healthy Programme–Peer delivered (THPP), in a primary care setting in Lilongwe District, Malawi | to evaluate its feasibility and acceptability to perinatal women with depression in communities surrounding Kabudula Community Hospital in rural Lilongwe District, Central Malawi. | - Mixed-methods study (qualitative component extracted) - Depression scales, interviews - T-test, thematic analysis | 7 | Female, (range of age 32–51 years, Mean of age: 36) | Acceptability and Feasibility of THPP (Traditional Home-Based Postnatal Program) :   - Mothers and their families found the modified THPP to be both acceptable and practicable.   Implementation Challenges:   - Peer volunteers faced challenges due to inadequate incentives |
| Pacichana-Quinayáz et al. (2016) | Colombia | Common Elements Treatment Approach based on a Cognitive Behavioral Intervention: implementation in the Colombian Pacific | to describe the implementation of the Common Elements Treatment Approach (CETA) for Afro-Colombian victims of violence in Buenaventura and Quibdó, Colombia | - Qualitative case study - Interviews - Narrative content analysis | 5 | Not provided | Challenges to Implementation psychological intervention:   - The continuing conflict in the region hampers the implementation of the Common Elements Treatment Approach - Economic instability in the region further complicates the intervention’s delivery - The scarcity of mental health resources affects both the implementation and effectiveness of the intervention |
| Pereira et al. (2011) | India | The integration of the treatment for common mental disorders in primary care: experiences of health care providers in the MANAS trial in Goa, India | to describe the experiences of integrating the MANAS intervention in primary care. | - Qualitative design, - Interviews, - thematic analysis | 17 | Female,  Mean: 27 | Key Factors for Enhancing LHWs' Acceptability and Integration:   - Adequate training for LHWs is essential - Building trust with patients and primary care teams is crucial - Allowing sufficient time for LHWs to establish relationships and deliver care - Demonstrating positive patient outcomes helps with acceptance - Regular supervision and support from psychiatrists is vital. |
| Rodríguez-Cuevas et al. (2021) | Mexico | Living Six Hours Away from Mental Health Specialists  Enabling Access to Psychosocial Mental Health Services Through the Implementation of Problem Management Plus Delivered by Community Health Workers in Rural Chiapas, Mexico | to evaluate the effectiveness of integrating community mental health workers (CMHWs) into rural primary care settings to address the mental health needs of these underserved communities. Specifically, the study aimed to pilot a mental health intervention and learn from its implementation to potentially expand the project | - Qualitative design - A field report - Not provided | 4 | Female, (range of age: 18–50) | - The PM+ intervention positively impacted patients' symptoms and improved LHW attitudes toward mental health and themselves. - Challenges such as intimate partner violence, structural stress, and COVID-19-related grief need to be addressed to better serve underserved areas, despite previous adaptations to the intervention. |
| Saleem et al. (2021) | Pakistan | A mixed methods exploration of the role of multi-family groups in community treatment of patients with depression and anxiety in Pakistan | The aim of this study is to explore the feasibility, experiences, and outcomes of using multi-family groups in the community treatment of patients with depression and anxiety in Karachi, Pakistan1. The study seeks to determine if this low-intensity intervention, facilitated by non-specialist mental health service providers, can improve subjective quality of life, self-reported symptoms of depression and anxiety, and social outcomes for patients | - Mixed-methods study (qualitative component extracted) - Psychometric scales, interviews - T-Test, thematic analysis | 3 | Female, age was not provided | - Community treatment of common mental health disorders using multi-family groups led by non-specialists is feasible - The approach is well-received by participants. - Improved Quality of Life - Reduction in Depression and Anxiety - Enhanced Social Outcomes |
| Selohilwe et al. (2023) | South Africa | Challenges and opportunities for implementation and dissemination of a task sharing counselling intervention for depression at primary health care level in South Africa | to understand multilevel factors contributing to implementation and potential dissemination of such a service for depression at PHC level | - Qualitative design - Interviews - Framework analysis | 19 | Not provided | Facilitators of the intervention:   - Supervision and support for counsellors. - Person-focused counselling approach. - Integration of counsellors within healthcare facilities.   Barriers:   - Insufficient organizational support. - Lack of dedicated space for counselling. - High counsellor turnover. - Absence of a designated role for the intervention within the system. - Exclusion of mental health treatment, including counselling, from mental health indicators |
| Singla et al.(2020) | India | Peer supervision for assuring the quality of non-specialist provider delivered psychological intervention | i) to estimate the reliability and predictive validity of a therapy quality measure for use by peers; ii) to assess the extent to which peer delivery agents could be trained to evaluate their peers’ counsellors as reliably as experts; and iii) to identify barriers and facilitators of several implemented models of peer supervision. | - Mixed-methods study (qualitative component extracted) - Psychological scales, interviews - Paired t-Test, framework analysis | 26 | Female, (range of age 27–50 years, Mean: 37.85) | - Non-specialist providers can be trained to conduct peer supervision and reliably assess therapy quality using valid measures. - Peer supervision involving experts was preferred for providing structured and reliable feedback. |
| Szajna et al. (2024) | India | Feasibility and acceptability of a community health worker administered behavioral activation intervention for postpartum depression: a single arm pilot study from India | to evaluate the feasibility and acceptability of community health workers (ASHAs) screening for and delivering a brief behavioral activation (BA) intervention for postpartum depression (PPD) among women in Belagavi, South India, and to assess the impact of this intervention on PPD symptoms. | - Mixed-methods study (qualitative component extracted) - EPDS scale and Interviews - the pre-test/post-test analysis and thematic analysis | 17 | Female, age was not provided | Feasibility and Acceptability of Behavioural Activation (BA)   - High intervention uptake and retention - Positive reception from participants - Community Health Worker (CHW) delivery was well-received - Improvements in depressive symptoms - Enhanced daily activity and engagement - Improvements in depressive symptoms   Challenges and Considerations   - Effective delivery depended on adequate CHW training and supervision - Balancing the intervention with other CHW responsibilities posed potential strain. - Adapting content to the local context and aligning with postpartum norms helped improve relevance and uptake |
| Wood et al. (2021) | India | “I will not step back”: A Qualitative Study of Lay Mental Health Workers’ Experiences in India | to understand the barriers and facilitators that lay health workers (LHWs) face in delivering mental healthcare | - Qualitative design - Interviews - Thematic analysis | 32 | Men (n=8) and women (n=24), Mean: 37-year-old | Barriers and Facilitators of intervention delivery by LHWs   - Factors include personal traits, family support, daily work relationships, and supervision. - Compensation issues were highlighted as a key factor. - Gender discrimination and stigma were identified as barriers |
| Yator et al. (2022) | Kenya | Acceptability and impact of group interpersonal therapy (IPT-G) on Kenyan adolescent mothers living with human immunodeficiency virus (HIV) a qualitative analysis | we seek to assess the acceptability and feasibility of Group Interpersonal Psychotherapy (IPT-G) delivered by CHWs among depressed postpartum adolescents (PPAs) living with human immunodeficiency virus (HIV). | - Qualitative design - Interviews - Thematic analysis | 8 | Female, Mean of age: 37 | - LHWs valued the intervention for improving their knowledge and skills. - Patients reported better interpersonal relationships, reduced distress, and less HIV-related stigma. - Primary healthcare workers successfully integrated the intervention sessions into their routine clinic activities |

**Supplementary Table 2. Characteristics, Roles, and Support Arrangements of Lay Health Workers in Included Studies**

| **Authors (Year)** | **Term used for LHW** | **Gender** | **Age (Mean)** | **Background and Roles** | **Education** | **Remuneration/Incentives/Voluntary** | **Supervision** |
| --- | --- | --- | --- | --- | --- | --- | --- |
|  |  |  |  |  |  |  |  |
| Abas et al. (2016) | *Ambuya utano* (grandmother health workers or Lay Health Workers) | women | The mean age of the LHWs is 62 years. | - Delivering mental health promotion talks addressing stress, depression, and local idioms of emotional distress. - Generating referrals through multiple pathways, including primary care, community sources, and self-referral. | primary school education | - Receiving a small monthly allowance of US$6–10 | LHWs received weekly supervision, which was crucial for maintaining the quality of care. |
| Ahmed et al. (2024) | Near-peer counsellor | Mixed gender men and women | The counsellors were youth aged 21–28 years (median 24) | - Functioning as near-peer providers of PST for adolescents living with HIV. - Delivering the adapted Friendship Bench intervention | NA | - Receiving financial compensation - Experiencing insufficient pay - Lacking transport allowance - Seeking formal employment recognition | - Receiving direct supervision from a clinical team of physicians and mental health professionals. - post-counselling debrief sessions after delivering Safe Haven counselling - support group sessions with other lay counsellors |
| Atif et al. (2016) | Peer Volunteer | Women | Mean = 33 years (range: 26–40 years) | - Peers are local women from the community with shared socio-demographic characteristics and life experiences similar to the target population | Mean = 11 years (range: 10–14 years) |  | PVs were supervised by the THPP facilitators through monthly group supervisions and field  supervisions. THPP facilitators were supervised fortnightly |
| Atif (2015) |  |  |  |  | Pakistan: Minimum 10 years of schooling (equivalent to GCSE and A-level)  India: Minimum 7 years of schooling (equivalent to 7th grade). | Pakistan: Peers were paid only the sustenance allowance for travel to trainings and supervision.  India: Peers were paid a fixed amount for every session they successfully delivered. | PVs were supervised by the THPP facilitators through monthly group supervisions and field  supervisions.THPP facilitators were supervised fortnightly |
| Atif et al. (2017) |  |  |  |  |  |  | - Pakistan: Peers were supervised by the THPP trainers through regular monthly group and field supervisions. - India: Peers were supervised by THPP trainers through fortnightly group supervision and two individual supervision sessions during internship. Supervision supported experiential learning, discussion of work-related stress, and maintenance of motivation. |
| Bah et al. (2025) | nonspecialists, mother-to-mother support groups (MMSGs) | Women | 25–50 years | MMSGs are described as unpaid lay women working at community level to facilitate early antenatal contacts and support mothers | NA | Serving as unpaid volunteers, with no salary provided for their MMSG role. | - Supervision was conducted biweekly through group supervision sessions. - A multi-tiered supervision team provided clinical, logistical, monitoring, and fidelity support. - The nurse psychosocial lead provided clinical oversight and mentoring. - Research assistants provided logistical and monitoring support. - The project lead ensured fidelity to the intervention model. |
| Burgess et al. (2025) | Health Extension Program | Women | The study only specifies that health extension workers (HEWs) were aged 18 years or older | - Staffing rural health posts as Health Extension Workers. - Providing prevention-based care, including hygiene, sanitation, disease prevention and control. - Offering counselling on breastfeeding, infant health, vaccines, and postpartum family planning | NA | - Working as salaried government employees under Ethiopia’s Federal Ministry of Health. - Receiving fixed monthly salaries as permanent public health workers. | - Receiving supervision from the research and clinical team, including trained psychologists and study coordinators - Participating in ongoing coaching and support, rather than supervision limited to monitoring - Receiving guidance on reflective listening as part of supervision - Receiving support to adapt CBT components to women’s cultural and literacy levels |
| Chau et al. (2024) | Social Collaborator | Mixed gender men and women with 78.7% were women and 21.3% were men | 48.9% of the providers (social workers and social collaborators combined) were over 55 years of age | - Recruited from existing community and social service roles - Social collaborators were often already active as village health workers or community representatives. - They provided support beyond formal mental health tasks, including general advice, encouragement around routines/exercise, and informal advocacy. - Functioning as locally embedded community providers with existing knowledge of community members and social networks. | Some social collaborators themselves have limited formal education, with some only completing education up to the ninth grade in remote areas | - Receiving no regular salary - Receiving no compensation for additional time spent on intervention-related activities. - Receiving no transport allowance for home visits - Absorbing personal costs associated with travel and extended community-based support. | - Supervision frequency was not specified, but oversight appeared continuous or periodic throughout the intervention. - Supervision and guidance were embedded within MOLISA’s structure. - Social collaborators reported continued connection and guidance from social workers even after the main RCT ended. - Continued supervision appeared accessible and valued by providers. |
| Chau et al. (2021) |  | Mixed gender (78.7% were women) | 55 (48.97% were over 55) |  |  |  |  |
| Chibanda (2015) | Lay Health Worker or *ambuya utano* (grandmother health provider) | Women | 58 (SD: 8.3) | - Being employed by city health services, rather than working as informal volunteers. - Their main role was community health outreach, including supporting people living with HIV/AIDS and tuberculosis. - They provided practical, psychological, spiritual, individual, and family support, and encouraged medication adherence. | 8–10 years of education | employed by the city health services, receiving transport money | A clinic staff nurse trained in counselling provided weekly group supervision at the largest clinic. The clinical psychologist and the psychiatrist provided further supervision every fortnightly and monthly, respectively. |
| Chibanda et al. (2017) |  |  |  |  |  |  |  |
| Davis et al. (2022 | community health workers (CHWs) | Women | Mean= 37.2 (28 - 46) | provide informational health visits to pregnant women and mothers of young babies in their communities. 2 - 11 years providing health visits to pregnant women before AFFIRM-SA training | grade 9 - grade 12 school level completed | - CHWs were paid employees within the short-term project and were not volunteers. - Their salaries were covered by the project’s human-resource budget. - They were specifically recruited and trained for the study. - Their project-employed status differentiated them from government CHWs who were unpaid or stipend-based. | - CHWs received weekly group-based specialist mental health supervision from a specialist mental health counsellor/social worker. - Supervision included case reviews, discussion of difficult cases, troubleshooting, and support to work with general health providers. - CHWs could consult the specialist counsellor for emergencies, including suicidal behaviour. - Monthly individual supervision was provided to discuss work progress. |
| Henrique et al.(2021) | Nurse Assistants (NAs) and community health workers (CHWs) | Women | Age range: 29–60 years.  6/11 participants aged 29–34 years.  3/11 participants aged 35–45 years.  2/11 participants aged 50–60 years. | - NAs and CHWs had different routine roles within the UBS. - NAs assisted nurses with health procedures and vaccination. - CHWs visited households to collect family health information and report it to the Family Health Team. - Despite different routine duties, NAs and CHWs performed the same role in the psychosocial intervention. | - NAs had at least secondary education and additional technical training in assisting nursing. - CHWs had at least secondary education but no formal health education or training. - CHWs lived in the UBS catchment area, indicating local community embeddedness. | - The article does not explicitly report salary or direct financial support for nurse assistants or CHWs. - NAs and CHWs were already employed staff within Brazil’s Family Health Strategy, part of the Unified Health System.   As regular public health staff, they can be inferred to be salaried employees rather than volunteers. | - Weekly group supervision was held at the UBS and conducted by a psychologist. - Supervision covered case discussion, difficulties following the intervention protocol, and use of the app. - Supervision also functioned as ongoing training. - Between sessions, CHWs could contact the clinical supervisor through a WhatsApp support group for clinical doubts or technological support. |
| Jacobs et al. (2021) | facility-based counsellors (FBCs) | Women= 13 (72.2%) Men= 5 (27.8) | an average age of 40 years (SD = 9.1), age range 28 - 55 | the majority of designated FBCs started  working as either home-based carers or community care workers before progressing to work as HIV  FBCs in PHC facilities. Dedicated FBCs had similar work experience but were unemployed at the  point of recruitment from the community. | All the FBCs had completed high school (12 years of education) and training in HIV adherence counselling. 4 FBSs had completed a four year university degree | he Facility-Based Counsellors (FBCs) were paid employees, but they complained about low salaries and lack of financial recognition for their expanding workload. | - FBCs received regular supervision, debriefing, and in-service training from a registered psychological counsellor experienced in MI-PST. - Supervision was consistent across sites and included debriefing, discussion of patient progress, challenges, and feedback on counselling sessions. - Audio-recorded counselling sessions were reviewed by the counselling supervisor using a fidelity checklist. - The fidelity checklist assessed whether the intervention was delivered as intended. |
| Jacobs (2020) |  |  |  |  |  |  |  |
| Joag et al (2020) | Champions | 37 men and 28 women. | between 28 and 45 years |  | - Non-literate: 29% 1st to 4th grade: 18% 5th to 10th grade: 43% Beyond 11th grade: 10%. - The average Champion had completed at least primary school. | - Atmiyata Champions worked voluntarily and received no salary or direct financial support. - They received non-financial incentives, including mobile phones, intervention films, and training certificates. - Training certificates could be added to their credentials for future work opportunities. - They also gained community recognition and pride from their role. | - Supervision followed a tiered structure: project lead/project manager supported CFs, and CFs supervised Champions. - CFs met Champions twice a month for supervision and mentoring. - The project lead met CFs and the project manager monthly to oversee implementation and provide mentoring. - During monitoring visits, CFs discussed counselling sessions with Champions and, with consent, sometimes met beneficiaries to understand the counselling process |
| Kidia et al. (2024) | village health workers | Men=1, Women=11 | not clear | NA | NA | Voluntary basis but received USD $10 per month for transport and cell phone credit | - Each rural health centre had a nurse supervisor and a mental health nurse to oversee the VHWs. - The supervision included ensuring proper implementation of problem-solving therapy, supporting VHWs, and handling referrals for more complex cases |
| Munodawafa et al. 2017, Munodawafa, 2018 | Community health workers | Women | 28 to 46 years, with a mean age of 37.2 years | - CHWs were selected from a local NGO.   They had at least two and a half years of community health promotion experience.   - Their routine work involved household health promotion visits with mothers and children under five. - They identified underweight children and children at risk of malnutrition. - They educated mothers on improving their own wellbeing and their children’s health | Their education ranged from grade 9 to grade 12. | - Receiving transport money, - a monthly stipend, and transport to inaccessible or dangerous areas | - CHWs were trained, supervised, and supported by a mental health counsellor with a master’s degree in Clinical Social Work. - Additional support was provided twice a month by a senior clinical psychologist/principal investigator. - Supervision included two to three hours of weekly group supervision and ongoing training. - CHWs also received 30 minutes of individual supervision each month. |
| Musyimi et al. (2019) | traditional birth attendants (TBAs), community health volunteers (CHVs) | Women | 36 to 65 years old. | They are informal health providers, often elderly women, who are respected in their communities. They provide various services including counselling, massaging, emotional support, and assistance during childbirth, particularly in rural settings with limited access to formal healthcare facilities | TBAs are often older, respected women who are part of the local community but do not have a formal education and are often illiterate | no indication that TBAs received any formal financial remuneration or regular funding from the government or health system. | - TBAs were not well supervised and had no formal supervisory system. - They lacked clear job descriptions and structured division of responsibilities with health workers. - Their only regular contact was monthly meetings with the public health officer. - These meetings appeared administrative or coordination-focused rather than clinical supervision. - No established supervision method or schedule was reported. |
| Myers et al. (2019) | community health workers (CHWs) | Women | NA | to deliver HIV adherence counselling, expanded to include mental health counselling. | completion of high school | - Designated CHWs were already salaried through NGO employers, and their job descriptions were expanded to include mental health counselling. - Dedicated CHWs were newly hired and appointed specifically to deliver counselling only. - Both designated and dedicated CHWs were paid. - The two groups were matched on remuneration. | - A psychological counsellor provided weekly individual supervision to both dedicated and designated CHWs. - Supervision included feedback on counselling sessions and guidance to improve fidelity and quality. - Retraining in aspects of the programme was provided when needed. - Debriefing was provided for difficult or challenging cases. |
| Ng'oma et al. (2024) | peer volunteers | Women | 36 years, (Range 32–51 years) | agriculture sector (n=1) and health sector (n=4), none (n=1) | - Volunteers had a minimum of eight years of education, equivalent to Standard 8. - Some held a Primary School Leaving Certificate, indicating completion of primary education. - Some held a Junior Certificate, indicating completion of eight years of primary education and two years of secondary education. - All volunteers were able to read and write. | - Peer volunteers received monetary compensation per session. - They were dissatisfied with the amount of compensation. - They expressed a preference for formal remuneration. - Lack of adequate financial incentives was identified as a common barrier in community-delivered interventions. | - Peer volunteers received fortnightly supportive supervision from the PI, research assistants, and environmental health staff. - Individual intervention sessions were reviewed during supervision. |
|  |  |  |  |  |  |  |  |
| Nyatsanza et al. (2016) | Community Health Workers | Women | not clear | not clear | not clear | - CHWs were recruited from and employed by a local NGO. - They worked within the formally funded AFFIRM-SA project. - They were paid employees rather than volunteers. - Salary amounts were not specified in the paper. - Payment was likely provided under the short-term project funding structure. | - CHW counsellors were trained, supervised, and supported by a mental health counsellor/clinical social worker. - Supervision was provided weekly in groups and biweekly individually. - Supervision monitored session progress, detected adverse events, discussed difficult cases, and provided counselling guidance. - Counsellors could access counselling on request from an external organisation for emotional support and burnout prevention. |
| Pacichana-Quinayáz et al. (2016) | Lay Psychosocial Community Workers (LPCWs) |  | NA | NA | NA | The paper directly reports that being an LPCW provided “generation of income and relative economic stability”, confirming that they were financially compensated | LPCWs were supervised by mental health professionals (psychologists and social workers) |
| Paudel et al. (2014) | Village health worker (VHW) | NA | NA | - The majority (57.7 %) of VHWs had work experience of more than 10 years, with 7 out of 26 (26.9 %) having more than 20 years experience with CRHP. - The more VHWs had field experience, the less they had formal education. | half of the VHWs had received formal education. Out of 26 VHWs, 11 (42.3 %) had no formal education at all. no VHWs with education of more than 10 years | NA | - VHWs usually attended monthly meetings at the main centre, which appeared to serve as routine supervision/contact with CRHP staff. - Supervision was embedded within CRHP’s existing community health structure. - CRHP programme staff and mobile health team members, including nurses, social workers, or trainers, acted as supervisors/facilitators. - No new or mental health-specific supervision framework was reported. |
| Pereira et al. (2011) | the Lay Health Counsellor (LHC) | women | 27 | The LHC acted as a case manager for all patients who screened positive for CMD and took overall responsibility for the intervention delivery, working in close collaboration with the primary care physician and a visiting psychiatrist | a female college graduate | - LHCs in the MANAS study were paid project staff, not unpaid volunteers. - They were employed and remunerated under project funding. - Recruitment and full-time placement imply formal employment rather than voluntary work. - They received a monthly salary/stipend, but the exact amount was not reported. | - Supervision was provided by a Clinical Specialist/visiting psychiatrist and through peer-group supervision. - The Clinical Specialist provided ongoing remote support and periodic in-person supervision visits; exact visit frequency was not specified. - Monthly peer-group supervision was held among LHCs, led by project staff and supported by the Clinical Specialist. |
| Rodríguez-Cuevas et al. (2021) | community mental health workers (CMHWs) | women | 18–50 years of age | CMHWs act as front‑line, non‑specialist providers who deliver, adapt and help sustain the PM+ mental health intervention in rural Chiapas | from middle to high school | - CMHWs held paid positions that contributed to household income. - They were employed by Compañeros En Salud, the Mexican branch of Partners In Health. - CMHWs were therefore paid workers within an NGO-supported health service structure, not unpaid volunteers. | - Received monthly in-person/field supervision and weekly tele supervision. - Field supervision involved monthly 2–3 day visits to each community. - Supervision included case consultations, live observation of PM+ sessions, and role plays. - Supervisors addressed countertransference, coping with mixed feelings, self-care, and self-disclosure. - Written case notes and assessments were evaluated during supervision. - Supervision also included follow-up meetings with the community physician. |
| Saleem et al. (2021) | Lay counsellors | women | NA | NA | NA | Lay counsellors were paid under the project implementation, supported under a UK NIHR-funded project | - the lay counsellors were supervised, but the supervision was limited to study implementation oversight rather than continuous clinical supervision. - Supervision and oversight were provided by the study investigators and project supervisors. |
| Selohilwe et al. (2023) | lay-counsellors | not clear | not clear | not clear | not clear | - Lay counsellors were salaried, project-employed staff under the PRIME-SA programme. - They were formally hired and paid rather than working as volunteers. - Their financial support depended on project funding. - District managers noted the absence of a dedicated mental health budget to sustain services beyond the project period. | - Received regular supervision and mentoring for continuous support and development. - After training, they received onsite in vivo supervision through an apprenticeship model across all eight sessions until competency was achieved. - Replacement counsellors received structured peer-to-peer learning with experienced counsellors before independent facility placement. - All lay counsellors received weekly offsite group supervision and could contact supervisors by phone when needed. - Supervision was provided by registered psychological counsellors and an intern clinical psychologist. |
| Singla et al.(2020) | *Sakhis* (meaning ‘friend’) were mothers living in the same or neighbouring community as mothers participating in the intervention | Women | 37.85 years (range 27–50 years), and they had about two children (range 1–3) | Peers were mothers belonging to the same or neighbouring communities as the trial participants with depression. These were women with children who had, a similar socio-demographic background as participants, and good communication skills. they had about two children (range 1–3) | mean education was 11.85 years (range from 9 to 15 years) | - the Sakhis did not receive a salary and worked without financial incentives. - most of both Sakhis and supervisors reported difficulties with travel due to a lack of compensation and a lack of time to attend meetings due to other commitments | - Monthly group supervision was encouraged in small groups of 2–4 peers. - Two forms were used: expert-facilitated peer supervision and peer-only “mini meetings. - In expert-facilitated supervision, one audio-recorded session was independently rated by the provider, peers, and expert. - Up to three-fourths of treatment sessions were audio-recorded for quality monitoring. - Audio recordings were randomly selected and stratified by audio quality, phase, and delivery agent. - In peer-only meetings, Sakhis met monthly in groups of 2–4 to discuss treatment cases. |
| Szajna et al. (2024) | Accredited Social Health Activists (ASHAs) | Women | NA | - ASHAs routinely visited women during prenatal and postnatal periods as part of usual care. - Their existing role provided a platform for integrating maternal mental health activities. - Their role was expanded to screen for postpartum depression using the EPDS. - They delivered a five-week home-based behavioural activation intervention to postpartum women who screened positive. | It only notes that all training materials were designed to match the existing education and literacy levels of the ASHAs, implying variable but generally limited formal education | - ASHAs/LHWs received financial compensation as part of the project. - The article does not specify the amount or structure of intervention-related compensation. - No complaints about salary or compensation were reported. - Paid status was confirmed, but remuneration details were limited. | - ASHAs received continuous and responsive supervision from a multidisciplinary research team. - Supervisors included a psychiatrist, community and psychiatric nurse researchers, and public health/midwifery faculty from US and India-based academic institutions. - Supervisors checked in randomly during sessions and provided on-demand support for risk-related cases. - ASHAs participated in post-intervention focus group interviews two weeks after completion to reflect on their experience and provide feedback. |
| Wood et al. (2021) | lay health workers (LHWs) | Men (n=8) and women (n=24) | 37 | 4.6 years of work experiences as LHWs | Middle or high school (n=12) Higher secondary (n=7) Graduation (n=7) Post graduation and above (n=6) | - NGO-provided resources, including salary, benefits, and materials, varied widely across programmes. - LHWs commonly requested higher salaries, travel allowances, lunch stipends, and health insurance. - Lack of financial stability was a recurring concern. - LHWs expressed anxiety about job insecurity, particularly where employment depended on short-term grant funding. | - LHWs were regularly supervised in most programmes, although supervision quality varied across NGOs. - Supervision was frequent, supportive, non-hierarchical, and encouraging. - Supervisors included programme staff and NGO-based mental health professionals, such as psychologists, social workers, or project coordinators. - Supervision included feedback, case discussion, and problem-solving. - LHWs spent an average of 3.7 hours per week receiving supervision, indicating weekly contact in most programmes. |
| Yator et al. (2022) | community health workers (CHWs) | Women | 37 | - CHWs were lay/non-medical providers in Kenya, comprising Community Health Assistants (CHAs) and Community Health Volunteers (CHVs). - CHWs were based in or linked to routine primary health care settings in Nairobi, specifically Kangemi and Kariobangi health centres. - CHWs acted as a link between the health facility and the community, including following up participants in their homes or community settings. | post-secondary school (n = 4), grade 12 (n = 1), grade 10 (n = 1), and below grade 8 (n = 2) | poor remuneration or complete lack of reward | - CHWs were closely and continuously supervised by the lead researcher, clinical supervisor, and research assistant. - The clinical supervisor was a final-year MSc Clinical Psychology student, while the research assistant supported recruitment and session logistics. - Weekly field notes were kept and reviewed. - All IPT-G sessions were audio-recorded for auditing and supervision. - Weekly supervision was conducted face-to-face, with continuous digital support via WhatsApp, phone, and SMS between sessions. - Audio review and feedback continued throughout the eight-week IPT-G intervention. |

**Supplementary Table 3. Characteristics of Interventions**

| **Authors (Year)** | **Name of Programs** | **Interventions** | **Number of sessions** | **Duration per session** |
| --- | --- | --- | --- | --- |
|  |  |  |  |  |
| Abas et al. (2016) | Friendship Bench | Problem Solving Therapy (PST) with components of behaviour activation | 6 sessions | Not clear, but LHWs emphasised that they spent a long time in the first session with the client, generating solutions as well as identifying problems ‘in case the client does not come back’. |
| Ahmed et al. (2024) | Safe Heaven | Problem solving therapy, behavioural activation, psychoeducation | Six problem‑solving therapy sessions delivered individually to adolescents living with HIV by near‑peer lay counsellors. | NA |
| Atif et al. (2016) | the Thinking Healthy Programme peer delivered (THPP) | CBT based intervention, employing the core principles and techniques of cognitive behaviour therapy (CBT), such as building an empathetic relationship, focusing on the here and now, behaviour activation and problem solving | 10 sessions over a period of four months (7 individual sessions delivered at home and 4 group sessions delivered at the Health House) | NA |
| Atif (2015) |  |  | Pakistan: Peer-delivered 10 home based individual sessions & 4 group sessions. India: Peer-delivered 6 to14 home based individual sessions. | NA |
| Atif et al. (2017) |  |  |  | NA |
| Bah et al. (2025) | riendship Bench Intervention (PST-FBI) delivered by nonspecialists, mother-to-mother support groups | Problem-Solving Therapy | four sessions | 60–90 minutes |
| Burgess et al. (2025) | the Mothers Time intervention | simplified Cognitive Behavioral Therapy (CBT)-based program combined with family planning counselling, specifically designed for postpartum women experiencing mild to moderate symptoms of depression or anxiety and who have an unmet need for family planning. | Three sessions | NA |
| Chau et al. (2024) | supported self-management (SSM) intervention | Structured sessions: Social collaborators (such as Community Health Workers) guide clients through a series of sessions focused on managing depressive symptoms. Behavioral activation and problem-solving: They teach practical strategies to help clients engage in meaningful activities and cope with challenges. Psychoeducation: They provide information about depression, its symptoms, and ways to manage it. Monitoring and support: They check progress, encourage adherence, and offer emotional support throughout the program. Linkage to care: If needed, they connect clients to additional health or social services. | Duration of the intervention The supported self‑management intervention was delivered over a two‑month period for each client. Number and spacing of sessions Coaching sessions were scheduled every two weeks, during which the social collaborator discussed progress, reviewed workbook concepts, and helped plan the next two weeks. Over a two‑month period with sessions every two weeks, this corresponds to approximately four coaching sessions per client (assuming clients remained engaged across the full intervention period). | NA |
| Chau et al. (2021) | The Mental Health in Adults and Children—Frugal Innovations (MAC-FI) study. | Supported self-management based on CBT | every two weeks in two months | NA |
| Chibanda (2015) | The Friendship Bench | Problem Solving Therapy (PST) with components of behavior activation | The 6 sessions were completed within a 4–6 week period, including one or two home  visits. Patients also received up to 6 brief text messages and/or calls reinforcing the PST approach and encouraging adherence to treatment | 30 - 45 minutes per session |
| Chibanda et al. (2017) |  |  |  |  |
| Davis et al. (2022) | Africa Focus on Intervention Research for Mental Health - South Africa (AFFIRM-SA) | psycho-education, PST, behavioral activation, cognitive reframing (healthy thinking), and relaxation training | a six-session task-shared manualised counselling intervention | approximately 1 hour in duration each |
| Henrique et al.(2021) | a new psychosocial intervention (PROACTIVE) | a standardised collaborative care psychosocial intervention | a 17-week home-based psychosocial intervention. The low intensity regimen has five additional sessions, being three sessions every other week and the last two, monthly. The high intensity regimen has eight additional sessions, being six sessions weekly and the last two, monthly | 50 min |
| Jacobs et al. (2021) | the project MIND counselling intervention | a three-session blended Motivational Interviewing and Problem-solving Therapy (MI-PST) , psychoeducation on depression and alcohol, how to cope with negative tought | 3 sessions of blended motivational interviewing/problem-solving therapy (MI-PST), with optional booster, to be delivered weekly. Participants had a 6-week window to complete all sessions before timing out of the intervention | Each counselling session is generally between 40 to 60 minutes in length |
| Jacobs (2020) |  |  |  |  |
| Joag et al (2020) | the Atmiyata intervention. an integrated care approach addressing both social and health care needs and consists of the following sub-interventions | Psychosocial intervention (social and mental health problems) | Each Champion delivers 4 to 6 counseling sessions to person with the distress and common mental health issues using basic counseling techniques of active listening, behavioural activation and problem solving. |  |
| Kidia et al. (2024) | Friendship Bench | problem solving therapy and includes an income generating component | six sessions |  |
| Munodawafa et al. (2017); Munodawafa (2018) | the AFFIRM-SA RCT ( the AFrica Focus on Intervention Research for Mental health, South Africa) | psycho-education, PST, behavioral activation, cognitive reframing (healthy thinking), and relaxation training | six to eight sessions, additional session if needed over a period of 3 to 4 months. The counseling sessions are conducted approximately every 2 weeks at the clinic or at the participant’s home (by prior arrangement) | approximately 1 hour per session |
| Musyimi et al. (2019) | NA | Psychological counselling, emotional support, psychosocial intervention |  |  |
| Myers et al. (2019) | Community health worker-delivered counselling for common mental disorders | motivational interviewing (MI) and problem-solving therapy (PST) | three to four sessions in six weeks | NA |
| Ng'oma et al. (2024) | Thinking Healthy Programme–Peer delivered (THPP), CBT based PST | psychoeducation, facilitating family support, behavioral activation and problem solving | eight to ten individual home-based sessions and two group sessions | NA |
| Nyatsanza et al. (2016) | the AFrica Focus on Intervention Research for Mental health (AFFIRM-SA) | CBT based intervention | six structured and manualised counselling sessions over a period of 3–4 months. | NA |
| Pacichana-Quinayáz et al. (2016) | ACOPLE | Common Elements Treatment Approach (CETA), based on cognitive-behavioral therapies, and the other was the Narrative Community-based Group Therapy (NCGT), based on narrative therapies | NA | NA |
| Paudel et al. (2014) | Comprehensive rural health project (CRHP) has been working in rural India since 1970 to address common health conditions of poor and marginalized people | counseling, social support, and referrals to hospitals. They also promote awareness campaigns in the community. | NA | NA |
| Pereira et al. (2011) | The MANAS intervention. MANAS means “humanity” in the local Konkani language. It is also an acronym for MANAShanty Sudhar shodh (“project to promote mental health”). | a range of psychosocial treatments, such as psychoeducation, antidepressants, and interpersonal therapy | six sessions (in average) to eight sessions | 30 minutes to an hour |
| Rodríguez-Cuevas et al. (2021) | Problem Management Plus | cognitive behavioural therapy basic skills | weekly sessions (mainly through home visits) with patients, on an individual basis. Any patient should complete the programme within 5 weeks, with the opportunity to have additional sessions if they require them or if the CMHW and the CMP consider it appropriate (which they discuss during clinical supervision). Nevertheless, because of harvesting seasons, as well as other life circumstances that force patients to be far from their homes, the intervention can take several months | 2 - 3 hours per session |
| Saleem et al. (2021) |  | psychoeducation and various methods of family therapy. monthly multi-family group meetings. Family’ here is defined as anyone who is close with the patient and provides support | The meetings were held once per month over 6 months at local community centres and were chaired by trained counsellors. Each group consisted of five or six patients, one or two family members per patient, and the counsellor. Six multi-family groups were set up for the 6-month period, with each facilitator running two groups. > 6 sessions in average |  |
| Selohilwe et al. (2023) | The Programme for Improving Mental health carE [18] in South Africa (PRIME-SA) | brief counselling intervention informed by adapted cognitive behavioural interventions, including problem solving, cognitive restructuring and behavioural activation | Nine sessions | 45 min to an hour each and the lay-counsellor provided telephonic reminders of the appointment before each session |
| Singla et al.(2020) | Thinking Healthy Program Peer-delivered (THPP) | behavioural activation, identifying, monitoring and replacing unhealthy behaviours with healthy ones and practicing them) as well as general (active listening, collaboration with the family, guided discovery using pictures and stories, homework | 14 sessions over the perinatal period (antenatal and postnatal) | each lasting up to 45 min |
| Szajna et al. (2024) | ASHA-delivered BA intervention | brief Behavioral Activation (BA) program for postpartum depression (PPD). | 5‑week home‑based behavioral activation (BA) programme, five weekly home visits | NA |
| Wood et al. (2021) | NA | basic counselling skills such as active listening and building a therapeutic relationship, and additional evidence-based practices for specific disorders, such as behavioural activation for depression | NA | NA |
| Yator et al. (2022) | Group Interpersonal Psychotherapy (IPT-G) | group interpersonal therapy (IPT-G) | weekly for eight sessions | 90 minutes |

**Supplementary Table 4. Patient Characteristics, Eligibility Criteria, and Mental Health Contexts in Included Studies**

| **Authors (Year)** | **Mental Health Problems** | **Inclusion Criteria** | | **Exclusion Criteria** | **Gender** | **Age (Mean)** | **Context of MH Problems** |
| --- | --- | --- | --- | --- | --- | --- | --- |
|  |  | **Diagnosis** | **Other criteria** |  |  |  |  |
| Abas et al. (2016) | CMDs, particularly depression and other related disorders | NA | NA | NA | not clear | not clear | many patients face a host of desperate problems, such as poverty, HIV/AIDS and domestic violence |
| Ahmed et al. (2024) | depression and anxiety | The study involved adolescents living with HIV (ALHIV) who were screened for depression and anxiety during routine HIV care visits at BBCCCOE. Adolescents aged 13–24 years living with HIV | All participants were existing paediatric or young adult patients at the clinic (0–26 years overall clinic population), but only those meeting the above age and symptom criteria were recruited into Safe Haven | NA | Mixed gender men and women | The intervention targeted adolescents aged 12–25 in principle, but the clients who actually participated in the pilot and are described in this paper were 13–24 years old | HIV |
| Atif et al. (2016) | Perinatal depression | have a score of 10 or more on the PHQ-9. | the criteria were to be of child-bearing age (between 18-45), be in their third trimester of pregnancy or have a child less than 3 months old, planned to reside in the study area for one year | NA | women | Mean = 28 years (range: 21–45 years) | Majority of families in the study area live in a joint family system (consisting of multiple generations; parents, their children, and the children’s spouses and offspring, living in the same house), and women are generally economically and socially dependent on significant members of their families (husband, mother-in-law, own parents), patriarchal society |
| Atif (2015) | Perinatal depression | have a score of 10 or more on the PHQ-9. | the criteria were to be of child-bearing age (between 18-45), be in their third trimester of pregnancy or have a child less than 3 months old, planned to reside in the study area for one year | NA | women | Mean = 28 years (range: 21–45 years) |  |
| Atif et al. (2017) | perinatal depression | screened ≥ 10 on the PHQ-9 for depression using the Patient Health Questionnaire (PHQ-9). | 18 years or older, in their second or third trimester of pregnancy or had infants up to four months of age | NA | women | Mean = 28 years (range: 21–45 years) |  |
| Bah et al. (2025) | perinatal depression | Pregnant women between 12 and 34 weeks’ gestation. New mothers between 2 and 12 weeks post‑delivery. SLPPDS score of 8 or higher (moderate to severe perinatal psychological distress) | Planning to remain in the study area for at least 3 months. | Acute medical illness or evidence of severe mental illness. Comorbid conditions such as hypertension, renal disease or diabetes. Inability to provide informed consent or conditions impairing understanding (e.g. severe intellectual disability). | women | aged 18 or older | sex- and gender-based violence; intimate partner violence; food insecurity; low literacy rate among women; poverty and gender norms; patrilineal family structures include |
|  |  |  |  |  |  |  |  |
|  |  |  |  |  |  |  |  |
| Burgess et al. (2025) | depression and anxiety | They had mild to moderate symptoms of depression or anxiety, identified through screening tools. Eligible if PHQ‑9 score 5–14 and/or GAD‑7 score 5–14, corresponding to mild–moderate symptom ranges; baseline mean scores were 8.8 (PHQ‑9) and 4.7 (GAD‑7). | They also had an unmet need for family planning (meaning they wanted to delay or avoid pregnancy but were not using contraception). Most were within the first year after childbirth, often facing social and economic challenges. | Women scoring ≥15 on PHQ‑9 or GAD‑7 or reporting suicidal ideation were to be referred to other services and not enrolled, as the intervention was not designed for severe disorders | postpartum mothers | Recruitment targeted women aged 16–25; those enrolled were aged 19–25 years. | Many women reported social isolation after birth, high household and agricultural workload, financial worries, and stress related to political conflict. some had little or no schooling and low literacy, others had attended college; some were first‑time mothers, others had up to three additional children. |
| Chau et al. (2024) | depression in | Screened for depression using standardized tools (e.g., PHQ-9 or similar). | NA | Cognitive impairment. Severe depression, suicidal ideation, or psychosis. Impaired vision sufficient to prevent reading the workbook. Illiteracy, as the intervention relies on a written workbook | Of the 45 clients interviewed, 4 (8.9%) were men and 41 (91.1%) were women. | Clients ranged from 24 to 67 years of age; one client did not report age. | poverty, gender-based discrimination,  urbanization, internal migration, and lifestyle changes |
| Chau et al. (2021) | depression | having had a score of 7 or over of WHO self-reporting questionnaire 20-item | Adults 18 years and older, those who were recently bereaved or had seriously ill family members, had experienced marital breakdown, had experienced financial loss or bankruptcy, or were reluctant to leave their home or to engage in normal social or work activity. | cognitive impairment based on patient history; symptoms of psychosis, severe depression or suicidal ideation; impaired vision or hearing; illiteracy. | Mixed Gender | of the 376 patients in the study, 18.7% were over 55 years of age | NA |
| Chibanda (2015) | depression and other common mental disorders | score > 7 on Shona Symptom Questionnaire screen for common mental disorders. | aged 18 and over; married, residents of geographical patches in Mbare, Harare, covered by the ten selected lay workers; | requiring acute medical attention such that they cannot participate; severe psychiatric symptoms and/or risk to self or others requiring specialist referral | Most participants were women | 27 | HIV |
| Chibanda et al. (2017) |  |  |  |  |  |  |  |
| Davis et al. (2022) | Perinatal depression | a cut-off of 13 or above on the Edinburgh Postnatal Depression Scale (EPDS). | 18 years or over, were pregnant, no later than 28 weeks gestation, spoke isiXhosa, and screened positive for depression |  |  | 27.7 | - Participants experienced persistent poverty, food insecurity, poor housing, inadequate services, and high costs of nutritious food. - Social stressors included unemployment, absent family members, limited partner support, relationship insecurity, and financial insecurity. - Many faced physical, sexual, and economic abuse, partner alcohol abuse, HIV/AIDS-related challenges, and repeated traumatic events. |
| Henrique et al.(2021) | depression | Older adults who were eligible were screened for depression symptoms (PHQ-9 > 9) and included in the sample in case of a positive screening | 60 years and over | complete deafness; terminal illness; psychotic symptoms; risk of suicide; inability to communicate due to cognitive impairment or other health problem | Mixed Gender, Three quarters of those interviewed were women | 61 - 88, half of the participants between the ages of 70 and 79 | 75% had no income or received the equivalent of one Brazilian minimum wage |
| Jacobs et al. (2021) | CMD, specifically depression or problem alcohol use | NA | NA | NA | NA | NA | NA |
| Jacobs (2020) |  |  |  |  |  |  |  |
| Joag et al (2020) | People with emotional stress and/or common mental health problems | PHQ score at either sub-threshold level for CMD or above at baseline | NA | NA | men and women | men and women, with the majority being between 18 and 39 years old | Much of rural India works with an informal economy and employment is often seasonal or in clusters, particularly for work in the agricultural sector. There are many self-help groups (SHG) focused on discussing and carrying out income generation activity |
| Kidia et al. (2024) | Common Mental Disorders (CMD): Including depression and anxiety | SSQ, score > 9 |  | severe mental illness or suicide risk |  |  | Mental distress was commonly understood through local idioms such as kufungisisa or “thinking too much.”  Mental health problems were often linked to bodily symptoms, especially “pressure” or high blood pressure.  Participants commonly explained mental illness through social and financial hardship, rather than purely biomedical causes.  Problems bringing people to the Friendship Bench were mainly related to daily life stressors, especially financial difficulties and relationship problems.  Reported stressors included inability to pay school fees, lack of money for business or livelihood activities, partner absence or failure to provide money, domestic violence, bereavement, and loss of family support. |
| Munodawafa et al. (2017); Munodawafa (2018) | perinatal depression | Screen positive for depression with a cut off of 13 or more on the EPDS. | mothers with perinatal depression having children under the age of 5 years, attending one of two antenatal clinics (Michael Mapongwana Community Health Center (CHC) or Site B CHC) in Khayelitsha, presenting for their first antenatal visit, no later than 26 weeks gestation. Living in Khayelitsha. 18 years or older. Able to give informed consent. Speak isiXhosa as a first language (or competently as a second language). | Do not require urgent medical attention or have severe mental health problems, defined as a diagnosis of schizophrenia, bipolar mood disorder, or currently experiencing an episode of psychosi | women | NA | Patients are women experiencing perinatal depression. Many patients face poor socio-economic circumstances such as inadequate housing, intimate partner violence, and lack of social support. |
| Musyimi et al. (2019) | maternal depression | Some quoted participants were identified by age, for example a 23-year-old postnatal mother, a 31-year-old antenatal service user, a 20-year-old new mother, and an 18-year-old adolescent who had attempted suicide. | NA | NA | NA | NA | poverty, family conflict, lack of support, limited access to formal mental healthcare, and reliance on informal community providers such as TBAs. |
| Myers et al. (2019) | HIV, diabetes, alcohol use or depression. | at least 18 years old; (2) on antiretroviral therapy (ART) for HIV or medication for diabetes; and (iii) reporting hazardous/harmful drinking using the Alcohol Use Disorders Identification Test (AUDIT)17 or probable depression using the Center for Epidemiological Studies Depression scale (CES-D).CES-D measures change in symptoms of depression, with a cut-off score ≥16 indicating probable depression | NA | Patients receiving other mental health treatment or participating in another study were excluded | women | 44 | harmful alcohol use, HIV, Diabetes |
| Ng'oma et al. (2024) | perinatal depression | the women were screened for depression using a locally adapted and validated Chichewa version of EPDS with a cutoff point of 12 or more | a pregnant woman aged 18 years and above, between 24–28 weeks gestation, and living in one of the 8 randomly selected villages. | women with psychosis and other disorders requiring urgent medical or psychiatric care | women | aged 18 years and above | The majority of Malawians live below the poverty line, lower levels of completed education for women compared to men, Many women reported experiencing physical and sexual violence, with husbands or partners being the main perpetrators of physical violence, HIV prevalence was 10.8% in women and 6.4% in men, maternal mortality remained high compared to other African countries |
| Nyatsanza et al. (2016) | perinatal depression | Users who scored 13 or more on the EPDS were assessed using the Major depression module of the Mini Neuropsychiatric Interview (MINI) v 6.0 [46]. The women who were diagnosed with a major episode of depression on the MINI were interviewed for this study | NA |  |  |  | High unemployment, violence, crime, substance abuse and intolerable living conditions such as inadequate sanitation and overcrowding are rampant. Some of the township’s inhabitants live in corrugated iron shacks without running water and electricity although some live in formal housing |
| Pacichana-Quinayáz et al. (2016) | depression and anxiety | NA |  |  | NA | NA | Arm conflicts, social segregation, racial discrimination, government neglect and abandonment of the state, massacres and massive displacement of people |
| Paudel et al. (2014) | depression | NA |  | NA | NA | NA | significant social and financial stressors |
| Pereira et al. (2011) | depressive and anxiety disorders, also referred to as common mental disorders (CMD) | Patients in all facilities were screened for CMD by a trained lay person who was called the Health assistant (HA) using the 12 item General Health Questionnaire (GHQ-12) with a cut-off score of 5/6 |  |  | 70.6% were women | 41 | limited specialist resources and stigma |
| Rodríguez-Cuevas et al. (2021) | Depression (65%), anxiety (21%), and psychosomatic complaints (14%) | Generalised Anxiety Disorder Scale (GAD-7) score cut points indicate severity of anxiety as follows: zero to four points (minimal), five to nine points (mild), 10–14 (moderate) and 15–21 (severe), PHQ and suicide rate assessment. Severity of depression symptoms is indicated by the following cut points: zero to four (minimal), five to nine (mild), 10–14 (moderate), 15–19 (moderately severe) and 20–27 (severe). |  | NA | About 88.7% of them were women (n = 63) and 11.27% (n = 8) were men | NA | 70% of the population living with multidimensional poverty and 50% living in rural areas , IPV, violence. Around 42.2%% of enrolled patients (n = 30) disclosed to have suffered violence in the past or were currently living IPV. The forms of violence ranged from controlling behaviours, which was the most common, to physical and sexual violence. |
| Saleem et al. (2012) | depression and anxiety | presented with symptoms of depression and anxiety (based on assessment on Aga Khan University Depression and Anxiety Scale (AKUADS)); and capacity to give informed consent | age between 18 and 65 years; resided within 20 km of the centre, | As the primary outcome was subjective quality of life, patients were excluded if they had a mean score of 5 or higher on the Manchester Short Assessment of Quality of Life (MANSA) scale. | Patients were predominantly women (23/30), and married (28/30). Most of the caregivers were immediate family members (spouse, parent/child) (29/34), and women (24/34). All three of the counsellors were women. | the mean age of patients was 39 years (SD = 9.6 years). The mean age of caregivers was 35 years (SD = 12.0 years). | NA |
| Selohilwe et al. (2023) | depression among patients with co-existing hypertension. Patients with mild to moderate symptoms | 18 years or older, report receiving treatment for hypertension and screen positive for depression with a total score of 9 or more on the (PHQ-9) | NA |  | not clear | not clear | Poverty |
| Singla et al.(2020) | Perinatal depression | 280 pregnant women, aged ≥18 years with moderate to severe depressive symptoms, as defined by a PHQ-9 score≥10 | aged ≥18 years |  | women |  | NA |
| Szajna et al. (2024) | mothers with post partum depression | a) were 6 – 12 weeks postpartum, b) had no self-reported history of previous substance use disorder or psychiatric illness, c) were 18 years of age or older, and d) could read and write in the local dialects of Kannada or Marathi and screened positive for mild to moderate PPD. | NA | 1) women who answered affirmatively to a question indicating thoughts of self-harm) and 2) women with severe depression (scores on the screening instrument of more than 14). Women with either of these exclusion criteria were referred to the team psychiatrist. | women | a mean age of 27 years, with an age range from 20 to 35 years (standard deviation 4.9) | domestic violence, and economic difficulties are contributing factors, as well as local practices for postpartum mothers such as limiting food and drink and restrictions on leaving the house or socializing for several months |
| Wood et al. (2021) | a range of mental illnesses including depression, anxiety, alcohol use disorders, autism, and severe mental illnesses | NA |  |  | NA | NA | Vulnerable populations such as those who live in poverty, are homeless, affected by domestic violence |
| Yator et al. (2022) | Postpartum depression | participants who scored > 10 on the Edinburgh postnatal depression scale and who were 6–12 weeks postpartum were eligible for the study | PPAs aged 15–24 years old and living with HIV, 6–12 weeks postpartum |  | All young women | 18 - 20: (n= 3), 21-24: (n=21) | HIV, and 54% participants experiencing IPV |

**Supplementary Table 5. Qualitative Data Extracted from Included Studies**

| **Authors (year)** | **Experiences** | | |
| --- | --- | --- | --- |
|  | **Authors' themes** | **Participants' Quotes (First order)** | **Authors' descriptions (Second order)** |
| Abas et al. (2016) | Acceptability and potential benefits | *A patient seems happy by just the progress that we’ve made in that they’ll maybe repeatedly say,  “Ok, I am going to try ABCD then I will come and see you on such and such a day”* | Similar kinds of opinions were expressed by the LHWs, many of whom referred to the first stage of the intervention as kuvhura pfungwa (Shona for ‘opening the mind’). Several elements of the intervention emerged as socially and culturally appropriate. These included the use of a screening tool which incorporated local terminology for emotional distress, especially ‘kufungisisa’ or ‘thinking too much’ which is a common idiom of emotional distress which shares features of both rumination and worry |
|  |  | *You will leave the session feeling better because you would have talked about things.’ (Patient) ‘By talking about why they are there, I open up their mind in that I try and get them to understand that they can get help* |  |
|  |  | *Talk therapy is the best, that’s the best way, you pick up certain stuff that they are saying, you pick  up the lifestyle that they are leading, you pick up certain things that maybe they used to do and  they are not doing* |  |
|  |  | *Patients that suffer from kufungisisa (thinking too much, a Shona idiom of emotional distress)…all  seem to benefit somehow.’* |  |
|  |  | *Through all this therapy they now manage to continue taking their medication the way they are  supposed to* |  |
|  |  | *‘SSQ helps me to see how the person is surviving because for some they will not even be able to  do their work. For some even to take their dishes outside, they cannot even do that. So it helps  me to know that.’* |  |
|  | Facilitating factors Socio-cultural appropriateness of the LHWs | *They call us Ambuya Utano.’ (grandmother health provider)* | Patients often spoke of the LHW as the ‘granny’ and the LHW often referred to the patients as ‘my daughter in-law’, ‘my son’, underlining the perception of the LHW as almost family members in their role in the community. Patients and staff indicated that in addition to being respected in the community, LHWs also commonly  shared the same social and economic problems as their clients, and as such were seen as very suitable to deliver the intervention. they have status and possess an intimate knowledge of the local language, norms and context, and a unique social and cultural understanding of the issues facing their patients. |
|  |  |  | Most patients expressed trust and respect for the LHWs. The LHWs were also seen to possess certain personal attributes and competencies which further facilitated their ability to provide mental health care. |
|  | Patient-focused and flexible approach | *‘Women from our community want to sell vegetables in the morning, not sit on the Bench, so we  see them in the afternoon when they are ready to talk.’* | They were commonly described, by patients, their supervisor and by each other as being ‘approachable’, ‘trustworthy’, ‘mature’, ‘motherly’, and possessing ‘strong listening skills’. LHWs were said to have ‘big ears, a small mouth, listening nodding whilst the person is talking’and ‘having [patients’] matters at heart’. Moreover, the LHWs connectedness to the community seemed to enhance their commitment to the intervention. Both patients and LHWs highlighted the patient-centred and flexible nature of the intervention, whereby it could be tailored to patient needs, such as having sessions conducted in their homes or being able to self-refer. This allowed counselling to be integrated alongside the wider health promotion duties of the LHWs. Numerous LHWs described how the ‘empowering’ nature of the therapy is rewarding not only for their patients, but also for themselves, providing personal satisfaction. The LHWs emphasised that they spent a long time in the first session with the client, generating solutions as well as identifying problems ‘in case the client does not come back |
|  |  | *It is like planting a seed, watering it to make it grow and then meeting to remove any weeds that  had sprung up* |  |
|  |  | *‘We hear, talk, empower the patient. We do not tell them what to do* |  |
|  |  | *‘She is the driver and I am the leader… [I] get in then the driver drives* |  |
|  |  | *We offer counselling to empower people to help themselves… When there are changes I feel  good that I have helped someone* |  |
|  |  |  |  |
|  | Challenges to implementation Socio-economic circumstances | *A case I found difficult is when the person needs assistance immediately. One day I actually used  my money after realizing that the situation was really desperate.’ (LHW) ‘They come in thinking that any problem, no matter what it is we can intervene, so we are more  like a social work office sometimes. We end up being that way- where people are displaced and  want to come and get assistance (LHW)* | Various potential challenges emerged. For example, despite the many benefits of the LHWs’ links to the community, this could also be testing. Most reported often being approached by community members with situations that they felt required their urgent attention and personal support. Relatedly, many patients face a host of desperate problems, such as poverty, HIV/AIDS and domestic violence |
|  | Supportive supervision structures | *We work very well just helping each other. If you make a mistake you are corrected and we help  each other.’ (LHW) ‘We have one day aside on a Wednesday when every LHW gives feedback on a case that they’re  working on, or even issues that are going on in the community.’ (LHW) ‘We work together, we work as one team.’ (LHW)* | All of the LHWs reported that their peers and the higher cadres made them feel well-supported to conduct the intervention,  and indicated that this contributed to the success of the intervention. This may explain the quantitative findings showing the drop in referrals they are making to the psychiatrist compared to 7 years earlier. |
|  | Insufficient training and financial support | *‘I would like to learn about such issues like dealing with a person who wants to commit suicide.  …I might have my own way but I would like to be trained so that I do not do anything that  lands me in jail after having let someone go ahead with committing suicide.’ (LHW) ‘There is no structure or system that’s there to support me when I’m in a situation like that…’  (Supervisor referring to lack of supervisory support for dealing with challenging clients) ‘The money that we get is not significantly different from those who do not work… There should  be a difference… Apart from our uniforms, we need bags to show that we are going to work.’  (LHW)* | Many LHWs and their supervisor indicated insufficient levels of training, such as for assessing and managing suicide and dealing with hostile patients, and the supervisor wanted supervision for herself. Moreover, although most LHWs expressed a strong commitment to the intervention, two suggested a need for further financial reward. Another potential challenge that emerged was the lack of followup and comprehensive documentation. Poor documentation could at least partly explain the quantitative finding of a lower than expected recorded number of follow-up appointments, although this could also be due to lack of training about need for follow-up of more depressed patients, or lack of financial incentives for LHWs. |
|  | Lack of follow and comprehensive  documentation | *‘The patient is the one who tells me when she wants to come back, maybe after a month.’ (LHW) ‘I do not dictate the pace.’ (LHW)* | Poor documentation could at least partly explain the quantitative finding of a lower than expected recorded number of follow-up appointments, although this could also be due to lack of training about need for follow-up of more depressed  patients, or lack of financial incentives for LHWs |
| Ahmed et al. (2024) | Client Reticence and Confidentiality Concerns | *“The first session that’s where I get problems where I have to make the client to feel comfortable when talking to you because some of these clients [they] get to see you so trying to talk to them when they had seen you before it’s a barrier… I have seen [one client] around because we live in the same neighborhood.”* | All but one of the peer counselors revealed that clients are usually reticent or reluctant to confide in them during initial sessions. According to Neo, adolescents may fear that the counselors will disclose information shared during the counseling sessions with the doctors at the clinic. Two counselors, Tebogo and Mpho, suggested that clients may not feel comfortable confiding in counselors whom they already knew or have seen prior to the counseling sessions |
|  |  | *“In most cases it’s not every youth or everyone that’s free to talk to someone their age… so most of the time… the clients that we see here know us… Maybe it’s just the human mind… if I tell you something personal and I see you in public… automatically I will start thinking maybe you told someone or you are going to just say what I told you out and everyone hears it.”* |  |
|  |  | *“…the first session I should think is the most difficult because this person doesn’t know whether to trust you or not because of how fast the information can travel nowadays. So, it’s a very difficult thing but as time goes, as the client [begins] to trust you, it’s fine. The problem is when you start the session”* | Despite initial concerns regarding confidentiality and privacy, counselors agreed that client trust usually improves as the sessions progress |
|  |  | *“The issue of them not opening up was for [me] to set up a platform where I can make them feel free that this is just me and you. [I] get them to tell [me] about themselves, what they are doing, school, what they like doing, hobbies… to make them open up… we just had to do icebreakers.”* | Some counselors explained how they resolved this challenge by ensuring confidentiality and utilizing communication strategies to gain trust and establish rapport among their clients. |
|  |  | *“I once had a client who never really said anything… [he] wasn’t really opening up across all repeated sessions and we then ended sessions just like that, we didn’t cover much though he had a few list of things… there were like four or five problems and then some of them we didn’t go over them… So we only attended like two… we should be glad as a counselor that at least you have managed to cover other problems because maybe [there is] one problem [that] needs two sessions or three sessions.”* | Oratile recommended an increase from six sessions to twelve, when necessary, to accommodate clients who may take longer to open up or have problems that take longer than six sessions to address. |
|  |  | *“But most of my clients… they left here very happy, to the point in whereby even after the whole sessions were done, even when they see me in the streets they will be like ‘Oh! Wow! I wish I could see you again, I wish we could have your contacts or something like that.’ I am not allowed to do that so I was like ‘you could just keep see[ing] me here [at the facility] or whatever’”* | Among her reticent clients, Lorato would foster rapport building by saying *“let’s just sit down and pretend like you and I are friends.”* Although most counselors were eventually able to develop camaraderie among their clients, they also had to reinforce professional boundaries |
|  | Scheduling Procedures | *“The challenge is always like coming here and also rescheduling issues… because maybe I will come here knowing my client will be here at two o’clock and then at half past two they will be telling me ‘no, I have family issues’… and then we reschedule to a different date [to] accommodate my client with me now, and I have to make sacrifices for my client because I understand it’s one of the things that we were told about. That sometimes you are going to have difficulties that you are going to invade some of the personal, private time” (Oratile).* | Several counselors implied that the initial procedures used to schedule clients for counseling sessions were barriers to Safe Haven implementation. At the beginning of the pilot, when counselors were scheduling their clients by appointment only, clients were missing their scheduled appointments or attending late |
|  |  | *“if we decided that we should meet with the client around one, then the client decides to come around two… it’s a problem because I do have other commitments outside there. So, waiting an hour for a client is a problem.” (Tebogo)* |  |
|  | Lack of Financial Resources | *“I am not financially stable” (Atujuna et al.) and “the money that we get is not even enough to support our own selves” (Obonye).* | Several counselors emphasized the need for basic financial support, particularly more transport funding to carry out their job responsibilities. Three counselors expressed that their current pay was not enough to afford transportation to the location of the counseling sessions, especially with recent fare increases |
|  |  | *“I’ve invested so much in this program but the rewards are not necessarily meeting my expectations.”* |  |
|  |  | *“My wish is for this thing to not just be a volunteer thing for us. I mean we are the first people in Botswana to having started this. And I am sure in the future we are going to have more people being a part of this, right? So, my wish is that we should not be seen just as volunteers… We may not be qualified like degree, diploma kind of qualified, but certificate yes, we have experience of a year by now. We have an experience of a year, and at some point in the future they should consider it for us it’s a job.”* |  |
|  |  | *“money is a problem… sometimes [the clients] will just pop up out from nowhere… Imagine if I get a call like now to come here… it’s gonna take me a long time, it’s gonna need money also. What if at that certain time I don’t have any money with me? You see that’s the kind of problem that we face” (Obonye).* |  |
|  | Counselor Psychological Wellbeing | *“say something that will remind [him] of [his] own problems” (Stockton et al.).* | Counselors commonly reported challenges with experiencing the same hardships as their clients which can compromise their ability to provide problem-solving therapy. One counselor shared that his clients would say something that will remind him of his own problems. These emotionally triggering experiences can make it difficult for counselors to continue with the therapy sessions and may therefore prompt an unwanted referral to another counselor. |
|  |  | *“open up some old wounds [and] bring some flashbacks” (Obonye).* |  |
|  |  | *“I can make an excuse to go drink water” (Oratile) and “I had to pretend like I wasn’t going through what [the client] is going through” (Maphisa et al.).* |  |
|  |  | *“there are scenarios where my client will be talking about [a] problem that I am basically going through right now. But when I try to refer him to another counselor, he doesn’t want that other counselor. He wants specifically to see me.”* |  |
|  |  | *“when you have cases that hit close to home, sometimes it can happen that you [are] the best person to help the client… that’s the only reason why I didn’t refer it… I try my best to still be objective [and] help the clients find solutions.”* |  |
| Atif et al. (2016) | Linkage with local PHC system | *Nobody knows about us whereas LHWs are working for the last 18–19 years. It would be really difficult for the PVs to work without their involvement. 33PV* |  |
|  | Intervention perceived positive | *In every following session I used to ask her about her mood and daily routine. She used to tell me what she has done, how she has taken care of her baby. This makes me realise that she is following my suggestions. Moreover the improvement in her says it all. 01PV* |  |
|  | Personal gains | *It has given me an opportunity to learn and gain experience. I hope I can get LHW’s post, as I would like to contribute to house income and want my daughter to feel proud of me. 07PV* |  |
|  | Family and community endorsement | *My family is supportive, without their encouragement, I would not have done this work. It would have been really difficult to leave housework and children. 01PV  They ask us about our programme, about our role, where we have received our training, how do we operate, what do we do? When I explain to them they get satisfied, knowing that we are doing this work with an objective. PV05* |  |
|  | Good training and supervision | *I like the training…the trainers were friendly…the way they explained the content was very good. I didn’t experience any problems in understanding it. Through it I have learned a lot, which has helped me to overcome my own depression. 15PV.  When xx (supervisor) accompanied me, mothers took me more seriously and shared their concerns more openly knowing that I have been properly trained and supervised. IDI-15PV* |  |
|  | Stigma of mental illness | *She got upset when I told her that the assessment indicated that she has depression. She said that she is not mad and stopped me from coming in when I went for my next visit. 22PV* |  |
|  |  | *They say that she (mother) is being possessed, so instead of medicines they go for talisman (spiritual treatment). 19LHW* |  |
| Atif (2015) | Acceptance of the PV and factors contributing to it > Ability to relate to the mothers > Experience of being a mother | *When they were telling their problems, I used to tell them that I had similar circumstances but God has helped me to overcome it. Time never stays the same, I was stressed when my children were young, now my tension is gone. Mothers used to benefit from my experiences. IDI-33PV* | All PVs, except one, were mothers themselves and about a quarter revealed that they had experienced perinatal depression. These experiences provided the PVs with good understanding of the mothers’ issues and the ability to empathise with them. The majority of PVs felt that being a mother was facilitative to their role and reported sharing their personal experiences with the mothers |
|  | Acceptance of the PV and factors contributing to it > Ability to relate to the mothers > Gaining mothers’ trust | *It’s all about maintaining confidentiality, if we reassure them of confidentiality, we will be able to gain their trust… gaining trust is everything, once we gain their trust then everything else will become effortless. IDI-07PV* | A PV gaining a mother’s trust was crucial before a significant relationship could be established. The majority of the PVs reported  gaining the trust of most of the families they were working with. This was achieved because they could relate to the mothers, gently assuring them of confidentiality and giving them time to build trust before making any attempt to talk about the sensitive issues. |
|  | Acceptance of the PV and factors contributing to it > Ability to relate to the mothers > Befriending | *They were same age as me, I was friendly with them. I told them that I have been through all this. I used to sit side by side with them, if they were sitting on floor I used to sit on floor, then they listened attentively to you and took things on board. These are poor rural people; they listen to you and regard you as their well-wisher. IDI-33PV* | The PVs’ acceptability was enhanced through befriending with the mothers. The majority of these mothers were socially isolated and were experiencing loneliness (see Section 6.1.1.2). The PVs provided them the opportunity to develop new relationships and participate in social activities through attending group sessions. More than half of the PVs recalled forming a friendly relationship with the mothers, who were similar age to them, by not just having a purely professional relationship with them, but by befriending them, sitting alongside them, being friendly with their families and even sharing their own personal information |
|  | Acceptance of the PV and factors contributing to it > Ability to relate to the mothers > Being local | *Mothers will only talk openly to someone who is locally based. It is a fact that only a local person can understand their problems because she has gone through similar problems. Here in village people have different circumstances and almost everyone is experiencing problems of some sort. IDI-33PV* | Geographical proximity to the mothers was another key factor facilitating acceptance. Like the mothers (see Section 6.1.2.1), PVs expressed advantages to being local, such as their social, cultural and linguistic awareness, their knowledge of the local community and even personal knowledge of the families that they would serve. PVs believed that this made the mothers feel at ease to express their issues. Additionally, their linguistic compatibility with the mothers enabled the latter to speak freely in their local dialect (Potohari). |
|  |  | *Not everyone has resources to look after their diet as suggested, so I review diet chart with them in accordance to their circumstances, otherwise it will be no good. For example if they can’t afford to take full glass of milk I suggest them to take half a glass. IDI-01PV* | In addition to helping the mothers share their personal issues, the PVs further helped mothers to address their problems by making use of their understanding of local psychosocial sensitivities. This knowledge provided the PV with the right tools to explore appropriate solutions to their problems. With PVs not being local, there could be a risk of proposing an option which was either not implementable or against the rural norms. |
|  |  | *She (mother) wanted to attend the group sessions. I tried to convince her family, but they didn’t give permission. So I held the group at her house. It was very useful for her. IDI-28PV* |  |
|  |  | *I was from a different village so they trusted me. They felt comfortable knowing that I didn’t know anyone in the village and therefore their information was safe with me. IDI-28PV* | However, some PVs felt that familiarity can reduce the ‘professional’ status of PVs, with less value given to their knowledge and skills. Moreover, sometimes a longstanding family feud might involve the PV and her client’s family, and in such cases it would be difficult, if not impossible, to fulfil this role. Lastly, the perceived risk of breach of confidentiality is also higher if the PV and the mother both reside in the same village. |
|  |  | *A mother living in the same village as her PV might not pay much attention to what she is being told. Whereas if a PV is from a different village, she will think that she has come from far and acknowledge her better. Moreover if she is from same village, mother might or might not have good relationship with her. If the families have interpersonal conflicts, the PV might feel uncomfortable visiting them. IDI-PV05* |  |
|  | Appropriateness of the intervention > Simple-to-deliver and intuitive intervention | *We say to mothers, they know everything, but usually they are so busy in raising their children and housework that they tend to ignore their health. We remind them the importance of taking good self-care in order to remain healthy and to look after their family. IDI-01PV* | The content of the intervention was simple and intuitive (Section 4.1.3). The key messages given were relevant to the mothers’ current circumstances and intuitive to both its receiver and deliverer. They were targeted to improve mothers’ wellbeing and were delivered in a sensitive and culturally appropriate manner. |
|  |  | *In the group they get the opportunity to learn from each other and understand that other women are going through similar problems. I think this understanding helped them in certain ways. IDI-14PV* | Almost all PVs’ appreciated the mixed method of delivery and expressed views similar to the mothers. Most PVs felt the groups facilitated learning from each other experiences and individual sessions allowed sharing personal experiences which could not be discussed in a group setting due to the fear of disclosing information and the concerns of bringing shame and embarrassment to their families. |
|  |  | *They do not share personal information during group sessions. In villages they believe that if others will find out about their domestic issues, they will gossip about it which will dishonour their family. They only shared such information with us at their homes. IDI-15PV* |  |
|  | Positive feedback reported or observed from mothers | *In every following session I used to ask her about her mood and daily routine. She used to tell me what she has done, how she has taken care of her baby. This makes me realise that she is following my suggestions. Moreover the improvement in her says it all. IDI-01PV* | Another factor important for the motivation of the PVs was the positive feedback reported or observed from mothers or their families. During each session the PV assessed the mother’s mood through recording her response on the mood chart (pictorial illustrations representing mood on a 5-point scale ranging from very sad to very happy). In addition they relied on accounts given by the mothers and their family members about their general wellbeing. Almost all PVs reported noticeable improvement in the mothers’ mood because of the support they were receiving from them. In addition, their improved mood was both the cause and effect of their improved interactions with their family and better care of their diet and rest. |
|  |  | *Her mother-in-law says that she is thankful that such help has been offered to them. The mother has now started talking to them, before she was keeping herself to herself. All the time she used to sit quietly, neither eating nor asking for anything else. She is so happy now that it seems magical. IDI-15PV* |  |
|  | Challenges in working as a peer volunteer > Lack of engagement > Lack of interest | *She demanded for some monetary help. She seemed more interested in money and asked me what will I give her and how otherwise are my visits helpful for her? I said that I will give you information about mother and child health, but she seemed more interested in tangible gains. IDI-PV05* | The PVs described various reasons which they believed lead to the mothers’ lack of interest in the intervention. Some PVs felt that lack of familiarity with the talking therapy combined with the lack of material incentives made the intervention less attractive to some mothers. Another reason could be a mismatch of educational qualifications; for example one PV felt that her skills were undermined by two mothers who possessed higher educational qualifications than her, resulting in a lack of engagement. Some PVs felt that the lack of interest could also be the manifestation of some underlying issues such as their denial of mental health problems, time constraints or family pressures. However, at times these reasons were not disclosed to the PVs, with the mothers making excuses to avoid receiving sessions. |
|  |  | *Initially they were not engaging with me. Both were graduates and more educated than me. They made it clear to me that they were more qualified. IDI-15PV* |  |
|  | Challenges in working as a peer volunteer > Lack of engagement >Lack of trust | *Women do not express their concerns because of fear of information being disclosed. Often the nature of domestic issues is such that mothers feel disclosure will disgrace their family’s standing in the community and will lead to family confrontation or argument. IDI-01PV* | As discussed in Section 6.1.4.1, the PVs being local and working in close knit communities led to a perception of risk of disclosure of information to the person/s known both to the mother and her family. Most PVs felt, this preoccupied some mothers and created a barrier to their work. The PVs acknowledged mothers’ fears of information being relayed back to their families resulting in domesticconflicts or word being spread in the neighbourhood resulting in bringing shame and disgrace to the family. In both cases a mother would be held answerable to her in-laws. |
|  | Stigma of mental illness | *They will deny or worry about being unwell. This will impact their mood adversely. Moreover some people feel ashamed of it, believing that it is either their family or their circumstances to be blamed of… as poverty is the biggest cause of depression. IDI-07PV* | The stigma of depression posed a major risk to the acceptability of the PVs who were working with the depressed mothers. During the training the PVs were made aware of the stigma of depression as a significant barrier to engagement and they were trained to deal with the issue sensitively. The majority of them were able to deal with the issue when it arose, avoiding unnecessary stress or embarrassment to the mothers, or resistance to the intervention. PVs discussed how they had tackled this issue, rather than how it had acted as a barrier. However in one case, a PV failed to address the issue sensitively, leading to a mother’s withdrawal from the study. |
|  |  | *She got upset when I told her that the assessment indicated that she has depression. She said that she is not mad and stopped me from coming when I went for my next visit. IDI-22PV* |  |
|  | Resistance from mothers’ families | *She (mother) said that her husband was saying that they (research team) have taken all the money and therefore just doing talking with us, without any monetary assistance. IDI-15PV* | Another likely challenge for the PVs was resistance from the mothers’ family. The mothers tended to abide by the decisions made by the elders in the family and therefore their approval of the PVs was crucial. There were several factors indicated by PVs which could influence the family’s opinion about them. In Pakistan, the perception of corruption is quite high and has resulted in citizens’ general mistrust of both the government and non-government organisations. New initiatives such as the one under which PVs were working can be looked at with suspicion. This can in turn undermine the PV’s role. Some PVs have reported the families’ misconceptions about their role, for example, the PVs corrupting their daughter-inlaws, instigating hatred towards them or encouraging them to go against their family’s wishes. |
|  |  | *If a family don’t know anything about a PV they might start doubting her intentions. They will wonder why she has come, what she is talking about, is she making the mother go against them or encouraging her to get a job. This made them worry about her housework responsibilities. IDI-07PV* |  |
|  |  | *Two mothers who were in their chilla period did not get permission from their families to attend group. IDI-07PV* | Resistance could also be linked to cultural beliefs, practices and superstitions. For example the chilla period was perceived to be a vulnerable period for the mother and therefore she was discouraged from attending the group sessions. If a baby is born prematurely extra precautionary measures are taken by the family. For example, families do not allow anyone to meet the mother or her baby, believing that it will bring harm to them. This could then stop a PV being able to contact the mother during this period. |
|  |  | *A baby was born to xx (mother) one month and five days early. In our village if a child is born before full term, family do not let anyone see the mother or the baby. They fear that it will cast a shadow which can make the baby grow weak and unwell. Some families believe that a mother shall not get near her child after combing her hair, or taking a bath neither do any other women during her menstrual cycle. IDI-15PV* |  |
|  |  | *I went to her house twice but they didn’t like me coming to their house, especially her motherin-law because of our family feud. She used to think that I am coming to spy on them. IDI-33PV* | Lastly conflicts or strained relationships between a PV’s family and a mother’s could cause a barrier to the delivery of the intervention. Such feuds can be very long-standing and destructive to individual relationships. One mother refused to see her PV because her in-laws were in dispute with the PV’s in-laws, and therefore her intentions for coming to their home were viewed with suspicion. |
| Atif et al. (2017) | Views about the intervention | *Mothers who cannot read understand the information through looking at these pictures in the manual. They talk about the pictures but actually they relate these to their own problems”. (Peer, Rawalpindi)* | The intervention uses the power of imagery and narratives to encourage mothers to become more active. Similar to the views expressed by mothers, the majority of peers felt that both the stories and pictures were effective tools for communicating, that mothers could relate to them and that they were motivational in improving mothers’ wellbeing. |
|  |  | *“You can tell that a mother is feeling better through her response to the mood chart and when she smiles and when she shares her thoughts and feelings with you.” (Peer, Goa)* | During each session the peer assessed the mother’s mood by recording her response on a mood chart (pictorial illustrations representing mood on a 5-point scale ranging from very sad to very happy). In addition, they relied on accounts given by the mothers and their family members about their general wellbeing. Almost all peers received positive feedback from mothers or their families about the intervention. |
|  | Views on potential untoward effects of the intervention | *I was awake all night because a mother told me not to come anymore. I got tensed, I didn’t speak to anyone, I was thinking I might have upset her or she didn’t like me, why she didn’t like me, why did she behave like this…what was her reason for doing that” (Peer,Rawalpindi)* | In Pakistan, a peer experienced stress because a mother wanted to withdraw from receiving the intervention. Her concerns were addressed sensitively during supervision. |
|  | Views on motivation of the peers | *“People ask us why we are going for this job if it does not pay us. If we got monthly salary, then, it would be good. We will also be interested in working.” (Peer, India).* | In Pakistan, peers were happy to work on a voluntary basis, however, in Goa, India, the peers expressed the need for some kind of incentive structure for this programme to be successful and to hold their interest. |
| Bah et al. (2025) | Feasibility | *Well, sometimes I visit them, and sometimes they come to meet me, but most of the time, I visit them. When you call them, they will tell you they are busy, “I am working,” “my child is this or that.” I will say to them, OK, I will come …. [ ] … like this other lady here, plenty of people were there, so when she sees me, she will wink her eyes to me and go inside and say, “aunty Fatmata has come.” So, her partner will excuse us, I will talk to her inside her house, and after talking with her, we will come outside. [MMSG 4]* | A new mother stated that the PST session was a two-way visit, rather than just perinatal women visiting the MMSGs, and provided the intervention with greater flexibility. A MMSG also explained how easy it was for them to support pregnant women and new mothers and the way they were able to work through their shared interests |
|  | Appropriateness | *I felt good because, firstly, through this training, it gave us the confidence to go and talk to perinatal women in the community. Some homes nearly fell apart, but because we went and talk with them, with the help of God, they came back together. So, that in itself is a good thing … [ ]. After all, we told them that if they have this problem, it is not nice to lock themselves in their rooms. Sometimes, you “play music,” “watch movies,” or “dance,” which is good for their wellbeing. [MMSG 6]* | The MMSGs expressed that the PST-FBI was congruent with their role’s expectations as volunteers. They were happy that the PST-FBI offered them skills and a structured way of supporting perinatal women that they never had before: listening, nonjudgmental, dealing with a difficult client, and problem-solving skills. According to one of them, the MMSG claimed that they were able to form a therapeutic alliance with the perinatal women and support them throughout the four sessions of the intervention: |
|  | Fidelity | *We followed what you trained us. We used to advise people, but quite different from your approach. You came and taught us a new method, that they should talk to us, and we should listen. And after they finished, we work on the problem disturbing them, so I think this is better. The method is good. When someone is talking about their issue, that is not backbiting. You are the one who said it, and you suggested how we should go about it through the brainstorming process, I am here just to guide. [MMSG 8]* | We evaluated MMSG adherence to protocol during the supportive supervision by reviewing their notes and discussing randomly selected cases. For each MMSG, we observed one of their sessions, noting the duration, adherence to activity order, joint activities, handout provision, next session scheduling and the relationship with the perinatal women. Feedback from participants was gathered to assess their engagement and the relevance of the content delivered. This comprehensive approach helped to ensure that the intervention maintained its integrity, while also providing insights into areas for improvement. A debrief with the MMSG followed each observed session. One MMSG described how she handled the PST-FBI with a particular perinatal woman: |
| Burgess et al. (2025) | Workload and responsibilities | *“I provide services like vaccines, family planning, monitoring, first aid and health education”“the program is vast and huge and has many activities within it.”* | In the first interview, prior to Mothers Time training, HEWs were asked to describe their current workload or responsibilities. They described long days, busy schedules, including lots of engagement with pregnant women and women who recently delivered. “I provide services like vaccines, family planning, monitoring, first aid and health education” said one HEW, explaining that, “the program is vast and huge and has many activities within it.” One key responsibility for HEWs is engaging with women at multiple key touchpoints before and after a birth, providing services and counseling on breastfeeding, vaccines, infant health, FP, and more. “We see a mother seven or eight times” one HEW explained, noting that through this long-term engagement, “we can establish a strong relationship.” |
|  |  | *“We see a mother seven or eight times” “we can establish a strong relationship.”* |  |
|  | Current approaches to mental health and Family Planning | *“We show them the picture of different family planning options like Depo for three months, pills for 28 days, or the implant for three years”* | HEWs provided details about their current approach to postpartum FP, noting that FP counseling typically happens at 42 days postpartum. HEWs described meeting mothers 1-on-1 and using visual aids to discuss different methods. |
|  |  | *“The only thing we can do is provide advice and make them feel strong.”* | HEWs described varying approaches for supporting mothers in their communities who seemed to be struggling emotionally. “The only thing we can do” one HEW explained, “is provide advice and make them feel strong.” Another noted, “I may spend more time on an emotionally stressed mother than a healthier one.” In a case where a mother had been abandoned by her husband, one HEW described collecting funds to support her. Overall, HEW were already well immersed in postpartum mental health and FP through their daily work, but had more tools and training specifically for FP. |
|  |  | *“I may spend more time on an emotionally stressed mother than a healthier one.”* |  |
|  | Feasibility of training | *“The contents were good” one HEW noted. “The training was delivered with examples, and simple language we can understand.”* | In training, many mental health concepts seemed new to HEWs. HEWs asked for clarification on several terms, and the trainer provided examples to facilitate understanding. HEWs shared their own examples of encountering women with postpartum mental health challenges, suggesting they understood basic concepts and the content resonated with their current experiences. In interviews, HEWs described the training as engaging and relevant. “The contents were good” one HEW noted. “The training was delivered with examples, and simple language we can understand.” |
|  |  | *“I didn’t have good knowledge about mental health before, but now I’ve had good lessons from the training.”* | HEWs appreciated the opportunity to learn more about mental health, with one noting, “I didn’t have good knowledge about mental health before, but now I’ve had good lessons from the training.” Although the HEWs developed some basic competencies through training, the HEWs and the trainer noted that two days were not enough for full mastery. Thus, the trainer provided coaching and supervision throughout implementation. |
| Chau et al. (2024) | Theme: facilitators and benefits Client-provider relationship building | *“I have to dedicate [time] and be subtle when visiting a client… [especially] if you meet their family. You could make some [jokes] or have lunch with them. And then when their family [goes] out, [leaving] you and [the client], you could start to talk to them. They will be more open and won’t be embarrassed.” (Provider F, Quang Nam)* | Due to the nature of the client-provider relationship and the fact that individuals with depression can be hesitant to socialize, it is up to the providers to take the lead in establishing a positive initial interaction and a relationship through friendliness and openness. One key factor that outlined successful client-provider relationships was the importance of providers building trust and rapport with clients over time. |
|  | Family and community connections | *I came to their house, not only because I’m the [social] collaborator, but also because I’m the head of the village, so I understand and know all the [family] situations in the village. So [if anyone] needs to talk to [me], I will [go] to their house to meet them and talk.” (Provider G, Da Nang)* | Another sub-theme supporting engagement with SSM was the importance of family and community connections. Clients identified strong social collaborators as being members of the communities in which they live and work. Because social collaborators live in the community and often hold concurrent jobs as commune health workers and/or community representatives, they have existing relationships with the clients. They may have a history of previously working with them on other local health initiatives and may be on friendly terms, or at least familiar with the client and their life circumstances. Providers working in their home communities have the benefit of existing connections, so they are not building trust from the ground up. |
|  | Theme: barriers: Clients' responsibilities | *“Based on the client their schedule, also their work [hours] and mine, I have to make it work for both of us.[…] I have to choose a time that would work for both of us.” (Provider S, Da Nang)* | This posed a challenge to finding appropriate times for providers to visit the clients, with providers reporting struggles working around client schedules. Sometimes providers had to visit a client multiple times before the client was available to engage with them, and providers were not compensated for the additional time and transportation costs. |
|  | Clients’ health conditions | *Many [households] are welcoming… I talk to them about this program [and] they try to understand and [are] supportive, but there are many houses that [don’t] understand so they get annoyed. (Provider P, Quang Nam)* |  |
|  |  | *“In this [locality], when we find a client, that client is assigned a [social] collaborator. [Social collaborators] consult with the client following the PHQ-9 assessment, but that client [does not] want to accept that she has depression because she is scared that people around her [will] know about her [disease]. For example, a client… dropped out the program because she [didn’t] want to share that she has depression.” (Provider T, Ben Tre)* | Stigma was identified by both clients and providers as a barrier to participation in the SSM intervention, although it was more frequently brought up by providers. Providers shared how not all clients were receptive of engaging with the SSM and providers once it was underway and how some clients dropped out of the intervention or were hesitant to open up to them because of the negative associations around depression. As one provider reported: |
|  |  | *From my point of view, maybe they were afraid of being interviewed. Many clients might think that I would [schedule an] interview with them to swear at them because they are crazy…” (Provider V, Da Nang)* |  |
|  |  | *“In this [locality], when we find a client, that client is assigned a [social] collaborator. [Social collaborators] consult with the client following the PHQ-9 assessment, but that client [does not] want to accept that she has depression because she is scared that people around her [will] know about her [disease]. For example, a client… dropped out the program because she [didn’t] want to share that she has depression.” (Provider T, Ben Tre)* |  |
|  |  | *“For example, [clients] avoid me… they do not want to see and talk with anyone at first, so I have to encourage their family, then [I will] gradually contact them. It is difficult to see [the client] immediately.” (Provider Q, Quang Ninh)* | Providers also shared that one of the strategies they used to gain the trust of clients is to first gain the support of family members. While some families did not want to engage with social collaborators due to low mental health awareness, many were happy to welcome the social collaborators, encouraging their family member with depression to participate |
|  |  | *If their [condition is serious], they need help from their family members… When we guided them, we told them that there are many activities [they can try and] have fun. If they were too sad, we showed them how to [develop routines], for example: wake up [early], clean the house and do physical exercise [every day]. I would recommend 30 minutes for exercise, but if that’s not possible we can [aim for] 20 minutes. Often, they didn’t want to, so we have to create motivation… their family members have to join them to let them get used to these activities.”(Provider R, Da Nang)* |  |
| Chau et al. (2021) | The role of social collaborators in the community | *That’s why around my neighborhood if anyone had any problems or got sick they would find me for consultation. So many people feel excited when they see me. I do everything with my heart and kindness. [SC, Quang Nam]* | Social collaborators spoke of the importance of their existing relationships with patients in their home communities |
|  |  | *The advantage is that I am living in the area and loved by people so when I come, people welcome  me very warmly. […] I also tell people that this is a national project that wants to bring health and wellness to the people in both spirit and body, so please allow me to ask some questions for this project. Please tell me so that I can evaluate and send to the senior level, if there is any problem for you in terms of mental health issues, there will be support. People are very honest and happy. [SC, Khanh Hoa]* | This social collaborator spoke of their unique position in the community and how their existing relationships resulted in people being more responsive, encouragingpatients to seek help from their local social collaborators when they were ill, and also suggested that the existing relationships facilitated their ongoing work. This contributed to “the community have[ing] more confidence  in social collaborators, strengthening their position in the community” |
|  |  | *My job is to go to meetings, mobilize self-management. […] I said if she [patient] could not go, I was willing to try to relax with her, leading her to walk with a walking stick. Many times, when she lies down at home, she felt depressed, not wanting to contact anyone. […] So I often came to advocate for and motivate her. […] I calm them down, invite them to be happy, suggest we need to still live, then I ask them some questions. I approach the community and ask for support for the people to overcome their mental illness. I support them with everything, from a piece of cake, or rice. […] I approach and confide with such patients. Seeing me, they are very happy because there is no one to talk to. [SC, Ben Tre]* | The scope of work that social collaborators were responsible for. |
|  |  |  |  |
|  | Challenges for program scalability with social collaborators | *I think young people like you [referring to the interviewer] are very talented. Old person like me cannot be talented like you. In fact, people said that they would like to see the old doctor, because they are  full of experience, but I think there is no experience because the things they have learnt in the past is different from now. Now when I went to big hospital like Thong Nhat Hospital, it is full of talented young  doctors, I found that the youth is talented. [SC, Long An]* | While older retirees were well known and respected in communities, which facilitated their ability to gain  patients’ trust, older age was also associated with difficulties in acquiring the new knowledge required for the interventionThus, while age and the related experience was definitely an advantage in encouraging trust with communities, our study also suggests that scaling up the intervention might require the development of training programs that are adapted for older learners. Conversely, while young people might learn more quickly, they might not connect as well with patients and may need more training and support to establish trusting relationships with people suffering from depression in the community. |
|  |  |  | Thus, while age and the related experience was definitely an advantage in encouraging trust with communities, our study also suggests that scaling up the intervention might require the development of training programs that are adapted for older learners. Conversely, while young people might learn more quickly, they might not connect as well with patients and may need more training and support to establish trusting relationships with people suffering from depression in the community |
| Chibanda (2015) | Intervention | *LHW1 “After working on the bench for a while we just slowly started using quite specific terms to describe  what we were doing and these (terms) just became normal after some time and we all then understood  what was meant when one of us said kuvhura pfungwa (opening the mind) or Kusimudzira (to uplift)….* | LHWs described three overarching concepts or categories that highlighted the steps of the PST intervention and how they linked to each other. These concepts were: Opening of the mind (Kuvhura pfungwa) which mainly  included the introductions, creating rapport, being nonjudgmental, listening and giving a summary of the listed  problems; Uplifting (kusimudzira) discussion of the listed problems and getting the client to decide which problem to focus on; Strengthening (kusimbisa) encouraging the client to stay focused once a problem had been selected, followed by brain storming of solutions and developing an action plan; and finally Strengthening further (kusimbisisa) described as a process of reinforcing achievements made during kusimbisa |
| Chibanda et al. (2017) |  |  |  |
|  | Kuvhura Pfungwa (Opening Up the Mind) | *LHW6 “When am working with a client I have to make sure that the client goes home with something  after the first session because they may not return since our people are very mobile, so I do opening the  mind (kuvhura pfungwa), and this will lead to easier work in kusimudzira (uplifiting).”* | The LHWs mostly agreed that they always aimed to cover the three components of kuvhura pfungwa (opening of the mind), kusimudzira (to uplift) and kusimbisa (to Strengthen) during the first session. Ensuring that the bulk of the work was done in the first session was critical because sometimes clients were unable to come back for subsequent sessions. Furthermore, LHWs felt that waiting for a week before a problem was reviewed was discouraging for clients. The LHWs felt that this first session, unlike as originally conceived (see Table 1), needed greater time and emphasis and those subsequent sessions could be shorter and delivered over 3–4 weeks |
|  |  | *LHW3 “I always start off by greeting and discussing general issues to make the client feel relaxed then I  introduce the issue of their SSQ score being high and suggest to them they may have kufungisisa (thinking  too much)…. Often they break down and cry when you suggest they have kufungisisa….”* | Key ingredients for successfully opening up of the mind (kuvhura pfungwa) included an ability to show empathy,  connecting with the client, being able to reflect and summarize key issues, being non-judgmental and not being prescriptive or instructive |
|  |  | *LHW2 “because I (LHW) also live with HIV when I show understanding (empathy), they are grateful and  when I share my own experience this helps to further open up their mind.”* | Connecting with clients came in several forms from touching the hand and other culturally appropriate parts of the  body, offering a tissue paper to a crying client, and praying. It was not unusual for the LHWs to use their own life  examples to help create rapport |
|  |  | *LHW5 “It’s important to be non-judgmental even when you are talking to a sex  worker and this really leads to (kuvhura pfungwa & kusimudzira).* |  |
|  |  | *LHW1 “So because we are known as Ambuya Utano (Lady health worker) there is a certain level of trust we get from PLWH. We have lived here for more than 20 years so we are known because with most of them we see each other at the market, we have helped them in the past maybe during the cholera outbreak, or when we carried out a home visit to see if they had medication for the HIV illness…”* | Living in the community they worked in seemed to be an added benefit for the LHWs as they felt that this brought  a certain level of respect and trust, making opening of the mind more possible. |
|  |  | *LHW8 “So we discuss and I give a summary of what they tell me (problems)…yes this is all done in the first visit… then I try look at how they used to cope before and what has changed…like what have they stopped doing. See, it can be small things like going to church, or like neighbors don’t talk to me anymore, stopped sweeping the front of the house….”* | Opening of the mind was further described as the stage where the client was now in free flow, revealing their challenges and problems. At this stage the LHWs would sometimes ask about how things were before these challenges arose with a focus on the things that the client used to do that she is not doing anymore due to kufungisisa (thinking too much). |
|  |  | *LHW6 “We have to find one problem to start with and this is the client’s job. So I briefly summarize then I just listen and encourage them to think and select one problem. Sometimes this takes time”.* | The final component of the kuvhura pfungwa was described as the point where the client now had to decide which problem to focus on. It seemed from the LHWs that clients hardly ever presented with one problem and as a result it often was difficult for them to select a single problem to work with |
|  |  | *LHW3 “because we are LHWs they think we have answers and we should tell them which problem to start with, so it can take going forward and backward before they identify one problem on their own and sometimes it can be an unusual one, like someone who is HIV+ and not taking medication once said to me, ‘I want to focus on problem for school fees for my child’”* | The LHWs sometimes felt there was pressure from clients for them to provide solutions. |
|  |  | *LHW3 “So sometimes you start working on the kuvhura pfungwa and you don’t finish it up in one sitting, then you meet the client in the community or at the market place and all I say is – I’m thinking of you  my child and I look forward to our next discussion”LHW1 “You know, sometimes a client will come and  knock on my door (at home) hours after our session and say ‘Ambuya (grandma) I know which problem we have to focus on’”* | The LHWs indicated that the first session would take up to an hour and in some instances it would be longer than an hour or would be carried over to the following day at the clinic or as a home visit. Sessions appeared to be less rigid after the first initial visit. |
|  | Kusimudzira (Uplifting) | *LHW1 “They are uplifted (kusimudzira) when they select a problem to work on even if it’s a small problem this gives them a new focus I think”.* | The Uplifting (kusimudzira) component was described as the part of the therapy where problem listing and selection of a single problem were carried out. |
|  |  | *LHW4 “ Just listening and summarizing what the client presents with helps to open the clients mind (kuvhura pfungwa) which then leads to the client being uplifted and deciding what they want to focus on (Kusimudzira) because they  know they have support”* |  |
|  |  | *LHW7 “Sometimes they focus on things I would not think were important. Once I had a client who  was HIV+and with low CD4 count and a child with kwash (malnutrition) and she had stopped taking her  HIV medication and she listed several problems and decided the most important one to focus on was how  she could find money to go and visit her mother’s grave in the village”.* | The type of problem selected by the client to focus on appeared not to be of primary concern to the LHWs, although at times they were dismayed at the choices that clients made. |
|  |  | *LHW2 “So once this lady who decided after opening the mind and kusimudzira that she would address her  lack of money by growing vegetables and sell them but she needed $5 to buy the seedlings. So we focused  on how she would get $5 dollars to start her business (kusimbisa)”.* | Success depended on the LHWs ability to work with any problem presented to her by a client. |
|  | Kusimbisa (Strengthening) | *LHW2 “So we talk about how they are going to sort out this problem identified during kusimudzira….She  needed $5 to start her business. She came up with several ways including asking her sister, her friend from  the market, offering to sweep the shop floors for a fee….”* | The kusimbisa component appeared to consist of brainstorming, exploring options, with emphasis on facilitating and not instructing. Through this process, a specific plan which include when and how it would be carried out emerged. |
|  |  | *LHW3 “ Yes, identifying a problem after listing is important but what is more important was if the client left the Bench with a plan for that problem, even if it was just a simple plan, like something to do, even to just make a phone call and talk to the church elder and then give feedback on this.”* |  |
|  |  | *LHW3 “Yes, identifying a problem after listing is important but what is more important was if the client  left the Bench with a plan for that problem, even if it was just a simple plan, even to just make a phone call  and talk to the church elder and then give feedback on this.”* | The emphasis on ensuring that clients walked away from the Bench with a tangible solution after the first session was a  recurring theme raised by LHWs |
|  |  | *LHW7 “Agreeing on things to do makes them feel supported (kusimbisa) because they feel you are interested in working with them. Yes, what is important is that we (LHWs) don’t tell them what to do but we provide guidance”* |  |
|  | Kusimbisisa | *LHW3 “I listen to the client when they come back to see me and I encourage them to stay focused and  sometimes I even tell them I will visit them at home to see how it is going.”* | a continuation of kusimbisa, and focused on reinforcing strategies agreed upon in the kusimbisa section |
|  |  |  |  |
|  | Duration of Sessions | *LHW4 “The first visit which is kuvhura pfungwa is important and this feeds into the others (kusimudzira  and kusimbisa), so it takes time to go through about an hour or even more …”* | The LHWs indicated that the first session would take up to an hour and in some instances it would be over an hour. |
|  | Difficult Cases | *LHW4 “When I have a person who is suicidal and is red flag and appears to have thought about how they  would kill themselves and the SSQ response to question 11 is “Yes” I immediately take the client to Ethel  (Supervisor)”.* | The LHWs described three main referral criteria for difficult cases (red flags) which they defined as: (1) suicidal cases;  (2) clients where the problem selected seemed impossible to resolve and (3) any case with an SSQ-14 score of 11 and  above. They indicated that over the years the rate of referral to the supervisor had reduced significantly because they  now felt confident with difficult clients. |
|  |  | *LHW1 “ Sometimes they would be so sad and they talk of ending it all, for me this is an emergency to take to our supervisor”* |  |
|  |  | *LHW6 “ So they come and elect a money related [problem] ….they say money mainly for school fees, their child is two weeks not at school because she has no money for school fees. We use the same approach of opening up the mind  and kusimudzira but in relation to the money for school fees….”* |  |
|  |  | *LHW3 “One woman needed $30 for school fees. After we talked about ways of making $30 she came up  with several solutions like firstly going to the school and asking the headmaster to take her son and giving a written guarantee to the school that she would bring the money in 3 weeks. See this helped her to relax, then we looked at how to find the money and she ended up deciding to borrow $10 dollars from her friend and she went to Norton where the fishermen are and she bought fresh fish for $7 and used the $3 for transport. She sold the fish for $15 and went back to the fishermen two times until she had $ 40, now she goes there regularly…”* | With clients who presented with a problem that is related to finances LHWs indicated that these tended to be difficult but over the years the approach has been to look at the reason for needing the money and focus on that |
|  |  | *LHW4 “I had a woman who lives here in Mbare who came with an SSQ-14 score of ten. So she was also  HIV+ and she was desperate to have regular money to feed her family, her husband had died. We spent close  to an hour doing kusimudzira (selecting and exploring a solution) and then we came up with a list of six  different things she could try. Eventually she tried the money club program where she joined a group where  she gave $1 each week and when it was her turn after 4 weeks she received all the group money”* |  |
| Davis et al. (2022) | Influence of context on therapeutic effectiveness | *Patient's quote* | These factors impeded potential efficacy of the sessions by ‘interrupting’ the improvements in mood or reduction in symptoms through their consistent presence in the women’s lives, and conversely, through the sessions not being able to address some of these socio-economic issues |
|  | Counselling factors affecting quality of delivery | *“Psychoeducation: The counsellor teaches the mother about mental problems by explaining the cause, what to expect and what the solution is. Psychoeducation helps to reduce being looked down upon due to depression. Did you hear? Psychoeducation is depression. Do not be confused when I use them interchangeably” (Csr A, AD, S1).* | certain strategies or behaviours used by the counsellors that did not align with what was intended in the counselling manual. The most common of these across all of the counsellors were: misinterpretation of therapeutic modalities, inadequate explanations of therapeutic activities or of the required homework, inappropriate reading from the manual, minimising problems that participants identified, providing absolute assurances for solving these problems, providing advice, and reprimanding participants |
|  |  |  | some counsellors seemed to misinterpret the therapeutic modalities that they were supposed to have been teaching to participants. This is demonstrated with an example of when a counsellor read an instructional sentence from the manual to a participant (not meant to be read aloud), and then went on to explain the concept to the participant in her own way |
|  |  |  | In addition, the counsellors tended to provide inadequate explanations of how to do the session activities or of the required homework, and sometimes read inappropriately from the manual (as above), when they were expected instead to explain these in their own words. |
|  |  | *“They know at church that I always sing even though I don’t know how to sing. Then they asked me, why am I not singing? And I would be thinking, they have no idea how much pain I am in”. Counsellor: “Okay so you say you like going to groups and singing. You can write your activities in your book. We will talk about them next week. You write the things you like” (Csr A, AF, S3).* | There was also evidence of counsellors minimising problems that the participants raised in the sessions, thereby potentially invalidating the experiences of the mothers. An example of this is shown below where a counsellor did not acknowledge or follow up a participant’s revelation of pain and lack of recognition of her illness at her church |
|  |  | *No, you have had counselling, so you are not going to have a problem. It can affect a baby if you are depressed when you are pregnant. If you talk about your feelings, nothing is going to happen” (Csr E, EA, S4)* | Another strategy that the counsellors used was that of making assurances that after the six counselling sessions, the mothers would feel fully better and that their problems would be solved. One participant asked a counsellor whether ‘feeling sad’ was going to affect her baby |
|  |  | *“[DE], let me give you a piece of advice about men. When you get angry that he spends time or drinks with his friends, the first thing in his mind is that his girlfriend or wife is controlling him. Do you understand? The right medicine for them is [for you] to keep quiet and not ask anything, even if you want to talk” (Csr D, DE, S6).* | Giving advice around how to solve problems or what to do about partners or boyfriends was a particularly common trend amongst counsellors. This is illustrated in the example below when a counsellor said to her participant. Another counsellor demonstrated her feelings about men |
|  |  | *“Now you think that he is cheating when he goes out. I can advise you that if he is buying food at home [for you], just keep quiet. All men are cheating so you need to look after your children” (Csr E, EA, S2)* |  |
|  |  | *“The last session you spoke about your likes and dislikes right? Where is your book? You do not write [your exercises]. I gave an example for you and you did not write. You see? I even said choose here all the way to this side. You see?.. I gave an example and even made brackets for you… You did not write. Write! Write! At all. Are you getting old? … Do not think you have escaped because you still have the book [at home]. I want that book and this one full for Session 5. Do you understand?” (Csr F, FA, S4).* | there were examples of counsellors scolding or reprimanding participants. The following example reveals visible frustration and scolding from a counsellor |
| Henrique et al.(2021) | Capability of non-specialists (Feeling capable of delivering the intervention) | *CHW 1: “Of course! I am sure [that a CHW could deliver the intervention]”; CHW 3: “We were well trained; we can do this [intervention]”.* | In general, the health workers did not experience problems delivering the programme and felt welcomed by the older adult participants. Only one CHW (CHW6) mentioned that the older adult participants she visited, refused to participate in the psychosocial sessions because the CHW was not a mental health specialist |
|  |  | *CHW 6: “There was only one older adult participant that I was seeing who was hesitant and gave up.”* |  |
|  | Training and clinical supervision (Feeling supported and prepared) | *CHW 8: “So, it was a support [for us], because we were afraid of what could happen [in the sessions] … What now? Then, we had the supervision to talk; they [supervisors] were there to help with our questions”.* | Health workers perceived the training received and the group supervision as opportunities to talk about difficulties and support their work. In addition, supervision was also perceived as a space to share experiences among health workers, learn from each other, and discuss difficulties related to the use of the PROACTIVE app during the home sessions. |
|  |  | *NA 2: “And then, in the supervision, everyone’s experiences counted, what they were going through [with the older adult participants] … Hence, I think we could get a little bit of all the experiences, you know?”.* |  |
|  | Team collaboration (Involvement of other members of the Family Health Team in handling cases) | *CHW 4: “I talked to the CHW – ‘Did you notice this and that?’ and she goes like – ‘No, but one day I noticed this other thing and you?’, then we started putting pieces together until we decided to gather with the FHT to have a different look at this older adult participant; there was something different, you know?”* | Health workers perceived their participation in the PROACTIVE programme as a facilitator of the communication with FHT members. One of the results of the collaboration between health workers and FHT’s doctors and nurses was the involvement of these health workers with some older adult participants who were receiving the psychosocial intervention: |
|  |  | *CHW 3: “Yes, the doctors, nurses [were collaborative], they got involved, they referred older adult participants, they followed [the older adult participants] closely; in team A, the doctor went to the older adult participant’s house [for a consultation] and after that the older adult participant came to the UBS for health exams”.* |  |
|  | Impact of the intervention in their work routine at the UBS (Professional impact) | *CHW 8: “Today, even without the tablet and not being part of the intervention programme any longer, we can still offer some counselling to people.”;* | All health workers indicated that participation in the PROACTIVE programme had a positive impact on their work at the UBS, as they acquired new skills. |
|  |  | *CHW 6- “Everything that we learned, that opened our mind, that helped in our work was valid. Every day we learn something, we change our point of view… This [the intervention programme] came to intensify it, to change our view”.* |  |
|  | Impact of the intervention in their work routine at the UBS (Institutional impact) | *CHW 1: “Yes, because it was an extra burden, wasn’t it? Our usual tasks never stopped, but it [the programme] was something, it left a good taste in my mouth…”.  NA 3: “Much more, much more time than we have imagined... But it was good”; CHW 7: “I didn’t know it was going to be that much”.  CHW 4: “There were frictions, you know? Because we did not do other things, we ended up taking that time to do these other things we had not planned… So, there were divergences”.* | The extra work associated with participating in the PROACTIVE programme was a hot topic during the focus groups. The main complaint was related to the difficulty of managing the time to perform the routine tasks at the UBS with psychosocial sessions at home, all within working hours. The home sessions were added to their other tasks at the UBS. They also felt the time spent with the PROACTIVE programme (sessions and supervision) was more than what they expected initially. Health workers also complained about frictions with colleagues over the time they spent on the PROACTIVE programme. |
| Jacobs et al. (2021) | Counsellors’ Perceptions of the Project MIND Training | *“It [project MIND training] was different because mainly at [organisation providing adherence counselling training] you talk about HIV and so, it has nothing to do with a person’s psychological state … this one is psychological all the way because it is a person’s problems. So that is the difference.” (ID06, designated counsellor).* | Counsellors described how the project MIND training supported implementation of task-shared mental health counselling. Training was well received by all FBCs who perceived several benefits in terms of building their professional counselling skills. In particular, designated counsellors highlighted their improved communication skills (such as the use of active listening, open-ended questions, and reflections) and understanding of psychosocial problems. They described applying these to their usual adherence counselling practice, enhancing their counselling relationships and improving counselling quality. |
|  |  | *“If we have a defaulter [patient who stopped taking ARVs] we can ask them what is the reason, why did you not come, what made you feel that way…which I did not do before because we did not go on the [project MIND] training … we did not know about this …” (ID11, designated counsellor).* |  |
|  |  | *“It benefitted me because my husband also drinks [alcohol], so … I have given him my [intervention] book. And then it was very helpful, and he was interested …” (ID01, designated counsellor).* | Most FBCs described how being trained in MI-PST benefitted them personally. After seeing how the problem-solving method helped their patients, some of these counsellors applied the problem-solving method to resolve their own challenges and assist those around them (e.g. problematic alcohol use among family members). |
|  |  | *“I learned so much about role plays so that we can familiarise how the intervention should be like … from session one to session three.” (ID08, dedicated counsellor).* | The FBCs emphasised that the role-playing and skills rehearsal enhanced their confidence in their ability to deliver the MIND intervention. According to these counsellors, the skills rehearsal aspect of training provided them with opportunities to rehearse their new counselling skills while simultaneously familiarising themselves with the manualised nature of the intervention: |
|  |  | *“The hours were too long. I mean too long hours can also put a person to like … get tired.” (ID17, dedicated counsellor).* | Some FBCs described struggling to maintain concentration during training and they were not accustomed to the intensity of each training day (6–7 h per day). These FBCs felt that they would benefit more from fewer hours of training per day and extending the number of days of training |
|  | Counsellors’ Perceptions of Clinical Supervision and Debriefing | *“I think for me supervision is important because you can get help with whatever case or any difficult situations that we are facing and also to know where to work on. It is also nice to hear that, oh, you are doing good or you can work on that or maybe do not use that and so on. So that really, really, really helps a person.” (ID03, designated counsellor).* | All FBCs reported being largely satisfied with the supervision and debriefing they received, emphasizing its role in their professional development and for ensuring quality of counselling. Weekly supervision and debriefing enhanced both their counselling skills and their confidence in their ability to deliver the intervention. They described valuing the opportunity that supervision afforded to discuss patient progress, assess counselling performance, and identify ways of improving their practice. |
|  |  | *“It is very important, because before I used to take on the whole world. Now, I know that as I care for my client, I must care for myself, because if I do not care for myself, I am going to burn out.” (ID18, designated counsellor).* | FBCs also valued the regular debriefing they received as part of their weekly supervision session, particularly in relation to prioritising self-care and preventing burnout. Regular opportunities for debriefing created a “safe space” to discuss difficult patient cases, personal issues that may be impacting on their counselling, issues around self-care, and strategies for coping with stress: |
|  |  | *“We do a bit of role-play also … so that is the one thing that stands out for me … when you put yourself in a client’s position … or you change roles, gives you a bit of an idea of what you must do.” (ID03, designated counsellor).* | When asked, FBCs identified several factors that either supported or hindered the degree to which they could engage and benefit from supervision and debriefing. With regards to the format and delivery of clinical supervision and debriefing, FBCs found the role-playing exercises conducted during supervision particularly beneficial. During these role-plays, the FBC and supervisor would re-enact challenging counsellor-patient interactions. The counsellor would assume the role of the patient and the supervisor the role of the counsellor. These role plays provided an opportunity to reflect on alternative approaches to problems, enabling the FBCs to reflect on past situations and practice managing them differently while preparing them to deal with such scenarios more effectively going forward. |
|  |  | *“He [counsellor supervisor] is always there to support, even via WhatsApp. I can ask him anything, any time he will respond swiftly.” (ID15, dedicated counsellor).* | FBCs described a good working relationship with the counselling supervisor as critical, describing the need for a supervisor that was trustworthy, respectful, and non-judgmental. FBCs also viewed the availability of the supervisor outside of formal supervision times as key for supporting them in their new role. They described “debriefing on-the-go” as key to the success of the programme: |
|  |  | *“If they can have mentoring [supervision and debriefing] where all the counsellors get together and then they debrief … with different cases and how they struggle and how do they cope.” (ID06, designated counsellor).* | FBCs provided suggestions for how the project MIND approach to supervision and debriefing could be strengthened including longer supervision sessions and more time with the supervisor, particularly during the first few weeks of their new role. As one FBC noted, “… when he leaves, I just feel that, oh, I needed one more hour.” (ID04, dedicated counsellor). Some expressed the desire for occasional group supervision and debriefing to help them feel connected and part of a community of practice where they could share their experiences and learn from each other. |
|  | Perceived Barriers and Facilitators to the Implementation of the Project MIND Intervention | *“At the beginning it was fine but as time went by it was such a lot of people we had to see and we had to see our work as well. So, it was tiring, because an hour to sit with a person is a lot when you know my colleagues need to see now maybe 20 or 30 more people because I am busy in this project.” (ID11, designated counsellor).* | Designated counsellors expressed concerns about the degree to which they could align the delivery of mental health counselling with their current workflow. They described competing task demands (including the management of chronic clubs which involves health promotion, provision of HIV testing and counselling, and other administrative duties) and uncertainty about how to reorganise their work schedule to accommodate MIND counselling. Several FBCs felt overwhelmed by these multiple task demands and needed help to prioritise their other work demands so that they could perform their duties optimally. |
|  |  | *“…Sometimes they [nurses] just say, ‘oh, this is the lay counsellor’, you know, you are just [irrelevant]”. (ID18, designated counsellor).* | By contrast, dedicated counsellors did not report the anxiety around competing task demands. Given their primary work task was mental health counselling, they were able to focus on counselling without feeling pressured to perform other tasks. When they were not busy counselling patients, they described offering their assistance to others in the facility such as “going to the reception to help them with the folders” or helping “if there is a doctor or the nurse that needs a translator”. Although more salient for designated FBCs, both types of FBCs explained how their role was assigned a low status within the healthcare facilities. Counsellors perceived their lack of status and authority as hindering their efforts to negotiate for private and a confidential space within which to deliver counselling. This was evident in the many disruptions that would occur during the counselling sessions when healthcare staff would often enter the room with a query or request. Both designated and dedicated FBCs felt that these interruptions reflected the low value that other health workers placed on their work, which contributed to feelings of marginalisation. |
|  |  | *“…we do not want to sound like [we] always wants recognition in the form of finance, but that would also help. Because I mean, your load is increasing, your targets are increasing, but money wise it is not increasing.” (ID13, designated counsellor).* | For a subgroup of FBCs, these feelings of being undervalued were reinforced by low levels of remuneration. In South Africa, FBCs are paid a stipend that is just above minimum wage. According to these FBCs, low remuneration decreased their motivation to provide the counselling service. Designated counsellors were particularly frustrated as their scope of work had increased without any adjustment to their remuneration. Many described situations where they felt that tasks were being ‘dumped’ on them without any recognition or reward for the work they do. |
| Jacobs (2020) | Counsellors’ perceptions of the benefits of the project MIND training | *“…the role play really, really helps a lot. It is good to understand content, but role play that is what matters when you do this counselling intervention.” (ID15, dedicated counsellor)* | Role-playing stood out as one of the key training aspects that most FBCs thought was beneficial for assessing their new counselling skills and their readiness to implement them into practice. It provided them with opportunities to rehearse their new counselling skills while simultaneously familiarising themselves with the manualised nature of the intervention. |
|  | Barriers and facilitators related to the implementation of the project MIND intervention > intervention characteristics | *“…It is programmed very well. It is very helpful, but it is too long.” (ID01, designated counsellor)* | With regards to the perceived complexity of the intervention, a barrier to implementing the project MIND intervention that was highlighted by most designated counsellors related to the length of the counselling sessions. Compared to their adherence counselling sessions, they felt that the project MIND counselling sessions were too long despite the benefits. These FBCs felt concerned about the many other adherence patients waiting to be seen while they were spending up to an hour counselling one of the project MIND patients |
|  |  | *“There is some of them…they do not understand because some of the Afrikaans words is just...too formal…” (ID03, designated counsellor)* | These FBCs indicated that the language and grammar was too formal compared to the local spoken languages in the communities. Some patients therefore struggled to understand some of the information initially. As a result, and contrary to expectation, many patients whose first language was isiXhosa for instance, requested to have their sessions in English as it was easier to understand. |
|  |  | *“…I will find with that some of the youth, they will say “no man, we want English… Xhosa [language] is too long for us”. (ID07, dedicated counsellor* |  |
|  |  | *“Some of them, they do not have time, to do homework in their home because of some many things like, there are -maybe, the funerals, or they are busy with their own things.” (ID05, designated counsellor)* | half of the group (both dedicated and designated counsellors) indicated that many of their patients struggled to complete the take-home activity part of the intervention and that it could be simplified further. According to this group of FBCs, these patients were challenged by poor literacy and time constraints |
|  |  | *“Most of them to be honest do not do their homework…because I have picked up that –some of our patients, like I said most of them is illiterate…” (ID15, dedicated counsellor)* |  |
|  |  | *“…On the first session yes, they do [participate] but not much, but on the second session they have got stories…” (ID01,designated counsellor)* | a few FBCs described how some patients appeared withdrawn and hesitant during the first session, which they ascribed to being the patients’ first counselling experience. However, the FBCs reported that this changed with patients engaging more as counselling progressed |
|  |  | *“It [project MIND] is a good intervention because people do not know always how to handle situations…they do not listen so when they come here to the intervention they learn how to listen and think about the negativity… how that situation will impact them negatively and positively.” (ID03, designated counsellor)* | These FBCs reported that the project MIND intervention enabled them to empower patients by guiding them through a process of problem-solving using their existing problems. This enabled patients to return home and implement their problemsolving strategies. By working with patients from different backgrounds and having the opportunity to relate to them over time, counsellors reported observing how the counselling sessions benefitted their patients |
|  |  | *“There is another client that I met, what, on Sunday? And then she saw me I was not at work. She says, ‘you know what; I have a good relationship with my daughter, and I have a good relationship with my mother, but my mother just died. But before she died, I feel that we just had a good time’… because she was not talking to her mother or the daughter, because of some family problems. But once she came to project MIND, she was able to resolve it in three weeks. So, it is good.” (ID06, designated counsellor)* |  |
|  | Barriers and facilitators related to the implementation of the project MIND intervention > Inner setting | *“…say for example we are three and the one is being booked for Project MIND, the pressure is on the other two to work for that one.” (ID13, designated counsellor)* | Designated counsellors expressed competing task demands (misalignment) as a barrier to implementing the project MIND intervention. These FBCs described having to oversee several chronic clubs, perform HIV testing and counselling, and complete their administration duties as some of their core duties. As delivering the project MIND intervention was an addition to their daily  duties, these FBCs felt that the task demands were often too much to perform all their duties optimally. |
|  |  | *“…On Wednesdays I am busy with mental illness [psychiatric clinic] with the psychiatrist and doctor…whereby I do not focus on Project MIND alone, but I also participate and assist also on the psychiatrist and doctor that is here.” (ID08, dedicated counsellor)* | Dedicated counsellors reported enjoying their work and being able to focus on delivering counselling without feeling pressured to perform other tasks (alignment). During times when they were not busy counselling patients, they offered their assistance to other areas in the facility such as helping healthcare staff or administrative personnel |
|  |  | *“As long as I do not have any clients, I go to the reception to help them with the folders.” and “…if there is the doctor or the nurse that need a translator, then I translate for them.” (ID14, dedicated counsellor)* |  |
|  |  | *“Sometimes I feel like…I do not know whether I am not accepted. I do not know. I do not know how can I put it, because sometimes I am doing counselling saying, “Eish [Sigh], I am not happy, [after being asked for the space by nurses].” (ID05, designated counsellor)* | Generally, the FBCs started working as home-based carers or community care workers and afterwards attained a training certificate to work as FBCs for NGO’s. This lowly status impacted their ability to negotiate space or advocate for privacy to deliver the project MIND counselling. Therefore, the FBCs felt that they were disrespected and treated as outsiders by some of the healthcare staff in the facilities. They also felt marginalised in the facility with no credence given to the important work they performed. |
|  |  | *“It is the space that we do not have, and you find that sometimes the nurses are demanding the room.” (ID05, designated counsellor)* | the inner setting construct of readiness for implementation, in the form of lack of resources, was described as a barrier to implementing the project MIND intervention into PHC facilities. For instance, most of the FBCs identified a lack of space as one of the main resource constraints. On several occasions, when counselling sessions were due, FBCs had to search for a room to deliver  the counselling. Moreover, there were certain days in the week where no counselling could take place due to additional healthcare staff such as optometrists and psychiatrists visiting the facility and occupying the rooms. |
|  |  | *“…on Thursdays we normally do not have sessions…what happens is there is an eye clinic…They visit this side, yes. So that makes it a little bit hard for us to work.” (ID17, dedicated counsellor)* |  |
|  |  |  |  |
|  |  |  |  |
|  |  | *“I do not have my privacy with my patients. So, there is that in and out happening.” (ID05, designated counsellor)* | the majority of FBCs identified culture (norms, values, and basic assumptions of a given organization) as a barrier. Counselling requires that privacy and confidentiality be maintained at all times which is in line with basic principles of client-centred care (Lamont-Mills, Christensen, & Moses, 2018). A lack of privacy and confidentiality was described by many FBCs as a challenge to providing client-centred counselling in these settings. During counselling sessions, healthcare staff would often enter the room to collect facility-related supplies such as files or medical supplies. During these interruptions, the counsellors had no choice but to pause the session in order to accommodate the staff. Despite designated counsellors having their own office in the facility compared to dedicated counsellors, counselling interruptions were consistent for both approaches. Counsellors felt that this interruption of the counselling sessions might have had an impact on the patient’s willingness to disclose personal information and fully engage in the counselling process. Moreover, not recognizing and prioritizing patients’ need for privacy by healthcare staff demonstrated the cross-cutting impactthis had on the inner setting context (interruption of counselling sessions) |
|  |  | *…when you are in the session and now the patient is like, does not want to open up. And the patient has opened-up before but now you have so many distractions. Everyone is entering the room…there is people sitting on the side, so you can see, it is not that the patient does not want to open-up to you. There are too many distractions.” (ID18, designated counsellor)* |  |
|  | Perceived benefits of supervision and debriefing | *“…we do a bit role-play also…so that is the one thing that stands out for me…when you put yourself maybe in a client’s position…or you change roles, so that gives you a bit of an idea of what you must do.” (ID03, designated counsellor)* | The majority of FBCs found the role-playing exercises conducted during supervision as particularly beneficial. The FBCs indicated that at times they would get stuck during a counselling session while not knowing what to say or how to proceed. However, during supervision the counsellors would assume the role of the patient and role-play challenging counselling scenarios with the counsellor  supervisor. This facilitated experiential learning. By means of role-playing such scenarios, it enabled the FBCs to reflect on past situations and practice managing them differently while simultaneously preparing them to deal with such scenarios more effectively thereafter. This also enabled the FBCs to, in a figurative sense, walk in the shoes of their patients to gain an insider’s perspective from their patients. |
|  |  | *“The supervisor will listen to the recording and then he comes every week for supervision. So at least he will tell me this, this and this we must improve there and there. We did well here, very good, this and this. So, the supervision helps me…” (ID06, designated counsellor)* | Initially, all FBCs were anxious about their sessions being audio recorded for supervision purposes. They indicated that this supervision approach was completely novel with none of them having any previous experience of this form of intensive clinical supervision. At first, counsellors felt that it would be used to check on their mistakes and for criticising their work. Despite some counsellors being anxious initially, the majority of counsellors expressed positive views about the counselling sessions being audio recorded to assess intervention fidelity and receiving feedback as part of supervision. After completing their first few counselling sessions and receiving fidelity feedback, most of the counsellors were able to adjust to this method of receiving feedback. Among the positive views, counsellors expressed that it helped them to identify the areas where they needed improvement and it motivated them to deliver the intervention to the best of their ability. |
| Joag et al. (2020) | Acceptability and feasibility of intervention components (Counselling) | *One-person (AB) … AB got married but the partner (left AB and) went back (to own) home the next day … AB’s partner refused to come back … AB was in great tension. I told AB, “Don’t think so much. If you think so much then it is you who will be stressed. When people start thinking they think big (magnify problems)”. I told AB, “You have a brother, a sister in law, take their support. Put your mind on your work.” … soon AB was better. Now AB says that AB will marry again. (Champ60224M03)* | Champions trained to deliver 4 to 6 sessions, depending upon need and as agreed between the Champion and the beneficiary to deliver behavioral activation, problem solving and training in relaxation exercises. The details of 6 sessions is attached as supplementary material. Champions were trained on symptoms of distress and mental health issues and to distinguish between common and severe mental disorders. Champions identified people with CMD through symptoms. About 17.5% percent of the respondents in the population survey self-reported that they received the intervention from the counsellors (Table 3). This self-report may be an under-estimate due to recall issues and counselling sessions happening in informal settings, so beneficiaries not perceiving them as counselling sessions. The qualitative interviews highlighted the adverse consequences of these sessions; that could occur if the problematic behavior or thought pattern continued; and included suggestions of how to change these. |
|  |  | *A patient’s (DB) partner was staying at parent’s house, and later married DB’s younger sibling. Since then DB’s partner had stopped paying attention to DB. It had affected DB’s mental health and DB was suffering from depression. If asked, DB would complain of headaches. I spoke to DB’s partner, DB’s mother and children. I told DB’s children to give special attention to DB and to take extra care, and make DB feel wanted, I said “This will help your parent to get well soon and back to everyday routine”. Earlier DB had stopped taking care of self, had stopped combing hair, taking a bath. But now DB is fine and back to normal. Champ60222M02* | Champions also counselled families of affected individuals on how they could change their own behaviors and help one another |
|  | Social benefits | *I had told one widow … She was in tension, (she said) “How to manage two daughters and one son?” Then I informed her “There is a widow pension scheme and you can apply”. (And) I told a person. he is now handicapped. I told him to gather all the documents like his Aadhar card (Government identity number), (told him) “Get yourself a handicap certificate and then you will get some money”. He submitted (the documents). (Champ60224M03)* | 276 families were assisted by the Champions to apply for social benefits in the state of Maharashtra. Social benefits included government schemes for vulnerable and marginalized sub-populations. For example, Champions facilitated following social benefits: Sanjay Gandhi Niradhar Scheme, Integrated Watershed Management Program, Life Insurance for people with disability, Mahatma Gandhi Rural Employment Scheme |
|  | Acceptability of champions | *Everybody has respect for me in the village … after (showing) the films, taking patients, people recognize me even better. When they (people who received Atmiyata) felt better … they told others about how they went to the hospital, got the medicines and are now feeling better etc., then the other person would say “Even I can’t sleep, I also experience numbness”. They used to come and say to me, “Do something for me also.” (Then) I spoke to them. (Champ60224M01)* | Champions were well known in the community, and their strong presence facilitated their work. People felt comfortable talking to them, trusted them and appreciated their work. |
|  |  | *(A patient’s wife) She says “Because of you, my life has become very happy. My husband used to sit on the cot and was always in tension, would forget about his work but from the day you spoke, he is very free.” I feel happy to hear this. (Champ60224M03)* |  |
|  |  | *People used to ask us “Are you doing this job because of payment? “I explained to them that we don’t get any payment. We have to do social work, volunteer ourselves for good work. The aim is how people can live happily, healthier mentally and physically as well. One person asked, “If you don’t have any profit why do you work here?” I told (that person) “I like it, that is why I do (it)”. (Champ60223M03)* | Champions enjoyed their work and found it fulfilling to help others. This made them willing to work voluntarily, without pay. |
|  |  | *I feel good when I do something for someone. Because of me if someone’s life improves, I feel happy. (Champ60224M11)* |  |
|  |  | *We started working in the village. But since the topic is sensitive, we did not tell the village that we were here for this particular project. During village visits, based on the symptoms told to us, we started identifying people with mental stress problems. (Champ60223M03)* | Some champions were cautious in how they approached people and attempted to earn the trust of others before helping them, often employing creative tactics. They were sensitive to the needs of others, and respected community norms. |
|  |  | *In the beginning I did not show the films directly. I first won their heart and made a place in their heart. I made them understand what qualities I have and only then I showed them the film. (Champ60224M04)* |  |
|  |  | *We used normal words and explained things while chatting. We did not make them (people) feel like they were mental, and we were treating them … If anyone from the patient’s family asked me why I had come, I used to tell them that I had come just to chat. While chatting, we used to get to the topic, and I would make them talk about the problem and try to explain to them. (Champ60222M02)* |  |
|  | Impact and benefits of the intervention | *In one family, the husband used to beat his wife, when I came to know about them, I visited them. I counselled them. Showed them the films on quarrels, addiction. Now fights between them have reduced to a large extent. (Champ60223M05)* | The qualitative in-depth interviews indicated that persons in the community experienced reduced symptoms and improved social, occupational and family functioning for a variety of problems such as depression, domestic violence, alcohol use, and severe mental illness. Employment, and family functioning also improved. These were attributed to a combination of intervention components |
|  |  | *I used to be very irritated. But now it is over. (Now) I take a deep breath. (Champ60224M03)* |  |
|  |  | *I have prevented many widows and separated women from suicidal thoughts. I myself had such thoughts but I overcame them. (I would think) “How can I do such things? I am the Champion of the project!... people will say “She used to counsel us and now she herself had tried this!”. These thoughts changed my mind-set. Now (I) am fearless and I feel confident. (Champ60224M11)* |  |
|  | Challenges | *(I) took him (a patient) to Civil, got medication for him. But he did not take the medicines … he was the same … in his trance. People in our area are mainly superstitious. They don’t have much faith in medication. We all (project staff) tried our best. His family members were not convinced. The vehicle was available, still they were not ready (to take him again to Civil). (Champ60224M10)* | Some community members had low awareness about mental health problems or expressed scepticism regarding the Champions’ work and their motives and resisted the intervention. |
|  |  | *She (a person) has left her husband and is staying alone, so I spoke to her brother about it and he told me, “What do have you to do with this (matter)?. She will look after herself!”. I tried telling the parents, but nothing happened. (Champ60224M01)* |  |
|  |  | *For just one paper I had to go many times. Every time it’s not possible for everyone. Money, time … all goes waste. (Champ60222M06)* | Some families of Champions objected to them working in Atmiyata as it was time consuming and there was no remuneration given for efforts. However, many changed their minds once they came to know about the importance of the work. Family members frequently helped the Champions, for example, in approaching persons in the community, or handling the mobile phone. Champions complained of the long and laborious application process and the bureaucracy involved in getting access to social benefits. |
|  |  | *There is only one lady (whose husband has left her). She is young still. I sent her to the Talalthi to get information on the various programs. I also gave her in writing the documents needed. The Talathi asked for the death certificate of her husband. But he is not dead. She is a destitute … but the Talathi is not listening. (Champ60224M04)* |  |
|  |  |  | Some Champions were also not able to help those with very severe symptoms of mental health problems. Other barriers included minor problems with using films (the voice quality was low, the screens of the phones were too small for proper display, or there were difficulties transferring films to other mobiles); the local hospital not having the necessary medication, the hospital being far away, which affected follow up; and some Mitras not having time to carry out their responsibilities.  There are challenges related to Implementation and health systems. Implementation related challenges included the following: 1. Champions found it difficult to attend training, which was addressed by arranging training venue, which was centrally located, arranging transport to training venue and planning dates by consensus. 2. Champions motivation was a challenge and we used few mitigation strategies as discussed above. 3 Champions had some difficulty using the app and smartphone which was addressed by ensuring that CFs provided continued support and during refresher sessions. |
| Kidia et al. (2024) | Clinical workflow and emphasis on documentation | *We want [the patient’s] thoughts, coming from within. So sometimes a person might say their problem and solve it themselves. But because they did not have the knowledge or they did not think about it like that, [Friendship Bench] might help them open up. (VHW, female)* | In terms of the counselling process, there was a disconnect between VHWs and patients. Some patients indicated that VHWs often provided prescriptive counselling while many VHWs said they focused on eliciting client problems and helped clients come up with their own solutions, rather than being prescriptive — a process they described as a cornerstone of their training. |
|  |  | *Wherever [the children] go, they would not return. So, I talked to her and treated her mind. I opened up her mind and said, “when you faced this problem did you think about telling your relatives?” (VHW, female)* | Therefore, the content of most counselling sessions was on navigating patients’ life problems. A VHW, for example, told the story of a widowed patient whose children kept running away from home |
|  |  | *If I have a red flag, I cannot counsel the client. The pressure might be too much for me, so I need help from the nurses who can send the client to the hospital (VHW, female).* | For nurses, when asked about their supervisory role of VHWs, many mentioned that this role included close monitoring to ensure VHWs are correctly implementing problem solving therapy and taking care of referrals of cases deemed too challenging for the VHWs. |
|  |  | *At the bench, I explain to my client why I think they are depressed and I ask them if they have other challenges/problems other than the ones I would have asked. I then take my notebook and write down the client’s problems. And then I will go through the problems with the clients. I then ask her which of the problems causes her to be more depressed. They tell me and I write the problem on the FB card. I would have written down important information about the client already, like the date, client’s phone number, and my name. (VHW, female)* | Both nurses and VHWs, however, emphasized medical documentation. Nurses felt that making sure that VHWs kept accurate and up-to-date records (via the Friendship Bench cards) was a priority and was the main way to supervise VHWs, rather than directly observing them. |
|  |  | *The emphasis on recordkeeping was even manifest in the tools VHWs requested to perform their duties. Among the many things that VHWs asked for, such as bicycles or money for cell phone service, they consistently requested office supplies to be able to optimize their recordkeeping: We need pens, markers, things to write on. (VHW, female)* |  |
|  |  | *What is left is the issue of making sure [they buy us] stationery when it is finished, so that we always have stationery. (VHW, male)* |  |
|  |  | *What I like most about my work is that lets say a client comes to the Bench and I have all my stationary. Then I know I am ready to work since I have to write down stuff as part of my work. (VHW, female)* |  |
|  | Accessibility > Barriers | *What I find difficult is the issue of the transport system … (VHW, female)* | Both providers and service users felt the intervention was not easily accessible. They perceived that this was due largely to the practicalities of rural life. Many VHWs and service users bemoaned transportation issues and difficulty traversing a challenging terrain, often on foot, and sometimes in extreme weather. This was exacerbated by a lack of money for transport via privately owned taxis known as kombis or commuters. |
|  |  | *Those who trained us had said when we’ll get … uniforms, bicycles, $10 transport. And now we are in the field and transport allowance is $2 and I use $2.50 a day. (VHW, male)* |  |
|  | Accessibility > Facilitators | *So, if I have a bicycle, I can tell the person where we can meet or go to their home. (VHW, female)* | Several VHWs asked for bicycles as a possible solution to this difficulty and many advocated for taking care of patients in the community (either in or closer to their homes) as a much more realistic alternative to having patients come to the clinic for care and as a solution for the significant loss to follow up after the first session of Friendship Bench. |
|  |  | *There are people who are too far still and I don’t know how some of these people could be reached. It could be done in a way that we go there [to patients' homes] for outreach … (VHW, female).* |  |
|  | Feasibility > barriers | *Yesterday it was raining, you will be wet and you start to talk to the client but you are dripping water. (VHW, female)* | Resource limitations in terms of protective clothing, office supplies, and transport made the intervention challenging in ways beyond accessibility for providers and patients alike. |
|  |  | *We need raincoats in case it rains so that I can come to the clinic or cover myself. (VHW, female)* |  |
|  |  | *And also about airtime. Say someone got counselling then they return home, if something else comes up and [they] want to ask the counsellor about it, they will need airtime to communicate (VHW, female).* |  |
| Munodawafa et al. 2017 | Implementation | *The reason I wanted to be a counsellor is because Ilike to communicate with people, I like to help people. (Counsellor 6)* | All six of the counsellors were motivated to becomecounsellors because they were empathic and altruistic. All counsellors felt that positive feedback from participants further motivated them. |
|  |  | *I did not believe in myself that I would be able to do the job, but because of the support we received from our supervisor and the counselling I did previously I was built from that… I felt really small especially because I was not educated, but experienced… now I am very competent... (Counsellor 1)* | Half the counsellors felt that they were confident enough to begin counselling after they received their initial training while the other half felt that they had grown as counsellors due to practice and supervision |
|  |  | *I enjoyed session two because there were lots of twists and turns there. The mother would share her first problem and you stop her so we can talk about that problem. You ask her what she has to say about her problem. Sometimes she would leave without giving you a solution, but next time she returns with a solution…The fourth session also helped them a lot. They thought of how to move on with their lives. (Counsellor 1)* | All the counsellors had good recall of the sessions and reported that since the content of the intervention was educational and beneficial to them and the participants, it made it easier to conduct the sessions. The counsellors enjoyed and thought the most helpful sessions were session two (problem solving) and session four (healthy thinking) |
|  |  | *We could not change [the sessions]. We had to do them as they were outlined because if you changed it you would make mistakes. [The manual] provided guidance… (Counsellor 5)* | All the counsellors felt that using the local language (isiXhosa) made conducting the sessions easier since participants could understand better. They also all felt that the manual based style of delivery made the sessions easier and they understood the importance of adhering to the manual |
|  |  | *As they start witnessing the beauty of your work they become more receptive. We met with the nurses and introduced ourselves to them. Thereafter we worked together and respected each other. If they needed the room we would sacrifice and conduct the session outside. (Counsellor 1)* | Counsellors indicated that having respect and professionalism made it easier to work with the nurses in the clinics. |
|  |  | *I was worried if I would be able to follow the manual and not add my own stuff. I was worried if I would be able to help the client with the problem they had, but at the end I was able to do so much so that others would tell me which sessions they enjoyed. (Counsellor 2)* | Barriers to implementation of the intervention included counsellor anxiety based on fear of deviating from the manual and stress of providing emotional support. In some cases counsellor fears were attributed to how the real life situation differed from the role plays used during training. |
|  |  | *I followed the format of the sessions, but sometimes I would find that the mother does not understand. I would need to explain* | Half the counsellors admitted to straying from the manual in order to help the participant understand some concepts outlined in the manual when needed. |
|  |  | *When I started doing counselling sessions I wished the supervisor would not be there in the room. It is your first time and the supervisor sitting there, writing down, you feel like you are making a mistake and not doing the right thing…. (Counsellor 3)* | All counsellors reported feeling anxious at the beginning. However three counsellors (1, 2 and 4) felt they lacked confidence to begin counselling due to inadequate training while the other three counsellors (3, 5 and 6) felt confident to begin counselling due to an adequate training period. The counsellors’ anxiety could have been exacerbated by the supervisor’s observation of sessions |
|  |  | *We forgot to take care of ourselves as counsellors. We put a lot of focus on the clients. We have our own problems and need to heal first before we help other people... (Counsellor 4)* | Three counsellors mentioned how providing emotional support to mothers reminded them of their own problems and the other three mentioned how counselling participants was emotionally taxing for them. |
|  | Mechanisms | *A person would know that she has a problem, but not know the major problem. This session taught them to list their problems and know which problem is major. (Counsellor 2)* | Counsellors felt that teaching “healthy thinking” and “problem solving” skills changed participant’s behavior. |
|  |  | *Older people know what they want. That makes it easy to work with older people than young people… A young person attends sessions once or twice and when she feels that she is fine she stops. (Counsellor 2)* | Counsellors also felt that older people were easier to work with compared to younger ones. |
|  | Context | *Finding a venue at the clinics was hard. Sometimes we ended up doing sessions outside and that made other mothers not attend sessions because she viewed it as unimportant because you cannot talk about something serious outside. (Counsellor 2)* | The counselors reported facing challenges negotiating the clinic environment. When asked in the interviews to retrospectively rate the clinics in terms of being welcome, (0 being not welcome and 10 welcome), the majority of the counsellors gave Clinic A ratings which ranged from 0 to 5, indicating that they did not feel welcome at the clinic when the project started, while clinic B was rated 10 by all the counsellors, showing that the counsellors felt welcome |
|  |  | *A mother would give birth and there would be nothing at home…She would phone me, “Sister I am at the hospital and my baby does not have nappies or vests or anything.” And I did not have anything myself and felt bad because I said I would be her counselor, but I am going to fail at supporting her in that way. (Counsellor 3)* | All the counsellors indicated that working with participants who needed material assistance was emotionally challenging and made them feel helpless |
|  |  | *I remember I was going to visit the mother after she gave birth…On the day that I was planning to visit her, we saw skollies (thugs) when we were on the station and had to turn back. (Counsellor 3)* | All the counsellors indicated that crime in the community made it dangerous to conduct home visits as they were worried about being robbed. |
|  |  | *Most of the problems they had were family feuds and with fathers of the children. A person would have problems because of the unexpected pregnancy. She would be confused and not know if she should keep the baby or not; she does not know if she will be a good mother or not… (Counsellor 5)* | At times participants were experiencing difficult circumstances in their lives which made regular attendance and behavior change difficult. |
|  |  | *…Some started, but dropped out after session 1. I think that maybe she thinks or feels that the information she shared with me is too much and cannot face me the following day. ” (Counsellor 3)* | The counsellors also felt that some participants dropped out because they felt ashamed for sharing sensitive information. |
|  |  |  | All the counsellors felt that reasons for nonengagement included: i) participants moving from the initial address given at recruitment and not leaving new contact details; ii) participants’ phones getting stolen; and iii) participants living in dangerous or inaccessible areas which resulted in loss of contact. The counsellors indicated that they employed various strategies which were effective in following up participants, such as visiting the participant’s last known address and speaking to neighbours, checking clinic records for updated details and being patient and persistent with following up their participants when they rescheduled appointments. |
| Munodawafa (2018) |  |  |  |
| Musyimi et al. (2019) | Utilizing TBAs’ Patient Rapport and Counseling Experience | *“We counsel people with mental illness and those with many thoughts… we sit them down, chat with them to identify their problem. We then give them talk therapy and tell them how they can lead normal lives.”* | TBAs could come in handy to offer the required support especially in solving family conflicts. This is important in maintaining good mental health during perinatal period and reducing the risk of maternal depression and possibly developmental disorders in the offspring. The involvement of TBAs in identification and addressing psychosocial stressors among mothers was highlighted in the discussions. This was viewed as crucial in improving the mental wellbeing of the mothers during the prenatal period |
|  |  | *After counseling her, we go to that family and talk to those people close to her….”* |  |
|  | Recognition and Appreciation of TBAs by the Healthcare System. | *“There are no records (at the hospital) when we refer mothers to hospitals to show how active we have been in community activities.”* | The study participants were asked to describe the relationship between the TBAs and the ministry of health and pointed out that TBAs are not accorded the recognition they deserve by the healthcare system. They identified the lack of documentation of their community involvement in health-related activities such as the referral of pregnant mothers. An elderly TBA (65 years) firmly explained, |
|  |  | *“We will feel appreciated by having a structured group of TBAs where we can share, initiate something jointly and perhaps receive regular updates on upcoming community activities.”* | The participants also stated that there are no regular meetings or forums for sharing knowledge or receiving updated information. An interview with a key informant in her mid-fifties revealed how the TBAs would feel appreciated if a recognized opportunity for having structured groups could be availed: |
|  |  | *“It’s just terrible. We are sidelined as TBAs. We need identification cards to identify us when we take the pregnant mothers to the hospital.”* | The TBAs also said that they do not have any form of identification when they take the pregnant mothers to the hospital and felt a need to be recognized during the referral process. A 62-year-old TBA states, |
|  |  |  | Healthcare workers (as described by a female nurse in her late fifties) also acknowledged the lack of appreciation for the TBAs and suggested the need to enhance this by promptly offering assistance to TBAs whenever they escort a patient to the hospital, by viewing them as coworkers and respectfully offering culturally acceptable greetings, promptly guiding and assisting them. |
|  | Training and Collaboration of TBAs with Healthcare Workers | *“If possible, a TBA should be taught how to handle these people with mental disorders. This is because a TBA is the “doctor” at the grassroots. No matter how a healthcare worker is skilled he/she cannot get time to go to the village. But a TBA can meet with the woman on a daily basis if need arises as they live in her neighborhood. This can be good because this problem will be treated very fast before it becomes full-blown.”* | TBAs are accepted as “grass root doctors” trusted by the community members. The community appreciates that the healthcare practitioners may not be in a position to offer the home- based interventions due to their busy schedules as opposed to the TBAs. If well trained, the TBAs could identify the patients’ psychosocial stressors that contribute to symptoms at an early stage and address them before they become full-blown. In a FGD, a 37-year-old TBA described, |
|  |  | *“TBAs can be trained that being a TBA is not an independent unit but it’s a multidisciplinary service for many other professions.” (45-year-old TBA)* | Training the TBAs can help to recognize the importance of team work and enable them become part of an effective healthcare system. This will make them appreciate other health related issues and foster a good relationship with other healthcare practitioners. TBAs’ training on mental health and inclusivity in the service provision could also motivate and increase their zeal in the service to the community as explained by the TBAs. |
|  |  | *“If we can be trained on mental disorders, we can feel motivated….” (62-year-old TBA)* |  |
| Myers et al (2019) | Confidence in ability to deliver mental health counselling | *The training should be about five days. Five days gives you enough time to role play and ask questions and grasp everything … The role play, there was not actually proper time for that. In the past I was running out of options because they [patients] were depressed … I learned through this intervention to give the participant space to open up.* | Both dedicated and designated CHWs had limited experience in delivering psychological counselling; this programme represented an expansion of their scope of work. They described the intervention as ‘something new’ and ‘different from the counselling we did’. As this was a shift in practice, they had some concerns about whether the training protocols adequately prepared them for intervention delivery. Most CHWs thought additional time was needed to rehearse the content of the sessions and to build their confidence and competencymost CHWs reported initially being a ‘bit scared because it was something new’, they still viewed their involvement with the programme as an opportunity to learn how to work more effectively with their patients and ‘to develop their skills’ |
|  | Perceived benefits of the intervention | *I saw that each session is making a difference to other people. And to see that people are coming again to finish their sessions … it’s telling me the intervention is making a difference to people (designated CHW)* | All CHWs viewed the intervention as acceptable and beneficial to patients with chronic disease. Dedicated and designated CHWs emphasised how ‘there is really a need for this intervention’ and that ‘a lot of patients here can benefit.’ The acceptability of the programme to patients seemed high, with CHWs commenting on how well patients engaged with the intervention. As one dedicated CHW described, ‘It seems like when I’m doing session one, they can’t wait for session two to give me feedback’. All CHWs were able to provide concrete examples of how patients applied the problem-solving skills they had learnt to resolve problems. They also observed positive changes in patients, which was personally rewarding |
|  |  | *I put it as part of my daily work … when I am busy with a session with another patient, then I also try to bring in Project Mind problem solving. It helps me understand where my patient is coming from. (designated CHW)* | Nonetheless, some CHWs felt that patients could benefit from more sessions for there to be ‘a difference in a person’s life.’ Some CHWs suggested offering an additional counselling session to review the patient’s progress in reaching their goals. Not only were there perceived benefits for patients who received this intervention, but other patients also seemed to benefit from the designated CHWs’ enhanced counselling skills. Several designated CHWs noted how they now routinely used their new counselling skills in their interactions with other patients |
|  |  | *I used to worry a lot. when I was doing the intervention then I realised that there are things we worry about that are not important. … so it seems like I am helping someone else, and I am also helping myself. (dedicated CHW)* | In addition, both the dedicated and designated CHWs appeared to benefit personally from this programme, describing how they now applied PST strategies to resolve problems in their own lives and limit negative thoughts: |
|  | Lessons for future implementation | *There was some miscommunication because they [NGO] didn’t have a lot of understanding as to what was expected from us. So for future, it would be nice if the managers could be in one of the training sessions just to know what it is all about … to avoid confusion as to the time spent with patients. (designated CHW)* | According to CHWs, support from the PHC facility, other CHWs and the NGOs that employed them (where applicable) was critical for facilitating counselling delivery. In facilities where the designated and dedicated CHWs described facility staff as ‘supportive’ and ‘interested’, the counselling proceeded smoothly. However, in PHC facilities where staff seemed less interested, CHWs were often interrupted during counselling, impacting on the therapeutic alliance. For these facilities, CHWs recommended that facility managers and staff are thoroughly informed about the initiative so that ‘they know what it is all about so when you are busy with a client they just give you some time to do it.’  For designated CHWs, competing priorities and limited time were barriers to counselling delivery. These CHWs felt they were ‘doing three jobs’, with almost all mentioning that it would be easier to deliver the programme if all they did ‘was concentrate on the intervention.’ This was not an issue for dedicated CHWs who had more time available to conduct the counselling. Where fellow CHWs assisted the designated CHW with their usual HIV care responsibilities, thereby freeing up some of the designated CHW’s time, the CHW felt more empowered to deliver the intervention. This realignment of the responsibilities of the CHW team to accommodate mental health counselling happened organically at some (but not all) of the designated sites. To facilitate this realignment in the future, designated CHWs thought it would be helpful for NGO employers and supervisors to be more involved in training and discussions about the implementation of the intervention, so that they ‘have a better understanding’ about what is required from their staff: |
| Ng'oma et al. (2024) | Perceived feasibility of THPP delivery > Difficult experiences during THPP delivery | *“The introduction session was difficult…the intervention was new…meeting the family for the first time. I did not know where and how to start but the rest were easy”. (PV5)* | Difficult experiences during THPP delivery. Some difficulties were reported during delivery of the THPP sessions. Some peer volunteers initially found delivering the introductory session challenging. Over time, they gradually gained more experience, which increased their confidence: |
|  |  | *“When participant had lots of issues, it became difficult to move from one step to another. It took me some time to understand and successfully deliver it to high need participants…” (PV4)* | A few peer volunteers found challenging to deliver intervention to mothers who were experiencing complex issues and had chronic symptoms while one mother stated that the intervention was complex initially: |
|  |  | *“… the good part was we were not alone in the programme we met research and hospital staff often, they were helping us how to go about the sessions and how to deal with issues”. (PV6)* | perceived support and good collaboration with the research and hospital staff was deemed helpful in managing the challenges faced in implementation: |
|  |  | *“…We worked very well with health workers….if faced with challenges, we were asking them”. (PV2)* |  |
|  | Positive experiences during THPP delivery. | *“…it was easy to explain to women through the pictures…even those who did not go to school understood it, even the old mother in-laws were able to understand the lessons through the pictures”.* | The THPP sessions are structured and use illustrations. Most peer volunteers reported that while structure simplified the sessions’ delivery, the contextually relevant illustrations helped to explain things better and make it more relatable to the mothers: |
|  | Unexpected personal benefits | *“It was very prestigious for us to be known as counsellors in our communities. We also felt proud to see healthy babies born during the program with our counselling”* | All peer volunteers reported to have benefited from the intervention. Talking about the personal gains, some mentioned improved problem-solving skills, interpersonal relationships and social support, while others gained respect in their communities and feelings of contentment to see the impact: |
|  | Barriers to THPP delivery. | *“…Much as we were volunteers, we were supposed to be paid a certain amount of money, which would at least have contributed towards the cost of farming”.* | Some participants, peer volunteers and mothers reported HIV/AIDs stigma as a potential barrier to the THPP delivery and access. The stigma resulted from peer volunteers being perceived as Anti-Retroviral Therapy (ART) providers, who provide community care to HIV positive women. This raised concern among a few mothers. Another barrier to the THPP delivery reported by the peer volunteers was their dissatisfaction with the stipend they were receiving: |
|  |  | *“there were some partners who seemed to be very busy and never attended the sessions…mostly those in polygamous marriages…”* | lack of partners’ involvement and support were identified as barriers to accomplish between session tasks. Most mothers suggested providing material resources/handouts for the partners to improve their engagement |
| Nyatsanza et al. (2016) | Counselling as an intervention | *Sometimes it won’t be easy to go for counselling. And also for us it is a cultural thing. Most of the people they don’t value the counselling. What I notice is that people go for counselling when they have a problem. We don’t go when there is no problem… Normally there is this thing of we go when things went very, very bad, it is when you will go for help. (Community Health Worker 4)* | In addition to the above service users’ reservations, nearly half of all participants, users and providers, (12,46 %) felt that counselling might not be easily taken up. |
|  | Clinic versus home visits | *You get away from the family, which is often the problem. It is easier to open up, and you get away from the problem. (HIV Community Health worker 1)* | The study results present conflicting views on location of the proposed counselling, women in this particular community showed a preference for individual clinic based counselling sessions. This would encourage them to leave their homes which in some cases exacerbated their depressive symptoms. A number of participants, both users and providers indicated a preference for clinic based visits (9, 35 %) with reasons such as stigma of being HIV positive, issues of confidentiality and leaving the source of depression |
|  |  | *The mothers would have to wait too long at the clinic. If they get hungry or tired, they sometimes just give up and go home. So we need to be able to refer them straight to a psychologist. (HIV Community Health Worker 3)* | Although clinic visits seemed to be preferred by most, some providers (3, 21 %) raised important points to consider such as lack of space and long waiting periods at the clinics. |
| Pacichana-Quinayáz et al. (2016) | Effects of armed conflict and poverty | *these two years we were in therapy and cut energy in the city, pulling tower energy, people had to be enclosed in neighborhoods, there were threatening statements in streets [...] , although you’ll heal them they living in a city so overwhelmed by armed conflict, so if patients have an improvement we cannot know how much they will last because the exposure to conflict.* | There is a resurgence of violence in Buenaventura, and to a lesser extent in Quibdó, as manifested by respondents. These events continue to be stressful factors that cause mental health symptoms in this population, generating uncertainty about whetherintervention effects were maintained in time or if it could have a setback as a result of that. |
|  |  | *Those who suffered displacement, which lost everything and here live in houses borrowed, rented and do not have money for paying [...] say: <look, I had my farm, I had it all, and I lost everything, now I live resigned but I will never forget that, that keeps me a little heartbroken>.* | Also, due to structural poverty in these municipalities, the consequences of being a victim intensify with the emergence and / or worsening of unsatisfied basic needs, such as improper conditions for human accommodation, inadequate sanitation, and high economic dependence. |
|  |  | *I also think that people have other priorities, sometimes for example, in economic sphere, if you are hungry, mental health will be secondary, or if you get a job for your family maintenance, during a short week, then you will prioritize that.* | Respondents considered these factors affecting attendance therapies, as some users expressed lack of financial resources to obtain food, being unemployed, and living in an informal economy; these provided reasons whytheycould not refuse to work when they had the opportunity. However, the ACOPLE team tried to adapt therapy schedules when itinterferedwith a participant’s job. |
|  | Trauma Severity | *Type of trauma had a better or worse prognosis, for example, those people with situations of torture or sexual violence had a much slower recovery, [...] if causing depression or anxiety was violence sexual, was much stronger and overcome symptoms.* | Some LPCWs consider that traumas and theirseverity are different in each person, including their ways of coping which could indicate the need for differential treatment in mental health. These are exemplified as severe trauma, those related to the murder of a son, sexual violence or torture. |
|  |  | *cases of domestic abuse [...] that were happening recently, also in the environment that we live in some ways is common, then it is as easy face it instead of a person who’s been raped or tortured during displacement.* | As for domestic violence, it is regarded as a problem of low complexity, unlike the traumas associated with displacement and torture. This is because communities perceive the issue of family violence as a common problem and it is considered easier for the victim to live and deal with it. |
|  | Perceived changes with CETA intervention | *I worked with a raped victim very young, so first day she came was terrible, overflowed emotionally and cried a lot, she said: <I never talk about this with anyone, this is killing me and I know what will happen to me if I do not talk > [...] It is easier than after downloading it to carry inside for many years because their fear to talk about it, when these people dare, recovery it is easier.* | Interviewees noted early changes in users. Since the first three sessions, less anxiety, more expression and confidence, better face, feeling better about the future, a greater understanding of the therapy, and concentration as a result of thatwere all appreciated. According to testimony, users acknowledge the opportunity to talk about their traumas as being an emotional release, particularly with the accompaniment of a counselor and a psychologist. |
|  |  | *So when you started work history and did relaxation techniques, we always remember to them measure their levels of anxiety and fear at home, and always told us: <I feel good since I’m talking><This really works>.* | They also recognized in users a better ability to handle some anxiety symptoms and implement strategies learned. In the users’ words, these techniques provided them with satisfaction since they were able to anticipate symptoms or a severe anxiety response to an episode. |
|  |  | *They tell: < Scares me go where my neighbor, talk with my relatives>, then they were slowly go out their homes [...] as time passed, they were saying felt much better and could perform such activities.* | Another notable change is an improvement in interpersonal relationships. Some LPCWs mention that changes in family dynamics and generating a conciliatory atmosphere between parents and sons in some users were both possibly due to a decrease in feelings of anger and sadness. Moreover, reducing fears allowed them to go out of their homes and frequent community environments that were avoided before, allowing for the establishment of relations with neighbors and family once again. |
|  |  | *I initially thought that therapy was for crazy ones, but not anymore [...] in sessions they said: <I used to think it was for crazy ones, but really we need this service because displacement consequences>  People who really change beginning to help others, or newer, which came after therapy, they begin to teach what they learned [...] we valued when they started making support networks with its neighbors and the institutions too* | Most respondents believed that intervention reduced stigma in mental health issues;this reduced stigma was not only observed in therapy users but also in the LPCWs. Also, it was identified that users recognizedthe benefits of therapy and sometimes expressed a desire to extend this service rather thanothers, which is consistent with an open attitude toward this type of intervention, making the community more active in seeking these services. |
|  | Characteristics and LPCWs performance | *All patients are different [...] if I start from first component until the sixth, possibly in that order works for you but will not work for another one* | Both municipalities referred to the flexibility of therapy or stiffness of it as a positive andnegative factor, respectively. Some individuals admitted that the therapy was stiff but allowed for its implementation; others considered it difficult to adapt to an individual’s needs. In contrast, some felt that the therapy was flexible as they could devote more sessions to a particular component if users so desired. |
|  |  | *Most of us are also displacement victims, then it is also very easy to understand, I think I know how to create empathy with people and that gives them confidence.* | Respondents mentioned an important point within the CETA intervention: In a first contact, LPCW must make an effective “hook” which allows one to determine if participants are interested in attending therapy. Supervisors believe that LPCW should have communication skills, be resourceful, flexible and have enough creativity to conduct therapy. Many agreed that LPCW empathy was one of the main strengths, developed because of their traumatic experiences. |
|  |  | *Every day there was more motivation work because I understood how to manage that component of trauma. [...] Then, because these work, I could use knowledge daily and family life.* | According to interviewees, there are some benefits for LPCW including the generation of income and relative economic stability, wellbeing, autonomy and mental health tools that strengthen the skills of individuals to develop their own work and extend the therapy benefits into their daily lives and family life. |
|  |  | *During therapy they heard similar situations as they had to live [...] , so I think they were quite strong themes. We had a space called <Help the helper>; oxygenate is part of the team and able to talk to everyone about what it is overwhelming to me as a therapist.* | Moreover, being aware of the emotional risks involved with LPCW exposed to stories about violence, respondents recognized difficulties they might have to handle in these difficult situations within therapies. However, they emphasized the supervisor support and the spaces where the team gathered for feedback as crucial for answering questions and allowing for emotional release. |
|  | Afro-Colombian culture approach | *CETA intervention has a lack of cultural approach, lacks of getting into black community and maybe there are things we should be working [...] there are rites, customs that we could recover and that could have a very healing effect on person.* | A point emphasized by interviewees refers to a participant approach and management of CETA intervention from a cultural perspective. Participants mentioned that the protocol language was technical and the LPCW’s discursive ability to transmit a message to the participant was necessary so that they could understand each component. Other issues they mentioned that should be considered for intervention were the conservation and implementation of Afro-Colombian traditions related to artistic and cultural activities such as dancing, singing, food, fabric or any art that would allow them to take time and generate a vindication of person in order to feel useful. |
|  |  | *Therapy was confronting person with their feelings, [...] so if those situations repeated, or someone in the community suffered a similar situation now they know how to procedure, they would empower them, and then collaborated members of community because intervention ends but knowledge remains into community.* | The importance of community networks within the Afro-Colombian culture was also evident. Furthermore, how, through the intervention, the knowledge and strategies learned in therapy could provide tools for users to respond to similar situations of victimization within their community were also evident. |
|  | Strategies to promote users’ wellbeing | *There are many people who always talk about economic situation, then say: <I have these problems because I do not have job>, sometimes we do not how answer to that [...] if we can bring some information that could benefit person, then it would help a little more.* | Respondents recognized that therapy produces positive changes; however, they mentioned that these changes are adversely affected by factors such as poverty and violence in these municipalities. Therefore, it is important to consider the need for strategic alliances with institutions that can promote the generation of incomeand the recognition of each person and their right to education, health, and recreation, among others. Also, this highlights that users able to bring this kind information could generate a greater well-being. Likewise, respondents stated that monitoring cases and contact with users once the intervention finished can promote their wellbeing since Afro-Colombian communities tend to generate ties and cultural feelings during these intervention processes. |
| Paudel et al. (2014) | Management Options | *“We will provide her counseling. We will listen whatever she tells us and we will keep that information confidential within us. We will open her a new way with cooperation. Even if she has left the Bachat Gat (Self Help Group) we will re-admit her and guide her. We will take her to hospital and we will start the medication after consulting with the doctor. We will tell her about the exercise and yoga.”* | All focus groups, with active participation of members, were able to identify management options for a patient with major depression. The following management options were frequently expressed by VHWs: counseling (n = 14), social support (n = 11), financial support (n = 9), referral to hospital (n = 7), medication (n = 3), awareness campaigns in the communities (n = 3), sharing self events (n = 2) and the role of yoga (n = 2). Sometimes participants expressed many management options at a time. At other times, one VHW mentioned one or two, prompting another VHW to mention further options. Some of the responses were as following: |
|  |  | *“We would admit her in Women’s Group. We would try to open her up and mix up with other women. We will make her talk so she empties her emotions. We would make her realize that you have children and if you have any problems from your husband, please do not worry about it. If you have any financial problems we will help you. Use your money for your child’s education.”* |  |
|  |  | *“We will make people aware about mental health problems. We will educate them.”* |  |
|  | Operational Skills | *“We will seek assistance from the Farmers Club, villagers, Village Committee Members. We will also get assistance from patient’s relatives and family members.”* | As part of the concluding question for the FGD, VHWs were asked to discuss what they would need to address mental health issues in the communities if they were the chief district health officer. Most of the participants seemed eager to envision themselves in this role and actively participated in the discussion. The most commonly expressed ways to manage the needs of the communities were the following: coordination with other existing health team and community people (n = 10), financial resources (n = 6), physical resources (n = 6), community participation (n = 5), human resources (n = 4), and research (n = 4).  .Most of the groups were aware about of importance of community based and comprehensive approaches to address mental health issues such as depression in their villages. Some of them also highlighted the importance of research to find out the status of depression, and also as part of monitoring and evaluation of the project. Some of the representative quotes are: |
|  |  | *“We need vehicle, training center, resource persons, medicines, trainers and sources of information to find out the patients.”* |  |
|  |  | *“We will visit all villages of the block and organize meetings to identify mental health patients. We will also conduct a survey. We will consult with the heads of villages, and local health persons. We will frequently visit villages for the follow up to gather relevant information as not necessarily family members provide true information. First we have to seed trust in the community.”* |  |
|  | Attitude and Practice/Behavior | *“…..We will talk to her and tell her that nothing is wrong with you, don’t worry…..”* | Attitudes and behaviors of VHWs regarding the patients with depression were assessed based on the responses of participants to one question: how would you approach someone with Radha Devi’s condition? Other important responses regarding attitude and practice were gleaned while VHWs shared their experiences gained while working in the field. VHWs were very open about their own mental illnesses. Many of them associated to family problems when asked to think of examples of mental illness. Participants also had tendency to tell depression sufferers to ignore the relevant stresses or to try to distract them as a way of helping. All the groups and almost every participant expressed their supportive attitudes toward mental health patients. They were also found to be cooperative and empathetic while dealing with patients with depression in the communities as elicited in the following quotes: Though CRHP has not formally implemented mental health projects in its project villages, most of the VHWs shared their experiences helping the patients with depression and other mental health problems. All groups were competent in describing management options, which indicated that they would be able to practice their knowledge appropriately in coming days. One VHW said, “I have already dealt with a case like Radhabai (Radha Devi).” |
|  |  | *“….The other day I met him and advised him not to think about his alcoholic father…. You have good life and there are your uncles to support you so you just don’t think too much about your father.”* |  |
|  |  | *“We will take her to the hospital, check her weight, will try to read her mental state. To release burden of her husband’s illness, we have to help her.”* |  |
| Pereira et al. (2011) | Psychosocial interventions delivered by the Lay Health Counsellor | *Some (patients faced) social difficulties like financial problem which is mainly due to seasonal work, daily wages, and alcoholism. Another problem was patients not having proper documentation to apply for social schemes e.g. unregistered marriage, so a woman cannot apply for widow pension. So I found that there is no proper channel to improve their social difficulties and their problem remains same and this worsens their health condition. But I tried to give them information about various available schemes and how to follow the procedure and some even applied for it." [LHC]* | the LHCs described a number of challenges that they faced in the delivery of the psychological treatments. During the early days of the program, some LHCs reported difficulty remembering the guidelines of psychoeducation, forgetting, for example, to provide advice on some symptoms or inquire about substance abuse. Some also experienced difficulty in handling talkative patients, working with patients with extreme social difficulties, counseling people with alcohol related problems, and communicating with elderly patients who found it difficult to follow the instructions. Less common difficulties were resentment by patients when the LHCs inquired about suicidal ideas and, particularly in the GP sector, patients hesitating to open up as they were known to the doctors and the staff. Some patients requested financial assistance and some refused treatment as they did not feel it would help them |
|  |  | *"I stressed to every patient that whatever they would confide in me would remain confidential. I felt these words would make the patient feel more comfortable with me and they would ventilate their feelings more easily. Another aspect of psychoeducation which I felt has helped a lot was the mind-body link which included the patient explaining the stressors in their life which in turn would affect their health." [LHC]* | the LHCs adopted a flexible approach of delivering psycho-education, improving their skills in asking about suicidal ideas, and providing concrete information on welfare schemes and referral agencies to address social difficulties and reviewing progress on these issues in follow up appointments. The LHCs observed that providing explanations about the importance of treatment and explaining the mind-body link also helped engage patients who were skeptical about the program's effectiveness. LHCs also learnt to emphasize confidentiality which helped patients become more comfortable in talking to them. |
|  |  | *"Patients told that they won't be able to come for so many sessions. Some patients postponed the appointments and finally they dropped out. Some refused to continue saying they are better and don't need to continue. I felt that IPT is a good therapy but the response in my PHC was poor. Whenever I introduced IPT to patients most of them said that IPT will not help them to deal with their problems; at least with medicines they will feel better." [LHC]* | LHCs delivered Interpersonal Therapy (IPT) face to face in the primary care practice. However LHCs faced great difficulties in delivering Interpersonal Therapy (IPT) for two major reasons: first, because patients were unable to attend the required minimum of 6 sessions, and secondly because some patients were skeptical about its potential benefits, preferring medication. |
|  |  | *"Doctor was convincing patients to meet HC and take benefits of the counseling and to regularly follow up. He was also discussing with me about the treatment of the patients." [LHC]* | Many LHCs mentioned how the conviction of the primary care staff about the benefits of the program was a critical element in enhancing patient engagement. They reported some challenges with engaging the primary care doctor, especially in the early days when some doctors were not referring patients for counseling or playing an active role in encouraging patients to accept the new treatments. However, the involvement of the doctors increased as they became aware of the positive feedback about the treatment from patients. In addition, some patients who had felt their health improve following counseling also encouraged others to participate and even created awareness in the community about the program. |
|  | Patient adherence | *"I used to emphasize taking medicines (antidepressants) for at least six months and gave flexible follow-up appointment according to patient's convenience. I am always in my cabin during the appointment timings as some patients were on time ...I remind patients who miss their appointments with phone calls, letters or contacting through PHC field health workers. It is natural that patients can forget their appointment when they are stressed or depressed." [LHC]* | MANAS faced a number of challenges in achieving patients adherence to the program for a variety of reasons; the team generated a range of strategies to overcome these challenges. These included a variety of strategies used by LHCs, sometimes with mixed responses. |
|  |  | *"Many times I am faced with the problem where patients do not like us to send letter to remind them of appointments or phone them. In one instance one patient came and told me "you stop sending letters to me, people are suspicious why letters are coming to me from the clinic. I will continue my medicines". So I stopped but that patient was regular in taking medicine. But there was another patient just opposite; she would come to the clinic only when she receives the reminder letter." [LHC]* |  |
| Rodríguez-Cuevas et al. (2021) | Impact in CMHWs and Community Care | *I live with my mom, and I used to fear making my own decisions before (becoming a CMHW). I would expect others to decide on my behalf, but not anymore. Now I make my own decisions and my mother and family respect them… I only studied until middle school, but now I am determined to major in psychology. I know this (job) made me fall for psychology, and I would not like to stop doing my work. You can help people not only by being a doctor or a nurse, but by being like me, some sort of a psychologist.* | CMHWS value the empowerment and noticeable changes in self-confidence that came with the process of becoming a CMHW, and by learning a strategy that could help people living with mental health conditions: |
|  |  | *People bear their mental health complaints, because they go to doctors that make them spend a fair amount of money without them giving a real solution or a diagnosis, in plain 2020! But here, within the community, a lot of people have benefited from the clinic and are content because they are able to move forward, in comparison to before, when they did not know where they could go to, and would just endure, because that is how it was supposed to be. Husbands used to mistreat women instead of supporting them: they would say they were lazy, that they just wanted to sleep, but now we know it’s due to a health condition.* | Moreover, having a psychosocial treatment option to deliver within the community has meant an epistemological rupture in mental health care in the communities where CMHWs live and serve, as expressed by a fellow CMHW: |
|  | Case Presentations 1: When adaptations are made to fit the context needs | *I had the case of a 64-year-old woman that attended the clinic with depressive symptoms. The physician referred her to me, so I went to her home to introduce her to the PM+ programme. She had an overly complicated case; she was a lonely woman, almost socially abandoned. She was living with diabetes and hypertension, both treated at her local clinic, and had lost most of her visual capacity. Hence, I had to adapt some of PM+ activities, such as the calendar for (the behavioural activation component) Get Going, Keep Doing, which I had to draw on a bigger scale in a flip chart sheet. She would place it on her walls and remember what we had agreed on doing. She also had some anxiety symptoms, such as fatigue and worrying too much. It was hard to see that she had several incurable diseases; however, depression had a cure. Over time, she started getting out of her house, she got closer to her family, with whom I also had the chance to speak to (to explain to them what depression and anxiety were and how they could help). Once, we went for a walk together and there was another time when we planted flowers together. We managed to get her a radio where she could listen to music. She was very content when we finished PM+ sessions. Afterwards, I would continue visiting her every once in a while (as part of the psychosocial accompaniment visits), because we are also part of the community and our patients will always be regarded as our patients, so we will never abandon them. Whenever I found her listening to the radio, I would feel very joyful.* |  |
|  | Case 2: When the implementation process differs from the book: | *I had the case of a 50-year-old woman whom the physician diagnosed with depression, so he referred her to me. During the first visit I made her, she shared that she had had suicidal ideation accompanied by low energy and negative thoughts. She told me that she missed her dead father, as well as her sons when they were away; she also had a difficult relationship with her husband, who had a heavy alcohol use. The patient kept talking and seemed to want to spill everything she had had to endure. I remember that during our training, we learnt how to obtain information from patients through the forms and get the clinical scores (PHQ-9 and GAD-7), but I couldn’t do it during the first session, as I didn’t want to interrupt her story, so I just let her talk. The same happened during the following sessions, in which I couldn’t do the questionnaires, but showed her the relaxation techniques and activities to get distracted from “thinking too much”. A safety plan was done too. I commented this case with my case supervisor and she told me that catharsis was important for this patient even if that meant not fully adhering to PM+ sessions, and that I could continue visiting her, because it helped her. Had I followed PM+ sessions’ structure by the book, I would have lost this patient, since she would have felt unheard and pressured by the questionnaires. Having patience with her helped her to feel free to express everything she wanted whenever she wanted to. She finished her PM+ sessions and now she enjoys visiting the village and talking with friends as part of her support network.* |  |
| Saleem et al. (2021) | Self-regulation | *“Some people said that they would not even leave their house. From this (the meetings) their social skills improved, they said they gained confidence to talk. Females would say that they were very fearful in talking, through this platform they would talk openly” [C, female, 27 y/o].* | Participants felt that the experience had had an impact on their self-regulation of emotions and behaviours. These changes were also noted by caregivers and others around them. |
|  | Sense of belonging | *“I felt a connection, if there was a problem then it was everyone’s problem.” [C, female, 27 y/o].* | Participants described their experience of sharing with others as a source of strength and hope which allowed them to unburden and look forward. |
| Selohilwe et al. (2023) | Facilitators (Counsellor organizationally integrated into facility, Supervision and  support for  project-employed counsellors) | *I was happy because I felt that I was taking the patient’s problem as my own, so he (the supervisor) helped me not do that…. (Counsellor2)* |  |
|  |  | *I was part of the team and I felt that the job I do is important to the team. (Counsellor2* |  |
|  |  | *I usually attended staff meetings and sometimes they would want to know about my work and how I am coping. It was then where I would communicate about patients and work. [This was helpful] because they would also motivate each other to start referring. (Counsellor5)* |  |
|  |  | *[Supervision could be improved] by making it a regular thing, because sometimes they would come and then take a while to come back again, and the supervision onsite is better because the focus is solely on you, unlike the [group] supervision. (Counsellor5)* |  |
|  | Barriers (Counsellor and  counselling activities not  integrated into the facility) | *[…] I didn’t have a good relationship with the facility manager […] she was not the kind of person who would want to know how is it going with my work, if I had space to work in and if I was fine. I had to stand up for myself to secure space to work in (Counsellor4)* | Half (n=4) the project- employed counsellors reported not feeling integrated into the facility and facility activities. This was reflected in not being given adequate space to work, not being included in staff meetings and generally feeling marginalized, with the counselling service not recognised as important. This had a negative impact on counsellors’ confidence and the programme generally |
|  |  | *[…] at first, they did not refer and I had to prove myself in order to have a working relationship with the sister. They didn’t see me as part of the facility- “no you are for depression and you are for PRIME”. It was difficult but I managed to pull through (Counsellor8)* |  |
|  | Barriers (Space) | *I have been working outside without a counselling room for a long time. I think that is one of the reasons patients don’t come back especially in winter because we have our sessions outside due to lack of space. (Counsellor2)* | Lack of dedicated counselling space was discussed as one of the main barriers in the inner setting by all of the eight lay-counsellors interviewed. The counsellors below discuss how the space challenge frustrated them and compromised patient privacy. |
|  |  | *[lack of space] affected my work a lot, because I realised the patients were losing their trust in me as they observed I don’t have a dedicated space to work in (Counsellor4)* |  |
|  |  | *[..] You struggle to get a place to work in, and that is frustrating even for patients because they never know where to find you (Counsellor5)* |  |
|  | Siloed approach to  HIV counselling and  testing | *We should know more [about mental health disorders] because some patients on ART have psych [disorders]. (HIV counsellor focus group3: counsellor)* | HIV counsellors interviewed indicated a willingness to be trained to deliver the counselling intervention. Over half the participants interviewed (57.9%) reported that the situations they encountered in their line of work often exceeded the counselling capacity and capabilities acquired during their training for HCT counselling. Participants in focus group 3 discussed mental health problems as part of HIV/AIDS counselling and an ensuing need to integrate psychosocial counselling as part of the functions of HIV/AIDS counselling. District manager 10 acknowledged that lay health workers can be trained to provide counselling for positive patient health outcomes |
|  |  | *I just wanted to know about people that have depression, they do come sometimes and [...] usually we find people that have depression also have hypertension and especially diabetes. (HIV counsellor focus group3: counsellor)* |  |
|  |  | *After post-counselling, it’s not every time that the person accepts [their status] you will see on their second visit that they have not yet accepted [their status] and that’s when mental [illness] appears. […] You often hear that mental health problems are caused by [people] receiving their [HIV] status and they start changing, start thinking about a lot of things and depression kicks in. [...] I really think we need to know more so we can identify the signs and symptoms when they come for their follow up sessions (HIV counsellor focus group3: counsellor)* |  |
| Singla et al.(2020) | Barriers | *“Mini Meetings [supervision without experts] are time consuming and also travel allowances aren't paid to us and neither are expenses … We visit once in a month and plus we conduct mini supervision ourselves so this is not productive for us.” (Sakhi_23, Round 1)* | Initially, the majority of both Sakhis and supervisors reported difficulties with travel due to a lack of compensation and a lack of time to attend meetings due to other commitments, especially in supervision where experts where not present. These barriers remained the most endorsed across the duration of the trial. |
|  |  | *“Sakhis say that they don't have time—that is why they don't have [mini-meetings]. But we encourage them to have these [mini-meetings] as they will benefit from it.” (Supervisor_03, Round 1)* |  |
|  |  | *“In absence of a supervisor, there is no control in our discussion also and we don't rate the session. So I don't feel it benefits us but consumes time.” (Sakhi_20, Round 1)* | Both experts and Sakhis reported that supervision without experts often lacked relevant feedback, and that there was overall low motivation to attend these sessions. This remained unchanged throughout the trial despite attempting to address challenges such as travel allowances. |
|  |  | *“When there are difficulties that have been brought up again there was a chance that the peer would give wrong feedback. Because there would be a wrong answer which couldn't be corrected because we [Supervisors] weren't there.” (Supervisor_02, Round 2)* |  |
|  |  | *“At first, Sakhis didn't understand the rating scale. But then, we told them to read each item and then we would discuss. […] After reading, they understand the meaning. But at first, they would fill in [the rating scale] because they have to do it without understanding.” (Supervisor_01, Round 1)* | Other key barriers reported by both stakeholder groups reflected peers' initial difficulties in rating the TQS and leading supervision as well as being score a ‘0’ to score individual items on the TQS. Initially, Sakhis reported feeling demotivated when rated ‘0’ on an item. By endline, a number of barriers such as initial difficulties with TQS items, a lack of financial incentive, competing demands and a low quality of feedback were less frequently mentioned |
|  |  | *“[…] After listening [to my recording] if all say that it was good then I feel very nice. If they rate me 0, then I feel bad thinking that I worked hard and still I got rated 0.” (Sakhi_17, Round 1)* |  |
|  | Facilitators | *“[Structured supervision] has helped a lot. We understand and learn from our mistakes. We get to know how we can deal with the problems … so there is improvement in our sessions.” (Sakhi_20, Round 2)* | The most commonly reported facilitators by Sakhis were the benefit of using a structured scale to rate therapy quality that in turn facilitated consistent and relevant feedback and fostered a greater understanding of Sakhis’ challenges and skills. This was also endorsed by all supervisors and across the duration of the trial. |
|  |  | *“When we fill in the TQS, we come to know where Sakhis are facing challenges and where they have done a good job. Suppose they have difficulties, we come to know and where else they need more training. That is why I feel it is very important.” (Supervisor_03, Round 1)* |  |
|  |  | *“When all the Sakhis would meet [for group supervision] there used to be good discussions on how to handle the difficult cases or any case. We would get an idea about it.” (Sakhi_11, Round 2)* | A major facilitator reported by both Sakhis and experts in supervision with experts was also the positive group processes, where both expressed that this was a chance to meet and solve common problems before bringing it up with the supervisor. |
|  |  | *“Group supervision is better since there are just 2–4 in a group. It is better because we can ask them what difficulties they face. […] And if they cannot do something, then they ask us. Since they are few in numbers, they ask us in what better way they can do it [the session] well.” (Supervisor_02, Round 1)* |  |
|  |  | *“When they [Sakhis] live close by, then mini-meetings happen very nicely. They make time and sit together or those who are at home only don't go for work. Their mini-meetings take place because the Sakhis are at home and don't go for work.” (Supervisor_04, Round 1)* | In general, both experts and Sakhis preferred the expert to be present to ensure that Sakhis attend supervision, that the information being shared was accurate, and to help with difficult cases. Similarly, both experts and Sakhis noted that living close to one another was a primary facilitator for peer supervision without an expert present. |
|  |  | *“… currently our group is really nice because we live close to each other so it is convenient for meetings, so we do mini meeting at my home or theirs.” (Sakhi_22, Round 2)* |  |
| Szajna et al. (2024) |  | *ASHA #3: Initially, we were concerned about administration of the EPDS. This was resolved during training by performing role play based on various scenarios from the training officers and later [by working with] the participants. ASHA #5: After training, we were much more confident in screening through the EPDS and [doing] the intervention through role play. ASHA #8: The tool [EDPS] was in the local language which made it easy to screen the mothers. ASHA #2: All ASHA workers need to be trained. These Activities were non-pharmacological and don’t involve any risk. ASHA #10: If more ASHAs were trained … it is helpful in preventing PPD by regular BA activities.* | All 24 participants completed the activities on their weekly Behavioral Activity Log, also suggesting high acceptability. Qualitative results from ASHAs and participants suggested enthusiasm for the training and intervention components. |
| Wood et al. (2021) | LHW Personal Characteristics | *I would like more of pointing out mistakes. I would even ask everyone in the group: tell me where I went wrong today…Then next time I would take care as much as possible not to make the mistake. (LHW, Female, 46 years)* | LHWs and stakeholders saw LHWs’ ability to give and receive feedback as a helpful personal trait. This was closely related to another important trait, i.e. a desire to improve and challenge oneself. A few LHWs described specifically asking for negative feedback in order to improve: |
|  |  | *I do not carry the workload at home because we were taught to always keep a boundary otherwise there will be so much pressure that it will hamper your personal life…That is why, when I leave home and my personal life, then I leave all my worries back there and when I go home from here, I leave it all here. (LHW, Female, 38 years)* |  |
|  | Training | *Earlier when I saw a mentally unstable person I would be scared. ‘Oh God! Will he hit [me] or what? Will he do something to me? It is better to not go in front of him.’ But now that fear is not there in our minds. He is not well…Those people do not trouble us on their own. If we trouble them, they trouble us. These are some small points about which we got information because of the training. (LHW, Female, 36 years)* | LHWs and stakeholders sometimes mentioned that the content of the training was challenging, but LHWs felt they were able to grasp the material by the end of the initial training, as well as through refresher or new trainings over the course of their job. A few LHWs said that they previously believed in certain myths about mental illness, but training changed their attitudes. Stakeholders expressed this idea strongly as well. |
|  | Supervision | *At no time was it dominating or getting angry with us for making a mistake. That never happened. So this was very helpful for us in this job. It’s never like this at other jobs; if we make a mistake we get fired. But it wasn’t like this over here. They would explain things well to us if they needed to tell us something and everything was communicated in a positive manner, we were encouraged, motivated and this helped us a lot. (LHW, Female, 25 years)* | LHWs valued the role of supervision and expressed that it had to be timely, frequent, and impartial in order to be effective. The quality of relationships between LHWs and their supervisors were a key factor in LHWs’ success. Most often, relationships were very positive. LHWs who had a good rapport and an egalitarian relationship with their supervisor often spoke more positively about the supervision they received. When LHWs did not have a good relationship with their supervisor or felt impacted by status differences, it affected their work negatively. |
|  |  | *There are [staff] in the clinics who are more educated than us. It’s difficult for them to accept us and what we do. It’s difficult for them to accept the change that we bring and it’s hard for them to blend in with us. They will not coordinate with us and they speak offensively about us. It’s also hard for other people to accept us since we are not highly educated and non-professionals. And they prefer doctors and professionals. (LHW, Female, 30 years)* |  |
|  | Working with patients | *“the patient is not available sometimes or even if the patient is at home they say that they don’t have time.” (LHW, Female, 25 years)* | One of the most commonly described challenges in LHWs’ daily work was contacting their patients or reaching them in order to conduct a counseling session. Often, a session could not happen because, for example |
|  |  | *[They would act like] how we behave with the salesman when they come door to door; “No, no, no we don’t want, don’t want,” that way they would [say]. So a little… I would feel bad, are we going to their doors to beg I would feel. (LHW, Female, 37 years)* | Patients and community members were sometimes uninterested or unwilling to engage |
|  |  | *They call us Didi [sister]. If anything happens in their own house or in the neighboring houses then they call us. And they trust us so much that if they have any family matter or if their relative has some problem, then they call us. (LHW, Female, 32 years)* | However, once LHWs succeeded in reaching patients, their relationships were generally positive. They built trust and developed friendly rapport with their patients: |
|  | Travel | *Bringing patients from there [their village] to here [the clinic] for treatment is very difficult. From there, we have to take two buses and one rickshaw. Now if it is us, seven kilometers, we will walk. If there is a two-wheeler we will get a ride, if not we will keep walking, then if there is someone with a rickshaw, someone known then… they will stop and take us. With so much difficulty we have to come here. (LHW, Female, 52 years)* | Most LHWs traveled to reach patients or get to a facility, using a variety of modes including walking, motorbike, rickshaw (three-wheeled multi-passenger vehicle), bus, or sometimes the office vehicle. Travel was sometimes timeconsuming and tiring for them |
|  | Work Hours | *None* | LHWs were sometimes asked to work long hours, on weekends, or during the evening. Traveling after dark sometimes made them feel unsafe, and LHWs also mentioned missing spending time with their family. They disliked when work times were not flexible or when they were expected to work a prescribed number of hours or at specific times, even if they had already completed all their tasks. |
|  | Environment | *I have an area in which there are many [drug] addicts, addicted a lot, meaning even during the daytime they are ‘into the addiction’. That is why, initially, I did not feel like going to that area. Even the people from the other area would tell me; madam you don’t go there. If you have to go there you don’t wear any gold because there are many robberies that take place there. (LHW, Female, 36 years)* | The physical environment that LHWs worked in was also challenging, either because of weather (extreme heat or rain) or  because of the safety or suitability of areas to conduct sessions |
|  | Workload | *Because [during counseling] you are thinking as they are talking. And travelling is so much. After doing all this you come to [the facility], you sit and some other work of typing…So it is too much, very horrible. This I did not like…Yes, it is too much, yes. Work is often emotionally taxing. (LHW, Female, 37 years)* | Many LHWs spoke of feeling burdened by their workload, while a few said that they felt motivated despite having lots of work. |
|  |  | *The reason we don’t feel the workload… when we look into cases, the patients can get back their life. The people who have been cooped up inside the house for 10-20 years, those who have lost a grip on life… when we interact with these groups, when they start brushing their teeth, when they start going out or talking to people or working, this recognition or good times of life make us happy. (LHW, Male, 41 years)* |  |
|  | LHW Role | *One of the challenges was that, sometimes a [patient] could not understand why we are coming there if we are not fulfilling their need. [For] example, if we are not giving medicine. We are just counseling and we are talking. But they feel that ok, if they are not getting any benefit, financial help or medicine help, then why should I give my time to them. (LHW, Male, 38 years)* | Several LHWs spoke of how their role could feel limiting. Sometimes, patients’ expectations did not align with what the LHWs were there to do. In addition, LHWs sometimes felt that there were additional tasks they could do or skills they wished to learn but that were outside their job description. |
|  |  | *Many times I felt that I should help them [with] this and that and then it was out of our protocols, we could not go, we could not do that. And I felt that the[patient] really needed it. (LHW, Male, 42 years)* |  |
|  | Job Security | *In any organization, [it should be discussed], ‘okay this project is getting over’, we should be taken in other project or new project so the security is there and we are stable…Otherwise we work for some time and then there is no work for us. We are left alone…So that is very hurtful. We love to work for [NGO]…but when the project ends it is very hurtful. (LHW, Male, 42 years)* | Some LHWs across programs and organizations described stress about job security as a challenge in their work. This was most prevalent in organizations funded by time-limited grants or that lacked certainty about funding |
|  |  | *There is always this thing in mind that, ‘What if this work stops? …What will we do if it stops or [the NGO] leaves…What if the NGO shuts…So this fear is always there. ‘What will happen?’ If this matter could somehow be fixed permanently, and we could be relieved about it, then, it would be really helpful. (LHW, Female, 28 years)* | To ease this, some LHWs suggested that NGOs could provide a certification of skills gained in the task-sharing intervention in order to enable them to seek similar jobs elsewhere. |
|  |  | *I know definitely that in research [projects] there are certain specific time limits… But if these things are benefitting someone, and someone needs them in the community, then we don’t feel too good about telling them that this is time-bound. Because suppose the project has ended today and you arrive tomorrow then our services are not available to you tomorrow. (LHW, Male, 32 years)* | In addition to the financial stress, some LHWs described the emotional toll of having projects end when the community was still in need of services: |
|  | Recruiting LHWs | *When we describe the work, it is very difficult for us to get a local volunteer or a [health] worker. Because [they say] “do we have to go and see them at home? Do we have to walk? Do we have to roam in the sun? Do we have to go so far? The salary is too low. You are asking us to walk so far for this. If I go to a shop, sit and do business, I will get so much. If I go to a company, whether there is work or not, I will get a salary for that month. But you are saying I have to see so many people at home, have to meet their needs, have to talk to them, that too you will have to talk a lot with them, things like these will be very challenging.” (LHW, Male, 34 years)* | Although some LHWs and stakeholders reported that their organizations had plenty of applicants for LHW positions, others struggled to recruit and retain LHWs. In general, NGOs that paid competitive salaries to LHWs had an easier time with recruitment. Challenges in recruiting LHWs were sometimes related to the difficult nature of the work: |
|  | Inefficient Systems | *Whatever we ask, [the government] won’t give it immediately. Whatever we say, they won’t do. They will say ‘It is for [the patient], why are you coming? Have you gotten money?’ Now, if an illiterate lady hasn’t received money, her son hasn’t received money, when we go and talk on their behalf, they will say ‘Who is this? Why are they coming? Why are you coming? Ask them to come.’ They will make them walk several times. (LHW, Male, 34 years)* | LHWs and stakeholders described slow, insufficient, or corrupt social service and health systems as a challenge in task-sharing work. Many LHWs reported that these shortcomings made it difficult to help their patients access services. |
|  |  | *So all the local governing bodies, clubs, municipality, they were under [name of political party] rule, and they went from this, there was this transformation to the [name of other political party] rule. So whatever allies we had, whatever contacts we had with the political leaders, we lost it all. So [LHWs’] salaries were stopped midway…they didn’t receive their salaries for 2 years. (Stakeholder, Female, 26 years)* | The task-sharing programs had widely varying relationships with local politics and politicians: some were directly funded by political parties, while others were independent but sought assistance from local political leaders, and others intentionally avoided any association with local political issues. Direct association with a political party was almost always a challenge in LHWs’ work because it created job instability and demands on their time. For instance, one program lost its funding when there was a change in political leadership: |
|  | Social context (Gender) | *When I discussed about working for this [program], at that time, my mother-in-law was alive. She stopped me. [She said,] “In our family, our daughters-in-law do not work.” (LHW, Female, 45 years)* | Social and cultural norms negatively impacted LHWs’ work in a variety of domains, most prominently gender, social class, religion, and stigma about mental illness. These issues were often compounded on top of other challenges discussed in previous sections, making them even more difficult to overcome. However, not every LHW experienced each of these challenges; for instance, a few female LHWs said gender was not a barrier in their work, but for the majority it was. Despite some LHWs’ efforts to see past them, these issues were a major factor in LHWs’ experiences. Both LHWs and stakeholders noted that gender norms—specifically discrimination against women—affected LHWs’ ability to do their work and community members’ ability to access services. For female LHWs, family expectations or fears about personal safety sometimes limited their ability to carry out their work, especially when it involved home visits or late hours. |
|  |  | *For me, the person I’m seeing is unwell, I don’t [care] whether it is men or ladies. But, things like that, he [the patient] pinched my lip and kissed. Those kinds of problems are there. (LHW, Female, 27 years)* | Female LHWs spoke about gender-based harassment and feeling unsafe or uncomfortable when performing their work, especially when travelling to remote areas or conducting home visits with male patients. |
|  |  | *Then I understood that this training was completely about me. That I have to know my mind. That I should give myself importance in life and that I have freedom and I also can do something. My body, my choice, my life, we did not know that we can avail of these things. We are girls, we are not supposed to do this, not do that. You have a life where you are supposed to take care of your husband only. [Now] I could leave that place and come out. (LHW, Female, 38 years)* | For some LHWs, the sense of empowerment they gained from their work was strongly related to embodying alternatives to traditional gender norms: |
|  |  | *In that locality [where a particular religious group lives], girls are not allowed to step out of their home. Which is why they were unable to take this service. (LHW, Female, 45 years)* | In other cases, cultural norms prevented female patients in the community from accessing services in the first place, largely because of barriers to leaving their homes alone (for services that were not home-based) or fears about the consequences of seeking help. |
|  |  | *The ladies are very afraid of society. If I take an action [in the case of domestic violence] then my husband will leave me. Or the name of my parents will be spoiled. (LHW, Female, 37 years)* |  |
|  | Social context (Class and Caste) | *There were one or two [potential patients] who were this, you know, this high class. And they felt that just talking is not solving their problem…they are saying this is for uneducated people. (LHW, Female, 57 years)* | Social class and the caste system impacted LHWs’ work in three ways. First, some upper-class community members were skeptical of the interventions. |
|  |  | *As soon as I went there and came back, my mother-in-law told me to bathe and then enter the house. When it was like that, they say ‘Because you come in, gods don’t come into the house…’ They have shouted at me saying things like this. (LHW, Female, 52 years)* | On the other hand, some LHWs faced stigma for working with lower-class or -caste patients. One LHW described working with people of a stigmatized caste. |
|  |  | *Now, say we are building awareness [in the community]. Now, SC [scheduled caste] community will be separate, MBC [most backward community, considered a slightly higher caste] community is there, they won’t sit together as a group. They will sit separately. It will be difficult for us. (LHW, Female, 38 years)* | Finally, the caste system hindered LHWs’ ability to deliver interventions because it caused division among their patients. |
|  | Social context (Religion) | *When we visit [religious group] houses, they have temples and all and the patient and others are not ready to accept us and they said we don’t want your treatment and avoided us and they said our gods don’t like you coming here. But we convinced them and made a good relation with them and I even prayed at their home. (LHW, Female, 30 years)* | Religious norms and beliefs also impacted LHWs’ work. Religious differences between the LHWs and patients were sometimes barriers to establishing trust. Religious and spiritual beliefs also created myths and stigma related to mental illness, which the stakeholders and LHWs had to overcome in order to deliver the interventions: |
|  | Stigma and Mental Illness | *[Family members] started asking questions like ‘Why do you people come here?’ They said, ‘After looking at your bags, other stuff and your vehicle, the neighbors started asking questions about us, so we don’t want your treatment…’ They don’t want to make public or exhibit that their [family member] is having a problem. (LHW, Female, 37 years)* | Stigma about mental illness was a major issue that created barriers to the task-sharing interventions. First, it deterred potential patients from seeking care or receiving proper care. In addition, patients’ families were sometimes afraid of facing discrimination in response to their family members’ mental illness and stigma sometimes led them to impede the delivery of care. They would deny that their family member had a mental illness at all, or would not seek or agree to treatment. |
|  |  | *[My] family may [think] “by mixing with mad people, she has become mad too. Her mind is gone too.” So when things like that come up, from family or from neighbors, at that time I have to make myself understand that if someone is calling me mad, am I mad? Or maybe I have to ignore them, thinking, how does it matter to me what they say. (LHW, Female, 28 years)* | Finally, LHWs described a common myth that by working with people with mental illness, they could “catch” mental illness too: |
| Yator et al. (2022) | Capability | *At the beginning of the sessions, there were challenges because all the people were still new, so it is later that people came to know each other and developed trust in the group and everyone could say all her issues (SK, age 50, CHV, Kariobangi).* | The CHWs found the intervention useful in terms of how the intervention built their knowledge and skills and successfully delivered IPT-G. The CHWs were very satisfied with their achievements and some shared their experiences on the milestones towards IPT-G delivery |
|  |  | *… it has changed us, as much as we were the teachers, but we feel that it helped us. So, I just pray that more of that kind comes and the way I said that when you just change one person, he/she will change five others and those five others will change others, and at the end of the day you may find that you have changed the whole country and even the world, so thank you (JA, age 24, CHV, Kangemi).* | One of the CHWs underscored the potential impact IPT-G in the community as indicated in her explanation: |
|  |  | *Not a burden because it was usually once a week, and if you are meeting those clients later, it is the one in which you can negotiate the time that you will meet, so I don’t think, maybe if others think otherwise (LN, age 34, CHA, Kangemi).* | One of the CHAs affirmed that the time for IPT-G sessions could easily embedded into their routine activities since it consumed only 90 min once a week. |
|  |  | *I can remember there is one who said after the sessions, ‘nowadays I can get out and talk with women, I can go out of the gate because when I was alone, I felt that I am not okay because I have HIV and I have given birth at a younger age. But now I have gotten that courage after these sessions to go outside I can talk, I have that courage,’ so that made her feel that they are many and she is not alone” (LN, age 34, CHA, Kangemi).* | After our study, most health care providers at the two health centres appreciated the impact of IPT-G on adolescents attending the PMTCT clinic, citing improved social functioning, better communication and appealing personal hygiene/grooming by the adolescent mothers. As an illustration to share this point further, one of our participants was grateful for participating in the sessions and shared her positivity: |
|  |  | *“It was just like Kangemi; at first, for the people with whom you are not familiar enough, they could not open up; you ask her a question, and she feels like where do you want to lock and take her? But now the second time, they will be free, and the third time she will feel like ‘I can remember what happened to me and how it is on somebody else,’ because we had supervision like from [the] CHA and MK and could correct us when [we were] wrong and it becomes normal” (SN, age 65, CHV, Kariobangi).* |  |
|  |  | *“I think even these girls have become role models in the community … we told them ‘if you find somebody who is in a situation maybe you can assist and feel that it [is] closer to yours or it is similar to yours, you are now empowered, and you can help this person at your level and if you feel that it is difficult is when you can refer.’ Still, now they are doing on their own, which is good (EO, age 24, CHV, Kariobangi).* |  |
|  | Motivation | *Even the sessions were very good; because some used to come and tell you, I have this and that problem and I passed through this, but I went and did what you told me, and I have succeeded, you get it? One member in our group, I recall, and even at the moment, she normally rings me and tells me, ‘that thing helped me, and I am able to help others’ (NG, age 43, CHV, Kangemi).* | The intervention empowered some of the adolescent mothers to help others, which was one of our intended purposes towards disseminating benefits to the community level: |
|  |  | *So, it was a very big problem at the beginning, but I am glad that as the sessions were proceeding, we became good friends; actually, we developed a rapport, and even until now, some still call ‘when are we meeting again?’ [Laughter]… (EO, age 24, CHV, Kariobangi).* | The IPT-G sessions formed a part of the entry point to enlarging the scope of their social support and even after the intervention they continued to share their issues: |
|  |  | *What I can say; when I joined—let me talk about group two, we came well but after attending like three sessions is when you could see them now pouring out [Laughter] … they tell you all their troubles in life… (FC, age 30, CHV, Kangemi).* | Others were of the view that follow-up is important because most of the participants open up to their issues in the middle of the sessions: |
|  |  | *So, we are also still doing follow-ups. Some even call by themselves, sometimes I call them, some even when they come for clinics, they just come looking for us which is good so, so they trust us. So, there is that trust, there is that friendship, there are so many benefits that came out of this, so I was just gladly sharing (EO, age 24, CHV, Kariobangi).* | The CHWs were now able to connect easily with the adolescent mothers after bonding with them during the sessions: |
|  |  | *I think for us [in] Kariobangi, we had a big challenge when it came to the venue of the meeting because sometimes you find we are here sometimes we are displaced at the tent, maybe the other tent is very dirty, sometimes we are in this other tent we come here displaced, so it was a very big challenge [cross talk] …. (EO, age 24, CHV, Kariobangi).* | Nevertheless, several CHWs acknowledged the lack of a designated space for mental health services as one of the limitations towards adopting IPT-G delivery to routine PMTCT services: |
|  |  | *We were okay; we had been given the maternity, a place somewhere…. You see, if we close the middle of the room too and pull the curtains, it was so good; it didn’t have an issue (LN, age 34, CHA, Kangemi).* | The space to administer mental health services was missing within the health centres as shown by how we had to improvise a room for the sessions: |
|  |  | *But let’s just say it was okay; even if it is bad, it is our place, so we cannot say that it is bad [laughter], but it was fine (JA, age 24, CHV, Kangemi).* | Another CHW seemed helpless because of the situation and was contented to the state of the available space despite all the challenges associated with it: |
|  |  | *The space was okay; it was somewhere where we don’t have issues with noise, but we don’t know next (SN, age 65, CHV, Kariobangi).* | The lack of a specified venue for mental health services caused uncertainty for effective service delivery for the CHWs: |
|  | Opportunity | *In fact we always talk about that group (IPT participants) wherever I go and maybe I meet a partner. I am still looking for a partner to support them. I always talk about it because we felt that we changed them and we wouldn't like them to get lost on the way, we would love them to come back and help the others (LN,age 34,CHA,Kangemi).* | The CHWs felt more aware of the needs of the PPAs after the group sessions |
|  | Building capacity and acknowledging the team | *“Follow-up of that client, you have done IPT for this client and you have done termination session; you have referred that client from facility to the community, who will do that follow-up? The community health workers. They are the linkage between the facility and the community and community to the facility.” (LN,age 34,CHA,Kangemi)* | it was very encouraging to hear from the CHWs that our participants after intervention became role models in the community by imparting skills in management of their day-to-day issues of life |
|  | Third IPT session is when disclosure happens | *“- -yes, they exist; when we started, we had a challenge because one we had not been trained, we had a challenge but we reached where we reached. I can say that even those girls when we start with them there are usually problems but the good thing is that mid-way they pick and can listen to you and sessions go well.” (JA,age 24,CHV,Kangemi)* |  |
|  | Group strengthens cohesion and empowers | *She (PPA) realizes that she is not the only one and that they are several, don't you even see that the first thing they were accepting so much whenever she hears that this one is also like me, even this one and the other one so she feels that 'we are many.” (NG,age,CHV.43,Kangemi)* |  |
|  |  | *“It used to help them to unwind because maybe somebody has an issue and feels like 'maybe it is only me who has this issue' so when she finds somebody in the group who says almost like similar to hers then she also opens her heart and speaks.” (EO, age 24,CHV. Kariobangi)* |  |
