# Supplementary material for: Delivering low-intensity psychological interventions in low- and middle-income countries: A systematic review and meta-ethnographic synthesis of lay health workers’ experiences
Source: Glob Ment Health (Camb). 2026 Jul 7;13:e157. doi: 10.1017/gmh.2026.10271 (PMC13433339; doi:10.1017/gmh.2026.10271)
Supplement: Supplementary file 1 [file S2054425126102714sup001.zip › Supplementary material 4 - CASP _Quality Appraisal.docx]

**Supplementary material 4**

**CASP appraisal of methodological strengths and limitations**

| **Author** | **Q1_**  **aims** | **Q2_**  **Methodology** | **Q3_**  **Design** | **Q4_Theoretical**  **Underpinnings** | **Q5_**  **Recruitment** | **Q6_Data**  **Collection** | **Q7_Researcher**  **Participant Relation** | **Q8_**  **Ethics** | **Q9_**  **Analysis** | **Q10_**  **Findings** | **Q11_**  **Value** | **Summary of methodological strengths and limitations** |
| --- | --- | --- | --- | --- | --- | --- | --- | --- | --- | --- | --- | --- |
| Pereira et al. (2011) | Yes | Yes | Somewhat | No | Yes | Somewhat | Somewhat | Yes | Can't tell | Yes | Yes | Clear aim, ethical approval, and transparent thematic analysis using *Atlas.ti.* Sampling strategy and consent procedures clearly described. Rich quotations provided, but limited reflexivity and minimal theoretical framing reduce transparency. |
| Paudel et al. (2014) | Yes | Yes | Somewhat | No | Yes | Somewhat | No | Yes | Somewhat | Somewhat | Yes | The study presents clear objectives and a culturally considerate approach; however, it falls short in analytical clarity and depth, offering only brief excerpts, lacking reflexivity and theoretical context. The absence of one transcript also compromises the study’s completeness |
| Atif (2015) | Yes | Yes | Yes | Somewhat | Somewhat | Somewhat | Somewhat | Yes | Somewhat | Somewhat | Somewhat | Well-designed and ethically conducted doctoral thesis with clear aims, rigorous analysis, and rich reflexive insight. Findings are well substantiated with participant quotations and contribute valuable knowledge on task-shifting in mental health care |
| **Author** | **Q1_**  **aims** | **Q2_**  **Methodology** | **Q3_**  **Design** | **Q4_Theoretical**  **Underpinnings** | **Q5_**  **Recruitment** | **Q6_Data**  **Collection** | **Q7_Researcher**  **Participant Relation** | **Q8_**  **Ethics** | **Q9_**  **Analysis** | **Q10_**  **Findings** | **Q11_**  **Value** | **Summary of methodological strengths and limitations** |
| Chibanda (2015) | Yes | Yes | Yes | Somewhat | Yes | Yes | Somewhat | Yes | Somewhat | Yes | Yes | Clear aims and appropriate qualitative design; data collection and ethical procedures are well described; findings are supported with quotes. However, lacks explicit theoretical/philosophical underpinning, reflexivity is underdeveloped, and data analysis is not fully transparent. |
| Atif et al. (2016) | Yes | Yes | Yes | No | Yes | Yes | Somewhat | Yes | Yes | Yes | Yes | Well-justified design and, triangulation, transparent analysis, though limited by lack of theoretical framework and partial reflexivity |
| Abas et.al. (2016) | Yes | Yes | Yes | No | Can't tell | Somewhat | No | Somewhat | Somewhat | Somewhat | Somewhat | Clear aims and ethical transparency, with reasonably described analytic methods and triangulated data sources, IDIs and FGDs, though limited by absent reflexivity, no theoretical framing, lack clarity on the recruitment strategy, and partially reported data capture procedures for FGDs or only noted by hand |
| **Author** | **Q1_**  **aims** | **Q2_**  **Methodology** | **Q3_**  **Design** | **Q4_Theoretical**  **Underpinnings** | **Q5_**  **Recruitment** | **Q6_Data**  **Collection** | **Q7_Researcher**  **Participant Relation** | **Q8_**  **Ethics** | **Q9_**  **Analysis** | **Q10_**  **Findings** | **Q11_**  **Value** | **Summary of methodological strengths and limitations** |
| Nyatsanza et al. (2016)* | Yes | Yes | Yes | No | Somewhat | Somewhat | No | Yes | Yes | Yes | Yes | Appropriate design and rigorous analysis with  rich, well-supported findings, but limited by absent theoretical framework, reflexivity, and partial sampling transparency |
| Pacichana-QuinayÃ¡z et al. (2016) | Yes | Yes | Yes | No | Somewhat | Somewhat | No | Yes | Somewhat | Somewhat | Yes | clear design and presentation of findings, but limited by lack of theoretical framework, absent reflexivity, unclear sampling decisions, and partial transparency in data collection and analysis |
| Atif et al. (2017) | Yes | Yes | Yes | No | Somewhat | Yes | No | Yes | Somewhat | Yes | Yes | Appropriate qualitative design with clearly described data collection and analysis, but limited by lack of theoretical framework, reflexivity, and justification for convenience sampling and research design. |
| Chibanda et al. (2017) | Yes | Yes | Yes | No | Somewhat | Yes | No | Yes | Somewhat | Yes | yes | Rigorous data collection and analysis processes with clearly presented findings, but weakened by lack of theoretical framework, absent reflexivity, and partial transparency in sampling and integration of participant groups. |
| **Author** | **Q1_**  **aims** | **Q2_**  **Methodology** | **Q3_**  **Design** | **Q4_Theoretical**  **Underpinnings** | **Q5_**  **Recruitment** | **Q6_Data**  **Collection** | **Q7_Researcher**  **Participant Relation** | **Q8_**  **Ethics** | **Q9_**  **Analysis** | **Q10_**  **Findings** | **Q11_**  **Value** | **Summary of methodological strengths and limitations** |
| Munodawafa et al. (2017)* | Yes | Yes | Yes | No | Somewhat | Somewhat | Somewhat | Yes | Somewhat | Somewhat | Yes | Appropriate design and detailed analytic process, triangulation of data sources but weakened by absent theoretical framing, limited reflexivity, and partial transparency in sampling and validation procedures |
| Munodafawa (2018) | Yes | Yes | Yes | No | Yes | Yes | No | Yes | Yes | Yes | Yes | Clear aims, appropriate design, and adequate data collection and analysis. Ethical approval obtained; findings supported by coherent quotes. However, lacks explicit theoretical underpinning, limited reflexivity, and insufficient detail on sampling rationale. |
| Musyimi et al. (2019) | Yes | Yes | Yes | No | Somewhat | Somewhat | No | Yes | Somewhat | Yes | Yes | Clear aim, ethical safeguards, and transparent thematic analysis with dual coding and NVivo. Findings are structured and supported by quotes. Limited detail on coding procedures and no reflexivity reduces overall transparency |
| **Author** | **Q1_**  **aims** | **Q2_**  **Methodology** | **Q3_**  **Design** | **Q4_Theoretical**  **Underpinnings** | **Q5_**  **Recruitment** | **Q6_Data**  **Collection** | **Q7_Researcher**  **Participant Relation** | **Q8_**  **Ethics** | **Q9_**  **Analysis** | **Q10_**  **Findings** | **Q11_**  **Value** | **Summary of methodological strengths and limitations** |
| Myers et al. (2019) | Yes | Yes | Yes | No | Somewhat | Somewhat | No | Yes | Somewhat | Yes | Yes | Clear research aims and appropriate design with integration of counselling into chronic disease care. Ethical safeguards and recruitment are well described. However, the paper lacks detail on data analysis procedures, does not discuss researcher reflexivity, and provides only minimal participant quotations, which reduces analytic transparency. |
| **Author** | **Q1_**  **aims** | **Q2_**  **Methodology** | **Q3_**  **Design** | **Q4_Theoretical**  **Underpinnings** | **Q5_**  **Recruitment** | **Q6_Data**  **Collection** | **Q7_Researcher**  **Participant Relation** | **Q8_**  **Ethics** | **Q9_**  **Analysis** | **Q10_**  **Findings** | **Q11_**  **Value** | **Summary of methodological strengths and limitations** |
| Joag et al (2020) | Yes | Yes | Yes | No | Somewhat | Somewhat | Can't tell | Yes | Somewhat | Somewhat | Yes | Clear research aim and relevant qualitative design. Thematic analysis is mentioned, and findings are supported by some participant quotes. However, the study lacks detail on sampling strategy, analytic procedures, and reflexivity, which reduces transparency and credibility. |
| Singla et al. (2020) | Yes | Yes | Yes | No | Somewhat | Yes | Somewhat | Somewhat | Somewhat | Yes | Yes | Clear aim, appropriate design, and useful insights into peer supervision in task-sharing models. Thematic analysis is described with some double coding and use of participant quotes. However, the study lacks reflexivity, provides limited detail on recruitment diversity, and does not fully explain how themes were developed or how distress was managed. |
| **Author** | **Q1_**  **aims** | **Q2_**  **Methodology** | **Q3_**  **Design** | **Q4_Theoretical**  **Underpinnings** | **Q5_**  **Recruitment** | **Q6_Data**  **Collection** | **Q7_Researcher**  **Participant Relation** | **Q8_**  **Ethics** | **Q9_**  **Analysis** | **Q10_**  **Findings** | **Q11_**  **Value** | **Summary of methodological strengths and limitations** |
| Jacobs (2020) * | Yes | Yes | Yes | Somewhat | Yes | Somewhat | Yes | Yes | Somewhat | Yes | Yes | Well-design master thesis with clear aim, appropriate methodology, and rigorous analysis guided by the Framework Approach. Reflexivity and potential bias were acknowledged and mitigated through independent interviewing and team supervision. Findings were well-supported by participant quotes. Minor limitations include a lack of critical discussion of contradictory data and theoretical assumptions. |
| Jacobs et al. (2021) * | Yes | Yes | Yes | No | Yes | Somewhat | No | Yes | Somewhat | Yes | Yes | Clear aim, appropriate methodology, and rigorous thematic analysis using the Framework Approach. Themes are well-supported by quotes, and ethical safeguards are strong. However, the study lacks discussion of reflexivity, does not describe researcher–participant relationships, and provides only limited detail on how interviews were conducted |
| **Author** | **Q1_**  **aims** | **Q2_**  **Methodology** | **Q3_**  **Design** | **Q4_Theoretical**  **Underpinnings** | **Q5_**  **Recruitment** | **Q6_Data**  **Collection** | **Q7_Researcher**  **Participant Relation** | **Q8_**  **Ethics** | **Q9_**  **Analysis** | **Q10_**  **Findings** | **Q11_**  **Value** | **Summary of methodological strengths and limitations** |
| Henrique et al. (2021) | Yes | Yes | Somewhat | No | Somewhat | Somewhat | No | Yes | Somewhat | Somewhat | Yes | Clear aim and appropriate qualitative design using triangulated data sources. Thematic analysis was conducted, and findings are supported by participant quotes. However, the study lacks reflexivity, provides limited detail on analytic procedures, and does not discuss theoretical underpinnings or recruitment challenges |
| **Author** | **Q1_**  **aims** | **Q2_**  **Methodology** | **Q3_**  **Design** | **Q4_Theoretical**  **Underpinnings** | **Q5_**  **Recruitment** | **Q6_Data**  **Collection** | **Q7_Researcher**  **Participant Relation** | **Q8_**  **Ethics** | **Q9_**  **Analysis** | **Q10_**  **Findings** | **Q11_**  **Value** | **Summary of methodological strengths and limitations** |
| Chau et al. (2021) * | Yes | Yes | Yes | No | Somewhat | Somewhat | No | Yes | Somewhat | Yes | Yes | Clear aim and appropriate qualitative design with thematic analysis and iterative coding. Participant quotes support the findings, and ethical procedures were followed. However, the study lacks reflexivity, provides minimal detail on how diverse participant data were integrated, and offers only limited discussion of sampling limitations and theoretical positioning |
| Rodriguez-Cuevas et al.  (2021) | Yes | Yes | Somewhat | No | Somewhat | Somewhat | No | Yes | Somewhat | Somewhat | Somewhat | Clear aim and appropriate qualitative design with use of narrative content analysis and participant quotes. Ethical procedures were described, and themes were well-aligned with the study aim. However, the study lacks reflexivity, offers minimal detail on analytic steps, and uses convenience sampling without strong justification, reducing overall transparency. |
| **Author** | **Q1_**  **aims** | **Q2_**  **Methodology** | **Q3_**  **Design** | **Q4_Theoretical**  **Underpinnings** | **Q5_**  **Recruitment** | **Q6_Data**  **Collection** | **Q7_Researcher**  **Participant Relation** | **Q8_**  **Ethics** | **Q9_**  **Analysis** | **Q10_**  **Findings** | **Q11_**  **Value** | **Summary of methodological strengths and limitations** |
| Saleem et al. (2021) | Yes | Yes | Yes | No | Somewhat | Somewhat | Can't tell | Yes | Yes | Yes | Yes | Clear research aim, appropriate qualitative design, and rigorous thematic analysis with supporting quotes and multi-analyst consensus. Ethical procedures were followed. However, the study lacks reflexivity, provides limited information on recruitment processes, and does not describe researcher–participant relationships or how interviews were conducted. |
| Wood et al. (2021) | Yes | Yes | Yes | No | Somewhat | Yes | No | Yes | Somewhat | Somewhat | Yes | The study presented clear research aims, appropriate methodology, and a clear thematic analysis process, including intercoder reliability. Findings are supported with quotes and provide practical insights into LHW empowerment and gendered challenges. However, the absence of reflexivity, theoretical framing, and strategies for managing contradictory perspectives lowers transparency and limits the study’s methodological depth |
| **Author** | **Q1_**  **aims** | **Q2_**  **Methodology** | **Q3_**  **Design** | **Q4_Theoretical**  **Underpinnings** | **Q5_**  **Recruitment** | **Q6_Data**  **Collection** | **Q7_Researcher**  **Participant Relation** | **Q8_**  **Ethics** | **Q9_**  **Analysis** | **Q10_**  **Findings** | **Q11_**  **Value** | **Summary of methodological strengths and limitations** |
| Davis et al. (2022) | Yes | Yes | Yes | Somewhat | Somewhat | Somewhat | No | Yes | Somewhat | Somewhat | Yes | The study uses grounded theory and triangulated data sources to explore task-sharing implementation in perinatal mental health. Thematic development is detailed, and findings are well-illustrated with quotes. However, reliance on random rather than theoretical sampling, lack of reflexivity, and limited discussion of disconfirming data reduce methodological transparency |
| Yator et al. (2022) | Yes | Yes | Yes | Somewhat | Somewhat | Yes | No | Yes | Somewhat | Yes | Yes | A well-structured qualitative study grounded in a relevant theoretical framework and using multiple data sources. Thematic analysis was clearly presented, with direct quotes illustrating key findings. However, the study lacks reflexivity, and the role of theory in data analysis is underdeveloped. Reporting on analytical decisions and researcher influence could have been stronger. |
| **Author** | **Q1_**  **aims** | **Q2_**  **Methodology** | **Q3_**  **Design** | **Q4_Theoretical**  **Underpinnings** | **Q5_**  **Recruitment** | **Q6_Data**  **Collection** | **Q7_Researcher**  **Participant Relation** | **Q8_**  **Ethics** | **Q9_**  **Analysis** | **Q10_**  **Findings** | **Q11_**  **Value** | **Summary of methodological strengths and limitations** |
| Selohilwe et al. (2023) | Yes | Yes | Yes | Somewhat | Yes | Yes | No | Yes | Somewhat | Yes | Yes | This process evaluation is grounded in a robust framework (CFIR), uses multiple stakeholder perspectives, and applies a structured analytical approach. Thematic findings are substantiated with participant quotes. However, the lack of reflexivity, limited detail on contradictory data, and absence of participant validation weaken transparency and reduce overall confidence |
| Kidia et al. (2024) | Yes | Yes | Yes | No | Somewhat | Yes | No | Yes | Somewhat | Yes | Yes | This study offers rich contextual insights into the feasibility and sustainability of a lay-delivered mental health intervention in rural Zimbabwe. It applies iterative data collection methods and thematic analysis with collaborative coding. However, limited reflexivity, lack of theoretical underpinning, and minimal attention to divergent data constrain its analytic depth and transparency |
| **Author** | **Q1_**  **aims** | **Q2_**  **Methodology** | **Q3_**  **Design** | **Q4_Theoretical**  **Underpinnings** | **Q5_**  **Recruitment** | **Q6_Data**  **Collection** | **Q7_Researcher**  **Participant Relation** | **Q8_**  **Ethics** | **Q9_**  **Analysis** | **Q10_**  **Findings** | **Q11_**  **Value** | **Summary of methodological strengths and limitations** |
| Ng'oma et al. (2024) | Yes | Yes | Yes | No | Somewhat | Somewhat | Somewhat | Yes | Yes | Yes | Yes | This study offers practical insights into the acceptability and feasibility of a peer-delivered psychosocial intervention for perinatal depression in rural Malawi. Thematic analysis is clearly described, and ethical safeguards are in place. However, the absence of a guiding theoretical framework, limited discussion of reflexivity, and lack of detail on data saturation and recruitment challenges reduce overall transparency |
| Ahmed et al. (2024) | Yes | Yes | Yes | Somewhat | Somewhat | Somewhat | Can't tell | Yes | Yes | Somewhat | Yes | The study offers valuable implementation insights grounded in the CFIR framework, with clearly structured themes supported by participant quotations. However, the study lacks transparency in recruitment, reflexivity, and data verification processes, slightly weakening overall confidence in the robustness of its qualitative reporting |
| **Author** | **Q1_**  **aims** | **Q2_**  **Methodology** | **Q3_**  **Design** | **Q4_Theoretical**  **Underpinnings** | **Q5_**  **Recruitment** | **Q6_Data**  **Collection** | **Q7_Researcher**  **Participant Relation** | **Q8_**  **Ethics** | **Q9_**  **Analysis** | **Q10_**  **Findings** | **Q11_**  **Value** | **Summary of methodological strengths and limitations** |
| Chau et al. (2024) | Yes | Yes | Yes | No | Somewhat | Yes | Somewhat | Yes | Yes | Yes | Yes | It offers well-structured, client-centred insights into the acceptability of a task-shifted intervention for depression in Vietnam. It demonstrates rigorous translation, coding, and use of NVivo software. However, limitations in theoretical grounding, reflexivity, and sampling transparency lower overall methodological confidence |
| Szajna et al. (2024) | Yes | Yes | Yes | No | Somewhat | Yes | Can't tell | Yes | Yes | Yes | Yes | credible and useful insights into the feasibility and acceptability of a BA intervention delivered by CHWs in India. Thematic analysis was conducted systematically using NVivo, and findings were clearly presented with participant quotes. Nonetheless, limitations such as the absence of a guiding theoretical framework, lack of reflexivity, and insufficient detail on saturation and CHW recruitment slightly reduce the confidence in methodological transparency |
| **Author** | **Q1_**  **aims** | **Q2_**  **Methodology** | **Q3_**  **Design** | **Q4_Theoretical**  **Underpinnings** | **Q5_**  **Recruitment** | **Q6_Data**  **Collection** | **Q7_Researcher**  **Participant Relation** | **Q8_**  **Ethics** | **Q9_**  **Analysis** | **Q10_**  **Findings** | **Q11_**  **Value** | **Summary of methodological strengths and limitations** |
| Burgess et al (2025) | Yes | Yes | Yes | Somewhat | Yes | Yes | No | Yes | Yes | Yes | Yes | The study demonstrated clear aims, appropriate qualitative design, and rigorous data collection and analysis. However, it lacked reflexivity and presented relatively short participant quotations, limiting deeper interpretive insight despite overall transparency and relevance. |
| Bah et al (2025) | Yes | Yes | Yes | No | No | Yes | No | Yes | Somewhat | Yes | Yes | The study had clear objectives, used appropriate data collection methods with diverse participants, and provided a systematic description of analysis using the CFIR framework. Findings were supported by participant quotes. However, the study lacked an explicit qualitative philosophical framework, did not discuss reflexivity, and provided limited detail on the qualitative sampling strategy. |
