# Supplementary material for: Delivering low-intensity psychological interventions in low- and middle-income countries: A systematic review and meta-ethnographic synthesis of lay health workers’ experiences
Source: Glob Ment Health (Camb). 2026 Jul 7;13:e157. doi: 10.1017/gmh.2026.10271 (PMC13433339; doi:10.1017/gmh.2026.10271)
Supplement: Supplementary file 1 [file S2054425126102714sup001.zip › Supplementary material 5 - Synthesis process.docx]

**Supplementary material 5**

**Synthesis process**

**Theme 1: Doing more than psychological therapy**

(Abas et al. 2016; Atif 2015; Chau et al. 2021; Chibanda et al. 2017; Joag et al. 2020; Munodawafa et al. 2017; Pacichana-Quinayáz et al. 2016; Paudel et al. 2014; Pereira et al. 2011; Wood et al. 2021)

| **First order construct** | **Second order construct** | **Translation** | **Third order construct** |
| --- | --- | --- | --- |
| *In every following session I used to ask her about her mood and daily routine. She used to tell me what she has done, how she has taken care of her baby. This makes me realise that she is following my suggestions. Moreover the improvement in her says it all. IDI-01PV (Atif, 2015)* | **Positive feedback reported or observed from mothers (Atif, 2015)**  Another factor important for the motivation of the PVs was the positive feedback reported or observed from mothers or their families. During each session the PV assessed the mother’s mood through recording her response on the mood chart (pictorial illustrations representing mood on a 5-point scale ranging from very sad to very happy). In addition, they relied on accounts given by the mothers and their family members about their general wellbeing. Almost all PVs reported noticeable improvement in the mothers’ mood because of the support they were receiving from them. In addition, their improved mood was both the cause and effect of their improved interactions with their family and better care of their diet and rest. | **A client-centred and flexible approach**  Atif (2015) showed how volunteers monitored mothers’ mood and daily routines, including how they cared for their babies, signalling that symptom change was tracked through everyday functioning, not only clinical scales. Abas et al. (2016) extended this by portraying interventions as patient-centred and flexible, sessions were tailored to women’s needs, circumstances, and preferred settings rather than rigidly following the manual. Pacichana-Quinayáz et al. (2016) similarly described LHWs helping patients manage anxiety through relaxation techniques while adjusting the pace and order of components to match different abilities and engagement levels. Chibanda et al. (2017) likewise highlighted how LHWs flexibly targeted depressive and anxious thinking, adapting cognitive strategies to patients’ contexts.  **Psychosocial approach, referral service, other additional services**  Other studies showed that this flexibility routinely drew LHWs into the socio-economic roots of distress. Pereira et al. (2011) described LHWs helping patients navigate financial problems, social services, and bureaucratic procedures, while Paudel et al. (2014) showed them coordinating hospital referrals and promoting activities such as exercise and yoga alongside counselling. Abas et al. (2016) further illustrated how LHWs sometimes took immediate action in crises linked to poverty, domestic violence, or HIV/AIDS, even using their own money to meet urgent needs. In Joag et al. (2020), one LHW not only supported a woman’s depressive symptoms but also intervened with her abusive husband and worked on family relationships. Chau et al. (2021) similarly depicted LHWs providing food, linking patients to community resources, and offering personalised encouragement, motivating, calming, and “inviting them to be happy.”  Wood et al (2021) illustrated that in areas with high mental health stigma, LHWs had to negotiate cultural and religious norms that discouraged engagement with psychological treatments such as counselling and behavioural activation. To overcome these barriers, they extended their work beyond manualised techniques, adapting therapeutic conversations, reframing symptoms, and using culturally resonant practices to build trust and reduce resistance | **Doing more than psychological therapy**  Across studies, LHWs described work that began with symptom-focused care but quickly expanded beyond it. Taken together, all studies translate into a shared picture of LHWs “doing more than psychological therapy”, combining symptom management with practical, relational, and social support to stabilise the conditions in which recovery can occur. |
| *‘We hear, talk, empower the patient. We do not tell them what to do (Abas et al., 2016)* | **Patient-focused and flexible approach (Abas et al., 2016).**  Both patients and LHWs highlighted the patient-centred and flexible nature of the intervention, whereby it could be tailored to patient needs, such as having sessions conducted in their homes or being able to self-refer |  |  |
| *‘She is the driver, and I am the leader… [I] get in then the driver drives (Abas et al., 2016)* |  |  |  |
| *‘By talking about why they are there, I open up their mind in that I try and get them to understand that they can get help (Abas et al., 2016)* |  |  |  |
| *So when you started work history and did relaxation techniques, we always remember to them measure their levels of anxiety and fear at home, and always told us: <I feel good since I’m talking><This really works>.* *Pacichana-Quinayáz et al. (2016)* | **Perceived changes with CETA intervention***.* **(Pacichana-Quinayáz et al., 2016)**  They also recognized in users a better ability to handle some anxiety symptoms and implement strategies learned. In the users’ words, these techniques provided them with satisfaction since they were able to anticipate symptoms or a severe anxiety response to an episode. |  |  |
| *All patients are different [...] if I start from first component until the sixth, possibly in that order works for you but will not work for another one* (Pacichana-Quinayáz et al., 2016) | **Characteristics and LPCWs performance**  Both municipalities referred to the flexibility of therapy or stiffness of it as a positive and negative factor, respectively. Some individuals admitted that the therapy was stiff but allowed for its implementation; others considered it difficult to adapt to an individual’s needs. In contrast, some felt that the therapy was flexible as they could devote more sessions to a particular component if users so desired. |  |  |
| *"LHW3 “So sometimes you start working on the kuvhura pfungwa and you don’t finish it up in one sitting, then you meet the client in the community or at the market place and all I say is – I’m thinking of you my child and I look forward to our next discussion” (Chibanda et al., 2017)* | **Kuvhura Pfungwa (Opening Up the Mind) (Chibanda et al., 2017)**  The LHWs indicated that the first session would take up to an hour and in some instances it would be longer than an hour or would be carried over to the following day at the clinic or as a home visit. Sessions appeared to be less rigid after the first initial visit. |  |  |
| *"LHW3 “I always start off by greeting and discussing general issues to make the client feel relaxed then I introduce the issue of their SSQ score being high and suggest to them they may have kufungisisa (thinking too much)…. Often they break down and cry when you suggest they have kufungisisa….”"(Chibanda et al., 2017)* | ***Kuvhura Pfungwa* (Opening Up the Mind)** (Chibanda et al., 2017)  "Key ingredients for successfully opening up of the mind (*kuvhura pfungwa*) included an ability to show empathy, connecting with the client, being able to reflect and summarize key issues, being non-judgmental and not being prescriptive or instructive" |  |  |
| *LHW6 “We have to find one problem to start with and this is the client’s job. So I briefly summarize then I just listen and encourage them to think and select one problem. Sometimes this takes time”. (Chibanda et al., 2017)* | The final component of the *kuvhura pfungwa* was described as the point where the client now had to decide which problem to focus on. It seemed from the LHWs that clients hardly ever presented with one problem and as a result it often was difficult for them to select a single problem to work with |  |  |
| *"A person would know that she has a problem, but not know the major problem. This session taught them to list their problems and know which problem is major. (Counsellor 2)"(Munodawafa et al., 2017)* | **Mechanism** (Munodawafa et al., 2017)  Counsellors felt that teaching “healthy thinking” and “problem solving” skills changed participant’s behaviour. |  |  |
| *Some (patients faced) social difficulties like financial problem which is mainly due to seasonal work, daily wages, and alcoholism. Another problem was patients not having proper documentation to apply for social schemes e.g. unregistered marriage, so a woman cannot apply for widow pension. So I found that there is no proper channel to improve their social difficulties and their problem remains same and this worsens their health condition. But I tried to give them information about various available schemes and how to follow the procedure and some even applied for it." [LHC] (Pereira et al. 2011)* | **Psychosocial interventions delivered by the Lay Health Counsellor** (Pereira et al., 2011)  the LHCs adopted a flexible approach of delivering psychoeducation, improving their skills in asking about suicidal ideas, and providing concrete information on welfare schemes and referral agencies to address social difficulties and reviewing progress on these issues in follow up appointments. |  |  |
| *“We will provide her counselling. We will listen whatever she tells us and we will keep that information confidential within us. We will open her a new way with cooperation. Even if she has left the Bachat Gat (Self Help Group) we will re-admit her and guide her. We will take her to hospital and we will start the medication after consulting with the doctor. We will tell her about the exercise and yoga.” (Paudel et al., 2014)* | **Operational Skills (Paudel et al., 2014)**  Sometimes participants expressed many management options at a time. At other times, one VHW mentioned one or two, prompting another VHW to mention further options. |  |  |
| *A case I found difficult is when the person needs assistance immediately. One day I actually used my money after realizing that the situation was really desperate.’ (LHW) ‘They come in thinking that any problem, no matter what it is we can intervene, so we are more like a social work office sometimes. We end up being that way- where people are displaced and want to come and get assistance (LHW) (Abas et al., 2016)* | **Challenges to implementation Socio-economic circumstances (Abas et al., 2016)**  Various potential challenges emerged. For example, despite the many benefits of the LHWs’ links to the community, this could also be testing. Most reported often being approached by community members with situations that they felt required their urgent attention and personal support. Relatedly, many patients face a host of desperate problems, such as poverty, HIV/AIDS and domestic violence |  |  |
| *In one family, the husband used to beat his wife, when I came to know about them, I visited them. I counselled them. Showed them the films on quarrels, addiction. Now fights between them have reduced to a large extent. (Champ60223M05) (Joag et al., 2020)* | **Impact and benefits of the intervention** *(Joag et al., 2020)*  The qualitative in-depth interviews indicated that persons in the community experienced reduced symptoms and improved social, occupational and family functioning for a variety of problems such as depression, domestic violence, alcohol use, and severe mental illness. Employment, and family functioning also improved. These were attributed to a combination of intervention components |  |  |
| *My job is to go to meetings, mobilize self-management. […] I said if she [patient] could not go, I was willing to try to relax with her, leading her to walk with a walking stick. Many times, when she lies down at home, she felt depressed, not wanting to contact anyone. […] So I often came to advocate for and motivate her. […]* *I calm them down, invite them to be happy, suggest we need to still live, then I ask them some questions. I approach the community and ask for support for the people to overcome their mental illness. I support them with everything, from a piece of cake, or rice. […] I approach and confide with such patients. Seeing me, they are very happy because there is no one to talk to. [SC]* **(**Chau et al., 2021) | **The role of social collaborators in the community (**Chau et al., 2021)  This social collaborator spoke of their unique position in the community and how their existing relationships resulted in people being more responsive, encouraging patients to seek help from their local social collaborators when they were ill, and also suggested that the existing relationships facilitated their ongoing work. This contributed to “the community have[ing] more confidence in social collaborators, strengthening their position in the community. |  |  |
| *When we visit [religious group] houses, they have temples and all and the patient and others are not ready to accept us and they said we don’t want your treatment and avoided us and they said our gods don’t like you coming here. But we convinced them and made a good relation with them and I even prayed at their home. (LHW, Female, 30 years) (Wood et al. 2021)* | **Social context (Religion) (Wood et al. 2021)**  Religious norms and beliefs also impacted LHWs’ work. Religious differences between the LHWs and patients were sometimes barriers to establishing trust. Religious and spiritual beliefs also created myths and stigma related to mental illness, which the stakeholders and LHWs had to overcome in order to deliver the interventions |  |  |

**Theme 2: Embedded in the community, precariously held by the system**

(Ahmed et al., 2024; Atif, 2015; Abas et al., 2016; Atif et al., 2017; Bah et al., 2025; Burgess et al., 2025; Chau et al., 2024; Chau et al., 2021; Chibanda et al., 2017; Henrique et al., 2021; Joag et al., 2020; Kidia et al., 2024; Ng'oma et al., 2024; Pacichana-Quinayáz et al., 2016; Szajna et al., 2024; Jacobs et al., 2021; Jacobs, 2020; Musyimi et al., 2019; Myers et al., 2019; Selohilwe et al., 2023; Saleem et al., 2021; Wood et al., 2021; Munodawafa et al., 2017; Nyatsanza et al., 2016; Ng'oma et al., 2024; Wood et al., 2021; Yator et al., 2022)

| **First order construct** | **Second order construct** | **Translation** | **Third order construct** |
| --- | --- | --- | --- |
| *“So because we are known as Ambuya Utano (Lady health worker) there is a certain level of trust we get from PLWH. We have lived here for more than 20 years so we are known because with most of them we see each other at the market, we have helped them in the past maybe during the cholera outbreak, or when we carried out a home visit to see if they had medication for the HIV illness…” (Chibanda et al., 2017)* | ***Kuvhura Pfungwa* (Opening Up the Mind) (Chibanda et al. 2017)**  Living in the community they worked in seemed to be an added benefit for the LHWs as they felt that this brought a certain level of respect and trust, making opening of the mind more possible. | **Community trust and recognition**  LHWs consistently described how deep community embeddedness underpinned their work. In Chibanda et al. (2017), decades of living alongside neighbours and participating in earlier public health campaigns created trust, particularly among people living with HIV. Similarly, Chau et al. (2021), Wood et al. (2021), and Yator et al. (2022) showed that LHWs’ long-term presence and prior community service meant they were already known, visible, and respected before mental health programmes began, making it easier to introduce both psychological and physical health interventions.  Moreover, being “local” did not just mean geographic closeness but shared social worlds. Atif (2015) illustrated how LHWs drew on their own experiences of hardship and family stress to position themselves as peers rather than outsiders. This authenticity, speaking the same language, knowing local struggles, sometimes sharing the same diagnoses, made community members feel understood rather than judged. In Chibanda et al. (2017), LHWs living with HIV described how disclosing their own status helped destigmatise discussions of mental health and encouraged clients to open up. Across these accounts, trust was not momentary but cumulative. Wood et al. (2021) showed how LHWs were affectionately addressed as Didi (“sister”), reflecting a familial closeness, while Yator et al. (2022) described clients seeking LHWs out in clinics or communities long after formal sessions had ended. Repeated contact and reliability signalled that LHWs would “keep coming back,” reinforcing their credibility. | **Embedded in the community**  Across studies, a shared construct emerges in which community recognition, built through shared histories and everyday presence, functions as a therapeutic resource that enables emotional openness, sustained engagement, and acceptance of psychological support. Yet the findings also complicate any simple assumption that “being local” is uniformly advantageous. Community embeddedness creates the conditions for deep, enduring relationships, but also exposes LHWs to pre-existing conflicts, expectations of material help, and fears about gossip or breaches of confidentiality. Trust therefore appears not as a given attached to locality, but as something continually negotiated through flexibility, emotional labour, and careful boundary work within dense community networks. In many cases, this hard-won, community-level legitimacy sits in tension with more precarious recognition from formal health systems, leaving LHWs strongly anchored “from below” but only weakly supported “from above |
| *They call us Didi [sister]. If anything happens in their own house or in the neighbouring houses then they call us. And they trust us so much that if they have any family matter or if their relative has some problem, then they call us. (LHW, Female, 32 years) (Wood et al., 2021)* | **Working with patients (Wood et al. 2021)**  once LHWs succeeded in reaching patients, their relationships were generally positive. They built trust and developed friendly rapport with their patients: |  |  |
| *"The advantage is that I am living in the area and loved by people so when I come, people welcome me very warmly. […] I also tell people that this is a national project that wants to bring health and wellness to the people in both spirit and body, so please allow me to ask some questions for this project. Please tell me so that I can evaluate and send to the senior level, if there is any problem for you in terms of mental health issues, there will be support. People are very honest and happy. [SC, Khanh Hoa]" (Chau et al., 2021)* | **The role of social collaborators in the community (Chau et al. 2021)**  This social collaborator spoke of their unique position in the community and how their existing relationships resulted in people being more responsive, encouraging patients to seek help from their local social collaborators when they were ill, and also suggested that the existing relationships facilitated their ongoing work. This contributed to “the community have[ing] more confidence |  |  |
| *When they were telling their problems, I used to tell them that I had similar circumstances, but God has helped me to overcome it. Time never stays the same, I was stressed when my children were young, now my tension is gone. Mothers used to benefit from my experiences. IDI-33PV (Atif 2015)* | **Experience of being a mother** (Atif 2015)  All PVs, except one, were mothers themselves and about a quarter revealed that they had experienced perinatal depression. These experiences provided the PVs with good understanding of the mothers’ issues and the ability to empathise with them. |  |  |
| *Mothers will only talk openly to someone who is locally based. It is a fact that only a local person can understand their problems because she has gone through similar problems. Here in village people have different circumstances and almost everyone is experiencing problems of some sort. IDI-33PV (Atif, 2015)* | **Acceptance of the PV and factors contributing to it > Ability to relate to the mothers > Being local (Atif, 2015)**  Geographical proximity to the mothers was another key factor facilitating acceptance. Like the mothers (see Section 6.1.2.1), PVs expressed advantages to being local, such as their social, cultural and linguistic awareness, their knowledge of the local community and even personal knowledge of the families that they would serve. PVs believed that this made the mothers feel at ease to express their issues. Additionally, their linguistic compatibility with the mothers enabled the latter to speak freely in their local dialect (Potohari). (Atif, 2015) |  |  |
| *They were same age as me, I was friendly with them. I told them that I have been through all this. I used to sit side by side with them, if they were sitting on floor I used to sit on floor, then they listened attentively to you and took things on board. These are poor rural people; they listen to you and regard you as their well-wisher. IDI-33PV (Atif 2015)* | **Acceptance of the PV and factors contributing to it (Atif, 2015)**  The PVs’ acceptability was enhanced through befriending with the mothers. The majority of these mothers were socially isolated and were experiencing loneliness (see Section 6.1.1.2). The PVs provided them the opportunity to develop new relationships and participate in social activities through attending group sessions. More than half of the PVs recalled forming a friendly relationship with the mothers, who were similar age to them, by not just having a purely professional relationship with them, but by befriending them, sitting alongside them, being friendly with their families and even sharing their own personal information |  |  |
| *"LHW2 “because I (LHW) also live with HIV when I show understanding (empathy), they are grateful and when I share my own experience this helps to further open up their mind.”"(Chibanda et al., 2017)* | **Kuvhura Pfungwa (Opening Up the Mind) (Chibanda et al., 2017)**  Connecting with clients came in several forms from touching the hand and other culturally appropriate parts of the body, offering a tissue paper to a crying client, and praying. It was not unusual for the LHWs to use their own life examples to help create rapport |  |  |
| *So, it was a very big problem at the beginning, but I am glad that as the sessions were proceeding, we became good friends; actually, we developed a rapport, and even until now, some still call ‘when are we meeting again?’ [Laughter]… (EO, age 24, CHV, Kariobangi). (Yator et al. 2022)* | **Motivation** (Yator et al. 2022)  The IPT-G sessions formed a part of the entry point to enlarging the scope of their social support and even after the intervention they continued to share their issues: |  |  |
| *So, we are also still doing follow-ups. Some even call by themselves, sometimes I call them, some even when they come for clinics, they just come looking for us which is good so, so they trust us. So, there is that trust, there is that friendship, there are so many benefits that came out of this, so I was just gladly sharing (EO, age 24, CHV, Kariobangi).(Yator et al. 2022)* | **Motivation (Yator et al. 2022)**  The CHWs were now able to connect easily with the adolescent mothers after bonding with them during the sessions: |  |  |
| *I was from a different village so they trusted me. They felt comfortable knowing that I didn’t know anyone in the village and therefore their information was safe with me. IDI-28PV (Atif, 2015)*  *A mother living in the same village as her PV might not pay much attention to what she is being told. Whereas if a PV is from a different village, she will think that she has come from far and acknowledge her better. Moreover if she is from same village, mother might or might not have good relationship with her. If the families have interpersonal conflicts, the PV might feel uncomfortable visiting them. IDI-PV05 (Atif, 2015)* | **Acceptance of the PV and factors contributing to it > Ability to relate to the mothers > Being local (Atif, 2015)**  However, some PVs felt that familiarity can reduce the ‘professional’ status of PVs, with less value given to their knowledge and skills. Moreover, sometimes a longstanding family feud might involve the PV and her client’s family, and in such cases it would be difficult, if not impossible, to fulfil this role. Lastly, the perceived risk of breach of confidentiality is also higher if the PV and the mother both reside in the same village. | **Community embeddedness**  community embeddedness emerged as a double-edged condition for LHW, patient relationships. Atif et al. (2015) showed that being “local” did not automatically guarantee trust: some mothers were reluctant to disclose sensitive domestic problems to LHWs from the same village, fearing gossip or damage to family honour, and preferred one-to-one home sessions over group formats where others might overhear. In this context, LHWs from neighbouring villages were sometimes perceived as more trustworthy precisely because they were outside local social networks and better able to guarantee confidentiality.  Similar tensions appeared in Jacobs (2020) and Ahmed et al. (2024), where pre-existing familiarity within the neighbourhood initially hindered openness. Youth in particular worried that personal disclosures could circulate within their social circles, leading to guarded participation and emotional withholding during early sessions. Here, community proximity intensified concerns about privacy, making trust something that had to be actively negotiated rather than assumed.  Other barriers further complicated the development of trusting relationships. Atif et al. (2015) described how unmet material expectations (such as the absence of financial or tangible support) led some clients to question LHWs’ intentions or disengage from the intervention altogether, while long-standing family feuds meant LHWs could be seen as intruders rather than helpers. Ahmed et al. (2024) likewise highlighted how LHWs invested significant time and emotional effort accommodating clients’ unpredictable schedules, yet still encountered minimal disclosure and limited engagement in early contacts. |  |
| *They do not share personal information during group sessions. In villages they believe that if others will find out about their domestic issues, they will gossip about it which will dishonour their family. They only shared such information with us at their homes. IDI-15PV (Atif, 2015)* | Almost all PVs’ appreciated the mixed method of delivery and expressed views similar to the mothers. Most PVs felt the groups facilitated learning from each other experiences and individual sessions allowed sharing personal experiences which could not be discussed in a group setting due to the fear of disclosing information and the concerns of bringing shame and embarrassment to their families. |  |  |
| *She demanded for some monetary help. She seemed more interested in money and asked me what will I give her and how otherwise are my visits helpful for her? I said that I will give you information about mother and child health, but she seemed more interested in tangible gains. IDI-PV05 (Atif, 2015)* | **Challenges in working as a peer volunteer > Lack of engagement > Lack of interest (Atif, 2015)**  The PVs described various reasons which they believed lead to the mothers’ lack of interest in the intervention. Some PVs felt that lack of familiarity with the talking therapy combined with the lack of material incentives made the intervention less attractive to some mothers. Another reason could be a mismatch of educational qualifications; for example one PV felt that her skills were undermined by two mothers who possessed higher educational qualifications than her, resulting in a lack of engagement. Some PVs felt that the lack of interest could also be the manifestation of some underlying issues such as their denial of mental health problems, time constraints or family pressures. However, at times these reasons were not disclosed to the PVs, with the mothers making excuses to avoid receiving sessions. |  |  |
| *Initially they were not engaging with me. Both were graduates and more educated than me. They made it clear to me that they were more qualified. IDI-15PV- (Atif, 2015)* |  |  |  |
| *Women do not express their concerns because of fear of information being disclosed. Often the nature of domestic issues is such that mothers feel disclosure will disgrace their family’s standing in the community and will lead to family confrontation or argument. IDI-01PV (Atif, 2015)* | **Challenges in working as a peer volunteer > Lack of engagement >Lack of trust (Atif, 2015)**  As discussed in Section 6.1.4.1, the PVs being local and working in close knit communities led to a perception of risk of disclosure of information to the person/s known both to the mother and her family. Most PVs felt, this preoccupied some mothers and created a barrier to their work. The PVs acknowledged mothers’ fears of information being relayed back to their families resulting in domestic conflicts or word being spread in the neighbourhood resulting in bringing shame and disgrace to the family. In both cases a mother would be held answerable to her in-laws. |  |  |
| *I went to her house twice but they didn’t like me coming to their house, especially her mother in-law because of our family feud. She used to think that I am coming to spy on them. IDI-33PV (Atif, 2015)* | **Resistance from mothers’ families (Atif, 2015)**  Lastly conflicts or strained relationships between a PV’s family and a mother’s could cause a barrier to the delivery of the intervention. Such feuds can be very long-standing and destructive to individual relationships. One mother refused to see her PV because her in-laws were in dispute with the PV’s in-laws, and therefore her intentions for coming to their home were viewed with suspicion. |  |  |
| *“…On the first session yes, they do [participate] but not much, but on the second session they have got stories…” (ID01,designated counsellor) (Jacobs, 2020)* | **Barriers and facilitators related to the implementation of the project MIND intervention > intervention characteristics (Jacobs, 2020)**  a few FBCs described how some patients appeared withdrawn and hesitant during the first session, which they ascribed to being the patients’ first counselling experience. However, the FBCs reported that this changed with patients engaging more as counselling progressed |  |  |
| *“The first session that’s where I get problems where I have to make the client to feel comfortable when talking to you because some of these clients [they] get to see you so trying to talk to them when they had seen you before it’s a barrier… I have seen [one client] around because we live in the same neighborhood.” (Ahmed et al. 2024)* | **Client Reticence and Confidentiality Concerns (Ahmed et al., 2024)**  All but one of the peer counselors revealed that clients are usually reticent or reluctant to confide in them during initial sessions. According to Neo, adolescents may fear that the counselors will disclose information shared during the counseling sessions with the doctors at the clinic. Two counselors, Tebogo and Mpho, suggested that clients may not feel comfortable confiding in counselors whom they already knew or have seen prior to the counseling sessions |  |  |
| *“In most cases it’s not every youth or everyone that’s free to talk to someone their age… so most of the time… the clients that we see here know us… Maybe it’s just the human mind… if I tell you something personal and I see you in public… automatically I will start thinking maybe you told someone or you are going to just say what I told you out and everyone hears it.” (Ahmed et al., 2024)* |  |  |  |
| *“…the first session I should think is the most difficult because this person doesn’t know whether to trust you or not because of how fast the information can travel nowadays. So, it’s a very difficult thing but as time goes, as the client [begins] to trust you, it’s fine. The problem is when you start the session” (Ahmed et al., 2024)* | Despite initial concerns regarding confidentiality and privacy, counselors agreed that client trust usually improves as the sessions progress |  |  |
| *“I once had a client who never really said anything… [he] wasn’t really opening up across all repeated sessions and we then ended sessions just like that, we didn’t cover much though he had a few list of things… there were like four or five problems and then some of them we didn’t go over them… So we only attended like two… we should be glad as a counselor that at least you have managed to cover other problems because maybe [there is] one problem [that] needs two sessions or three sessions.” (Ahmed et al., 2024)* | Oratile recommended an increase from six sessions to twelve, when necessary, to accommodate clients who may take longer to open up or have problems that take longer than six sessions to address. |  |  |
| *“The challenge is always like coming here and also rescheduling issues… because maybe I will come here knowing my client will be here at two o’clock and then at half past two they will be telling me ‘no, I have family issues’… and then we reschedule to a different date [to] accommodate my client with me now, and I have to make sacrifices for my client because I understand it’s one of the things that we were told about. That sometimes you are going to have difficulties that you are going to invade some of the personal, private time” (Oratile). (Ahmed et al., 2024)* | Several counselors implied that the initial procedures used to schedule clients for counseling sessions were barriers to Safe Haven implementation. At the beginning of the pilot, when counselors were scheduling their clients by appointment only, clients were missing their scheduled appointments or attending late. |  |  |
| **First order construct** | **Second order construct** | **Translation** | **Third order construct** |
| *In one family, the husband used to beat his wife, when I came to know about them, I visited them. I counselled them. Showed them the films on quarrels, addiction. Now fights between them have reduced to a large extent. (Champ60223M05) (Joag et al. 2020)* | **Impact and benefits of the intervention** (Joag et al. 2020)  persons in the community experienced reduced symptoms and improved social, occupational and family functioning for a variety of problems such as depression, domestic violence, alcohol use, and severe mental illness. Employment, and family functioning also improved. These were attributed to a combination of intervention components | **Programmatic-based support from health institutions:**  Visual tools, psychoeducation materials, stationary, training and role play, supervision, debriefing,  Across studies, institutional support emerged as a crucial scaffold that enabled LHWs to deliver care with confidence, but it was often partial and time-limited. Training and role-play were repeatedly described as turning abstract manuals into usable skills. In Jacobs (2020) and Szajna et al. (2024), LHWs said that “the role play really, really helps” and that practice sessions made them “more confident” screening and counselling mothers. For some, training was personally transformative; one provider in Atif (2015) reflected that it “helped [me] overcome [my] own depression,” while others felt it legitimised their role in the eyes of communities.  Supervision and structured “Help the Helper” spaces (Pacichana-Quinayáz et al., 2016; Jacobs et al., 2020, 2021) offered emotional containment and guidance for difficult cases, with LHWs emphasising that supervision “helps me” to see “where we did well” and “what to improve.” These forums reinforced good practice while quietly teaching self-care: as one counsellor noted, “I must care for myself, because if I do not care for myself, I am going to burn out” (Jacobs et al., 2021).  Practical resources, visual tools, locally translated scales, stationery, and films, also signalled institutional backing. Visual materials meant that “even the old mother-in-laws were able to understand” (Ng’oma et al., 2024), and having “all my stationery… I am ready to work” (Kidia et al., 2024) conveyed preparedness. | **Precariously held by the system**  Across studies, LHWs appeared simultaneously supported and sidelined. Training, supervision, and practical tools (such as stationery, visual aids, and translated screening instruments) enabled them to deliver care, but these supports were fragile, often project-bound, inconsistently replenished, and contingent on individual managers’ goodwill rather than system-wide guarantees. At the same time, LHWs’ work was structurally under-remunerated, with low or absent pay, broken promises around stipends, and out-of-pocket expenses leaving them to absorb both the emotional and financial costs of sustaining task-shifted mental health care. These vulnerabilities were compounded by unsafe or unstable working conditions, including long and sometimes risky travel and delivering care in insecure environments. Taken together, these accounts translate into a shared construct in which LHWs occupy the margins of health systems: reliant on personal negotiation rather than formal integration, their roles are tolerated more than embedded, and their work and wellbeing remain precariously held by the very systems they are expected to strengthen. |
| *At the bench, I explain to my client why I think they are depressed and I ask them if they have other challenges/problems other than the ones I would have asked. I then take my notebook and write down the client’s problems. And then I will go through the problems with the clients. I then ask her which of the problems causes her to be more depressed. They tell me and I write the problem on the FB card. I would have written down important information about the client already, like the date, client’s phone number, and my name. (VHW, female)* | **Clinical workflow and emphasis on documentation (Kidia et al., 2024)**  Both nurses and VHWs, however, emphasized medical documentation. Nurses felt that making sure that VHWs kept accurate and up-to-date records (via the Friendship Bench cards) was a priority and was the main way to supervise VHWs, rather than directly observing them. |  |  |
| *“…it was easy to explain to women through the pictures…even those who did not go to school understood it, even the old mother in-laws were able to understand the lessons through the pictures”.* (Ng'oma et al., 2024) | **Positive experiences during THPP delivery.** (Ng'oma et al., 2024)  The THPP sessions are structured and use illustrations. Most peer volunteers reported that while structure simplified the sessions’ delivery, the contextually relevant illustrations helped to explain things better and make it more relatable to the mothers: |  |  |
| *“We show them the picture of different family planning options like Depo for three months, pills for 28 days, or the implant for three years” (Burgess et al. 2025)* | **Current approaches to mental health and Family Planning (Burgess et al., 2025)**  HEWs provided details about their current approach to postpartum FP, noting that FP counseling typically happens at 42 days postpartum. HEWs described meeting mothers 1-on-1 and using visual aids to discuss different methods. |  |  |
| *During therapy they heard similar situations as they had to live [...] , so I think they were quite strong themes. We had a space called <Help the helper>; oxygenate is part of the team and able to talk to everyone about what it is overwhelming to me as a therapist. Pacichana-Quinayáz et al. (2016)* | **Characteristics and LPCWs performance**  Moreover, being aware of the emotional risks involved with LPCW exposed to stories about violence, respondents recognized difficulties they might have to handle in these difficult situations within therapies. However, they emphasized the supervisor support and the spaces where the team gathered for feedback as crucial for answering questions and allowing for emotional release. |  |  |
| *“…the role play really, really helps a lot. It is good to understand content, but role play that is what matters when you do this counselling intervention.” (ID15, dedicated counsellor) (Jacobs 2020)* | **Counsellors’ perceptions of the benefits of the project MIND training (Jacobs 2020)**  Role-playing stood out as one of the key training aspects that most FBCs thought was beneficial for assessing their new counselling skills and their readiness to implement them into practice. It provided them with opportunities to rehearse their new counselling skills while simultaneously familiarising themselves with the manualised nature of the intervention. |  |  |
| *I like the training…the trainers were friendly…the way they explained the content was very good. I didn’t experience any problems in understanding it. Through it I have learned a lot, which has helped me to overcome my own depression. 15PV. When xx (supervisor) accompanied me, mothers took me more seriously and shared their concerns more openly knowing that I have been properly trained and supervised. IDI-15PV (Atif 2015)* | **Good training supervision** (Atif 2015) |  |  |
| *“And then, in the supervision, everyone’s experiences counted, what they were going through [with the older adult participants] … Hence, I think we could get a little bit of all the experiences, you know?”.(Henrique et al. 2021)* | **Training and clinical supervision (Feeling supported and prepared)** (Henrique et al., 2021)  Health workers perceived the training received and the group supervision as opportunities to talk about difficulties and support their work. In addition, supervision was also perceived as a space to share experiences among health workers, learn from each other, and discuss difficulties related to the use of the PROACTIVE app during the home sessions. |  |  |
| *ASHA #3: Initially, we were concerned about administration of the EPDS. This was resolved during training by performing role play based on various scenarios from the training officers and later [by working with] the participants. (Szajna et al. 2024)*  *ASHA #5: After training, we were much more confident in screening through the EPDS and [doing] the intervention through role play. (Szajna et al., 2024)*  *ASHA #2: All ASHA workers need to be trained. These Activities were non-pharmacological and don’t involve any risk.*  *ASHA #10: If more ASHAs were trained … it is helpful in preventing PPD by regular BA activities.*  *(Szajna et al. 2024)* | All 24 participants completed the activities on their weekly Behavioral Activity Log, also suggesting high acceptability. Qualitative results from ASHAs and participants suggested enthusiasm for the training and intervention components.(Szajna et al. 2024) |  |  |
| *I felt good because, firstly, through this training, it gave us the confidence to go and talk to perinatal women in the community. Some homes nearly fell apart, but because we went and talk with them, with the help of God, they came back together. So, that in itself is a good thing … [ ]. After all, we told them that if they have this problem, it is not nice to lock themselves in their rooms. Sometimes, you “play music,” “watch movies,” or “dance,” which is good for their wellbeing. [MMSG 6] (Bah et al. 2025)* | **Appropriateness (Bah et al., 2025)**  The MMSGs expressed that the PST-FBI was congruent with their role’s expectations as volunteers. They were happy that the PST-FBI offered them skills and a structured way of supporting perinatal women that they never had before: listening, nonjudgmental, dealing with a difficult client, and problem-solving skills. According to one of them, the MMSG claimed that they were able to form a therapeutic alliance with the perinatal women and support them throughout the four sessions of the intervention: |  |  |
| *We followed what you trained us. We used to advise people, but quite different from your approach. You came and taught us a new method, that they should talk to us, and we should listen. And after they finished, we work on the problem disturbing them, so I think this is better. The method is good. When someone is talking about their issue, that is not backbiting. You are the one who said it, and you suggested how we should go about it through the brainstorming process, I am here just to guide. [MMSG 8] (Bah et al., 2025)* | **Fidelity (Bah et al., 2025)**  We evaluated MMSG adherence to protocol during the supportive supervision by reviewing their notes and discussing randomly selected cases. For each MMSG, we observed one of their sessions, noting the duration, adherence to activity order, joint activities, handout provision, next session scheduling and the relationship with the perinatal women. Feedback from participants was gathered to assess their engagement and the relevance of the content delivered. This comprehensive approach helped to ensure that the intervention maintained its integrity, while also providing insights into areas for improvement. A debrief with the MMSG followed each observed session. One MMSG described how she handled the PST-FBI with a particular perinatal woman: |  |  |
| *“The contents were good” one HEW noted. “The training was delivered with examples, and simple language we can understand.” (Burgess et al. 2025)* | **Feasibility of training (Burgess et al., 2025)**  In training, many mental health concepts seemed new to HEWs. HEWs asked for clarification on several terms, and the trainer provided examples to facilitate understanding. HEWs shared their own examples of encountering women with postpartum mental health challenges, suggesting they understood basic concepts and the content resonated with their current experiences. In interviews, HEWs described the training as engaging and relevant. “The contents were good” one HEW noted. “The training was delivered with examples, and simple language we can understand.” |  |  |
| *“The contents were good” one HEW noted. “The training was delivered with examples, and simple language we can understand.” (Burgess et al. 2025)* | **Feasibility of training (Burgess et al., 2025)**  HEWs appreciated the opportunity to learn more about mental health, with one noting, “I didn’t have good knowledge about mental health before, but now I’ve had good lessons from the training.” Although the HEWs developed some basic competencies through training, the HEWs and the trainer noted that two days were not enough for full mastery. Thus, the trainer provided coaching and supervision throughout implementation. |  |  |
| *“…we do a bit role-play also…so that is the one thing that stands out for me…when you put yourself maybe in a client’s position…or you change roles, so that gives you a bit of an idea of what you must do.” (ID03, designated counsellor) (Jacobs, 2020)* | **Perceived benefits of supervision and debriefing (Jacobs, 2020)**  The majority of FBCs found the role-playing exercises conducted during supervision as particularly beneficial. The FBCs indicated that at times they would get stuck during a counselling session while not knowing what to say or how to proceed. However, during supervision the counsellors would assume the role of the patient and role-play challenging counselling scenarios with the counsellor  supervisor. This facilitated experiential learning. By means of role-playing such scenarios, it enabled the FBCs to reflect on past situations and practice managing them differently while simultaneously preparing them to deal with such scenarios more effectively thereafter. This also enabled the FBCs to, in a figurative sense, walk in the shoes of their patients to gain an insider’s perspective from their patients. |  |  |
| *“The supervisor will listen to the recording and then he comes every week for supervision. So at least he will tell me this, this and this we must improve there and there. We did well here, very good, this and this. So, the supervision helps me…” (ID06, designated counsellor) (Jacobs, 2020)* | **Perceived benefits of supervision and debriefing (Jacobs, 2020)**  Initially, all FBCs were anxious about their sessions being audio recorded for supervision purposes. They indicated that this supervision approach was completely novel with none of them having any previous experience of this form of intensive clinical supervision. At first, counsellors felt that it would be used to check on their mistakes and for criticising their work. Despite some counsellors being anxious initially, the majority of counsellors expressed positive views about the counselling sessions being audio recorded to assess intervention fidelity and receiving feedback as part of supervision. After completing their first few counselling sessions and receiving fidelity feedback, most of the counsellors were able to adjust to this method of receiving feedback. Among the positive views, counsellors expressed that it helped them to identify the areas where they needed improvement and it motivated them to deliver the intervention to the best of their ability. |  |  |
| *“I think for me supervision is important because you can get help with whatever case or any difficult situations that we are facing and also to know where to work on. It is also nice to hear that, oh, you are doing good or you can work on that or maybe do not use that and so on. So that really, really, really helps a person.” (ID03, designated counsellor). (Jacobs et al. 2021)* | **Counsellors’ Perceptions of Clinical Supervision and Debriefing (Jacobs et al. 2021)**  All FBCs reported being largely satisfied with the supervision and debriefing they received, emphasizing its role in their professional development and for ensuring quality of counselling. Weekly supervision and debriefing enhanced both their counselling skills and their confidence in their ability to deliver the intervention. |  |  |
| *“It is very important, because before I used to take on the whole world. Now, I know that as I care for my client, I must care for myself, because if I do not care for myself, I am going to burn out.” (ID18, designated counsellor). (Jacobs et al., 2021)* | FBCs also valued the regular debriefing they received as part of their weekly supervision session, particularly in relation to prioritising self-care and preventing burnout. Regular opportunities for debriefing created a “safe space” to discuss difficult patient cases, personal issues that may be impacting on their counselling, issues around self-care, and strategies for coping with stress: |  |  |
| *I usually attended staff meetings and sometimes they would want to know about my work and how I am coping. It was then where I would communicate about patients and work. [This was helpful] because they would also motivate each other to start referring. (Counsellor5) (Selohilwe et al., 2023)* | **Counsellor organisationally integrated into facility** (Selohilwe et al. 2023)  Only half of the project-employed counsellors (four out of eight) reported they felt accepted organizationally as part of the facility staff (i.e. provided feedback regarding patients, were considered as staff members and included in staff meetings). Being organisationally integrated reportedly helped build their confidence, validate their role and encourage referrals from nurses for patients identified with depressive symptoms. | **Lack of recognition from health institutions, lack of integration into healthcare settings**  Across settings, LHWs’ accounts converged on a pattern of partial, fragile integration into formal health systems. In Wood et al. (2021), LHWs described working alongside more educated clinic staff who neither accepted their role nor coordinated with them, preferring to defer to professional doctors. Similar hierarchies appeared in Jacobs et al. (2021), where nurses were reported to dismiss LHWs’ contributions and treat them as peripheral to “real” clinical work.  Selohilwe et al. (2023) showed how this marginal status translated into everyday organisational struggles: LHWs had to negotiate for basic workspace, advocate for referrals, and repeatedly prove their worth before being treated as part of the team. Myers et al. (2019) extended this picture to managerial levels, highlighting how weak communication between NGOs and implementers and poor understanding of LHW roles fostered unrealistic expectations and operational confusion.  Musyimi et al. (2019) and Jacobs (2020) further illustrated the lack of structural anchoring, with LHWs and traditional birth attendants working without identification cards, reliable access to counselling rooms, or consistent institutional backing. |  |
| *What is left is the issue of making sure [they buy us] stationery when it is finished, so that we always have stationery. (VHW, male) (Kidia et al. 2024)* | **Clinical workflow and emphasis on documentation (Kidia et al. 2024)** |  |  |
| *What I like most about my work is that lets say a client comes to the Bench and I have all my stationary. Then I know I am ready to work since I have to write down stuff as part of my work. (VHW, female) (Kidia et al. 2024)* |  |  |  |
| *ASHA #8: The tool [EDPS] was in the local language which made it easy to screen the mothers. (Szajna., 2024)* | All 24 participants completed the activities on their weekly Behavioral Activity Log, also suggesting high acceptability. Qualitative results from ASHAs and participants suggested enthusiasm for the training and intervention components. (Szajna et al., 2024) |  |  |
| *There are [staff] in the clinics who are more educated than us. It’s difficult for them to accept us and what we do. It’s difficult for them to accept the change that we bring and it’s hard for them to blend in with us. They will not coordinate with us and they speak offensively about us. It’s also hard for other people to accept us since we are not highly educated and non-professionals. And they prefer doctors and professionals. (LHW, Female, 30 years) (Wood et al., 2021)* | **Supervision** (Wood et al., 2021)  LHWs valued the role of supervision and expressed that it had to be timely, frequent, and impartial in order to be effective. The quality of relationships between LHWs and their supervisors were a key factor in LHWs’ success. Most often, relationships were very positive. LHWs who had a good rapport and an egalitarian relationship with their supervisor often spoke more positively about the supervision they received. When LHWs did not have a good relationship with their supervisor or felt impacted by status differences, it affected their work negatively. |  |  |
| *“…Sometimes they [nurses] just say, ‘oh, this is the lay counsellor’, you know, you are just [irrelevant]”. (ID18, designated counsellor). (Jacobs et al., 2021)* | **Perceived Barriers and Facilitators to the Implementation of the Project MIND Intervention (Jacobs et al., 2021)**  Although more salient for designated FBCs, both types of FBCs explained how their role was assigned a low status within the healthcare facilities. Counsellors perceived their lack of status and authority as hindering their efforts to negotiate for private and a confidential space within which to deliver counselling. This was evident in the many disruptions that would occur during the counselling sessions when healthcare staff would often enter the room with a query or request. Both designated and dedicated FBCs felt that these interruptions reflected the low value that other health workers placed on their work, which contributed to feelings of marginalisation. |  |  |
| *[…] I didn’t have a good relationship with the facility manager […] she was not the kind of person who would want to know how is it going with my work, if I had space to work in and if I was fine. I had to stand up for myself to secure space to work in (Counsellor4) (Selohilwe et al., 2023)*  *[…] at first, they did not refer and I had to prove myself in order to have a working relationship with the sister. They didn’t see me as part of the facility- “no you are for depression and you are for PRIME”. It was difficult but I managed to pull through (Counsellor8) (Selohilwe et al., 2023)* | **Barriers (Counsellor and counselling activities not integrated into the facility)** (Selohilwe et al., 2023)  Half (n=4) the project- employed counsellors reported not feeling integrated into the facility and facility activities. This was reflected in not being given adequate space to work, not being included in staff meetings and generally feeling marginalized, with the counselling service not recognised as important. This had a negative impact on counsellors’ confidence and the programme generally |  |  |
| *There was some miscommunication because they [NGO] didn’t have a lot of understanding as to what was expected from us. So for future, it would be nice if the managers could be in one of the training sessions just to know what it is all about … to avoid confusion as to the time spent with patients. (designated CHW) (Myers et al., 2019)* | **Lessons for future implementation** (Myers et al., 2019)  According to CHWs, support from the PHC facility, other CHWs and the NGOs that employed them (where applicable) was critical for facilitating counselling delivery. In facilities where the designated and dedicated CHWs described facility staff as ‘supportive’ and ‘interested’, the counselling proceeded smoothly. However, in PHC facilities where staff seemed less interested, CHWs were often interrupted during counselling, impacting on the therapeutic alliance |  |  |
| *“It’s just terrible. We are sidelined as TBAs. We need identification cards to identify us when we take the pregnant mothers to the hospital.” (A 62-year-old TBA) (Musyimi et al., 2019)* | **Recognition and Appreciation of TBAs by the Healthcare System (Musyimi et al. 2019)**  The TBAs also said that they do not have any form of identification when they take the pregnant mothers to the hospital and felt a need to be recognized during the referral process |  |  |
| *“Sometimes I feel like…I do not know whether I am not accepted. I do not know. I do not know how can I put it, because sometimes I am doing counselling saying, “Eish [Sigh], I am not happy, [after being asked for the space by nurses].” (ID05, designated counsellor) (Jacobs, 2020)*  *“It is the space that we do not have, and you find that sometimes the nurses are demanding the room.” (ID05, designated counsellor) (Jacobs, 2020)* | **Barriers and facilitators related to the implementation of the project MIND intervention > Inner setting (Jacobs, 2020)**  Generally, the FBCs started working as home-based carers or community care workers and afterwards attained a training certificate to work as FBCs for NGO’s. This lowly status impacted their ability to negotiate space or advocate for privacy to deliver the project MIND counselling. Therefore, the FBCs felt that they were disrespected and treated as outsiders by some of the healthcare staff in the facilities. They also felt marginalised in the facility with no credence given to the important work they performed. |  |  |
| *“We will feel appreciated by having a structured group of TBAs where we can share, initiate something jointly and perhaps receive regular updates on upcoming community activities.” (Musyimi et al., 2019)* | **Recognition and Appreciation of TBAs by the Healthcare System.(Musyimi et al., 2019)**  The participants also stated that there are no regular meetings or forums for sharing knowledge or receiving updated information. An interview with a key informant in her mid-fifties revealed how the TBAs would feel appreciated if a recognized opportunity for having structured groups could be availed: |  |  |
| *…when you are in the session and now the patient is like, does not want to open up. And the patient has opened-up before but now you have so many distractions. Everyone is entering the room…there is people sitting on the side, so you can see, it is not that the patient does not want to open-up to you. There are too many distractions.” (ID18, designated counsellor) (Jacobs, 2020)* | the majority of FBCs identified culture (norms, values, and basic assumptions of a given organization) as a barrier. Counselling requires that privacy and confidentiality be maintained at all times which is in line with basic principles of client-centred care (Lamont-Mills, Christensen, & Moses, 2018). A lack of privacy and confidentiality was described by many FBCs as a challenge to providing client-centred counselling in these settings. During counselling sessions, healthcare staff would often enter the room to collect facility-related supplies such as files or medical supplies. During these interruptions, the counsellors had no choice but to pause the session in order to accommodate the staff. Despite designated counsellors having their own office in the facility compared to dedicated counsellors, counselling interruptions were consistent for both approaches. Counsellors felt that this interruption of the counselling sessions might have had an impact on the patient’s willingness to disclose personal information and fully engage in the counselling process. Moreover, not recognizing and prioritizing patients’ need for privacy by healthcare staff demonstrated the cross-cutting impact this had on the inner setting context (interruption of counselling sessions) (Jacobs, 2020) |  |  |
| *The mothers would have to wait too long at the clinic. If they get hungry or tired, they sometimes just give up and go home. So we need to be able to refer them straight to a psychologist. (HIV Community Health Worker 3)(Nyatsanza et al., 2016)* | **Clinic versus home visits (Nyatsanza et al. 2016)**  The study results present conflicting views on location of the proposed counselling, women in this particular community showed a preference for individual clinic-based counselling sessions. This would encourage them to leave their homes which in some cases exacerbated their depressive symptoms. |  |  |
| *Finding a venue at the clinics was hard. Sometimes we ended up doing sessions outside and that made other*  *mothers not attend sessions because she viewed it as unimportant because you cannot talk about something*  *serious outside. (Counsellor 2) (Munodawafa et al., 2017)* | **Context** (Munodawafa et al., 2017)  The counsellors reported facing challenges negotiating the clinic environment. When asked in the interviews to retrospectively rate the clinics in terms of being welcome, (0 being not welcome and 10 welcome), the majority of the counsellors gave Clinic A ratings which ranged from 0 to 5, indicating that they did not feel welcome at the clinic when the project started, while clinic B was rated 10 by all the counsellors, showing that the counsellors felt welcome |  |  |
| *I think for us [in] Kariobangi, we had a big challenge when it came to the venue of the meeting because sometimes you find we are here sometimes we are displaced at the tent, maybe the other tent is very dirty, sometimes we are in this other tent we come here displaced, so it was a very big challenge [cross talk] …. (EO, age 24, CHV, Kariobangi). (Yator et al., 2022)* | **Motivation (Yator et al. 2022)**  several CHWs acknowledged the lack of a designated space for mental health services as one of the limitations towards adopting IPT-G delivery to routine PMTCT services |  |  |
| *I have been working outside without a counselling room for a long time. I think that is one of the reasons patients don’t come back especially in winter because we have our sessions outside due to lack of space. (Counsellor2) (Selohilwe et al., 2023)* | **Barriers** (Space) (Selohilwe et al., 2023)  Lack of dedicated counselling space was discussed as one of the main barriers in the inner setting by all of the eight lay-counsellors interviewed. The counsellors below discuss how the space challenge frustrated them and compromised patient privacy. |  |  |
| *Yesterday it was raining, you will be wet and you start to talk to the client but you are dripping water. (VHW, female)"*  *We need raincoats in case it rains so that I can come to the clinic or cover myself. (VHW, female)*  *And also about airtime. Say someone got counselling then they return home, if something else comes up and [they] want to ask the counsellor about it, they will need airtime to communicate (VHW, female). (Kidia et al., 2024)* | **Feasibility > barriers (Kidia et al., 2024)**  Resource limitations in terms of protective clothing, office supplies, and transport made the intervention challenging in ways beyond accessibility for providers and patients alike. |  |  |
| *The money that we get is not significantly different from those who do not work… There should be a difference… Apart from our uniforms, we need bags to show that we are going to work.’ (LHW) (Abas et al., 2016)* | **Insufficient training and financial support (Abas et al. 2016)**  although most LHWs expressed a strong commitment to the intervention, two suggested a need for further financial reward | **Financial constraints**  Across programmes, financial precarity emerged as a shared mechanism keeping LHW roles fragile and undervalued. In Abas et al. (2016) and Atif et al. (2017), LHWs described how the absence of meaningful financial differentiation from non-LHWs undermined their status, with some reporting that “people ask us why we are going for this job if it does not pay us.” Rather than being recognised as part of the health workforce, their efforts were seen as irrational or expendable.  Jacobs et al. (2021) showed how expanding responsibilities without commensurate pay deepened this sense of injustice: “your load is increasing, your targets are increasing, but money-wise it is not increasing.” Ng’oma et al. (2024) echoed this, as volunteers realised that roles initially framed as supported positions were effectively unpaid, eroding trust and motivation.  Financial strain also shaped the practical feasibility of care. In Kidia et al. (2024), one LHW explained, “I use $2.50 a day,” despite receiving only a $2 allowance, illustrating how transport and field costs were effectively subsidised from personal income. Ahmed et al. (2024) captured the emotional dimension of this mismatch, with one worker reflecting, “I’ve invested so much in this program but the rewards are not necessarily meeting my expectations.” |  |
| *“People ask us why we are going for this job if it does not pay us. If we got monthly salary, then, it would be good. We will also be interested in working.” (Peer, India). (Atif et al., 2017)* | **Views on motivation of the peers** (Atif et al., 2017)  In Pakistan, peers were happy to work on a voluntary basis, however, in Goa, India, the peers expressed the need for some kind of incentive structure for this programme to be successful and to hold their interest. |  |  |
| *“…we do not want to sound like [we] always wants recognition in the form of finance, but that would also help. Because I mean, your load is increasing, your targets are increasing, but money wise it is not increasing.” (ID13, designated counsellor).* (*Jacobs et al., 2021)* | **Perceived Barriers and Facilitators to the Implementation of the Project MIND Intervention (*Jacobs et al., 2021)***  For a subgroup of FBCs, these feelings of being undervalued were reinforced by low levels of remuneration. In South Africa, FBCs are paid a stipend that is just above minimum wage. According to these FBCs, low remuneration decreased their motivation to provide the counselling service. Designated counsellors were particularly frustrated as their scope of work had increased without any adjustment to their remuneration. Many described situations where they felt that tasks were being ‘dumped’ on them without any recognition or reward for the work they do. |  |  |
| *“…Much as we were volunteers, we were supposed to be paid a certain amount of money, which would at least have contributed towards the cost of farming”. (Ng’oma et al., 2024)* | **Barriers to THPP delivery. *(Ng’oma et al., 2024)***  Some participants, peer volunteers and mothers reported HIV/AIDs stigma as a potential barrier to the THPP delivery and access. The stigma resulted from peer volunteers being perceived as Anti-Retroviral Therapy (ART) providers, who provide community care to HIV positive women. This raised concern among a few mothers. Another barrier to the THPP delivery reported by the peer volunteers was their dissatisfaction with the stipend they were receiving: |  |  |
| *“I am not financially stable” (Atujuna et al.) and “the money that we get is not even enough to support our own selves” (Obonye). (Ahmed et al., 2024)*  *“I’ve invested so much in this program but the rewards are not necessarily meeting my expectations.” (Ahmed et al., 2024)* | ***Lack of Financial Resources (Ahmed et al., 2024)***  *Several counselors emphasized the need for basic financial support, particularly more transport funding to carry out their job responsibilities. Three counselors expressed that their current pay was not enough to afford transportation to the location of the counseling sessions, especially with recent fare increases* |  |  |
| *“money is a problem… sometimes [the clients] will just pop up out from nowhere… Imagine if I get a call like now to come here… it’s gonna take me a long time, it’s gonna need money also. What if at that certain time I don’t have any money with me? You see that’s the kind of problem that we face” (Obonye). (Ahmed et al., 2024)* |  |  |  |
| *What I find difficult is the issue of the transport system … (VHW, female) (Kidia et al., 2024)*  *Those who trained us had said when we’ll get … uniforms, bicycles, $10 transport. And now we are in the field and transport allowance is $2 and I use $2.50 a day. (VHW, male) (Kidia et al., 2024)* | ***Accessibility (Kidia et al., 2024)***  *Both providers and service users felt the intervention was not easily accessible. They perceived that this was due largely to the practicalities of rural life. Many VHWs and service users bemoaned transportation issues and difficulty traversing a challenging terrain, often on foot, and sometimes in extreme weather. This was exacerbated by a lack of money for transport via privately owned taxis known as kombis or commuters.* |  |  |
| *"I remember I was going to visit the mother after she gave birth…On the day that I was planning to visit*  her, we saw skollies (thugs) when we were on the station and had to turn back. (Counsellor 3)" (Munodawafa et al., 2017) | **Context** (Munodawafa et al., 2017)  All the counsellors indicated that crime in the community made it dangerous to conduct home visits as they were worried about being robbed. | **Safety, Environmental Instability, and Accessibility of Service Delivery**  The physical and social environments in which LHWs operate often pose direct threats to their safety and hinder the accessibility of care. In Munodawafa et al. (2017), a counsellor recalled being forced to abandon a postnatal home visit due to the presence of local thugs (“skollies”), reflecting how violence and criminal activity can abruptly disrupt planned health outreach. Similarly, Wood et al. (2021) illustrated the dangers of working in areas with prevalent drug addiction and robbery. One LHW expressed reluctance to enter a high-risk neighborhood where wearing gold could provoke theft, emphasising the persistent threat of harm even during daytime visits.  Geographical and transport-related barriers further compound the difficulty of delivering and accessing care. Another LHW in Wood et al. (2021) described the arduous process of escorting patients from remote villages to clinics, involving multiple bus and rickshaw rides or long walks when transport was unavailable. These logistical burdens illustrate how infrastructural inadequacies and poor transportation networks impede both the providers’ ability to deliver care and the patients’ capacity to receive it. |  |
| *Bringing patients from there [their village] to here [the clinic] for treatment is very difficult. From there, we have to take two buses and one rickshaw. Now if it is us, seven kilometers, we will walk. If there is a two-wheeler we will get a ride, if not we will keep walking, then if there is someone with a rickshaw, someone known then… they will stop and take us. With so much difficulty we have to come here. (LHW, Female, 52 years) (Wood et al., 2021)* | **Travel (Wood et al. 2021)**  Most LHWs travelled to reach patients or get to a facility, using a variety of modes including walking, motorbike, rickshaw (three-wheeled multi-passenger vehicle), bus, or sometimes the office vehicle. Travel was sometimes time consuming and tiring for them |  |  |
| *I have an area in which there are many [drug] addicts, addicted a lot, meaning even during the daytime they are ‘into the addiction’. That is why, initially, I did not feel like going to that area. Even the people from the other area would tell me; madam you don’t go there. If you have to go there you don’t wear any gold because there are many robberies that take place there. (LHW, Female, 36 years) (Wood et al. 2021)* | **Environment** (Wood et al., 2021)  "The physical environment that LHWs worked in was also challenging, either because of weather (extreme heat or rain) or because of the safety or suitability of areas to conduct sessions" |  |  |

**Theme 3: Growing in the role**

(Chau et al., 2021; Munodawafa et al., 2017; Ng'oma et al., 2024; Davies et al., 2022; Atif et al., 2016; Henrique et al., 2021; Jacobs et al., 2021; Pacichana-Quinayáz et al., 2016; Rodríguez-Cuevas et al., 2021; Myers et al., 2019)

| **First order construct** | **Second order construct** | **Translation** | **Third order construct** |
| --- | --- | --- | --- |
| *I was worried if I would be able to follow the manual and not add my own stuff. I was worried if I would be able to help the client with the problem they had, but at the end I was able to do so much so that others would tell me which sessions they enjoyed. (Counsellor 2) (Munodawafa et al., 2017)* | **Implementation** (Munodawafa et al., 2017)  Barriers to implementation of the intervention included counsellor anxiety based on fear of deviating from the manual and stress of providing emotional support. In some cases counsellor fears were attributed to how the real life situation differed from the role plays used during training. | **Emotional burden of treatment fidelity**  Munodawafa et al. (2017) highlight how counsellors initially struggled with anxiety about deviating from the manual or not being helpful enough. This anxiety grew during supervisory observations, where the presence of a supervisor heightened the feeling of being scrutinised, thus undermining self-confidence. For some LHWs, pressure to deliver the intervention “correctly” led to more directive, even scolding, responses when patients did not complete CBT homework (Davis et al., 2022). These reactions reflected a strong sense of responsibility for patients’ progress, broader healthcare norms that equate firmness with care, and the strain of adhering to structured manuals when patients could not engage as the protocol assumed.  **Interplay of age, supervision and professional insecurity**  Generational differences also played a role in the challenges LHWs faced. Older LHWs often felt less confident in adapting to new therapeutic methods compared to their younger colleagues, who were perceived as more technologically skilled and quicker to learn (Chau et al., 2021). These anxieties were compounded by the pressure of performing under supervision, as seen in Munodawafa et al. (2017), where some LHWs felt insecure when being observed by superiors.  **Negotiating legitimacy and identity in the role**  Chau et al. (2021) show that some LHWs positioned themselves as inferior in relation to age and expertise, particularly when comparing themselves with younger health workers perceived as more talented and modern. Similarly, Atif et al. (2015) show how interactions with more educated patients could undermine LHWs’ confidence and authority, as social and educational hierarchies made them feel less legitimate in the helper role. Taken together, these studies suggest that LHWs’ early experiences were often shaped by self-doubt and by comparisons with more socially valued forms of expertise.  By contrast, Joag et al. (2020) reflects a different position. Here, the role of “Champion” had been internalised as part of the LHW’s sense of self, fostering fearlessness, confidence, and a stronger sense of purpose. Read together, these studies suggest that feelings of inferiority were not merely barriers to practice, but part of a broader process through which LHWs negotiated who they were in relation to the role. Over time, some moved from seeing themselves as inadequate or less authoritative than others toward inhabiting a more confident and purposeful identity as a credible helper.  **Progressive skill acquisition and growing clinical confidence**  LHWs also encountered challenges when dealing with complex cases. Ng’oma et al. (2024) and Kidia et al. (2024) described situations where LHWs were unsure how to progress with high-need clients and recognized the need to refer cases beyond their capacity. These instances illustrate the limits of role adaptability, requiring LHWs to balance persistence with professional judgment. Despite these early struggles, LHWs described a transformation in their roles, both in terms of emotional resilience and skill development. Many became more confident, autonomous, and respected in their communities (Henrique et al., 2021; Jacobs et al., 2021; Pacichana-Quinayáz et al., 2016; Rodríguez-Cuevas et al., 2021). | **Growing in the role**  Across diverse settings, LHWs’ experiences suggest that the role required more than the acquisition of technical skills. It involved an ongoing process of negotiating confidence, legitimacy, and identity in relation to patients, supervision, and wider social hierarchies. Growth was not only about learning how to deliver an intervention, but also about becoming able to inhabit the role of a credible helper despite initial insecurity, self-doubt, or perceived lack of authority. In this sense, the LHW role emerged as a dynamic and evolving experience, shaped by practice, reflection, relational encounters, and changing self-understanding. |
| *When I started doing counselling sessions I wished the supervisor would not be there in the room. It is your first time and the supervisor sitting there, writing down, you feel like you are making a mistake and not doing the right thing…. (Counsellor 3) (Munodawafa et al., 2017)* | **Implementation** (Munodawafa et al., 2017)  All counsellors reported feeling anxious at the beginning. However, three counsellors (1, 2 and 4) felt they lacked confidence to begin counselling due to inadequate training while the other three counsellors (3, 5 and 6) felt confident to begin counselling due to an adequate training period. The counsellors’ anxiety could have been exacerbated by the supervisor’s observation of sessions |  |  |
| **“***The last session you spoke about your likes and dislikes right? Where is your book? You do not write [your exercises]. I gave an example for you and you did not write. You see? I even said choose here all the way to this side. You see?.. I gave an example and even made brackets for you… You did not write. Write! Write! At all. Are you getting old? … Do not think you have escaped because you still have the book [at home]. I want that book and this one full for Session 5. Do you understand?” (Csr F, FA, S4).* (Davis et al., 2022) | **Counselling factors affecting quality of delivery (Davis et al., 2022)**  there were examples of counsellors scolding or reprimanding participants. The following example reveals visible frustration and scolding from a counsellor |  |  |
| *I think young people like you [referring to the interviewer] are very talented. Old person like me cannot be talented like you. In fact, people said that they would like to see the old doctor, because they are full of experience, but I think there is no experience because the things they have learnt in the past is different from now. Now when I went to big hospital (….), it is full of talented young doctors, I found that the youth is talented. [SC, Long An] (Chau et al., 2021)* | **Challenges for program scalability with social collaborators (Chau et al. 2021)**  older age was also associated with difficulties in acquiring the new knowledge required for the intervention. Thus, while age and the related experience was definitely an advantage in encouraging trust with communities, our study also suggests that scaling up the intervention might require the development of training programs that are adapted for older learners |  |  |
| *Initially they were not engaging with me. Both were graduates and more educated than me. They made it clear to me that they were more qualified. IDI-15PV (Atif et al, 2015)* | **Challenges in working as a peer volunteer > Lack of engagement > Lack of interest (Atif et al, 2015)**  The PVs described various reasons which they believed lead to the mothers’ lack of interest in the intervention. Some PVs felt that lack of familiarity with the talking therapy combined with the lack of material incentives made the intervention less attractive to some mothers. Another reason could be a mismatch of educational qualifications; for example one PV felt that her skills were undermined by two mothers who possessed higher educational qualifications than her, resulting in a lack of engagement. Some PVs felt that the lack of interest could also be the manifestation of some underlying issues such as their denial of mental health problems, time constraints or family pressures. However, at times these reasons were not disclosed to the PVs, with the mothers making excuses to avoid receiving sessions. |  |  |
| *I have prevented many widows and separated women from suicidal thoughts. I myself had such thoughts but I overcame them. (I would think) “How can I do such things? I am the Champion of the project!... people will say “She used to counsel us and now she herself had tried this!”. These thoughts changed my mind-set. Now (I) am fearless and I feel confident. (Champ60224M11)* (Joag et al. 2020) | **Impact and benefits of the intervention (Joag et al. 2020)**  The qualitative in-depth interviews indicated that persons in the community experienced reduced symptoms and improved social, occupational and family functioning for a variety of problems such as depression, domestic violence, alcohol use, and severe mental illness. Employment, and family functioning also improved. These were attributed to a combination of intervention components |  |  |
| *“When participant had lots of issues, it became difficult to move from one step to another. It took me some time to understand and successfully deliver it to high need participants…” (PV4) (Ng’oma et al. 2024)* | **Perceived feasibility of THPP delivery > Difficult experiences during THPP delivery (Ng’oma et al., 2024)**  A few peer volunteers found challenging to deliver intervention to mothers who were experiencing complex issues and had chronic symptoms while one mother stated that the intervention was complex initially: |  |  |
| *If I have a red flag, I cannot counsel the client. The pressure might be too much for me, so I need help from the nurses who can send the client to the hospital (VHW, female). (Kidia et al, 2024)* | **Clinical workflow and emphasis on documentation (Kidia et al, 2024)**  For nurses, when asked about their supervisory role of VHWs, many mentioned that this role included close monitoring to ensure VHWs are correctly implementing problem solving therapy and taking care of referrals of cases deemed too challenging for the VHWs. |  |  |
| *Every day there was more motivation work because I understood how to manage that component of trauma. [...] Then, because these work, I could use knowledge daily and family life.* | **Characteristics and LPCWs performance (Pacichana-Quinayáz et al., 2016)**  According to interviewees, there are some benefits for LPCW including the generation of income and relative economic stability, wellbeing, autonomy and mental health tools that strengthen the skills of individuals to develop their own work and extend the therapy benefits into their daily lives and family life. |  |  |
| *I live with my mom, and I used to fear making my own decisions before (becoming a CMHW). I would expect others to decide on my behalf, but not anymore. Now I make my own decisions and my mother and family respect them… I only studied until middle school, but now I am determined to major in psychology. I know this (job) made me fall for psychology, and I would not like to stop doing my work. You can help people not only by being a doctor or a nurse, but by being like me, some sort of a psychologist.* (Rodríguez-Cuevas et al., 2021) | **Impact in CMHWs and Community Care (Rodríguez-Cuevas et al., 2021)**  CMHWS value the empowerment and noticeable changes in self-confidence that came with the process of becoming a CMHW, and by learning a strategy that could help people living with mental health conditions: |  |  |
| *“It benefitted me because my husband also drinks [alcohol], so … I have given him my [intervention] book. And then it was very helpful, and he was interested …” (ID01, designated counsellor).* (Jacobs et al., 2021) | **Counsellors’ Perceptions of the Project MIND Training (Jacobs et al., 2021)**  Most FBCs described how being trained in MI-PST benefitted them personally. After seeing how the problem-solving method helped their patients, some of these counsellors applied the problem-solving method to resolve their own challenges and assist those around them (e.g. problematic alcohol use among family members). |  |  |
| *“I learned so much about role plays so that we can familiarise how the intervention should be like … from session one to session three.” (ID08, dedicated counsellor).* (Jacobs et al., 2021) | **Counsellors’ Perceptions of the Project MIND Training (Jacobs et al., 2021)**  The FBCs emphasised that the role-playing and skills rehearsal enhanced their confidence in their ability to deliver the MIND intervention. According to these counsellors, the skills rehearsal aspect of training provided them with opportunities to rehearse their new counselling skills while simultaneously familiarising themselves with the manualised nature of the intervention: |  |  |
| *CHW 1: “Of course! I am sure [that a CHW could deliver the intervention]”; CHW 3: “We were well trained; we can do this [intervention*]”. (Henrique et al., 2021) | **Capability of non-specialists (Feeling capable of delivering the intervention) (Henrique et al., 2021)**  In general, the health workers did not experience problems delivering the programme and felt welcomed by the older adult participants. Only one CHW (CHW6) mentioned that the older adult participants she visited, refused to participate in the psychosocial sessions because the CHW was not a mental health specialist |  |  |
| *CHW 8: “Today, even without the tablet and not being part of the intervention programme any longer, we can still offer some counselling to people.”;* (Henrique et al., 2021) | **Impact of the intervention in their work routine at the UBS (Professional impact) (Henrique et al., 2021)**  All health workers indicated that participation in the PROACTIVE programme had a positive impact on their work at the UBS, as they acquired new skills. |  |  |
| *CHW 6- “Everything that we learned, that opened our mind, that helped in our work was valid. Every day we learn something, we change our point of view… This [the intervention programme] came to intensify it, to change our view”.* (Henrique et al., 2021) |  |  |  |
| *I used to worry a lot. when I was doing the intervention then I realised that there are things we worry about that are not important…so it seems like I am helping someone else, and I am also helping myself. (dedicated CHW)* (Myers et al., 2019) | **Perceived benefits of the intervention (Myers et al., 2019)**  In addition, both the dedicated and designated CHWs appeared to benefit personally from this programme, describing how they now applied PST strategies to resolve problems in their own lives and limit negative thoughts: |  |  |

**Theme 4: The hidden cost of caring**

(Ahmed et al. 2024; Atif 2015; Burgess et al. 2025; Chau et al. 2024; Chibanda et al. 2017; Henrique et al. 2021; Jacobs et al. 2021; Munodawafa et al. 2017; Pacichana-Quinayáz et al. 2016; Wood et al. 2021)

| **First order construct** | **Second order construct** | **Translation** | **Third order construct** |
| --- | --- | --- | --- |
| *When they were telling their problems, I used to tell them that I had similar circumstances, but God has helped me to overcome it. Time never stays the same, I was stressed when my children were young, now my tension is gone. Mothers used to benefit from my experiences. IDI-33PV (Atif 2015)* | **Experience of being a mother** (Atif 2015)  All PVs, except one, were mothers themselves and about a quarter revealed that they had experienced perinatal depression. These experiences provided the PVs with good understanding of the mothers’ issues and the ability to empathise with them. | **Caring through shared pain**  The study by Atif et al. (2015) demonstrated how LHWs used self-disclosure based on their own experiences to empathise with patients and build strong relationships. By sharing personal experiences, LHWs created a foundation of trust and relatability, encouraging patients to engage openly.    Similarly, Chibanda et al. (2017) showed that LHWs used self-disclosure by sharing experiences, such as living with HIV, to show empathy and further encourage patients to open up emotionally.  Likewise, Pachichana-Quinayáz et al. (2016) highlighted the role of empathy in building patient trust, with LHWs using statements like “most of us are displacement victims,” showing shared traumatic experiences with patients. This approach, using self-disclosure as an “empathy hook,” was seen as a valuable way to engage patients in therapy. Nevertheless, these strategies could also become emotionally overwhelming, leading LHWs to seek a “Help the Helper” space for mutual support and reflection.  Ahmed et al. (2024) further explored how LHWs’ shared experiences with clients created both emotional closeness and internal strain. One LHW described how their client’s problems mirrored their own ongoing struggles. Although they attempted to refer the client to another counsellor, the client refused. The client was insisting on working only with them. This situation illustrates the complexity of therapeutic relationships grounded in shared experience, while it may foster trust and rapport, it can also place an emotional burden on the LHW, who must navigate personal  vulnerability alongside professional responsibility.  This emotional dissonance was further illustrated by another LHW who acknowledged that certain client narratives “hit close to home,” making it difficult to remain unaffected. Despite this, they felt a strong sense of responsibility to stay objective and continue providing support, even when it was personally triggering. Stockton et al. (in Ahmed et al., 2024) also echoed this emotional intensity, describing how specific conversations could "remind LHWs of their own problems" or "open up old wounds."  **When caring consumes the self**  LHWs’ accounts suggest that caring often became self-consuming, requiring them to suppress their own emotions during sessions, sacrifice personal time, and repeatedly prioritise clients’ needs over their own wellbeing. Ahmed et al. (2024) show how some LHWs had to conceal their own emotional distress in order to remain composed during counselling. One worker described making an excuse “to go drink water” to manage overwhelming feelings, while also “pretend[ing] like I wasn’t going through what [the client] is going through,” highlighting the strain of maintaining therapeutic presence while holding back personal emotion. This emotional containment was part of a wider pattern in which care extended beyond the session itself. In Wood et al. (2021), a female LHW described the cumulative demands of travel, counselling, and administrative work as overwhelming: “You are thinking as they are talking… it is too much, very horrible,” and characterised the role as “emotionally taxing,” suggesting that emotional and cognitive strain were embedded in the work itself.  This burden also extended into LHWs’ private time and everyday lives. Ahmed et al. (2024) describe how rescheduling appointments because of client cancellations or family problems often meant sacrificing personal time, something one LHW described as expected: “we were told… you are going to invade some of the personal, private time.” The same study also highlighted frustration when clients did not keep to agreed appointment times, with one LHW noting that “waiting an hour for a client is a problem” when other responsibilities also had to be managed. Burgess et al. (2025) similarly illustrated how care expanded in response to patients’ emotional needs, with one worker explaining, “I may spend more time on an emotionally stressed mother than a healthier one.” Over time, this ongoing extension of care could come at the expense of LHWs’ own wellbeing. In Munodawafa et al. (2017), one counsellor reflected that “we forgot to take care of ourselves… we have our own problems and need to heal first,” while in Jacobs et al. (2021), a designated counsellor recalled that caregiving had once led to emotional overload: “I used to take on the whole world.” Together, these accounts show that the hidden cost of caring lay not only in supporting others through distress, but also in how such care gradually eroded boundaries, consumed personal resources, and displaced attention from LHWs’ own needs. | **The hidden cost of caring.**  Across studies, LHWs’ accounts converge on a shared construct in which emotional labour operates as the often-unseen engine of task-shifted psychological care. Caring through shared pain shows how LHWs draw on personal histories of hardship and social proximity to build trust and therapeutic connection. Yet this closeness also carries costs. In when caring consumes the self, LHWs described suppressing their own emotions during sessions, extending care into personal time, and repeatedly prioritising clients’ needs over their own wellbeing. Over time, this combination of emotional containment, personal sacrifice, and self-neglect creates a cumulative burden that is rarely recognised.  This hidden cost is intensified by workload and role strain, as LHWs juggle counselling, community duties, and family responsibilities, often stretching time, energy, and emotional resources to their limits. For many women, care is further shaped by gendered risk: navigating harassment, safety concerns, and expectations of modesty and obedience when entering homes, travelling alone, or challenging abusive dynamics. Stigma and cultural or religious resistance add another layer, as LHWs absorb rejection, moral judgement, and suspicion during home visits while working to make mental health support locally acceptable.  Taken together, these sub-themes suggest that task-shifted psychological care depends not only on the redistribution of therapeutic tasks, but also on LHWs’ willingness to absorb emotional, relational, and sometimes physical risks so that others can access care. The very qualities that make them effective, empathy, availability, and community embeddedness, also expose them to harm. Emotional labour therefore emerges not as a peripheral issue, but as a core mechanism through which both care and cost are produced. |
| *“because I (LHW) also live with HIV when I show understanding (empathy), they are grateful and when I share my own experience this helps to further open up their mind.”"(Chibanda et al., 2017)* | ***Kuvhura Pfungwa* (Opening Up the Mind) (Chibanda et al., 2017)**  Connecting with clients came in several forms from touching the hand and other culturally appropriate parts of the  body, offering a tissue paper to a crying client, and praying. It was not unusual for the LHWs to use their own life examples to help create rapport. |  |  |
| *Most of us are also displacement victims, then it is also very easy to understand, I think I know how to create empathy with people and that gives them confidence. . Pacichana-Quinayáz et al. (2016)* | **Characteristics and LPCWs performance**  Respondents mentioned an important point within the CETA intervention: In a first contact, LPCW must make an effective “hook” which allows one to determine if participants are interested in attending therapy. Supervisors believe that LPCW should have communication skills, be resourceful, flexible and have enough creativity to conduct therapy. Many agreed that LPCW empathy was one of the main strengths, developed because of their traumatic experiences. |  |  |
| *During therapy they heard similar situations as they had to live [...] , so I think they were quite strong themes. We had a space called <Help the helper>; oxygenate is part of the team and able to talk to everyone about what it is overwhelming to me as a therapist. Pacichana-Quinayáz et al. (2016)* |  |  |  |
| *“there are scenarios where my client will be talking about [a] problem that I am basically going through right now. But when I try to refer him to another counselor, he doesn’t want that other counselor. He wants specifically to see me.” (Ahmed et al., 2024)*  *“say something that will remind [him] of [his] own problems” (Stockton et al.). (Ahmed et al., 2024).*  *when you have cases that hit close to home, sometimes it can happen that you [are] the best person to help the client… that’s the only reason why I didn’t refer it… I try my best to still be objective [and] help the clients find solutions.” (Ahmed et al., 2024)* | **Counselor Psychological Wellbeing (Ahmed et al., 2024)**  Counselors commonly reported challenges with experiencing the same hardships as their  clients which can compromise their ability to provide problem-solving therapy. One counselor shared that his clients would say something that will remind him of his own problems. These emotionally triggering experiences can make it difficult for counselors to continue with the therapy sessions and may therefore prompt an unwanted referral to another counselor. |  |  |
| *“I can make an excuse to go drink water” (Oratile) and “I had to pretend like I wasn’t going through what [the client] is going through” (Maphisa et al.). (Ahmed et al., 2024)* | **Counselor Psychological Wellbeing (Ahmed et al., 2024)**  Counselors mentioned ways in which they tried to conceal their emotions during the counseling sessions. These emotionally triggering experiences can make it difcult for counselors to continue with the therapy sessions and may therefore prompt an  unwanted referral to another counselor |  |  |
| *Because [during counseling] you are thinking as they are talking. And travelling is so much. After doing all this you come to [the facility], you sit and some other work of typing…So it is too much, very horrible. This I did not like…Yes, it is too much, yes. Work is often emotionally taxing. (LHW, Female, 37 years)(Wood et al., 2021)* | **Workload** (Wood et al., 2021)  Many LHWs spoke of feeling burdened by their workload, while a few said that they felt motivated despite having lots of work. |  |  |
| *“The challenge is always like coming here and also rescheduling issues… because maybe I will come here knowing my client will be here at two o’clock and then at half past two they will be telling me ‘no, I have family issues’… and then we reschedule to a different date [to] accommodate my client with me now, and I have to make sacrifices for my client because I understand it’s one of the things that we were told about. That sometimes you are going to have difficulties that you are going to invade some of the personal, private time” (Oratile). (Ahmed et al., 2024)* | **Scheduling Procedures (Ahmed et al., 2024)**  Several counselors implied that the initial procedures used to schedule clients for counseling sessions were barriers to Safe Haven implementation. At the beginning of the pilot, when counselors were scheduling their clients by appointment only, clients were missing their scheduled appointments or attending late |  |  |
| *“if we decided that we should meet with the client around one, then the client decides to come around two… it’s a problem because I do have other commitments outside there. So, waiting an hour for a client is a problem.” (Tebogo) (Ahmed et al., 2024)* |  |  |  |
| *“I may spend more time on an emotionally stressed mother than a healthier one.”(Burgess et al. 2025)* | **Current approaches to mental health and Family Planning (Burgess et al., 2025)**  HEWs described varying approaches for supporting mothers in their communities who seemed to be struggling emotionally. “The only thing we can do” one HEW explained, “is provide advice and make them feel strong.” Another noted, “I may spend more time on an emotionally stressed mother than a healthier one.” In a case where a mother had been abandoned by her husband, one HEW described collecting funds to support her. Overall, HEW were already well immersed in postpartum mental health and FP through their daily work, but had more tools and training specifically for Family Planning. |  |  |
| *We forgot to take care of ourselves as counsellors. We put a lot of focus on the clients. We have our own problems and need to heal first before we help other people... (Counsellor 4) (Munodawafa et al. 2017)* | **Implementation** (Munodawafa et al. 2017)  Three counsellors mentioned how providing emotional support to mothers reminded them of their own problems and the other three mentioned how counselling participants was emotionally taxing for them. |  |  |
| *“It is very important, because before I used to take on the whole world. Now, I know that as I care for my client, I must care for myself, because if I do not care for myself, I am going to burn out.” (ID18, designated counsellor). (Jacobs et al., 2021)* | **Counsellors’ Perceptions of Clinical Supervision and Debriefing (Jacobs et al., 2021)**  FBCs also valued the regular debriefing they received as part of their weekly supervision session, particularly in relation to prioritising self-care and preventing burnout. Regular opportunities for debriefing created a “safe space” to discuss difficult patient cases, personal issues that may be impacting on their counselling, issues around self-care, and strategies for coping with stress: |  |  |

| *“…say for example we are three and the one is being booked for Project MIND, the pressure is on the other two to work for that one.” (ID13, designated counsellor) (Jacobs, 2020)* | **Barriers and facilitators related to the implementation of the project MIND intervention (Jacobs, 2020)**  Designated counsellors expressed competing task demands (misalignment) as a barrier to implementing the project MIND intervention. These FBCs described having to oversee several chronic clubs, perform HIV testing and counselling, and complete their administration duties as some of their core duties. As delivering the project MIND intervention was an addition to their daily duties, these FBCs felt that the task demands were often too much to perform all their duties optimally. | **When the work never ends**  Several studies revealed that LHWs experienced significant workload strain as a result of integrating psychological interventions into their already full schedules. In Jacobs (2020), designated counsellors described how limited staffing meant the absence of one team member led to additional burdens on others: “we are three and the one is being booked for Project MIND, the pressure is on the other two.” Similarly, these LHWs juggled dual responsibilities delivering Project MIND while continuing to assist in psychiatric clinics highlighting blurred boundaries between intervention roles and routine duties.  Jacobs et al. (2021) further illustrated that while delivering an hour-long session felt meaningful, it also resulted in accumulated pressure, as colleagues had to compensate by attending to 20–30 other patients. Henrique et al. (2021) echoed this tension: while the programme was rewarding, LHWs acknowledged it was unexpectedly time-consuming and created conflict with their pre-existing tasks, described as an “extra burden” and a source of “friction” in day-to-day operations.  Burgess et al. (2025) showed that LHWs were already responsible for an extensive array of services, vaccination, family planning, first aid, and health education. The psychological programme, while seen as valuable, added to the complexity and volume of their duties. This convergence of responsibilities points to role overload and fatigue, suggesting that implementation demands must be balanced with feasible workloads for sustainable service delivery. |  |
| --- | --- | --- | --- |
| *“…On Wednesdays I am busy with mental illness [psychiatric clinic] with the psychiatrist and doctor…whereby I do not focus on Project MIND alone, but I also participate and assist also on the psychiatrist and doctor that is here.” (ID08, dedicated counsellor) (Jacobs, 2020)* | **Barriers and facilitators related to the implementation of the project MIND intervention > Inner setting (Jacobs et al., 2021)**  Dedicated counsellors reported enjoying their work and being able to focus on delivering counselling without feeling pressured to perform other tasks (alignment). During times when they were not busy counselling patients, they offered their assistance to other areas in the facility such as helping healthcare staff or administrative personnel |  |  |
| *“At the beginning it was fine but as time went by it was such a lot of people we had to see and we had to see our work as well. So, it was tiring, because an hour to sit with a person is a lot when you know my colleagues need to see now maybe 20 or 30 more people because I am busy in this project.” (ID11, designated counsellor). (Jacobs et al., 2021)* | **Perceived Barriers and Facilitators to the Implementation of the Project MIND Intervention (Jacobs et al., 2021)**  Designated counsellors expressed concerns about the degree to which they could align the delivery of mental health counselling with their current workflow. They described competing task demands (including the management of chronic clubs which involves health promotion, provision of HIV testing and counselling, and other administrative duties) and uncertainty about how to reorganise their work schedule to accommodate MIND counselling. Several FBCs felt overwhelmed by these multiple task demands and needed help to prioritise their other work demands so that they could perform their duties optimally.. |  |  |
| *“Yes, because it was an extra burden, wasn’t it? Our usual tasks never stopped, but it [the programme] was something, it left a good taste in my mouth…”. (Henrique et al., 2021)*  *“Much more, much more time than we have imagined... But it was good”; CHW 7: “I didn’t know it was going to be that much”. (Henrique et al., 2021)* | **Impact of the intervention in their work routine at the UBS (Institutional impact) (Henrique et al., 2021)**  The extra work associated with participating in  the PROACTIVE programme was a hot topic during the focus groups. The main complaint was related to the difficulty of managing the time to perform the routine tasks at the UBS with psychosocial sessions at home, all within working hours. The home sessions were added to their other tasks at the UBS. They also felt the time spent with the PROACTIVE programme (sessions and supervision) was more than what they expected initially. Health workers also complained about frictions with colleagues over the time they spent on the PROACTIVE programme. |  |  |
| *There were frictions, you know? Because we did not do other things, we ended up taking that time to do these other things we had not planned… So, there were divergences* (Henrique et al., 2021) | **Workload and responsibilities (Burgess et al. 2025)**  In the first interview, prior to Mothers Time training, HEWs were asked to describe their current workload or responsibilities. They described long days, busy schedules, including lots of engagement with pregnant women and women who recently delivered. “I provide services like vaccines, family planning, monitoring, first aid and health education” said one HEW, explaining that, “the program is vast and huge and has many activities within it.” One key responsibility for HEWs is engaging with women at multiple key touchpoints before and after a birth, providing services and counseling on breastfeeding, vaccines, infant health, FP, and more. “We see a mother seven or eight times” one HEW explained, noting that through this long-term engagement, “we can establish a strong relationship.” |  |  |
| *“I provide services like vaccines, family planning, monitoring, first aid and health education”“the program is vast and huge and has many activities within it.” (Burgess et al. 2025)* | *In the first interview, prior to Mothers Time training, HEWs were asked to describe their current workload or responsibilities. They described long days, busy schedules, including lots of engagement with pregnant women and women who recently delivered. “I provide services like vaccines, family planning, monitoring, first aid and health education” said one HEW, explaining that, “the program is vast and huge and has many activities within it.” One key responsibility for HEWs is engaging with women at multiple key touchpoints before and after a birth, providing services and counseling on breastfeeding, vaccines, infant health, FP, and more. “We see a mother seven or eight times” one HEW explained, noting that through this long-term engagement, “we can establish a strong relationship.”* |  |  |
| *“I may spend more time on an emotionally stressed mother than a healthier one.” (Burgess et al. 2025)* |  |  |  |
| *"When I discussed about working for this [program], at that time, my mother-in-law was alive. She stopped me. [She said,] “In our family, our daughters-in-law do not work.” (LHW, Female, 45 years)(Wood et al., 2021)* | **Social context** **(Gender)** (Wood et al., 2021)  Social and cultural norms negatively impacted LHWs’ work in a variety of domains, most prominently gender, social class, religion, and stigma about mental illness. | **Caring under gendered risk**  Several studies illustrate how entrenched gender norms and social stigma surrounding mental health present significant challenges for Lay Health Workers (LHWs) in delivering psychological interventions. In the study by Wood et al. (2021), LHWs reported facing resistance from their own families due to prevailing gender expectations, such as the notion that daughters-in-law should not work outside the home. Additionally, LHWs, particularly women, faced gender-based harassment from male patients, which created a hostile and unsafe environment for intervention delivery.  **Facing stigma at the doorstep**  Social stigma also influenced how mental health services were perceived by communities. LHWs described patients associating counseling with being "uneducated" or "mad," often leading to avoidance, dropout, or outright denial of mental illness (Wood et al., 2021; Chau et al., 2024). Others reported being treated with suspicion, akin to intrusive salespeople, or facing accusations that interacting with people with mental illness would result in their own "madness" (Wood et al., 2021).  Religious and cultural beliefs also posed obstacles; LHWs were rejected from households where spiritual or religious practices were perceived as incompatible with mental health treatment. In some cases, families preferred talismans or spiritual healing over medical care, attributing symptoms to possession rather than mental illness (Atif et al., 2015). The fear of social judgment and shame further deterred patients from accepting diagnoses or participating in sessions, reflecting deep-seated stigma and a lack of mental health literacy (Chau et al., 2024; Atif et al., 2015).  Collectively, these experiences demonstrate how gender roles, stigma, and cultural perceptions not only discourage service uptake but also undermine the credibility and safety of LHWs. These factors function as social barriers that are deeply embedded and difficult to address through training or technical solutions alone. |  |
| *"For me, the person I’m seeing is unwell, I don’t [care] whether it is men or ladies. But, things like that, he [the patient] pinched my lip and kissed. Those kinds of problems are there. (LHW, Female, 27 years) (Wood et al. 2021)"* | Female LHWs spoke about gender-based harassment and feeling unsafe or uncomfortable when performing their work, especially when travelling to remote areas or conducting home visits with male patients." |  |  |
| *There were one or two [potential patients] who were this, you know, this high class. And they felt that just talking is not solving their problem…they are saying this is for uneducated people. (LHW, Female, 57 years) (Wood et al. 2021)* | **Social context** **(Class and Caste)** (Wood et al., 2021)  Social class and the caste system impacted LHWs’ work in three ways. First, some upper-class community members were skeptical of the interventions. |  |  |
| *When we visit [religious group] houses, they have temples and all and the patient and others are not ready to accept us and they said we don’t want your treatment and avoided us and they said our gods don’t like you coming here. But we convinced them and made a good relation with them and I even prayed at their home. (LHW, Female, 30 years) (Wood et al. 2021)* | **Social context (Religion) (Wood et al. 2021)**  Religious norms and beliefs also impacted LHWs’ work. Religious differences between the LHWs and patients were sometimes barriers to establishing trust. Religious and spiritual beliefs also created myths and stigma related to mental illness, which the stakeholders and LHWs had to overcome in order to deliver the interventions |  |  |
| *Family members] started asking questions like ‘Why do you people come here?’ They said, ‘After looking at your bags, other stuff and your vehicle, the neighbors started asking questions about us, so we don’t want your treatment…’ They don’t want to make public or exhibit that their [family member] is having a problem. (LHW, Female, 37 years) (Wood et al. 2021)* | **Stigma and Mental Illness** (Wood et al., 2021)  Stigma about mental illness was a major issue that created barriers to the task-sharing interventions. First, it deterred potential patients from seeking care or receiving proper care. In addition, patients’ families were sometimes afraid of facing discrimination in response to their family members’ mental illness and stigma sometimes led them to impede the delivery of care. They would deny that their family member had a mental illness at all, or would not seek or agree to treatment. |  |  |
| *[They would act like] how we behave with the salesman when they come door to door; “No, no, no we don’t want, don’t want,” that way they would [say]. So a little… I would feel bad, are we going to their doors to beg I would feel. (LHW, Female, 37 years)(Wood et al., 2021)* |  |  |  |
| *[My] family may [think] “by mixing with mad people, she has become mad too. Her mind is gone too.” So when things like that come up, from family or from neighbors, at that time I have to make myself understand that if someone is calling me mad, am I mad? Or maybe I have to ignore them, thinking, how does it matter to me what they say. (LHW, Female, 28 years)* (Wood et al., 2021) | Finally, LHWs described a common myth that by working with people with mental illness, they could “catch” mental illness too: |  |  |
| *“In this [locality], when we find a client, that client is assigned a [social] collaborator. [Social collaborators] consult with the client following the PHQ-9 assessment, but that client [does not] want to accept that she has depression because she is scared that people around her [will] know about her [disease]. For example, a client… dropped out the program because she [didn’t] want to share that she has depression.” (Provider T, Ben Tre****) (Chau et al. 2024)*** | **Client’s health conditions (Chau et al., 2024)**  Stigma was identified by both clients and providers as a barrier to participation in the SSM intervention, although it was more frequently brought up by providers. Providers shared how not all clients were receptive of engaging with the SSM and providers once it was underway and how some clients dropped out of the intervention or were hesitant to open up to them because of the negative associations around depression. As one provider reported: |  |  |
| *From my point of view, maybe they were afraid of being interviewed. Many clients might think that I would [schedule an] interview with them to swear at them because they are crazy…” (Provider V, Da Nang)* (Chau et al., 2024) |  |  |  |
| *“In this [locality], when we find a client, that client is assigned a [social] collaborator. [Social collaborators] consult with the client following the PHQ-9 assessment, but that client [does not] want to accept that she has depression because she is scared that people around her [will] know about her [disease]. For example, a client… dropped out the program because she [didn’t] want to share that she has depression.” (Provider T, Ben Tre)* (Chau et al., 2024) |  |  |  |
| *She got upset when I told her that the assessment indicated that she has depression. She said that she is not mad and stopped me from coming in when I went for my next visit. 22PV* (Atif, 2015) | **Stigma of mental illness (Atif, 2015)**  The stigma of depression posed a major risk to the acceptability of the PVs who were working with the depressed mothers. During the training the PVs were made aware of the stigma of depression as a significant barrier to engagement and they were trained to deal with the issue sensitively. The majority of them were able to deal with the issue when it arose, avoiding unnecessary stress or embarrassment to the mothers, or resistance to the intervention. PVs discussed how they had tackled this issue, rather than how it had acted as a barrier. However in one case, a PV failed to address the issue sensitively, leading to a mother’s withdrawal from the study. |  |  |
| *They say that she (mother) is being possessed, so instead of medicines they go for talisman (spiritual treatment). 19LHW* (Atif, 2015) |  |  |  |
| *They will deny or worry about being unwell. This will impact their mood adversely. Moreover some people feel ashamed of it, believing that it is either their family or their circumstances to be blamed of… as poverty is the biggest cause of depression. IDI-07PV (Atif, 2015)* |  |  |  |

**References**

**Abas M, Bowers T, Manda E, Cooper S, Machando D, Verhey R, Lamech N, Araya R and Chibanda D** (2016) ‘Opening up the mind’: problem-solving therapy delivered by female lay health workers to improve access to evidence-based care for depression and other common mental disorders through the Friendship Bench Project in Zimbabwe. *International Journal of Mental Health Systems* **10**(1)**,** 39. <https://doi.org/10.1186/s13033-016-0071-9>.

**Ahmed CV, Van Pelt AE, Buttenheim AM, Poku O, Rice BM, Lowenthal ED and Brooks MJ** (2024) Implementation Determinants of Problem-Solving Therapy Delivered by Near-Peer Lay Counselors for Youth Living with HIV in Botswana: Lay Counsellor Perspectives. *Global Implementation Research and Applications* **4**(4)**,** 381-393. <https://doi.org/10.1007/s43477-024-00126-6>.

**Atif N** (2015) The acceptability of peer volunteers as delivery agents of a psychosocial intervention for perinatal depression in rural Pakistan: a qualitative study. Student thesis: PhD, University of Manchester.

**Bah AJ, Wurie HR, Samai M, Horn R and Ager A** (2025) Feasibility, acceptability and preliminary effectiveness of a culturally adapted nonspecialist delivery Problem-Solving Therapy: Friendship Bench Intervention for perinatal psychological distress in Sierra Leone. *Cambridge Prisms: Global Mental Health* **12,** e16. <https://doi.org/10.1017/gmh.2025.6>.

**Burgess S, Bitew T, Haile A, Souffrant J, Shattuck D, Van Lith LM, Moore J and Hendrickson ZM** (2025) The feasibility and acceptability of implementing simplified cognitive behavioral therapy approaches to support postpartum mental health and address associated social and behavioral barriers to postpartum family planning in Amhara, Ethiopia: a qualitative study. *BMC Women's Health* **25**(1)**,** 248. <https://doi.org/10.1186/s12905-025-03744-w>.

**Chau LW, Lou H, Murphy JK, Nguyen VC, Small W, Samji H and O’Neil J** (2024) Client experiences of a task-shifting supported self-management intervention for depression in Vietnam. *BMC Health Services Research* **24**(1)**,** 1563. <https://doi.org/10.1186/s12913-024-12036-2>.

**Chau LW, Murphy J, Nguyen VC, Lou H, Khanh H, Thu T, Minas H and O’Neil J** (2021) Lay social workers implementing a task-sharing approach to managing depression in Vietnam. *International Journal of Mental Health Systems* **15**(1)**,** 52. <https://doi.org/10.1186/s13033-021-00478-8>.

**Chibanda D, Cowan F, Verhey R, Machando D, Abas M and Lund C** (2017) Lay Health Workers' Experience of Delivering a Problem Solving Therapy Intervention for Common Mental Disorders Among People Living with HIV: A Qualitative Study from Zimbabwe. *Community Ment Health J* **53**(2)**,** 143-153. <https://doi.org/10.1007/s10597-016-0018-2>.

**Henrique MG, de Paula Couto MCP, Araya R, Mendes AV, Nakamura CA, Hollingworth W, van de Ven P, Peters TJ and Scazufca M** (2021) Acceptability and fidelity of a psychosocial intervention (PROACTIVE) for older adults with depression in a basic health unit in São Paulo, Brazil: a qualitative study. *BMC Public Health* **21**(1)**,** 2278. <https://doi.org/10.1186/s12889-021-12402-3>.

**Jacobs Y, Myers B, van der Westhuizen C, Brooke-Sumner C and Sorsdahl K** (2021) Task Sharing or Task Dumping: Counsellors Experiences of Delivering a Psychosocial Intervention for Mental Health Problems in South Africa. *Community Mental Health Journal* **57**(6)**,** 1082-1093. <https://doi.org/10.1007/s10597-020-00734-0>.

**Jacobs YA** (2020) The experiences of facility-based counsellors who provide a psychosocial intervention for mental illness in primary healthcare facilities in the Western Cape. Master Thesis, University of Cape Town.

**Joag K, Shields-Zeeman L, Kapadia-Kundu N, Kawade R, Balaji M and Pathare S** (2020) Feasibility and acceptability of a novel community-based mental health intervention delivered by community volunteers in Maharashtra, India: the Atmiyata programme. *BMC Psychiatry* **20**(1)**,** 48. <https://doi.org/10.1186/s12888-020-2466-z>.

**Kidia K, Machando D, Dzoro V, Chibanda D, Abas M, Manda E, Mutengerere A, Nyandoro T, Chawarika M, Majichi D, van Dijk JH and Jack H** (2024) Rural Friendship Bench: A qualitative study in Zaka district, Zimbabwe. *Social Science & Medicine* **348,** 116791. <https://doi.org/https://doi.org/10.1016/j.socscimed.2024.116791>.

**Munodawafa M, Lund C and Schneider M** (2017) A process evaluation exploring the lay counsellor experience of delivering a task shared psycho-social intervention for perinatal depression in Khayelitsha, South Africa. *BMC Psychiatry* **17**(1)**,** 236. <https://doi.org/10.1186/s12888-017-1397-9>.

**Musyimi CW, Mutiso VN, Nyamai DN, Ebuenyi ID and Ndetei DM** (2019) Integration of Traditional Birth Attendants into Mental Healthcare: A Multistakeholder Qualitative Study Exploration. *Biomed Res Int* **2019,** 8195267. <https://doi.org/10.1155/2019/8195267>.

**Nyatsanza M, Schneider M, Davies T and Lund C** (2016) Filling the treatment gap: developing a task sharing counselling intervention for perinatal depression in Khayelitsha, South Africa. *BMC Psychiatry* **16**(1)**,** 164. <https://doi.org/10.1186/s12888-016-0873-y>.

**Pacichana-Quinayáz SG, Osorio-Cuéllar GV, Bonilla-Escobar FJ, Fandiño-Losada A and Gutiérrez-Martínez MI** (2016) Common Elements Treatment Approach based on a Cognitive Behavioral Intervention: implementation in the Colombian Pacific. *Cien Saude Colet* **21**(6)**,** 1947-1956. <https://doi.org/10.1590/1413-81232015216.07062015>.

**Paudel S, Gilles N, Hahn S, Hexom B, Premkumar R, Arole S and Katz C** (2014) Impact of Mental Health Training on Village Health Workers Regarding Clinical Depression in Rural India. *Community Mental Health Journal* **50**(4)**,** 480-486. <https://doi.org/10.1007/s10597-013-9630-6>.

**Pereira B, Andrew G, Pednekar S, Kirkwood BR and Patel V** (2011) The integration of the treatment for common mental disorders in primary care: experiences of health care providers in the MANAS trial in Goa, India. *Int J Ment Health Syst* **5**(1)**,** 26. <https://doi.org/10.1186/1752-4458-5-26>.

**Selohilwe O, Fairall L, Bhana A, Kathree T, Zani B, Folb N, Lund C, Thornicroft G and Petersen I** (2023) Challenges and opportunities for implementation and dissemination of a task- sharing counselling intervention for depression at primary health care level in South Africa. *Int J Ment Health Syst* **17**(1)**,** 7. <https://doi.org/10.1186/s13033-023-00575-w>.

**Szajna A, Tekkalaki B, Nandagaon V, Udapi G, Sogalad M, Dandagi S, Kole U, Patil S, Raddi S, Short V and Kelly PJ** (2024) Feasibility and acceptability of a community health worker administered behavioral activation intervention for postpartum depression: a single arm pilot study from India. *Frontiers in Psychiatry* **Volume 15 - 2024**. <https://doi.org/10.3389/fpsyt.2024.1284674>.

**Wood S, Seevak E, Bhatia U, McBain R and Nadkarni A** (2021) “I will not step back”: A Qualitative Study of Lay Mental Health Workers’ Experiences in India. *Administration and Policy in Mental Health and Mental Health Services Research* **48**(4)**,** 695-706. <https://doi.org/10.1007/s10488-020-01102-6>.

**Yator O, Khasakhala L, Stewart G-J and Kumar M** (2022) Acceptability and impact of group interpersonal therapy (IPT-G) on Kenyan adolescent mothers living with human immunodeficiency virus (HIV): a qualitative analysis. *BMC Women's Health* **22**(1)**,** 240. <https://doi.org/10.1186/s12905-022-01807-w>.
