# Supplementary material for: Delivering low-intensity psychological interventions in low- and middle-income countries: A systematic review and meta-ethnographic synthesis of lay health workers’ experiences
Source: Glob Ment Health (Camb). 2026 Jul 7;13:e157. doi: 10.1017/gmh.2026.10271 (PMC13433339; doi:10.1017/gmh.2026.10271)
Supplement: Supplementary file 1 [file S2054425126102714sup001.zip › Supplementary material 6 - GRADE CERQual.docx]

**Supplementary material 5. The** **GRADE-CERQual assessment of confidence in qualitative evidence**

**Review question: What are the experiences of LHWs in delivering low-intensity psychological interventions for individuals with depression and anxiety in LMICs?**

1. **Doing more than psychological therapy**

| **Summary of review finding** | **Components** | **Explanation of GRADE‐CERQual assessment** | **GRADE-CERQual assessment of confidence in the evidence** |
| --- | --- | --- | --- |
| Across studies, LHWs described delivering core low-intensity psychological techniques (for example, psychoeducation, problem-solving, relaxation, healthy thinking), but their work routinely extended beyond manualised therapy in response to patients’ everyday realities. They frequently addressed social, family, and practical determinants of distress, helping patients navigate poverty and basic needs, link to services, manage family conflict or domestic violence, and stabilise living circumstances, often stepping into advocacy, mediation, and practical support roles.  This “doing more” was also shaped by cultural and religious contexts and high stigma, as LHWs adapted how they talked about distress, reframed symptoms, and used locally resonant approaches to build trust and sustain engagement. Overall, the finding suggests that effective LIPI delivery in LMICs is not confined to technique delivery alone but often depends on LHWs’ ability to integrate therapeutic work with flexible, context-responsive support. | **Methodological limitations** | **Moderate Concerns**  There are moderate concerns related to methodological limitations. These include limited theoretical transparency, minimal reflexive discussion of researcher–participant relationships, incomplete justification of sampling strategies, and variable reporting of analytic rigour (for example, handling of deviant cases or use of validation strategies). All contributing studies had clear aims, appropriate qualitative designs, and reported ethical procedures, but several recurring limitations were evident. Although some studies demonstrated strong analytic practices and data triangulation, these strengths were not consistent across the body of evidence. Taken together, these limitations justify moderate concerns regarding methodological quality. | **Moderate Confidence**  We downgraded by one level for methodological limitations, due to moderate concerns about limited theoretical transparency, reflexivity, and reporting of analytic rigour in several contributing studies. Coherence was strong, with a clear and consistent pattern across settings, and adequacy was supported by rich data from nine studies across multiple LMIC contexts. Relevance was subject to only minor concerns, as the contributing studies examined LHWs delivering LIPIs in LMICs that were largely focused on depression and anxiety, although a small number involved broader common mental disorder or mixed-condition populations. Taken together, this supports moderate confidence that LHWs routinely extend their work beyond structured psychological techniques to address broader practical, relational, and social needs. |
|  | **Coherence** | **Minor Concerns**  A minor nuance was that the degree of flexibility and role extension varied across programmes: some accounts described highly adaptive practice, whereas others depicted more manual-driven delivery. Nonetheless, the overarching pattern of moving beyond therapy to meet contextual needs remained consistent. There was a clear and coherent fit between the data and the review finding: across studies, LHWs’ accounts converged on a shared trajectory in which delivery began with symptom-focused techniques but routinely expanded into broader practical, relational, and social support, including referrals, welfare navigation, crisis assistance, and family involvement. |  |
|  | **Adequacy** | **Minor Concerns**  Nine contributing studies and rich quotations provide sufficient quantity and depth to support the main claim. Minor concerns relate to thinner coverage of some nuanced subcomponents (e.g., domestic violence, family conflict, and certain “social work” functions which appear as vivid examples but may come from fewer quotations than the broader flexible, patient-led support component) but these do not undermine the overall adequacy for this finding. |  |
|  | **Relevance** | **Minor Concerns**  The contributing studies were conducted in LMIC settings and examine LHWs delivering low-intensity psychological interventions, providing a close match to the review question and context of interest. Across contexts, LHW accounts consistently describe extending their work beyond manualised techniques to address practical, family, and social determinants of distress, such as welfare navigation, safety support, and family engagement, making the finding highly applicable to understanding real-world LIPI delivery. Minor concerns arise because a small subset of contributing studies involved broader common mental disorder or mixed-condition populations rather than depression/anxiety-only samples, introducing some indirectness relative to the review’s specific focus (Chibanda et al., 2017; Wood et al., 2021). In addition, programmes varied in how tightly LHW roles were bounded, with some models more explicitly incorporating social support functions. However, these nuances do not substantially reduce the relevance of the overall finding. |  |
| **Studies contributing to the review finding:**  (Abas et al., 2016; Atif, 2015; Chau et al., 2021; Chibanda et al., 2017; Joag et al., 2020; Munodawafa et al., 2017; Pacichana-Quinayáz et al., 2016; Paudel et al., 2014; Pereira et al., 2011; Wood et al., 2021) | | |  |

2. **Embeddedness in the community, precariously held by the system**

| **Summary of review finding** | **Components** | **Explanation of GRADE‐CERQual assessment** | **GRADE-CERQual assessment of confidence in the evidence** |
| --- | --- | --- | --- |
| The review finding “Embeddedness in the community, precariously held by the system” captures a central tension in LHW delivery of LIPIs: strong legitimacy “from below” (community recognition) but fragile legitimacy “from above” (formal system support). LHWs’ long-term community presence, shared histories, everyday visibility, and prior service, acts as a therapeutic resource, helping clients feel safe, understood, and willing to engage. However, “being local” also creates boundary strain and potential mistrust (for example, fears of gossip or confidentiality breaches, resurfacing conflicts, expectations of material help), so trust must be continually negotiated through flexibility and emotional/boundary work.  In parallel, formal support (training, supervision, tools and materials) can enable delivery and reinforce legitimacy, but is often project-bound, inconsistently available, and reliant on individual goodwill rather than system guarantees, alongside structural undervaluing through low or absent pay, broken stipend promises, out-of-pocket costs, and sometimes unsafe or unstable delivery conditions. Taken together, the finding suggests that LHWs may be highly recognised and relied upon in community life while remaining only weakly integrated and insecurely supported within health systems, leaving their role “precariously held” despite its importance. | **Methodological limitations** | **Moderate Concerns**  Across 25 contributing studies, we have moderate concerns regarding methodological limitations. While most studies used appropriate qualitative methods and generally reported data collection and analysis procedures, limitations recur around; limited reflexive reporting of researcher positionality/relationships, under-specified qualitative theoretical orientation, sampling approaches that may constrain variation (e.g., convenience or trial-driven recruitment, incomplete reporting of non-participation), and inconsistent reporting of credibility strategies such as handling contradictory cases or member checking. These issues may narrow the range of perspectives captured and reduce analytic transparency, but are partially mitigated by generally clear aims, systematic analyses, and frequent use of recorded interviews/translated transcripts and stakeholder triangulation. | **Moderate Confidence**  We judged confidence in this finding to be moderate. This reflects moderate concerns regarding methodological limitations across the 25 contributing studies (including limited reflexive reporting, under-specified theoretical orientation, and incomplete description of sampling and credibility strategies), alongside only minor concerns regarding coherence, adequacy, and relevance.  Taken together, these issues suggest that the finding is likely a reasonable representation of how LHWs are both trusted and embedded in communities yet remain precariously positioned within formal health systems, although future high-quality studies may refine aspects of this interpretation. |
|  | **Coherence** | **Minor Concerns**  Minor concerns relate to the interpretive step that links these two strands (embeddedness in the community, precariously held by the system) into a single “tension” construct, as some contributing studies speak more strongly to one side than the other; however, the overall explanatory account remains plausible and internally consistent. There is a clear and consistent fit between the data and the review finding. Across studies, community recognition is repeatedly evidenced through LHWs’ longstanding embeddedness, shared lived experience, and cumulative trust that enables openness and sustained engagement. The synthesis also coherently captures how community embeddedness can be double-edged, with confidentiality concerns, pre-existing conflicts, and fears of gossip complicating trust, while, in parallel, the data consistently show that formal system recognition and support are often fragile, characterised by limited integration, low status, inadequate resources, and financial and environmental constraints. |  |
|  | **Adequacy** | **Minor Concerns**  The finding is supported by a large body of evidence (25 studies contributing data) and includes rich first-order quotations and consistent second-/third-order constructs capturing both community-level legitimacy and system-level precarity. Reported LHW sample sizes indicate substantial participant coverage overall (≥273 LHWs across studies reporting numbers), including several moderate-to-large samples (e.g., Abas et al., 2016; Chau et al., 2024; Wood et al., 2021; Selohilwe et al., 2023). Minor concerns remain because some studies contributing to the finding have small LHW samples and some included papers do not clearly report LHW participant numbers in the extracted context. |  |
|  | **Relevance** | **Minor Concerns**  Minor concerns arise because a small subset of contributing studies involve contexts that are slightly less closely aligned with the review’s core population, for example mixed diagnostic groups (such as CMD including problem alcohol use, or broader mental health service delivery beyond depression/anxiety). In addition, one contributing study involved a mixed adolescent and young-adult recipient group (13–24 years) (Ahmed et al, 2024), and one study with mixed-condition populations (Wood et al., 2021) rather than samples focused exclusively on depression and/or anxiety introducing some indirectness; however, the theme’s central explanatory point, community recognition alongside precarious system positioning, remains applicable to LHW-delivered LIPIs in LMIC settings. |  |
| **Studies contributing to the review finding**  (Abas et al., 2016; Ahmed et al., 2024; Atif, 2015; Atif et al., 2017; Bah et al., 2025; Burgess et al., 2025; Chau et al., 2024; Chau et al., 2021; Chibanda et al., 2017; Henrique et al., 2021; Jacobs et al., 2021; Jacobs, 2020; Joag et al., 2020; Kidia et al., 2024; Munodawafa et al., 2017; Musyimi et al., 2019; Myers et al., 2019; Ng'oma et al., 2024; Nyatsanza et al., 2016; Pacichana-Quinayáz et al., 2016; Saleem et al., 2021; Selohilwe et al., 2023; Szajna et al., 2024; Wood et al., 2021; Yator et al., 2022) | | |  |

3. **Growing in the role**

| **Summary of review finding** | **Components** | **Explanation of GRADE‐CERQual assessment** | **GRADE-CERQual assessment of confidence in the evidence** |
| --- | --- | --- | --- |
| LHWs described growth as a gradual, experience-driven process, moving from initial uncertainty to increasing confidence, competence, and legitimacy in the role. Early in delivery, many relied heavily on manuals and supervision, feeling anxious about “doing it right,” scrutinised when observed, and unsure about managing sessions independently. With practice, supported by training, role-play, feedback, and repeated real-world encounters, they began to internalise techniques, make more confident decisions, and respond more autonomously to client needs while remaining within intervention guidance.  Growth was also shaped by challenge. LHWs described managing complex cases, negotiating interpersonal and social hierarchies, and balancing counselling with other work demands. The trajectory was not always linear: some accounts described frustration or more directive delivery under the pressure of manual adherence and performance expectations during early adaptation. Over time, however, many LHWs reported feeling more capable, more legitimate as helpers, and, in some cases, changed in how they understood themselves through the work. | **Methodological limitations** | **Moderate Concerns**  We have moderate concerns regarding methodological limitations. While all contributing studies used appropriate qualitative methods with clear aims and generally adequate ethical procedures, most did not report theoretical/epistemological positioning and many lacked reflexive discussion. Recruitment and data collection procedures were frequently only partially reported (e.g., limited discussion of non-participation or saturation; samples drawn from trial completers or selected deliverers), and analytic credibility strategies (e.g., handling contradictory cases, respondent validation) were inconsistently described. These limitations may influence how “growth” and professional development are interpreted and may under-represent more challenging trajectories | **Moderate Confidence**  We had moderate concerns about methodological limitations, including limited reflexivity, under-reported sampling and analysis procedures, and inconsistent use or reporting of credibility strategies, which may have under-represented more challenging or negative developmental trajectories. Coherence was good, with a clear, internally consistent pattern of LHWs moving from early anxiety and uncertainty toward greater confidence, autonomy, and a stronger sense of role identity, even where delivery was occasionally strained or directive. Adequacy was generally good, with 11 studies providing rich and varied accounts of skill acquisition and confidence-building, although some subcomponents (for example, generational dynamics and work–family tensions) were less thickly evidenced. Relevance concerns were minor: despite variation in programmes and role labels (for example, perinatal vs older adults; peer volunteers vs lay counsellors), all studies focused on LHWs delivering LIPIs for adult depression and/or anxiety in LMICs, closely matching the review question. |
|  | **Coherence** | **Minor Concerns**  Minor concerns relate to some evidence of strained delivery (for example, directive or scolding interactions) and uneven depth across certain subcomponents (such as generational or work–family tensions). However, these patterns reflect variability in the process of “growing in the role” rather than conflicting evidence, and do not undermine the overall internal consistency of the synthesis. There is a clear fit between the data and the review finding: across studies, LHWs describe an arc from early anxiety about manual adherence, supervision, and managing complex real-world cases toward increasing confidence, autonomy, and role identity, with several accounts also noting personal and family-level benefits |  |
|  | **Adequacy** | **Minor Concerns**  Minor concerns remain because some subcomponents, such as generational dynamics or balancing domestic responsibilities, are less consistently or less richly evidenced than the core elements of the finding. The finding draws on 10 contributing studies and is supported by detailed LHW accounts of skill acquisition, confidence-building, and shifts in perceived capability and professional identity. Sample sizes range from small-to-moderate qualitative samples (for example, n=5–18 in several studies) to a larger dataset (for example, Chau et al.: n=47 LHWs), which broadens the range of LHW perspectives and reduces uncertainty about the volume of contributing data. Overall, the evidence is rich and varied for the central pattern of role development, with multiple studies offering detailed accounts of how LHWs gain confidence and competence over time |  |
|  | **Relevance** | **Minor Concerns**  Minor concerns relate to variation across programme contexts, including perinatal and older-adult interventions, and to differences in LHW role labels and organisational arrangements, which may shape how growth is experienced. However, these studies still examined adult depression and/or anxiety LIPIs in LMICs and therefore remained closely aligned with the review question. Although role labels varied (for example, peer volunteers, community health workers, lay counsellors, and community collaborators), all participants met the review definition of LHWs. These differences may reflect variation in formal integration, supervision, and role expectations across settings, which could influence how confidence, competence, and role development were experienced. Overall, this introduced only minor indirectness and did not meaningfully reduce the applicability of the finding. |  |
| **Studies contributing to the review finding:**  (Atif et al., 2016; Chau et al., 2021; Davies et al., 2022; Henrique et al., 2021; Jacobs et al., 2021; Munodawafa et al., 2017; Myers et al., 2019; Ng'oma et al., 2024; Pacichana-Quinayáz et al., 2016; Rodríguez-Cuevas et al., 2021) | | |  |

4. **The hidden cost of caring**

| **Summary of review finding** | **Components** | **Explanation of GRADE‐CERQual assessment** | **GRADE-CERQual assessment of confidence in the evidence** |
| --- | --- | --- | --- |
| LHWs described a hidden cost to delivering LIPIs: care often depended on emotional closeness, with empathy and self-disclosure helping to build trust and relatability. Yet this same closeness heightened emotional exposure, especially when patients’ stories echoed LHWs’ own experiences, requiring ongoing emotional self-regulation to remain calm, composed, and therapeutically present despite inner distress.  This emotional demand extended beyond the session itself. LHWs described care as consuming personal time, energy, and attention as counselling was layered onto existing family, community, and clinical responsibilities. The burden was further intensified by unpredictable patient demands, stigma and rejection during home visits, and, in some settings, gendered safety risks such as harassment. Overall, the finding suggests that task-shifted counselling depends on LHWs’ ongoing capacity to absorb emotional, relational, and sometimes physical strain in order to sustain care, underscoring the need for structured support such as supervision, debriefing, workload protection, boundary guidance, and safeguarding measures. | **Methodological limitations** | **Moderate Concerns**  We have moderate concerns about methodological limitations**.** Although the contributing studies generally used appropriate qualitative approaches and most reported ethical procedures and clear presentation of findings with participant quotations, several recurring limitations were identified. These include limited reflexive reporting, under-specified qualitative/theoretical positioning, and sampling often embedded within feasibility/trial or implementation contexts, with inconsistent reporting of saturation and variable depth of emotional accounts. Analytic transparency was uneven in some studies (e.g., limited discussion of deviant cases or integration across participant groups). These limitations may shape how fully the “hidden cost” and emotional labour of caring were elicited and interpreted, but do not invalidate the finding given the overall breadth and consistency of the evidence base. | **Moderate Confidence**  We had moderate concerns about methodological limitations, including limited reflexivity, under-specified theoretical positioning, and trial-embedded sampling that may have constrained the depth and range of emotional accounts. However, the finding shows strong coherence, with consistent descriptions across studies of LHWs using self-disclosure and shared experience to build trust while managing emotional triggering, concealment of distress, role strain, and gendered safety risks. Adequacy and relevance concerns were minor, as twelve studies with 142 LHWs provided rich data from LMIC LHWs delivering LIPIs, directly addressing the emotional and relational burdens captured in this theme. |
|  | **Coherence** | **Minor Concerns**  Minor concerns relate to the breadth of the construct and some conceptual overlap with other barrier-focused findings; however, the integrative explanation remains plausible and internally consistent. The review finding demonstrates a clear and convincing fit with the underlying data. Across studies, LHWs consistently describe using shared experiences and self-disclosure to build trust, while simultaneously managing emotional triggering, concealing their own distress, and maintaining composure. The synthesis coherently traces a cumulative burden through time sacrifice, workload and role strain, and incorporates stigma and gendered safety risks as additional layers that intensify the emotional and relational costs of caring. |  |
|  | **Adequacy** | **Minor Concerns**  Minor concerns about adequacy. Ten studies with 142 LHWs provide a substantial quantity of data, including rich accounts supporting the core pattern of emotional labour and cumulative burden. Minor concerns relate to uneven depth across some subcomponents (e.g., safety/sexual harassment) and less coverage of mitigating or positive experiences compared with the dominant “cost” narratives. |  |
|  | **Relevance** | **Minor Concerns**  Minor concerns about relevance remain due to contextual and population variation in a small subset of studies. These included perinatal and postpartum programmes, one study involving young people aged 13–24 (Ahmed et al., 2024), and three studies with broader common mental disorder or mixed-condition populations rather than samples focused exclusively on depression and/or anxiety. One of these involved patients experiencing depression alongside alcohol-related problems (Jacobs et al.2021), while another involved broader CMD presentations (Chibanda et al., 2017; Wood et al., 2021). However, all of these studies still examined LHW-delivered psychological care in LMIC settings and contributed directly to understanding the emotional, relational, and practical demands of providing care. Overall, this introduced only minor indirectness and did not substantially reduce the applicability of the finding to the review question. |  |
| **Studies contributing to the review finding:**  (Ahmed et al., 2024; Atif, 2015; Burgess et al., 2025; Chau et al., 2024; Chibanda et al., 2017; Henrique et al., 2021; Jacobs et al., 2021; Munodawafa et al., 2017; Pacichana-Quinayáz et al., 2016; Wood et al., 2021) | | |  |

**References**

Abas, M., Bowers, T., Manda, E., Cooper, S., Machando, D., Verhey, R., Lamech, N., Araya, R., & Chibanda, D. (2016). ‘Opening up the mind’: problem-solving therapy delivered by female lay health workers to improve access to evidence-based care for depression and other common mental disorders through the Friendship Bench Project in Zimbabwe. *International Journal of Mental Health Systems*, *10*(1), 39. <https://doi.org/10.1186/s13033-016-0071-9>

Ahmed, C. V., Van Pelt, A. E., Buttenheim, A. M., Poku, O., Rice, B. M., Lowenthal, E. D., & Brooks, M. J. (2024). Implementation Determinants of Problem-Solving Therapy Delivered by Near-Peer Lay Counselors for Youth Living with HIV in Botswana: Lay Counsellor Perspectives. *Global Implementation Research and Applications*, *4*(4), 381-393. <https://doi.org/10.1007/s43477-024-00126-6>

Atif, N. (2015). *The acceptability of peer volunteers as delivery agents of a psychosocial intervention for perinatal depression in rural Pakistan: a qualitative study* University of Manchester].

Atif, N., Krishna, R. N., Sikander, S., Lazarus, A., Nisar, A., Ahmad, I., Raman, R., Fuhr, D. C., Patel, V., & Rahman, A. (2017). Mother-to-mother therapy in India and Pakistan: adaptation and feasibility evaluation of the peer-delivered Thinking Healthy Programme. *BMC Psychiatry*, *17*(1), 79. <https://doi.org/10.1186/s12888-017-1244-z>

Atif, N., Lovell, K., Husain, N., Sikander, S., Patel, V., & Rahman, A. (2016). Barefoot therapists: barriers and facilitators to delivering maternal mental health care through peer volunteers in Pakistan: a qualitative study. *Int J Ment Health Syst*, *10*, 24. <https://doi.org/10.1186/s13033-016-0055-9>

Bah, A. J., Wurie, H. R., Samai, M., Horn, R., & Ager, A. (2025). Feasibility, acceptability and preliminary effectiveness of a culturally adapted nonspecialist delivery Problem-Solving Therapy: Friendship Bench Intervention for perinatal psychological distress in Sierra Leone. *Cambridge Prisms: Global Mental Health*, *12*, e16, Article e16. <https://doi.org/10.1017/gmh.2025.6>

Burgess, S., Bitew, T., Haile, A., Souffrant, J., Shattuck, D., Van Lith, L. M., Moore, J., & Hendrickson, Z. M. (2025). The feasibility and acceptability of implementing simplified cognitive behavioral therapy approaches to support postpartum mental health and address associated social and behavioral barriers to postpartum family planning in Amhara, Ethiopia: a qualitative study. *BMC Women's Health*, *25*(1), 248. <https://doi.org/10.1186/s12905-025-03744-w>

Chau, L. W., Lou, H., Murphy, J. K., Nguyen, V. C., Small, W., Samji, H., & O’Neil, J. (2024). Client experiences of a task-shifting supported self-management intervention for depression in Vietnam. *BMC Health Services Research*, *24*(1), 1563. <https://doi.org/10.1186/s12913-024-12036-2>

Chau, L. W., Murphy, J., Nguyen, V. C., Lou, H., Khanh, H., Thu, T., Minas, H., & O’Neil, J. (2021). Lay social workers implementing a task-sharing approach to managing depression in Vietnam. *International Journal of Mental Health Systems*, *15*(1), 52. <https://doi.org/10.1186/s13033-021-00478-8>

Chibanda, D., Cowan, F., Verhey, R., Machando, D., Abas, M., & Lund, C. (2017). Lay Health Workers' Experience of Delivering a Problem Solving Therapy Intervention for Common Mental Disorders Among People Living with HIV: A Qualitative Study from Zimbabwe. *Community Ment Health J*, *53*(2), 143-153. <https://doi.org/10.1007/s10597-016-0018-2>

Davies, T., Lund, C., & Schneider, M. (2022). Implementation of a task-shared psychosocial intervention for perinatal depression in South Africa: A grounded theory process evaluation. *SSM - Mental Health*, *2*, 100056. <https://doi.org/https://doi.org/10.1016/j.ssmmh.2021.100056>

Henrique, M. G., de Paula Couto, M. C. P., Araya, R., Mendes, A. V., Nakamura, C. A., Hollingworth, W., van de Ven, P., Peters, T. J., & Scazufca, M. (2021). Acceptability and fidelity of a psychosocial intervention (PROACTIVE) for older adults with depression in a basic health unit in São Paulo, Brazil: a qualitative study. *BMC Public Health*, *21*(1), 2278. <https://doi.org/10.1186/s12889-021-12402-3>

Jacobs, Y., Myers, B., van der Westhuizen, C., Brooke-Sumner, C., & Sorsdahl, K. (2021). Task Sharing or Task Dumping: Counsellors Experiences of Delivering a Psychosocial Intervention for Mental Health Problems in South Africa. *Community Mental Health Journal*, *57*(6), 1082-1093. <https://doi.org/10.1007/s10597-020-00734-0>

Jacobs, Y. A. (2020). *The experiences of facility-based counsellors who provide a psychosocial intervention for mental illness in primary healthcare facilities in the Western Cape* University of Cape Town]. <https://open.uct.ac.za/items/aef15291-b807-49e7-b583-2ecf734b7617>

Joag, K., Shields-Zeeman, L., Kapadia-Kundu, N., Kawade, R., Balaji, M., & Pathare, S. (2020). Feasibility and acceptability of a novel community-based mental health intervention delivered by community volunteers in Maharashtra, India: the Atmiyata programme. *BMC Psychiatry*, *20*(1), 48. <https://doi.org/10.1186/s12888-020-2466-z>

Kidia, K., Machando, D., Dzoro, V., Chibanda, D., Abas, M., Manda, E., Mutengerere, A., Nyandoro, T., Chawarika, M., Majichi, D., van Dijk, J. H., & Jack, H. (2024). Rural Friendship Bench: A qualitative study in Zaka district, Zimbabwe. *Social Science & Medicine*, *348*, 116791. <https://doi.org/https://doi.org/10.1016/j.socscimed.2024.116791>

Munodawafa, M., Lund, C., & Schneider, M. (2017). A process evaluation exploring the lay counsellor experience of delivering a task shared psycho-social intervention for perinatal depression in Khayelitsha, South Africa. *BMC Psychiatry*, *17*(1), 236. <https://doi.org/10.1186/s12888-017-1397-9>

Musyimi, C. W., Mutiso, V. N., Nyamai, D. N., Ebuenyi, I. D., & Ndetei, D. M. (2019). Integration of Traditional Birth Attendants into Mental Healthcare: A Multistakeholder Qualitative Study Exploration. *Biomed Res Int*, *2019*, 8195267. <https://doi.org/10.1155/2019/8195267>

Myers, B., Petal, P.-W., Claire van der, W., Crick, L., Carl, L., John, A. J., Naomi, S. L., Christopher, B., Tracey, N., Peter, M., Dan, J. S., & Katherine, S. (2019). Community health worker-delivered counselling for common mental disorders among chronic disease patients in South Africa: a feasibility study. *BMJ Open*, *9*(1), e024277. <https://doi.org/10.1136/bmjopen-2018-024277>

Ng'oma, M., Atif, N., Meltzer-Brody, S., Chirwa, E., & Stewart, R. C. (2024). Piloting a psychosocial intervention for perinatal depression, the Thinking Healthy Programme-Peer delivered (THPP), in a primary care setting in Lilongwe District, Malawi. *PLOS Glob Public Health*, *4*(5), e0002128. <https://doi.org/10.1371/journal.pgph.0002128>

Nyatsanza, M., Schneider, M., Davies, T., & Lund, C. (2016). Filling the treatment gap: developing a task sharing counselling intervention for perinatal depression in Khayelitsha, South Africa. *BMC Psychiatry*, *16*(1), 164. <https://doi.org/10.1186/s12888-016-0873-y>

Pacichana-Quinayáz, S. G., Osorio-Cuéllar, G. V., Bonilla-Escobar, F. J., Fandiño-Losada, A., & Gutiérrez-Martínez, M. I. (2016). Common Elements Treatment Approach based on a Cognitive Behavioral Intervention: implementation in the Colombian Pacific. *Cien Saude Colet*, *21*(6), 1947-1956. <https://doi.org/10.1590/1413-81232015216.07062015>

Paudel, S., Gilles, N., Hahn, S., Hexom, B., Premkumar, R., Arole, S., & Katz, C. (2014). Impact of Mental Health Training on Village Health Workers Regarding Clinical Depression in Rural India. *Community Mental Health Journal*, *50*(4), 480-486. <https://doi.org/10.1007/s10597-013-9630-6>

Pereira, B., Andrew, G., Pednekar, S., Kirkwood, B. R., & Patel, V. (2011). The integration of the treatment for common mental disorders in primary care: experiences of health care providers in the MANAS trial in Goa, India. *Int J Ment Health Syst*, *5*(1), 26. <https://doi.org/10.1186/1752-4458-5-26>

Rodríguez-Cuevas, F. G., Valtierra-Gutiérrez, E. S., Roblero-Castro, J. L., & Guzmán-Roblero, C. (2021). Living Six Hours Away from Mental Health Specialists: Enabling Access to Psychosocial Mental Health Services Through the Implementation of Problem Management Plus Delivered by Community Health Workers in Rural Chiapas, Mexico. *Intervention Journal of Mental Health and Psychosocial Support in Conflict Affected Areas*, *19*(1). <https://journals.lww.com/invn/fulltext/2021/19010/living_six_hours_away_from_mental_health.10.aspx>

Saleem, S., Baig, A., Sajun, S., Bird, V., Priebe, S., & Pasha, A. (2021). A mixed methods exploration of the role of multi-family groups in community treatment of patients with depression and anxiety in Pakistan. *International Journal of Mental Health Systems*, *15*(1), 78. <https://doi.org/10.1186/s13033-021-00500-z>

Selohilwe, O., Fairall, L., Bhana, A., Kathree, T., Zani, B., Folb, N., Lund, C., Thornicroft, G., & Petersen, I. (2023). Challenges and opportunities for implementation and dissemination of a task- sharing counselling intervention for depression at primary health care level in South Africa. *Int J Ment Health Syst*, *17*(1), 7. <https://doi.org/10.1186/s13033-023-00575-w>

Szajna, A., Tekkalaki, B., Nandagaon, V., Udapi, G., Sogalad, M., Dandagi, S., Kole, U., Patil, S., Raddi, S., Short, V., & Kelly, P. J. (2024). Feasibility and acceptability of a community health worker administered behavioral activation intervention for postpartum depression: a single arm pilot study from India [Original Research]. *Frontiers in Psychiatry*, *Volume 15 - 2024*. <https://doi.org/10.3389/fpsyt.2024.1284674>

Wood, S., Seevak, E., Bhatia, U., McBain, R., & Nadkarni, A. (2021). “I will not step back”: A Qualitative Study of Lay Mental Health Workers’ Experiences in India. *Administration and Policy in Mental Health and Mental Health Services Research*, *48*(4), 695-706. <https://doi.org/10.1007/s10488-020-01102-6>

Yator, O., Khasakhala, L., Stewart, G.-J., & Kumar, M. (2022). Acceptability and impact of group interpersonal therapy (IPT-G) on Kenyan adolescent mothers living with human immunodeficiency virus (HIV): a qualitative analysis. *BMC Women's Health*, *22*(1), 240. <https://doi.org/10.1186/s12905-022-01807-w>
