# Supplementary material for: Delivering low-intensity psychological interventions in low- and middle-income countries: A systematic review and meta-ethnographic synthesis of lay health workers’ experiences
Source: Glob Ment Health (Camb). 2026 Jul 7;13:e157. doi: 10.1017/gmh.2026.10271 (PMC13433339; doi:10.1017/gmh.2026.10271)
Supplement: Supplementary file 1 [file S2054425126102714sup001.zip › Supplementary material 7 - Audit Trail of Synthesis Revisions.docx]

**Impact of Excluding Giusto et al. (2021) and Verhey et al. (2019) on the Meta-Ethnographic Synthesis**

Following careful re‑examination of the eligibility criteria during the revision process, and in response to reviewer and editor suggestions, we re‑assessed studies involving mixed conditions (e.g., mental illness, depression, anxiety, alcohol use, and trauma). We excluded two studies from the final synthesis (Giusto et al. 2021; Verhey et al. 2019). Giusto et al. (2021) was excluded after author clarification confirmed that depression was not an eligibility criterion and depressive symptoms were assessed only as an outcome. Verhey et al. (2019) was excluded because the qualitative sample was specifically selected for trauma/PTSD-related experiences, making it too indirect for our review focus on depression/anxiety. We have updated the PRISMA flow diagram, study characteristics table, synthesis tables, and CERQual assessments accordingly. These exclusions did not materially alter the main interpretation of the review, although some theme wording and study counts were revised. Both studies supported the theme “Growing in the role” and “Hidden cost of caring”.

1. **Growing in the role**

Before removal, Giusto et al. (2021) contributed to sub-theme “Identity status and transformation”, a broader reading of role development by suggesting that LHW role could spill into family and everyday life, while also creating tensions with other work and caregiving responsibilities. After exclusion, the theme became less focused on role expansion across home and community spaces and more clearly centred on the gradual development of counselling skills, confidence, and legitimacy within the helper role itself. We therefore revised the relevant sub-theme to emphasise negotiating legitimacy and identity in the role, rather than a broader account of identity transformation across multiple social settings. This change did not alter the overall interpretation that growth was gradual and experience-driven, but it reduced emphasis on wider role spillover and strengthened alignment with the review question.

Before removing Verhey et al. (2019), the theme was supported by a broader claim that LHWs became not only more skilled, but also more emotionally resilient, respected, and transformed by the role. Verhey et al. (2019) was one of the studies cited in the sub-theme “Progressive skill acquisition and growing clinical confidence,” where the interpretation was that many LHWs became “more confident, autonomous, and respected in their communities.” The older third-order construct also framed the theme in quite expansive terms, referring to confidence, role identity, emotional resilience, improved self-understanding, and enhanced family respect. After removing Verhey, the theme is better expressed as a process of learning, gaining confidence, and negotiating legitimacy in the role, rather than a broad transformation across emotional, family, and community life. What remains much more solidly supported is the theme’s core developmental curve where LHWs begin with uncertainty, rely heavily on manuals and supervision, feel anxious about “doing it right,” and gradually become more confident through training, role-play, feedback, and repeated practice.

**Before excluding Giusto et al. (2021)**

| **First order construct** | **Second order construct** | **Translation** | **Third order construct** |
| --- | --- | --- | --- |
| *The skills I acquired for guiding the clients helped me and my family a lot. For the children, I used the skill of scheduled activities, I picked some which were applicable to me and used it for my children. It was very beneficial. (Giusto et al., 2021)* | Lay counselors all described positive changes in clients. These included decreased drinking, increased acceptance in the community, increased attention to their role in the family including increased trust of partners, acceptance of partners leaving the home to look for work, effort to help caregiving, and increased time at home; and improved family interactions.  Counselors also noticed changes in their own lives as a result of using the skills taught in LEAD; these included more open  communication, improved time management, and increased attention to family. One counselor reported that his interactions and time with family changed (Giusto et al., 2021) | **Identity and status transformation**  While some drew on intervention skills to  support their families, this role expansion  also created tensions as LHWs struggled  to balance counselling work with existing  employment and caregiving obligations  (Giusto et al., 2021).  At the same time, interactions with more educated patients, who sometimes questioned their competence, underscored how LHWs must navigate entrenched social hierarchies that can erode their authority (Atif et al., 2015). Overall, these findings illustrated how LHWs new roles reshape their sense of self and status across home and clinical  Settings | **Growing in the role**  LHWs' experiences across diverse settings reveal that their roles require more than just technical skill, they demand internal changes, such as shifts in confidence, role identity, and emotional resilience. This process of personal growth and professionalisation led to improved self-understanding and enhanced family respect, marking the LHW role as a dynamic and evolving experience, shaped not just by  manuals but by personal and  emotional development |
| *Before we started the program, I faced challenges. One, this is a totally different field... so you can just imagine how difficult it was to balance the two, but along the way I was able to balance and enjoy the counseling. So, the main challenge was balancing between my own work [and the]* needs my family. (Giusto et al., 2021) | When asked for their opinions on implementation, all lay counselors indicated wanting the intervention to be longer to allow for more repetition and more time to help fathers practice the skills. Two counselors also suggested the program involve family members to help fathers stay accountable to reducing drinking and to help monitor activity completion. Counselors also reported preferring to have more sessions per week, more frequent trainings, and more cases to build mastery. However, alongside this, one challenge all counselors noted was balancing their work and family commitments with counselling (Giusto et al., 2021) |  |  |
| *Initially they were not engaging with me. Both were graduates and more educated than me. They made it clear to me that they were more qualified. IDI-15PV (Atif et al, 2015)* | Challenges in working as a peer volunteer > Lack of engagement > Lack of interest (Atif 2015)  The PVs described various reasons which they believed lead to the mothers lack of interest in the intervention. Some PVs felt that lack of familiarity with the talking therapy combined with the lack of material incentives made the intervention less attractive to some mothers. |  |  |

**After excluding Giusto et al. 2021)**

| **First order construct** | **Second order construct** | **Translation** | **Third order construct** |
| --- | --- | --- | --- |
| *I think young people like you [referring to the interviewer] are very talented. Old person like me cannot be talented like you. In fact, people said that they would like to see the old doctor, because they are full of experience, but I think there is no experience because the things they have learnt in the past is different from now. Now when I went to big hospital (….), it is full of talented young doctors, I found that the youth is talented. [SC, Long An] (Chau et al. 2021)* | Challenges for program scalability with social collaborators (Chau et al. 2021)  older age was also associated with difficulties in acquiring the new knowledge required for the intervention. Thus, while age and the related experience was definitely an advantage in encouraging trust with communities, our study also suggests that scaling up the intervention might require the development of training programs that are adapted for older learners | **Negotiating legitimacy and identity in the role**  Chau et al. (2021) show that some LHWs positioned themselves as inferior in relation to age and expertise, particularly when comparing themselves with younger health workers perceived as more talented and modern. Similarly, Atif et al. (2015) show how interactions with more educated patients could undermine LHWs’ confidence and authority, as social and educational hierarchies made them feel less legitimate in the helper role. Taken together, these studies suggest that LHWs’ early experiences were often shaped by self-doubt and by comparisons with more socially valued forms of expertise.  By contrast, Joag et al. (2020) reflects a different position. Here, the role of “Champion” had been internalised as part of the LHW’s sense of self, fostering fearlessness, confidence, and a stronger sense of purpose. Read together, these studies suggest that feelings of inferiority were not merely barriers to practice, but part of a broader process through which LHWs negotiated who they were in relation to the role. Over time, some moved from seeing themselves as inadequate or less authoritative than others toward inhabiting a more confident and purposeful identity as a credible helper. | **Growing in the role**  Across diverse settings, LHWs’ experiences suggest that the role required more than the acquisition of technical skills. It involved an ongoing process of negotiating confidence, legitimacy, and identity in relation to patients, supervision, and wider social hierarchies. Growth was not only about learning how to deliver an intervention, but also about becoming able to inhabit the role of a credible helper despite initial insecurity, self-doubt, or perceived lack of authority. In this sense, the LHW role emerged as a dynamic and evolving experience, shaped by practice, reflection, relational encounters, and changing self-understanding. |
| *Initially they were not engaging with me. Both were graduates and more educated than me. They made it clear to me that they were more qualified. IDI-15PV (Atif et al, 2015)* | Challenges in working as a peer volunteer > Lack of engagement > Lack of interest (Atif  et al, 2015)  The PVs described various reasons which they believed lead to the mothers lack of interest in the intervention. Some PVs felt that lack of familiarity with the talking therapy combined with the lack of material incentives made the intervention less attractive to some mothers. |  |  |
| *I have prevented many widows and separated women from suicidal thoughts. I myself had such thoughts but I overcame them. (I would think) “How can I do such things? I am the Champion of the project!... people will say “She used to counsel us and now she herself had tried this!”. These thoughts changed my mind-set. Now (I) am fearless and I feel confident. (Champ60224M11)* (Joag et al. 2020) | Impact and benefits of the intervention (Joag et al. 2020)  The qualitative in-depth interviews indicated that persons in the community experienced reduced symptoms and improved social, occupational and family functioning for a variety of problems such as depression, domestic violence, alcohol use, and severe mental illness. Employment, and family functioning also improved. These were attributed to a combination of intervention components |  |  |

**Before excluding Verhey et al. (2019)**

| **First order construct** | **Second order construct** | **Translation** | **Third order construct** |
| --- | --- | --- | --- |
| *I live with my mom, and I used to fear making my own decisions before (becoming a CMHW). I would expect others to decide on my behalf, but not anymore. Now I make my own decisions and my mother and family respect them, I only studied until middle school, but now I am determined to major in psychology. I know this (job) made me fall for psychology, and I would not like to stop doing my work. You can help people not only by being a doctor or a nurse, but by being*  *like me, some sort of a psychologist. (Rodríguez-Cuevas et al. 2021)* | Impact in CMHWs and Community Care  (Rodríguez-Cuevas et al., 2021)  CMHWS value the empowerment and noticeable changes in self-confidence that came with the process of becoming a CMHW, and by learning a strategy that could help people living with  mental health conditions | **Progressive skill acquisition and growing clinical confidence**  LHWs also encountered challenges when  dealing with complex cases. Ng’oma et al. (2024) and Kidia et al. (2024) described  situations where LHWs were unsure how to progress with high-need clients and  recognised the need to refer cases beyond  their capacity. These instances illustrate the limits of role adaptability, requiring LHWs to balance persistence with professional judgment. Despite these early struggles, LHWs described a transformation in their roles, both in terms of emotional resilience and skill development. Many became more confident, autonomous, and respected in  their communities (Henrique et al., 2021;  Jacobs et al., 2021; Pacichana-Quinayáz et al., 2016; Rodríguez-Cuevas et al., 2021; Verhey et al., 2019). | **Growing in the role**  LHWs' experiences across diverse settings reveal that their roles require more than just technical skill, they demand internal changes, such as shifts in confidence, role identity, and emotional resilience.  This process of personal growth and professionalisation led to improved self-understanding and enhanced family respect, marking the LHW role as a dynamic and evolving experience, shaped not just by  manuals but by personal and  emotional development. |
| *I have now built a very strong relationship with most of the patients and we now know*  *each other a lot (Verhey et al., 2019)* | **LHWs experience with the FB (Verhey et al.,**  **2019)**  The Ambuya Utano role included visiting  People’s homes to educate about hygiene,  vaccination drives, and health campaigns, and using soft skills such as empathy and loyalty. |  |  |
| *It goes hand in hand. The bench work has given us skills that we did not have before. The Friendship Bench work has improved*  *our impact in the community. (Verhey et al., 2019)* | **Socioeconomic upheaval (Verhey et al., 2019)**  LHWs recognized the impact of their work and wished to be empowered and encouraged to continue |  |  |
| *“It benefitted me because my husband also drinks [alcohol], so … I have given him my [intervention] book. And then it was very helpful, and he was interested …” (ID01, designated counsellor).* (Jacobs et al. 2021) | **Counsellors’ Perceptions of the Project MIND Training (Jacobs et al., 2021)**  Most FBCs described how being trained in MI-PST benefitted them personally. After seeing how the problem-solving method helped their patients, some of these counsellors applied the problem-solving method to resolve their own challenges and assist those around them (e.g. problematic alcohol use among family members). |  |  |
| *“I learned so much about role plays so that we can familiarise how the intervention should be like … from session one to session three.” (ID08, dedicated counsellor).* (Jacobs et al., 2021) | **Counsellors’ Perceptions of the Project MIND Training (Jacobs et al., 2021)**  The FBCs emphasised that the role-playing and skills rehearsal enhanced their confidence in their ability to deliver the MIND intervention. According to these counsellors, the skills rehearsal aspect of training provided them with opportunities to rehearse their new counselling skills while simultaneously familiarising themselves with the manualised nature of the intervention: |  |  |
| *CHW 1: “Of course! I am sure [that a CHW could deliver the intervention]”; CHW 3: “We were well trained; we can do this [intervention*]”. (Henrique et al. 2021) | **Capability of non-specialists (Feeling capable of delivering the intervention) (Henrique et al., 2021)**  In general, the health workers did not experience problems delivering the programme and felt welcomed by the older adult participants. Only one CHW (CHW6) mentioned that the older adult participants she visited, refused to participate in the psychosocial sessions because the CHW was not a mental health specialist |  |  |
| *CHW 8: “Today, even without the tablet and not being part of the intervention programme any longer, we can still offer some counselling to people.”;* (Henrique et al., 2021) | **Impact of the intervention in their work routine at the UBS (Professional impact) (Henrique et al., 2021)**  All health workers indicated that participation in the PROACTIVE programme had a positive impact on their work at the UBS, as they acquired new skills. |  |  |
| *CHW 6- “Everything that we learned, that opened our mind, that helped in our work was valid. Every day we learn something, we change our point of view… This [the intervention programme] came to intensify it, to change our view”.* (Henrique et al., 2021) |  |  |  |
| *I used to worry a lot. when I was doing the intervention then I realised that there are things we worry about that are not important…so it seems like I am helping someone else, and I am also helping myself. (dedicated CHW)* (Myers et al. 2019) | **Perceived benefits of the intervention (Myers et al., 2019)**  In addition, both the dedicated and designated CHWs appeared to benefit personally from this programme, describing how they now applied PST strategies to resolve problems in their own lives and limit negative thoughts |  |  |

**After excluding Verhey et al. (2019)**

| **First order construct** | **Second order construct** | **Translation** | **Third order construct** |
| --- | --- | --- | --- |
| *“When participant had lots of issues, it became difficult to move from one step to another. It took me some time to understand and successfully deliver it to high need participants…” (PV4) (Ng'oma et al. 2024)* | **Perceived feasibility of THPP delivery > Difficult experiences during THPP delivery (Ng’oma et al., 2024)**  A few peer volunteers found challenging to deliver intervention to mothers who were experiencing complex issues and had chronic symptoms while one mother stated that the intervention was complex initially: | **Progressive skill acquisition and growing clinical confidence**  LHWs also encountered challenges when dealing with complex cases. Ng’oma et al. (2024) and Kidia et al. (2024) described situations where LHWs were unsure how to progress with high-need clients and recognized the need to refer cases beyond their capacity. These instances illustrate the limits of role adaptability, requiring LHWs to balance persistence with professional judgment. Despite these early struggles, LHWs described a transformation in their roles, both in terms of emotional resilience and skill development. Many became more confident, autonomous, and respected in their communities (Henrique et al., 2021; Jacobs et al., 2021; Pacichana-Quinayáz et al., 2016; Rodríguez-Cuevas et al., 2021). | **Growing in the role**  Across diverse settings, LHWs’ experiences suggest that the role required more than the acquisition of technical skills. It involved an ongoing process of negotiating confidence, legitimacy, and identity in relation to patients, supervision, and wider social hierarchies. Growth was not only about learning how to deliver an intervention, but also about becoming able to inhabit the role of a credible helper despite initial insecurity, self-doubt, or perceived lack of authority. In this sense, the LHW role emerged as a dynamic and evolving experience, shaped by practice, reflection, relational encounters, and changing self-understanding. |
| *If I have a red flag, I cannot counsel the client. The pressure might be too much for me, so I need help from the nurses who can send the client to the hospital (VHW, female). (Kidia et al. 2024).* | **Clinical workflow and emphasis on documentation (Kidia et al, 2024)**  For nurses, when asked about their supervisory role of VHWs, many mentioned that this role included close monitoring to ensure VHWs are correctly implementing problem solving therapy and taking care of referrals of cases deemed too challenging for the VHWs. |  |  |
| *Every day there was more motivation work because I understood how to manage that component of trauma. [...] Then, because these work, I could use knowledge daily and family life. (Pacichana-Quinayáz et al. 2016)* | **Characteristics and LPCWs performance (Pacichana-Quinayáz et al., 2016)**  According to interviewees, there are some benefits for LPCW including the generation of income and relative economic stability, wellbeing, autonomy and mental health tools that strengthen the skills of individuals to develop their own work and extend the therapy benefits into their daily lives and family life. |  |  |
| *I live with my mom, and I used to fear making my own decisions before (becoming a CMHW). I would expect others to decide on my behalf, but not anymore. Now I make my own decisions and my mother and family respect them… I only studied until middle school, but now I am determined to major in psychology. I know this (job) made me fall for psychology, and I would not like to stop doing my work. You can help people not only by being a doctor or a nurse, but by being like me, some sort of a psychologist.* (Rodríguez-Cuevas et al., 2021) | **Impact in CMHWs and Community Care (Rodríguez-Cuevas et al., 2021)**  CMHWS value the empowerment and noticeable changes in self-confidence that came with the process of becoming a CMHW, and by learning a strategy that could help people living with mental health conditions: |  |  |
| *“It benefitted me because my husband also drinks [alcohol], so … I have given him my [intervention] book. And then it was very helpful, and he was interested …” (ID01, designated counsellor).* (Jacobs et al., 2021) | **Counsellors’ Perceptions of the Project MIND Training (Jacobs et al., 2021)**  Most FBCs described how being trained in MI-PST benefitted them personally. After seeing how the problem-solving method helped their patients, some of these counsellors applied the problem-solving method to resolve their own challenges and assist those around them (e.g. problematic alcohol use among family members). |  |  |
| *“I learned so much about role plays so that we can familiarise how the intervention should be like … from session one to session three.” (ID08, dedicated counsellor).* (Jacobs et al., 2021) | **Counsellors’ Perceptions of the Project MIND Training (Jacobs et al., 2021)**  The FBCs emphasised that the role-playing and skills rehearsal enhanced their confidence in their ability to deliver the MIND intervention. According to these counsellors, the skills rehearsal aspect of training provided them with opportunities to rehearse their new counselling skills while simultaneously familiarising themselves with the manualised nature of the intervention: |  |  |
| *CHW 1: “Of course! I am sure [that a CHW could deliver the intervention]”; CHW 3: “We were well trained; we can do this [intervention*]”. (Henrique et al., 2021) | **Capability of non-specialists (Feeling capable of delivering the intervention) (Henrique et al., 2021)**  In general, the health workers did not experience problems delivering the programme and felt welcomed by the older adult participants. Only one CHW (CHW6) mentioned that the older adult participants she visited, refused to participate in the psychosocial sessions because the CHW was not a mental health specialist |  |  |
| *CHW 8: “Today, even without the tablet and not being part of the intervention programme any longer, we can still offer some counselling to people.”;* (Henrique et al., 2021) | **Impact of the intervention in their work routine at the UBS (Professional impact) (Henrique et al., 2021)**  All health workers indicated that participation in the PROACTIVE programme had a positive impact on their work at the UBS, as they acquired new skills. |  |  |
| *CHW 6- “Everything that we learned, that opened our mind, that helped in our work was valid. Every day we learn something, we change our point of view… This [the intervention programme] came to intensify it, to change our view”.* (Henrique et al., 2021) |  |  |  |
| *I used to worry a lot. when I was doing the intervention then I realised that there are things we worry about that are not important…so it seems like I am helping someone else, and I am also helping myself. (dedicated CHW)* (Myers et al., 2019) | **Perceived benefits of the intervention (Myers et al., 2019)**  In addition, both the dedicated and designated CHWs appeared to benefit personally from this programme, describing how they now applied PST strategies to resolve problems in their own lives and limit negative thoughts |  |  |

2. **Hidden cost of caring**

Before exclusion, Verhey et al. (2019) and Giusto et al. (2021) contributed to The hidden cost of caring in different but complementary ways. Verhey et al. (2019) contributed most directly to the sub-theme that was then framed as “carrying others’ pain while hiding one’s own.” It provided a vivid illustration of the emotional cost of empathy through the quote, *“I have such empathy for these clients… At times I go on the side and cry, but I stand with them.”* This was interpreted as showing the duality of compassion and composure: LHWs were deeply affected by clients’ suffering yet still felt required to remain strong and continue caring. In this way, Verhey et al. (2019) helped foreground emotional concealment, empathic overload, and the invisible work of “holding it together” while delivering support.

By contrast, Giusto et al. (2021) contributed more indirectly, but still meaningfully, to the same theme. Rather than illustrating concealed emotion within counselling encounters, it showed how the demands of care extended beyond the formal session and created tension with other parts of everyday life. It supported two linked ideas: first, that counsellors carried counselling practices into their own families, shaping communication, time management, and family relationships; and second, that they struggled to balance counselling responsibilities with their other work and caregiving commitments. Giusto et al. (2019) therefore strengthened the interpretation that caring could become unbounded and self-consuming, spilling across home, work, and family life.

After excluding both studies, The hidden cost of caring remained conceptually intact, but its emphasis became more integrated. In the earlier synthesis, the theme gave distinct space to the intimate emotional toll of empathy, particularly through Verhey et al. (2019)’s example of privately crying while remaining outwardly composed. Once that study was removed, this dimension was no longer strong enough to stand as a separate sub-theme on its own. Instead, it was incorporated into the broader sub-theme when caring consumes the self. As a result, the revised theme now places less emphasis on a distinct account of emotional concealment and more emphasis on the cumulative costs of care: emotional containment, self-sacrifice, extension of care into personal time, workload strain, and exposure to gendered and stigmatising risks. This revision did not change the overall interpretation that caring carried hidden costs, but it made the theme more coherent and better aligned with the remaining evidence base.

**Before excluding Verhey et al. (2019)**

| **First order construct** | **Second order construct** | **Translation** | **Third order construct** |
| --- | --- | --- | --- |
| *“I can make an excuse to go drink water” (Oratile) and “I had to pretend like I wasn’t going through what [the client] is going through” (Maphisa et al.). (Ahmed et al. 2024)* | Counselor Psychological Wellbeing (Ahmed et al., 2024)  Counselors mentioned ways in which they tried to conceal their emotions during the counseling sessions. These emotionally triggering experiences can make it difcult for counselors to continue with the therapy sessions and may therefore prompt an  unwanted referral to another counselor | **Carrying others pain while hiding**  **one’s own**  Ahmed et al. (2024) described that having to excuse herself I can make an excuse to go drink water as a way to discreetly manage overwhelming emotions during a session. Moreover, the authors shared that despite deeply relating to a client’s situation, she had to pretend like I wasn’t going through what [the client] is going through, suggesting the strain of maintaining emotional distance while internally struggling. This emotional concealment extended beyond individual sessions to the broader  workload. In Wood et al. (2021), a female  LHW expressed how the cumulative toll of travel, counselling, and administrative duties became overwhelming: You are thinking as they are talking it is too much, very horrible. While some of this burden was logistical, she described the  work as emotionally taxing,indicating  that cognitive and emotional fatigue was intrinsic to the counselling role.Verhey et al. (2019) offered a more visceral glimpse into the emotional cost of empathy. One LHW explained: I have such empathy for these clients At times I go on the side and cry, but I stand with them. This statement reflects the duality of compassion and composure, LHWs often feel deeply for their clients, yet must present strength, regulate their own emotions, and continue delivering support | **The hidden cost of caring.**  Across studies, LHWs’ accounts converge on a shared construct in which emotional labour is the invisible engine of task-shifted through shared pain and carrying others pain while hiding one’s own shows how they draw on personal histories of hardship to build trust and intimacy while suppressing their own distress to appear composed, hopeful, and reassuring. Over time, holding others suffering with limited space to express their own creates a cumulative emotional burden that is rarely acknowledged. This hidden cost is intensified by workload and role strain, as LHWs juggle counselling, community duties, and family responsibilities, often stretching time, energy, and emotional resources to breaking point. For psychological care. Caring many women, care is further shaped by gendered risk: navigating harassment, safety concerns, and expectations of modesty and obedience when entering homes, travelling alone, or challenging abusive dynamics. Stigma and cultural religious resistance add another layer, as LHWs absorb rejection, moral judgement, and suspicion during home visits while working to reframe mental health support in locally acceptable terms. Taken together, these subthemes show that task-shifting hinges on LHWs’ willingness to absorb emotional, relational, and sometimes physical risks so that others can access care. The very qualities that make them effective empathy, availability, embeddedness also expose them to harm, making emotional labour a core mechanism through which both care and cost are produced |
| *Because [during counseling] you are thinking as they are talking. And travelling is so much. After doing all this you come to [the facility], you sit and some other work of typing…So it is too much, very horrible. This I did not like…Yes, it is too much, yes. Work is often emotionally taxing. (LHW, Female, 37 years)(Wood et al. 2021)* | Workload (Wood et al., 2021)  Many LHWs spoke of feeling burdened by their workload, while a few said that they felt motivated despite having lots of work. |  |  |
| *I have such empathy for these clients, I always want to help them. At times I go on the side and cry but I stand with them. I push for them to get help because they really touch my heart (Verhey et al., 2019)* | Mechanism (Verhey et al., 2019)  The Ambuya Utano role included visiting people’s homes to educate about hygiene, vaccination drives, and health campaigns, and using soft skills such as empathy and loyalty |  |  |
| *“The challenge is always like coming here and also rescheduling issues… because maybe I will come here knowing my client will be here at two o’clock and then at half past two they will be telling me ‘no, I have family issues’… and then we reschedule to a different date [to] accommodate my client with me now, and I have to make sacrifices for my client because I understand it’s one of the things that we were told about. That sometimes you are going to have difficulties that you are going to invade some of the personal, private time” (Oratile). (Ahmed et al., 2024)* | Scheduling Procedures (Ahmed et al., 2024)  Several counselors implied that the initial procedures used to schedule clients for counseling sessions were barriers to Safe Haven implementation. At the beginning of the pilot, when counselors were scheduling their clients by appointment only, clients were missing their scheduled appointments or attending late |  |  |
| *“I may spend more time on an emotionally stressed mother than a healthier one.”(Burgess et al. 2025)* | **Current approaches to mental health and Family Planning (Burgess et al., 2025)**  HEWs described varying approaches for supporting mothers in their communities who seemed to be struggling emotionally. “The only thing we can do” one HEW explained, “is provide advice and make them feel strong.” Another noted, “I may spend more time on an emotionally stressed mother than a healthier one.” In a case where a mother had been abandoned by her husband, one HEW described collecting funds to support her. Overall, HEW were already well immersed in postpartum mental health and FP through their daily work, but had more tools and training specifically for Family Planning. |  |  |
| *We forgot to take care of ourselves as counsellors. We put a lot of focus on the clients. We have our own problems and need to heal first before we help other people... (Counsellor 4) (Munodawafa et al. 2017)* | **Implementation** (Munodawafa et al. 2017)  Three counsellors mentioned how providing emotional support to mothers reminded them of their own problems and the other three mentioned how counselling participants was emotionally taxing for them. |  |  |

**After excluding Verhey et al. (2019)**

| **First order construct** | **Second order construct** | **Translation** | **Third order construct** |
| --- | --- | --- | --- |
| *“I can make an excuse to go drink water” (Oratile) and “I had to pretend like I wasn’t going through what [the client] is going through” (Maphisa et al.). (Ahmed et al., 2024)* | **Counselor Psychological Wellbeing (Ahmed et al., 2024)**  Counselors mentioned ways in which they tried to conceal their emotions during the counseling sessions. These emotionally triggering experiences can make it difcult for counselors to continue with the therapy sessions and may therefore prompt an  unwanted referral to another counselor | **When caring consumes the self**  LHWs’ accounts suggest that caring often became self-consuming, requiring them to suppress their own emotions during sessions, sacrifice personal time, and repeatedly prioritise clients’ needs over their own wellbeing. Ahmed et al. (2024) show how some LHWs had to conceal their own emotional distress in order to remain composed during counselling. One worker described making an excuse “to go drink water” to manage overwhelming feelings, while also “pretend[ing] like I wasn’t going through what [the client] is going through,” highlighting the strain of maintaining therapeutic presence while holding back personal emotion. This emotional containment was part of a wider pattern in which care extended beyond the session itself. In Wood et al. (2021), a female LHW described the cumulative demands of travel, counselling, and administrative work as overwhelming: “You are thinking as they are talking… it is too much, very horrible,” and characterised the role as “emotionally taxing,” suggesting that emotional and cognitive strain were embedded in the work itself.  This burden also extended into LHWs’ private time and everyday lives. Ahmed et al. (2024) describe how rescheduling appointments because of client cancellations or family problems often meant sacrificing personal time, something one LHW described as expected: “we were told… you are going to invade some of the personal, private time.” The same study also highlighted frustration when clients did not keep to agreed appointment times, with one LHW noting that “waiting an hour for a client is a problem” when other responsibilities also had to be managed. Burgess et al. (2025) similarly illustrated how care expanded in response to patients’ emotional needs, with one worker explaining, “I may spend more time on an emotionally stressed mother than a healthier one.” Over time, this ongoing extension of care could come at the expense of LHWs’ own wellbeing. In Munodawafa et al. (2017), one counsellor reflected that “we forgot to take care of ourselves… we have our own problems and need to heal first,” while in Jacobs et al. (2021), a designated counsellor recalled that caregiving had once led to emotional overload: “I used to take on the whole world.” Together, these accounts show that the hidden cost of caring lay not only in supporting others through distress, but also in how such care gradually eroded boundaries, consumed personal resources, and displaced attention from LHWs’ own needs. | **The hidden cost of caring.**  Across studies, LHWs’ accounts converge on a shared construct in which emotional labour operates as the often-unseen engine of task-shifted psychological care. Caring through shared pain shows how LHWs draw on personal histories of hardship and social proximity to build trust and therapeutic connection. Yet this closeness also carries costs. In when caring consumes the self, LHWs described suppressing their own emotions during sessions, extending care into personal time, and repeatedly prioritising clients’ needs over their own wellbeing. Over time, this combination of emotional containment, personal sacrifice, and self-neglect creates a cumulative burden that is rarely recognised.  This hidden cost is intensified by workload and role strain, as LHWs juggle counselling, community duties, and family responsibilities, often stretching time, energy, and emotional resources to their limits. For many women, care is further shaped by gendered risk: navigating harassment, safety concerns, and expectations of modesty and obedience when entering homes, travelling alone, or challenging abusive dynamics. Stigma and cultural or religious resistance add another layer, as LHWs absorb rejection, moral judgement, and suspicion during home visits while working to make mental health support locally acceptable.  Taken together, these sub-themes suggest that task-shifted psychological care depends not only on the redistribution of therapeutic tasks, but also on LHWs’ willingness to absorb emotional, relational, and sometimes physical risks so that others can access care. The very qualities that make them effective, empathy, availability, and community embeddedness, also expose them to harm. Emotional labour therefore emerges not as a peripheral issue, but as a core mechanism through which both care and cost are produced. |
| *Because [during counseling] you are thinking as they are talking. And travelling is so much. After doing all this you come to [the facility], you sit and some other work of typing…So it is too much, very horrible. This I did not like…Yes, it is too much, yes. Work is often emotionally taxing. (LHW, Female, 37 years)(Wood et al., 2021)* | **Workload** (Wood et al., 2021)  Many LHWs spoke of feeling burdened by their workload, while a few said that they felt motivated despite having lots of work. |  |  |
| *“The challenge is always like coming here and also rescheduling issues… because maybe I will come here knowing my client will be here at two o’clock and then at half past two they will be telling me ‘no, I have family issues’… and then we reschedule to a different date [to] accommodate my client with me now, and I have to make sacrifices for my client because I understand it’s one of the things that we were told about. That sometimes you are going to have difficulties that you are going to invade some of the personal, private time” (Oratile). (Ahmed et al., 2024)* | **Scheduling Procedures (Ahmed et al., 2024)**  Several counselors implied that the initial procedures used to schedule clients for counseling sessions were barriers to Safe Haven implementation. At the beginning of the pilot, when counselors were scheduling their clients by appointment only, clients were missing their scheduled appointments or attending late |  |  |
| *“if we decided that we should meet with the client around one, then the client decides to come around two… it’s a problem because I do have other commitments outside there. So, waiting an hour for a client is a problem.” (Tebogo) (Ahmed et al., 2024)* |  |  |  |
| *“I may spend more time on an emotionally stressed mother than a healthier one.”(Burgess et al. 2025)* | **Current approaches to mental health and Family Planning (Burgess et al., 2025)**  HEWs described varying approaches for supporting mothers in their communities who seemed to be struggling emotionally. “The only thing we can do” one HEW explained, “is provide advice and make them feel strong.” Another noted, “I may spend more time on an emotionally stressed mother than a healthier one.” In a case where a mother had been abandoned by her husband, one HEW described collecting funds to support her. Overall, HEW were already well immersed in postpartum mental health and FP through their daily work, but had more tools and training specifically for Family Planning. |  |  |
| *We forgot to take care of ourselves as counsellors. We put a lot of focus on the clients. We have our own problems and need to heal first before we help other people... (Counsellor 4) (Munodawafa et al. 2017)* | **Implementation** (Munodawafa et al. 2017)  Three counsellors mentioned how providing emotional support to mothers reminded them of their own problems and the other three mentioned how counselling participants was emotionally taxing for them. |  |  |
| *“It is very important, because before I used to take on the whole world. Now, I know that as I care for my client, I must care for myself, because if I do not care for myself, I am going to burn out.” (ID18, designated counsellor). (Jacobs et al., 2021)* | **Counsellors’ Perceptions of Clinical Supervision and Debriefing (Jacobs et al., 2021)**  FBCs also valued the regular debriefing they received as part of their weekly supervision session, particularly in relation to prioritising self-care and preventing burnout. Regular opportunities for debriefing created a “safe space” to discuss difficult patient cases, personal issues that may be impacting on their counselling, issues around self-care, and strategies for coping with stress. |  |  |

**Before excluding Giusto et al. (2019)**

| **First order construct** | **Second order construct** | **Translation** | **Third order construct** |
| --- | --- | --- | --- |
| *“…say for example we are three and the one is being booked for Project MIND, the pressure is on the other two to work for that one.” (ID13, designated counsellor) (Jacobs, 2020)* | **Barriers and facilitators related to the implementation of the project MIND intervention (Jacobs, 2020)**  Designated counsellors expressed competing task demands (misalignment) as a barrier to implementing the project MIND intervention. These FBCs described having to oversee several chronic clubs, perform HIV testing and counselling, and complete their administration duties as some of their core duties. As delivering the project MIND intervention was an addition to their daily duties, these FBCs felt that the task demands were often too much to perform all their duties optimally. | **When the work never ends**  Several studies revealed that LHWs experienced significant workload strain as a result of integrating psychological interventions into their already full schedules. In Jacobs (2020), designated counsellors described how limited staffing meant the absence of one team member led to additional burdens on others: we are three and the one is being booked for Project MIND, the pressure is on the other two. Similarly, these LHWs juggled dual responsibilities delivering Project MIND while continuing to assist in psychiatric clinics highlighting blurred boundaries between intervention roles and routine duties. Jacobs et al. (2021) further illustrated that while delivering an hour-long session felt meaningful, it also resulted in accumulated pressure, as colleagues had to compensate by attending to 20-30 other patients. Henrique et al. (2021) echoed this tension: while the programme was rewarding, LHWs acknowledged it was unexpectedly time-consuming and created conflict with their pre-existing tasks, described as an extra burden and a source of friction in day-to-day operations.  Burgess et al. (2025) showed that LHWs were already responsible for an extensive array of services, vaccination, family planning, first aid, and health education. The psychological programme, while seen as valuable, added to the complexity and volume of their duties. This convergence of responsibilities points to role overload and fatigue, suggesting that implementation demands must be balanced with feasible workloads for sustainable service delivery. | **The hidden cost of caring.**  Across studies, LHWs’ accounts converge on a shared construct in which emotional labour is the invisible engine of task-shifted through shared pain and carrying others pain while hiding one’s own shows how they draw on personal histories of hardship to build trust and intimacy while suppressing their own distress to appear composed, hopeful, and reassuring. Over time, holding others suffering with limited space to express their own creates a cumulative emotional burden that is rarely acknowledged. This hidden cost is intensified by workload and role strain, as LHWs juggle counselling, community duties, and family responsibilities, often stretching time, energy, and emotional resources to breaking point. For psychological care. Caring many women, care is further shaped by gendered risk: navigating harassment, safety concerns, and expectations of modesty and obedience when entering homes, travelling alone, or challenging abusive dynamics. Stigma and cultural religious resistance add another layer, as LHWs absorb rejection, moral judgement, and suspicion during home visits while working to reframe mental health support in locally acceptable terms. Taken together, these subthemes show that task-shifting hinges on LHWs’ willingness to absorb emotional, relational, and sometimes physical risks so that others can access care. The very qualities that make them effective empathy, availability, embeddedness also expose them to harm, making emotional labour a core mechanism through which both care and cost are produced |
| *“…On Wednesdays I am busy with mental illness [psychiatric clinic] with the psychiatrist and doctor…whereby I do not focus on Project MIND alone, but I also participate and assist also on the psychiatrist and doctor that is here.” (ID08, dedicated counsellor) (Jacobs, 2020)* | **Barriers and facilitators related to the implementation of the project MIND intervention > Inner setting (Jacobs et al., 2021)**  Dedicated counsellors reported enjoying their work and being able to focus on delivering counselling without feeling pressured to perform other tasks (alignment). During times when they were not busy counselling patients, they offered their assistance to other areas in the facility such as helping healthcare staff or administrative personnel |  |  |
| *“At the beginning it was fine but as time went by it was such a lot of people we had to see and we had to see our work as well. So, it was tiring, because an hour to sit with a person is a lot when you know my colleagues need to see now maybe 20 or 30 more people because I am busy in this project.” (ID11, designated counsellor). (Jacobs et al., 2021)* | **Perceived Barriers and Facilitators to the Implementation of the Project MIND Intervention (Jacobs et al., 2021)**  Designated counsellors expressed concerns about the degree to which they could align the delivery of mental health counselling with their current workflow. They described competing task demands (including the management of chronic clubs which involves health promotion, provision of HIV testing and counselling, and other administrative duties) and uncertainty about how to reorganise their work schedule to accommodate MIND counselling. Several FBCs felt overwhelmed by these multiple task demands and needed help to prioritise their other work demands so that they could perform their duties optimally.. |  |  |
| *“Yes, because it was an extra burden, wasn’t it? Our usual tasks never stopped, but it [the programme] was something, it left a good taste in my mouth…”. (Henrique et al., 2021)*  *“Much more, much more time than we have imagined... But it was good”; CHW 7: “I didn’t know it was going to be that much”. (Henrique et al., 2021)* | **Impact of the intervention in their work routine at the UBS (Institutional impact) (Henrique et al., 2021)**  The extra work associated with participating in  the PROACTIVE programme was a hot topic during the focus groups. The main complaint was related to the difficulty of managing the time to perform the routine tasks at the UBS with psychosocial sessions at home, all within working hours. The home sessions were added to their other tasks at the UBS. They also felt the time spent with the PROACTIVE programme (sessions and supervision) was more than what they expected initially. Health workers also complained about frictions with colleagues over the time they spent on the PROACTIVE programme. |  |  |
| *There were frictions, you know? Because we did not do other things, we ended up taking that time to do these other things we had not planned… So, there were divergences* (Henrique et al., 2021) | **Workload and responsibilities (Burgess et al. 2025)**  In the first interview, prior to Mothers Time training, HEWs were asked to describe their current workload or responsibilities. They described long days, busy schedules, including lots of engagement with pregnant women and women who recently delivered. “I provide services like vaccines, family planning, monitoring, first aid and health education” said one HEW, explaining that, “the program is vast and huge and has many activities within it.” One key responsibility for HEWs is engaging with women at multiple key touchpoints before and after a birth, providing services and counseling on breastfeeding, vaccines, infant health, FP, and more. “We see a mother seven or eight times” one HEW explained, noting that through this long-term engagement, “we can establish a strong relationship.” |  |  |
| *“I provide services like vaccines, family planning, monitoring, first aid and health education”“the program is vast and huge and has many activities within it.” (Burgess et al. 2025)* | *In the first interview, prior to Mothers Time training, HEWs were asked to describe their current workload or responsibilities. They described long days, busy schedules, including lots of engagement with pregnant women and women who recently delivered. “I provide services like vaccines, family planning, monitoring, first aid and health education” said one HEW, explaining that, “the program is vast and huge and has many activities within it.” One key responsibility for HEWs is engaging with women at multiple key touchpoints before and after a birth, providing services and counseling on breastfeeding, vaccines, infant health, FP, and more. “We see a mother seven or eight times” one HEW explained, noting that through this long-term engagement, “we can establish a strong relationship.”* |  |  |
| *“I may spend more time on an emotionally stressed mother than a healthier one.” (Burgess et al. 2025)* |  |  |  |
| *Before we started the program, I faced challenges. One, this is a totally different field... so you can just imagine how difficult it was to balance the two, but along the way I was able to balance and enjoy the counseling. So, the main challenge was balancing between my own work [and the] needs my family. (Giusto et al., 2021)* | **Acceptability (Giusto et al., 2021)**  When asked for their opinions on implementation, all lay counselors indicated wanting the intervention to be longer to allow for more repetition and more time to help fathers practice the skills. Two counselors also suggested the program involve family members to help fathers stay accountable to reducing drinking and to help monitor activity completion. Counselors also reported preferring to have more sessions per week, more frequent trainings, and more cases to build mastery. However, alongside this, one challenge all counselors noted was balancing their work and family commitments with counseling |  |  |

**After excluding Giusto et al. (2021)**

| **First order construct** | **Second order construct** | **Translation** | **Third order construct** |
| --- | --- | --- | --- |
| *“…say for example we are three and the one is being booked for Project MIND, the pressure is on the other two to work for that one.” (ID13, designated counsellor) (Jacobs, 2020)* | **Barriers and facilitators related to the implementation of the project MIND intervention (Jacobs, 2020)**  Designated counsellors expressed competing task demands (misalignment) as a barrier to implementing the project MIND intervention. These FBCs described having to oversee several chronic clubs, perform HIV testing and counselling, and complete their administration duties as some of their core duties. As delivering the project MIND intervention was an addition to their daily duties, these FBCs felt that the task demands were often too much to perform all their duties optimally. | **When the work never ends**  Several studies revealed that LHWs experienced significant workload strain as a result of integrating psychological interventions into their already full schedules. In Jacobs (2020), designated counsellors described how limited staffing meant the absence of one team member led to additional burdens on others: “we are three and the one is being booked for Project MIND, the pressure is on the other two.” Similarly, these LHWs juggled dual responsibilities delivering Project MIND while continuing to assist in psychiatric clinics highlighting blurred boundaries between intervention roles and routine duties.  Jacobs et al. (2021) further illustrated that while delivering an hour-long session felt meaningful, it also resulted in accumulated pressure, as colleagues had to compensate by attending to 20–30 other patients. Henrique et al. (2021) echoed this tension: while the programme was rewarding, LHWs acknowledged it was unexpectedly time-consuming and created conflict with their pre-existing tasks, described as an “extra burden” and a source of “friction” in day-to-day operations.  Burgess et al. (2025) showed that LHWs were already responsible for an extensive array of services, vaccination, family planning, first aid, and health education. The psychological programme, while seen as valuable, added to the complexity and volume of their duties. This convergence of responsibilities points to role overload and fatigue, suggesting that implementation demands must be balanced with feasible workloads for sustainable service delivery. | **The hidden cost of caring.**  Across studies, LHWs’ accounts converge on a shared construct in which emotional labour operates as the often-unseen engine of task-shifted psychological care. Caring through shared pain shows how LHWs draw on personal histories of hardship and social proximity to build trust and therapeutic connection. Yet this closeness also carries costs. In when caring consumes the self, LHWs described suppressing their own emotions during sessions, extending care into personal time, and repeatedly prioritising clients’ needs over their own wellbeing. Over time, this combination of emotional containment, personal sacrifice, and self-neglect creates a cumulative burden that is rarely recognised.  This hidden cost is intensified by workload and role strain, as LHWs juggle counselling, community duties, and family responsibilities, often stretching time, energy, and emotional resources to their limits. For many women, care is further shaped by gendered risk: navigating harassment, safety concerns, and expectations of modesty and obedience when entering homes, travelling alone, or challenging abusive dynamics. Stigma and cultural or religious resistance add another layer, as LHWs absorb rejection, moral judgement, and suspicion during home visits while working to make mental health support locally acceptable.  Taken together, these sub-themes suggest that task-shifted psychological care depends not only on the redistribution of therapeutic tasks, but also on LHWs’ willingness to absorb emotional, relational, and sometimes physical risks so that others can access care. The very qualities that make them effective, empathy, availability, and community embeddedness, also expose them to harm. Emotional labour therefore emerges not as a peripheral issue, but as a core mechanism through which both care and cost are produced. |
| *“…On Wednesdays I am busy with mental illness [psychiatric clinic] with the psychiatrist and doctor…whereby I do not focus on Project MIND alone, but I also participate and assist also on the psychiatrist and doctor that is here.” (ID08, dedicated counsellor) (Jacobs, 2020)* | **Barriers and facilitators related to the implementation of the project MIND intervention > Inner setting (Jacobs et al., 2021)**  Dedicated counsellors reported enjoying their work and being able to focus on delivering counselling without feeling pressured to perform other tasks (alignment). During times when they were not busy counselling patients, they offered their assistance to other areas in the facility such as helping healthcare staff or administrative personnel |  |  |
| *“At the beginning it was fine but as time went by it was such a lot of people we had to see and we had to see our work as well. So, it was tiring, because an hour to sit with a person is a lot when you know my colleagues need to see now maybe 20 or 30 more people because I am busy in this project.” (ID11, designated counsellor). (Jacobs et al., 2021)* | **Perceived Barriers and Facilitators to the Implementation of the Project MIND Intervention (Jacobs et al., 2021)**  Designated counsellors expressed concerns about the degree to which they could align the delivery of mental health counselling with their current workflow. They described competing task demands (including the management of chronic clubs which involves health promotion, provision of HIV testing and counselling, and other administrative duties) and uncertainty about how to reorganise their work schedule to accommodate MIND counselling. Several FBCs felt overwhelmed by these multiple task demands and needed help to prioritise their other work demands so that they could perform their duties optimally.. |  |  |
| *“Yes, because it was an extra burden, wasn’t it? Our usual tasks never stopped, but it [the programme] was something, it left a good taste in my mouth…”. (Henrique et al., 2021)*  *“Much more, much more time than we have imagined... But it was good”; CHW 7: “I didn’t know it was going to be that much”. (Henrique et al., 2021)* | **Impact of the intervention in their work routine at the UBS (Institutional impact) (Henrique et al., 2021)**  The extra work associated with participating in  the PROACTIVE programme was a hot topic during the focus groups. The main complaint was related to the difficulty of managing the time to perform the routine tasks at the UBS with psychosocial sessions at home, all within working hours. The home sessions were added to their other tasks at the UBS. They also felt the time spent with the PROACTIVE programme (sessions and supervision) was more than what they expected initially. Health workers also complained about frictions with colleagues over the time they spent on the PROACTIVE programme. |  |  |
| *There were frictions, you know? Because we did not do other things, we ended up taking that time to do these other things we had not planned… So, there were divergences* (Henrique et al., 2021) | **Workload and responsibilities (Burgess et al. 2025)**  In the first interview, prior to Mothers Time training, HEWs were asked to describe their current workload or responsibilities. They described long days, busy schedules, including lots of engagement with pregnant women and women who recently delivered. “I provide services like vaccines, family planning, monitoring, first aid and health education” said one HEW, explaining that, “the program is vast and huge and has many activities within it.” One key responsibility for HEWs is engaging with women at multiple key touchpoints before and after a birth, providing services and counseling on breastfeeding, vaccines, infant health, FP, and more. “We see a mother seven or eight times” one HEW explained, noting that through this long-term engagement, “we can establish a strong relationship.” |  |  |
| *“I provide services like vaccines, family planning, monitoring, first aid and health education”“the program is vast and huge and has many activities within it.” (Burgess et al. 2025)* | *In the first interview, prior to Mothers Time training, HEWs were asked to describe their current workload or responsibilities. They described long days, busy schedules, including lots of engagement with pregnant women and women who recently delivered. “I provide services like vaccines, family planning, monitoring, first aid and health education” said one HEW, explaining that, “the program is vast and huge and has many activities within it.” One key responsibility for HEWs is engaging with women at multiple key touchpoints before and after a birth, providing services and counseling on breastfeeding, vaccines, infant health, FP, and more. “We see a mother seven or eight times” one HEW explained, noting that through this long-term engagement, “we can establish a strong relationship.”* |  |  |

**References**

**Ahmed CV, Van Pelt AE, Buttenheim AM, Poku O, Rice BM, Lowenthal ED and Brooks MJ** (2024) Implementation Determinants of Problem-Solving Therapy Delivered by Near-Peer Lay Counselors for Youth Living with HIV in Botswana: Lay Counsellor Perspectives. *Global Implementation Research and Applications* **4**(4)**,** 381-393. <https://doi.org/10.1007/s43477-024-00126-6>.

**Atif N** (2015) The acceptability of peer volunteers as delivery agents of a psychosocial intervention for perinatal depression in rural Pakistan: a qualitative study. Student thesis: PhD, University of Manchester.

**Burgess S, Bitew T, Haile A, Souffrant J, Shattuck D, Van Lith LM, Moore J and Hendrickson ZM** (2025) The feasibility and acceptability of implementing simplified cognitive behavioral therapy approaches to support postpartum mental health and address associated social and behavioral barriers to postpartum family planning in Amhara, Ethiopia: a qualitative study. *BMC Women's Health* **25**(1)**,** 248. <https://doi.org/10.1186/s12905-025-03744-w>.

**Chau LW, Murphy J, Nguyen VC, Lou H, Khanh H, Thu T, Minas H and O’Neil J** (2021) Lay social workers implementing a task-sharing approach to managing depression in Vietnam. *International Journal of Mental Health Systems* **15**(1)**,** 52. <https://doi.org/10.1186/s13033-021-00478-8>.

**Giusto A, Johnson SL, Lovero KL, Wainberg ML, Rono W, Ayuku D and Puffer ES** (2021) Building community-based helping practices by training peer-father counselors: A novel intervention to reduce drinking and depressive symptoms among fathers through an expanded masculinity lens. *International Journal of Drug Policy* **95,** 103291. <https://doi.org/https://doi.org/10.1016/j.drugpo.2021.103291>.

**Henrique MG, de Paula Couto MCP, Araya R, Mendes AV, Nakamura CA, Hollingworth W, van de Ven P, Peters TJ and Scazufca M** (2021) Acceptability and fidelity of a psychosocial intervention (PROACTIVE) for older adults with depression in a basic health unit in São Paulo, Brazil: a qualitative study. *BMC Public Health* **21**(1)**,** 2278. <https://doi.org/10.1186/s12889-021-12402-3>.

**Jacobs Y, Myers B, van der Westhuizen C, Brooke-Sumner C and Sorsdahl K** (2021) Task Sharing or Task Dumping: Counsellors Experiences of Delivering a Psychosocial Intervention for Mental Health Problems in South Africa. *Community Mental Health Journal* **57**(6)**,** 1082-1093. <https://doi.org/10.1007/s10597-020-00734-0>.

**Joag K, Shields-Zeeman L, Kapadia-Kundu N, Kawade R, Balaji M and Pathare S** (2020) Feasibility and acceptability of a novel community-based mental health intervention delivered by community volunteers in Maharashtra, India: the Atmiyata programme. *BMC Psychiatry* **20**(1)**,** 48. <https://doi.org/10.1186/s12888-020-2466-z>.

**Kidia K, Machando D, Dzoro V, Chibanda D, Abas M, Manda E, Mutengerere A, Nyandoro T, Chawarika M, Majichi D, van Dijk JH and Jack H** (2024) Rural Friendship Bench: A qualitative study in Zaka district, Zimbabwe. *Social Science & Medicine* **348,** 116791. <https://doi.org/https://doi.org/10.1016/j.socscimed.2024.116791>.

**Munodawafa M, Lund C and Schneider M** (2017) A process evaluation exploring the lay counsellor experience of delivering a task shared psycho-social intervention for perinatal depression in Khayelitsha, South Africa. *BMC Psychiatry* **17**(1)**,** 236. <https://doi.org/10.1186/s12888-017-1397-9>.

**Myers B, Petal P-W, Claire van der W, Crick L, Carl L, John AJ, Naomi SL, Christopher B, Tracey N, Peter M, Dan JS and Katherine S** (2019) Community health worker-delivered counselling for common mental disorders among chronic disease patients in South Africa: a feasibility study. *BMJ Open* **9**(1)**,** e024277. <https://doi.org/10.1136/bmjopen-2018-024277>.

**Ng'oma M, Atif N, Meltzer-Brody S, Chirwa E and Stewart RC** (2024) Piloting a psychosocial intervention for perinatal depression, the Thinking Healthy Programme-Peer delivered (THPP), in a primary care setting in Lilongwe District, Malawi. *PLOS Glob Public Health* **4**(5)**,** e0002128. <https://doi.org/10.1371/journal.pgph.0002128>.

**Pacichana-Quinayáz SG, Osorio-Cuéllar GV, Bonilla-Escobar FJ, Fandiño-Losada A and Gutiérrez-Martínez MI** (2016) Common Elements Treatment Approach based on a Cognitive Behavioral Intervention: implementation in the Colombian Pacific. *Cien Saude Colet* **21**(6)**,** 1947-1956. <https://doi.org/10.1590/1413-81232015216.07062015>.

**Rodríguez-Cuevas FG, Valtierra-Gutiérrez ES, Roblero-Castro JL and Guzmán-Roblero C** (2021) Living Six Hours Away from Mental Health Specialists: Enabling Access to Psychosocial Mental Health Services Through the Implementation of Problem Management Plus Delivered by Community Health Workers in Rural Chiapas, Mexico. *Intervention Journal of Mental Health and Psychosocial Support in Conflict Affected Areas* **19**(1).

**Verhey R, Chibanda D, Vera A, Manda E, Brakarsh J and Seedat S** (2019) Perceptions of HIV-related trauma in people living with HIV in Zimbabwe’s Friendship Bench Program: A qualitative analysis of counselors’ and clients’ experiences. *Transcultural Psychiatry* **57**(1)**,** 161-172. <https://doi.org/10.1177/1363461519850337>.

**Wood S, Seevak E, Bhatia U, McBain R and Nadkarni A** (2021) “I will not step back”: A Qualitative Study of Lay Mental Health Workers’ Experiences in India. *Administration and Policy in Mental Health and Mental Health Services Research* **48**(4)**,** 695-706. <https://doi.org/10.1007/s10488-020-01102-6>.
