# Supplementary material for: Delivering low-intensity psychological interventions in low- and middle-income countries: A systematic review and meta-ethnographic synthesis of lay health workers’ experiences
Source: Glob Ment Health (Camb). 2026 Jul 7;13:e157. doi: 10.1017/gmh.2026.10271 (PMC13433339; doi:10.1017/gmh.2026.10271)
Supplement: Peer review history

# Reviewer report: Review: Delivering low-intensity psychological interventions in low- and middle-income countries: A systematic review and meta-ethnographic synthesis of lay health workers’ experiences — R0/PR2

**DOI:** 10.1017/gmh.2026.10271.pr2

**Authors:** Arvin Bhana

**Affiliations:** University of KwaZulu-Natal College of Health Sciences, South Africa

A well-written and well-structured MS.

The background to task sharing is informative. What appears to be lacking in this review is the extent to which LHWs are part of a formalized arrangement of providing task-shared mental health services relative to those based outside of formalized care. In the Myers et al. (2019) study, this is described as dedicated and designated CHWs. This distinction is important, as the emotional support and supervision required by LWHs is likely more available within an organized labor arrangement than an ad hoc, external arrangement. Is there any evidence that LHW services that are part of an existing health system are likely to provide better emotional support to them? The Selohilwe et al. (2023) reference makes a similar reference to the integration of counselors in healthcare facilities. If this data is unavailable, the discussion and recommendations for future studies could include suggestions of integrating LHWs into existing support systems to mitigate the emotional labor experienced.

The example provided of the synthesis process is a valuable addition to providing clarity about how the data was analyzed.

Confidentiality of information is important. The name of an institution appears on p. 25, lines 14-15. It should be redacted.

# Reviewer report: Review: Delivering low-intensity psychological interventions in low- and middle-income countries: A systematic review and meta-ethnographic synthesis of lay health workers’ experiences — R0/PR3

**DOI:** 10.1017/gmh.2026.10271.pr3

This paper addresses a very important issue, with implications for both policy and practice in the global mental health field. The authors explain clearly their rationale for the study, and make a strong case for its relevance.

The Introduction section is strong. It refers to relevant research, and is written in a clear and concise way. The Methodology section is also clearly written and has an appropriate level of detail. There are some points I suggest the authors address, either in the Introduction or in the Methodology, to clarify the scope of their study.

Firstly, in relation to the low-intensity psychological interventions (LIPIs), it would be helpful to have some more details about how they define this term and what kind of approaches are included and excluded. The focus on depression and anxiety implies that the authors are focusing on LIPIs which aim to reduce symptoms of diagnosed mental health problems (or problems which reach a diagnosable level of severity). Yet many LIPIs, including PM+ and SH+, are transdiagnostic and can be used with people who do not necessarily meet the criteria for depression or anxiety. I would be interested to know why the authors focus on depression and anxiety in particular, rather than the high levels of distress which most LIPIs are intended to address. In the Results section (Table 3) it would be helpful to include the LIPI which is the focus of each study.

Secondly, some more clarity around the definition of lay health workers, as used in this study, would also be helpful. Much of the task shifting in the global mental health field relates to non-mental health workers also including this in their roles (e.g. community health workers, nurses in primary health care centres). The term ‘lay’ is sometimes used to indicate workers in other fields who have no background in mental health. In the context of this paper, it seems this is not the group which is focused on. Instead, the authors are focusing on (I think) people who not only have no background in mental health, but also no background in general health. It would be useful to include a paragraph in the Intro to make this clear and explain who these people tend to be (e.g. natural helpers in a community), how they are identified and what their roles and responsibilities might be. It would also be useful to include a column in Table 3 outlining the characteristics of the LHWs beyond gender and age. This has relevance for how the findings are interpreted, and how they can influence policy and practice.

The Discussion section is well written. The authors make clear the limitations of the findings, especially in relation to geographical representation. The additional features of the studies mentioned above (type of LIPI and type of LHW) would also strengthen the authors’ ability to reflect on the implications of the findings.

The relevance of the findings for practice are stated clearly, and are well grounded in the findings.

# Reviewer report: Review: Delivering low-intensity psychological interventions in low- and middle-income countries: A systematic review and meta-ethnographic synthesis of lay health workers’ experiences — R0/PR4

**DOI:** 10.1017/gmh.2026.10271.pr4

**Authors:** Liana Chase

This review makes a valuable contribution to knowledge. It provides a clear and well-structured synthesis of research on an understudied topic: the impacts on LHWs of delivering psychological interventions in LMIC. The methodology is robust and well-described. The interpretations seem sound and well-supported with examples. The conclusions and recommendations derived from this work have the potential to positively influence practice in the field of global mental health. I wholeheartedly recommend publication following a few issues are addressed:

• Impact Statement and Discussion: Whilst I am in strong agreement with the author’s argument about the importance of recognizing and providing training and support around LWHs’ emotional labour, I was surprised that the impact statement and discussion seemed to frame this as the main/primary solution to hidden care costs and burnout. Two other points seem equally important: recognizing and scaffolding the relational labour LHWs do and improving working conditions for LHWs. Without the latter, there is a risk of implying that we need to build LHWs’ capacity to stoically tolerate or ‘manage’ their own suffering rather than addressing the structural conditions of labour in this field that clearly exacerbate this.

• Review Parameters: I would be curious to hear a little more justification for the exclusive focus on psychological interventions for depression and anxiety. This seems to rule out a range of LHW-delivered interventions that are transdiagnostic or ‘psychosocial’ in nature.

• Discussion: A number of critical scholars have made points similar to yours drawing on ethnographic research on LHW interventions that did not meet your inclusion criteria. It might be helpful to engage with their work at least in the discussion section: Leocata et al.’s work on ‘when the trial ends’, Kottai and Ranganathan’s work on task-shifting in Kerala, and Chase’s work on lay counselling in Nepal (notes on translation, valuing community workers, and the double-edged sword of community). A systematic review of the literature on community health workers (Ahmed et al. 2021) reflects that similar conversations about hidden costs of care have been happening in the wider global health arena for some years.

• The term ‘collectivist cultures’ strikes me as somewhat outdated and crude; some further explanation or nuance here would be helpful.

• Supplementary material might be more extensive than necessary and suffers from formatting issues; I defer to the editor’s guidance on this.

# Reviewer report: Review: Delivering low-intensity psychological interventions in low- and middle-income countries: A systematic review and meta-ethnographic synthesis of lay health workers’ experiences — R0/PR5

**DOI:** 10.1017/gmh.2026.10271.pr5

**Authors:** Dristy Gurung

**Affiliations:** School of Medicine and Health Sciences, George Washington Elementary School, United States

This is a timely and policy-relevant review and meta-ethnographic synthesis that makes an important contribution by foregrounding the often-overlooked emotional and relational labour involved in delivering mental healthcare in LMICs by community/lay health workers. However, there are several areas that would benefit from clarification and strengthening before publications

Major Comments:

Methods:

- It would be helpful for readers if authors list specific review questions that guided their review process, analysis and results. Currently, the question “What are the experiences of LHWs delivering these interventions in LMICs?” is too vague and has been reviewed/reported well in past publications. It doesn’t clarify what was looked at specifically and why this review was necessary, considering the number of other publications exploring similar themes.

- Search strategy: Were there any study date restrictions?

- IRR process: 0.30 and 0.40 Kappa is quite low- comment on how the authors moved forward with conflicts even with low IRRs among reviewers.

- The included studies seem quite limited, given the volume of publications from countries such as Uganda, Nigeria, Nepal, and Bangladesh on this topic. This could be due to screening discrepancies resulting from low IRR or issues with search strategies. Additionally, date restrictions are not mentioned. It would be good if authors could comment on the limitations of their review process. I would also encourage authors to discuss more explicitly how the uneven distribution of studies affects the transferability of the findings and the extent to which the conclusions can be presented as globally applicable.

- I am confused how the authors were able to extract qualitative data/experiences of LHWs only relevant to depression/anxiety when they included papers that had information from both common and severe mental disorders? A lot of the first-order participants’ quotes that the authors have included could be about any condition and not specific to depression and anxiety. Could the authors explain more about how this was done? This clarity is specifically important since the broader inclusions of other disorders may have shaped some of the themes around “doing more than psychological therapy” and “hidden costs of caring”.

- While the authors have quite aptly synthesized the cultural and personal relationship of lay health workers in providing community care, I think the discussion would be strengthened if the authors could also include a gendered and moral perspective in their argument. Please refer to the studies below that have argued about these issues in task sharing in community-based mental healthcare in Nepal.

o Chase L. The double-edged sword of ‘community’ in community-based psychosocial care: reflections on task-shifting in rural Nepal. Anthropology & Medicine. 2023 Jul 3;30(3):294-309.

o Chase LE, Gurung D, Shrestha P, Rumba S. Gendering psychosocial care: risks and opportunities for global mental health. The Lancet Psychiatry. 2021 Apr 1;8(4):267-9.

Minor comments

- The characteristics table is useful, but it may help distinguish more clearly which studies were purely qualitative vs mixed-methods with extractable qualitative components.

- A slightly clearer explanation of what authors mean by “low-intensity psychological interventions” across different contexts would improve readability, especially because the included interventions appear quite heterogeneous.

- The supplementary material 3 in the current enclosed format is unreadable.

# Reviewer report: Review: Delivering low-intensity psychological interventions in low- and middle-income countries: A systematic review and meta-ethnographic synthesis of lay health workers’ experiences — R0/PR6

**DOI:** 10.1017/gmh.2026.10271.pr6

Delivering low-intensity psychological interventions in low and middle-income countries: a systematic review and meta-ethnographic synthesis of lay health workers’ experiences

D GMH-2026-0056

Thank you for the opportunity to review this paper. This study reviews the qualitative literature regarding the experiences of lay health workers delivering mental health and psychosocial support (MHPSS) interventions in low- and middle-income country (LMIC) settings. As the authors note, this remains an important yet underexplored dimension of task-shifting research. The paper and its subsequent analysis make a meaningful contribution to our understanding of the lived experiences of lay health workers, moving well beyond the more technical and economic arguments typically advanced in the task-sharing literature.

The study is clearly and thoroughly described. The background is sufficient and well-grounded, the methodology is rigorous, and the themes are thoughtfully constructed and well-rounded. The discussion effectively synthesizes the key findings and draws out meaningful implications for lay health workers, the broader MHPSS field, and future research directions. It was genuinely a pleasure to read. I have a few minor suggestions for revision that I believe would further strengthen the paper.

Comments:

Synthesis: Please provide a bit more detail in the synthesis section to enhance understanding of the analytic process. Include examples of the key themes for first- and second-order constructs. What is meant by reciprocal translation in this context.

Pg 21/new header. On page 21, I believe “findings” would be a better header for this section and remove duplication with the earlier section “synthesis” which elaborates the analysis process.

Very thoughtful and engaging discussion. Please also comment on the extent to which you see new projects (E.g., EQUIP) which focuses on development of core competency scales across intervention types, addressing some of the gaps you observed in your study.

In addition to future research attending to the impact and regulation of emotional labor among lay health workers, I think you are well positioned to comment on broader structural systems and the extent to which they contribute to such “unpaid” emotional labor and/or organizational and workforce development strategies that are needed to support and bolster community providers.

Future research may also be advised to investigate provider wellbeing alongside intervention studies and your team may be well-positioned to advise on the methodologies as well as the development/adaptation/existence of such measures to support such investigation.

# Editor report: Recommendation: Delivering low-intensity psychological interventions in low- and middle-income countries: A systematic review and meta-ethnographic synthesis of lay health workers’ experiences — R0/PR7

**DOI:** 10.1017/gmh.2026.10271.pr7

**Affiliations:** Partners In Health, United States

Dear Authors,

Thank you for your submission. The following comments reflect themes identified across peer review and additional editorial observations.

1. The appendix tables are illegible and need to be reformatted. 

2. Provide a working definition for lay health workers and apply it consistently throughout

3. Provide a definition and rationale for “low-intensity intervention.”

4. Similar to point 2, CBT is used as a search term and treated operationally as a component of low-intensity intervention, but does not appear as a formal inclusion or exclusion criterion. Please either (a) add CBT as an explicit inclusion or exclusion criterion, or (b) add a column to the study summary table capturing intervention type/technique (e.g. CBT, problem-solving therapy, stress management) and intended treatment target. This is a request to restructure how extracted data is presented in the table, not a request to revisit inclusion criteria or rescreen studies. 

5. The column in Table 3, labeled ‘Patients’, would be better relabeled as ‘Treatment Target’ or ‘Intervention Target’. 

6. Please provide justification for focusing solely on anxiety and depression.

7. Several included studies addressed conditions beyond depression & anxiety (e.g., substance use, more severe conditions), which risks skewing the data presented (as one reviewer aptly pointed out). If the authors used a distinct process for discerning or extracting qualitative data specific to populations delivering depression- or anxiety-targeted interventions, please describe it in detail. Otherwise, please consider either excluding these studies from the qualitative synthesis or acknowledging them as a substantive limitation with implications for interpretation.

8. In the methods for screening, the IRR is low. Please confirm whether double screening was conducted and acknowledge IRR as a limitation regardless. 

9. For clarity, please put in the results section the number of articles excluded at titles & abstract screening, and the number excluded at full text, to aptly represent the PRISMA chart.

10. The results report that 35 publications meeting the inclusion criteria were collapsed to 28 unique studies after accounting for materials in multiple formats—please reflect this in the PRISMA chart. 

11. The authors cite Kohrt 2018 and aptly call for training and supervision that extends beyond technical fidelity, including competencies that focus on empathy, trust building, and communication. The latest global standard and competency frameworks & tools for training and supervision that address this directly is the WHO/UNICEF EQUIP (Kohrt et al., Lancet Psychiatry, 2025;12(1):67–80, https://doi.org/10.1016/S2215-0366(24)00183-4) and the foundational heling skills curriculum (Pedersen et al, GMH, 10, e55, https://doi.org/10.1017/gmh.2023.43). It may be useful to consider reframing the discussion to reflect the latest global standards for the training & supervision of lay health workers, and potentially refine the core argument being made in relation to this recommendation. 

12. The concern raised regarding search dates has been reviewed and is not considered a required revision.

# Editor report: Decision: Delivering low-intensity psychological interventions in low- and middle-income countries: A systematic review and meta-ethnographic synthesis of lay health workers’ experiences — R0/PR8

**DOI:** 10.1017/gmh.2026.10271.pr8

**Affiliations:** Johns Hopkins University Bloomberg School of Public Health, United States

No accompanying comment.

# Reviewer report: Review: Delivering low-intensity psychological interventions in low- and middle-income countries: A systematic review and meta-ethnographic synthesis of lay health workers’ experiences — R1/PR10

**DOI:** 10.1017/gmh.2026.10271.pr10

Thankyou for your careful consideration and response to the reviewers' comments. In my opinion, the paper is much improved as a result, and I am happy to recommend publication.

# Reviewer report: Review: Delivering low-intensity psychological interventions in low- and middle-income countries: A systematic review and meta-ethnographic synthesis of lay health workers’ experiences — R1/PR11

**DOI:** 10.1017/gmh.2026.10271.pr11

**Authors:** Arvin Bhana

**Affiliations:** University of KwaZulu-Natal College of Health Sciences, South Africa

Lay health workers come in “all shapes and sizes.” Definitional criteria are therefore a likely minefield. My impression is that the literature does not always provide a clear indication of their training, roles, and how they fit into the larger health system. It may be useful to underscore that this challenge is difficult to easily tease apart. The distinction between specialist and non-specialist is fine, but it’s the diversity of lay health worker characteristics themselves that poses a challenge. The authors are clearly mindful of these challenges and include them in their Discussion (p. 33).

# Reviewer report: Review: Delivering low-intensity psychological interventions in low- and middle-income countries: A systematic review and meta-ethnographic synthesis of lay health workers’ experiences — R1/PR12

**DOI:** 10.1017/gmh.2026.10271.pr12

**Authors:** Liana Chase

I am satisfied that the authors have addressed my concerns in the revised version of the article.

# Editor report: Recommendation: Delivering low-intensity psychological interventions in low- and middle-income countries: A systematic review and meta-ethnographic synthesis of lay health workers’ experiences — R1/PR13

**DOI:** 10.1017/gmh.2026.10271.pr13

**Affiliations:** Partners In Health, United States

The response to reviews was well considered and the manuscript is much improved. It’s great to see how much the peer review process enhanced the paper, including the refinement of criteria leading to exclusion of two articles. The supplementary material related to this is well noted and appreciated. To ensure readers are clear at the methods step, please move your related description on this process from the results into the methods, under “selection process”, and in addition to the text you have, please note that the criteria were operationally refined after initial screening, which led to x # of studies returning to full text screening for reappraisal. Then coninute with the rest of the statement you have “two studies were exluded…with author contact” and the reference to the suppl file. 2. Also please add this step in the PRISMA table, the re-review step and the 2 additional exclusions should appear as a distinct node with a footnote, rather than absorbed within the orginal exlcusion numbers. 

minor grammatical and punction fixes needed.

# Editor report: Decision: Delivering low-intensity psychological interventions in low- and middle-income countries: A systematic review and meta-ethnographic synthesis of lay health workers’ experiences — R1/PR14

**DOI:** 10.1017/gmh.2026.10271.pr14

**Affiliations:** Johns Hopkins University Bloomberg School of Public Health, United States

No accompanying comment.

# Editor report: Recommendation: Delivering low-intensity psychological interventions in low- and middle-income countries: A systematic review and meta-ethnographic synthesis of lay health workers’ experiences — R2/PR16

**DOI:** 10.1017/gmh.2026.10271.pr16

**Affiliations:** Partners In Health, United States

No accompanying comment.

# Editor report: Decision: Delivering low-intensity psychological interventions in low- and middle-income countries: A systematic review and meta-ethnographic synthesis of lay health workers’ experiences — R2/PR17

**DOI:** 10.1017/gmh.2026.10271.pr17

**Affiliations:** Johns Hopkins University Bloomberg School of Public Health, United States

No accompanying comment.
